# Synthesis of Tetra-*ortho*-Methoxylated Azobenzene Photoswitches via Sequential Catalytic C–H Activation and Methoxylation

**DOI:** 10.1021/acs.joc.4c01554

**Published:** 2024-11-08

**Authors:** Albert Ruiz-Soriano, Lara Lamelza, Elena Pizzamiglio, Xavier Just-Baringo

**Affiliations:** Laboratori de Química Orgànica, Facultat de Farmàcia, Universitat de Barcelona, 08028 Barcelona, Spain

## Abstract

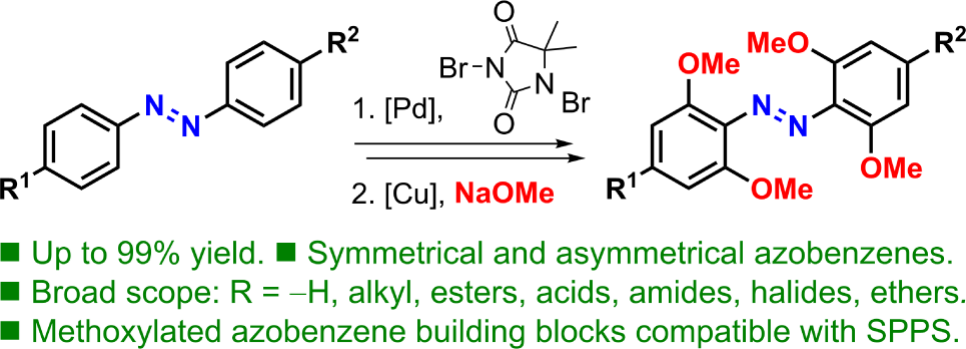

Functionalized tetra-*ortho*-methoxyazobenzenes have been prepared in a two-step approach based on palladium-catalyzed C–H *ortho* bromination of azobenzenes, followed by copper-catalyzed methoxylation. The method has shown a broad tolerance to different functional groups that could not be incorporated by previous strategies. With this two-step transition metal-catalyzed strategy, we achieved overall yields that range from good to excellent and enable the exploitation of these highly coveted photoswitches. The superior robustness of this scaffold for solid phase peptide synthesis (SPPS) applications when compared to its chlorinated counterpart has been demonstrated after extensive treatments with piperidine while bound to a RinkAmide ChemMatrix resin, showcasing their potential for use in the synthesis of red-light-operated peptides.

Modulating the properties of molecules at will with light has been a highly appealing goal for a very long time, as photons do not generate waste and can be delivered with high spatiotemporal precision.^1^ In order to achieve this control, several photoswitchable scaffolds have been developed over the years. However, azobenzenes have been the most common choice for the development of a myriad of applications using small molecules, biomolecules^2^ and materials.^3^ The multiple synthetic methods available and the ease of tuning their properties by incorporating different substituents have made azobenzenes a very popular motif.

Photopharmacology^4^ deals with the study of light-regulated molecules for medical use and has made extensive use of azobenzenes to develop new therapeutic strategies and imaging techniques. In this regard, avoiding the use of UV light for the operation of azobenzenes has been in the focus to avoid its harmful consequences to living tissue.^5^ Thus, there has been a huge effort during the last years to develop substituted azobenzenes that can be isomerized using wavelengths within the biological window, which ranges from the red region of the visible spectrum to the near-infrared and grants both safety and deeper tissue penetration. On this regard, tetra-*ortho*-chlorinated, tetra-*ortho*-fluorinated and tetra-*ortho*-methoxylated azobenzenes have received the most attention and several approaches to their syntheses have been reported, which can be classified into three main strategies: 1) oxidative dimerization of anilines;^6^ 2) addition of *ortho*-lithiated species to diazonium salts;^7^ and 3) palladium-catalyzed C–H *ortho*-functionalization.^8^ The oxidative dimerization of anilines is limited to the preparation of symmetrical products and the presence of *ortho*-functionalization severely lowers the yields (Scheme 1A).^6^ Alternatively, the addition of *ortho*-lithiated reagents to diazonium salts provides better yields, albeit with a narrower substrate scope due to the harsh reagents needed that limit functional group tolerance.^7^ Finally, the palladium-catalyzed C–H *ortho*-methoxylation reaction was severely hampered by a reduced scope and very low yields.^8^ Hence, a more general method for the synthesis of functionalized tetra-*ortho*-methoxylated azobenzenes has remained elusive.

**Scheme 1:**
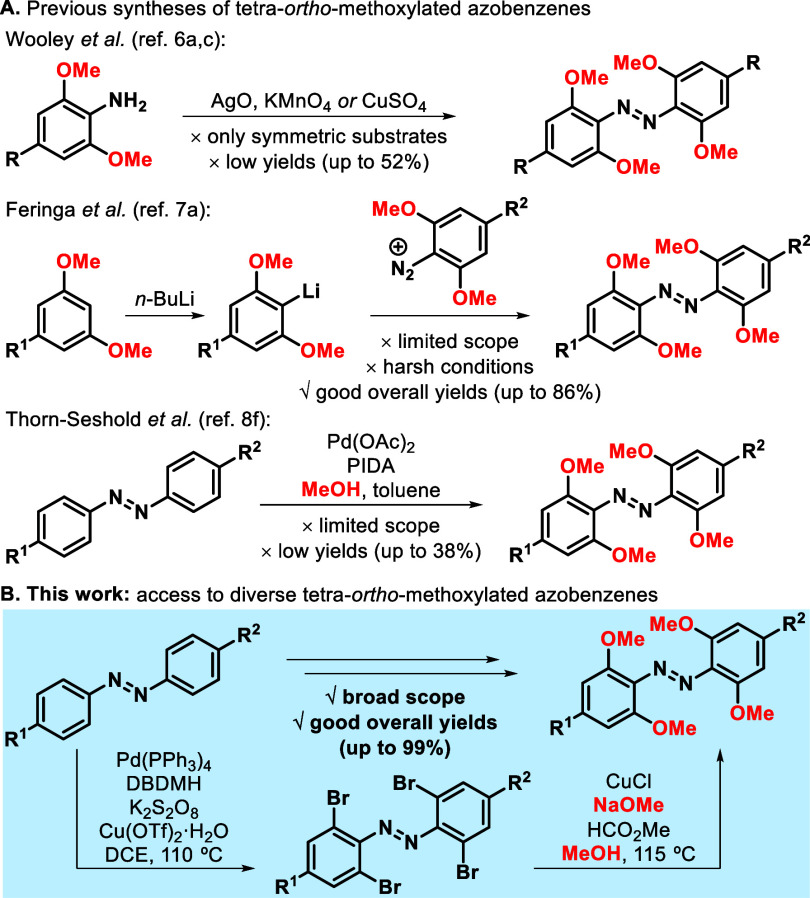
Methods for the Synthesis of Tetra-*ortho*-Methoxyazobenzenes

One of the advantages of tetra-*ortho*-methoxylated azobenzenes is the weaker leaving group character of methoxide when compared to fluoride and chloride. Indeed, the use of tetra-*ortho*-halogenated azobenzene amino acids in Solid Phase Peptide Synthesis (SPPS) has been hampered by their reactivity with piperidine during the Fmoc deprotection steps, leading to nucleophilic aromatic substitution byproducts (*vide infra*). This has been reported by Vázquez and coworkers when using a tetra-*ortho*-fluorinated azobenzene amino acid^6c^ and also limited the use of a tetra-*ortho*-chloroazobenzene amino acid. In order to access the desired substrates, we embarked in the development of a two-step approach toward tetra-*ortho*-methoxylated azobenzenes that relied in the palladium-catalyzed C–H bromination of azobenzenes and their subsequent methoxylation under copper catalysis (Scheme 1B).^9^

## Results and Discussion

We first focused on the development of a general method for the C–H bromination of azobenzenes (Table 1). Xia and coworkers recently reported a protocol that faced several scope restrictions.^8d^ For instance, the reported conditions are not compatible with benzylic positions, which we could confirm in our laboratory and can attribute to the undesired benzylic bromination of the substrates (see Scheme S1).^10^ This is in agreement with the proposed formation of bromine radicals that would be responsible for the observed side reactivity, which we could prevent using Cu(OTf)_2_ as a cocatalyst.^11^ Additionally, we found that the addition of H_2_O is essential.

**Table 1:**
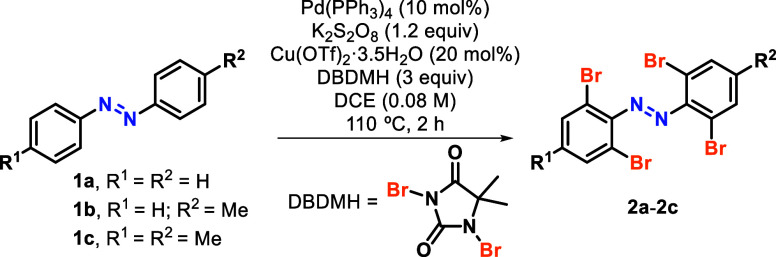
Optimization of the Palladium-Catalyzed C–H *ortho*-Bromination of Azobenzenes^a^

Entry	SM	Variation from optimized conditions	Yield (%)^b^
1	**1a**	none	99
2	**1a**	40 mol % Cu(OTf)_2_·3.5H_2_O instead of 20 mol %	92
3	**1a**	5 mol % Pd(PPh_3_)_4_ instead of 10 mol %	86
4	**1a**	Pd(OAc)_2_ instead of Pd(PPh_3_)_4_	50
5	**1a**	No Pd(PPh_3_)_4_	0
6	**1a**	No Cu(OTf)_2_. H_2_O added separately	90
7	**1a**	No K_2_S_2_O_8_	87
8	**1a**	Anhydrous Cu(OTf)_2_ and DCE. Run under N_2_	0
9	**1b**	none	75
10	**1b**	40 mol % Cu(OTf)_2_·3.5H_2_O instead of 20 mol %	60
11	**1c**	none	67
12	**1c**	40 mol % Cu(OTf)_2_·3.5H_2_O instead of 20 mol %	77

a Reactions run with **1a**–**c** (0.20 mmol) and nondried DCE in a tube sealed under air.

b Isolated yields.

We started screening the Pd catalyst (Table 1, entries 1–5) and observed that Pd(PPh_3_)_4_ outperforms Pd(OAc)_2_ (entry 4) and that reducing the Pd loading to 5% gave a lower yield (entry 3). DBDMH (1,3-dibromo-5,5-dimethylhydantoin) was used as a bromine source and proved to be a very reliable and stable reagent. Importantly, the reaction did not occur at all in the absence of palladium (entry 5), indicating that copper is not a competent catalyst for this directed C–H activation reaction. During this optimization process, we also determined that the presence of H_2_O is essential for the reaction to proceed, as the expected product was never obtained under strictly anhydrous conditions (entry 8).^12^ Interestingly, removal of either Cu(OTf)_2_ (entry 6) or K_2_S_2_O_8_ (entry 7) did not result in huge drops in yield for the bromination of **1a**. Nonetheless, best results were obtained when all reagents were present (entry 1). These optimized conditions also tamed the reactivity of the intermediate bromine species to allow a selective C–H *ortho*-bromination of azobenzenes in the presence of benzylic positions (entries 9–12), furnishing products **2b** and **2c** in very good yields. It is worth noting that for substrates with free *para*-positions (**1a** and **1b**), Cu(OTf)_2_ loadings higher than 20 mol % led to detectable amounts of *para*-bromination. Thus, 20 mol % Cu(OTf)_2_ was used for substrates bearing unsubstituted *para*-positions, whereas 40 mol % was used for 4,4′-disubstituted substrates to achieve higher yields as well as reducing the extent of benzylic bromination in benzylic substrates (see Scheme S1).^10^ With a reliable C–H bromination method in hand, we embarked in the development of a methoxylation reaction of tetra-*ortho*-brominated azobenzenes. To the best of our knowledge, the methoxylation of halogenated arenes under copper catalysis has never been reported in azobenzenes.^13^ A previous attempt of methoxylating *ortho*-halogenated azobenzenes offered a poor perspective as super stoichiometric amounts of copper were used and provided poor results for the substitution of more than one halogen .^14^ However, after a quick optimization of the reaction conditions, we obtained very good results (Table 2). When interrogating the reaction, we found that a slight excess base was optimal to efficiently promote the transformation (entries 1 and 2). Using a larger excess of either sodium methoxide (entry 3) or methyl formate (entry 4),^13b^ resulted in reduced yields in both cases. Moreover, when using copper(II) salts, the reaction did not perform to the same standard (entries 5 and 6). Interestingly, in the absence of a Cu source, some background reaction was still observed, but resulted in a very poor yield (entry 7). Importantly, when testing these optimal conditions on a tetra-*ortho*-chlorinated analogue the reaction failed to provide the methoxylated product **3a** (see Scheme S4).^10^

**Table 2:**
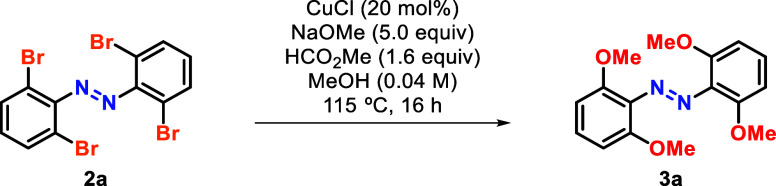
Optimization of the Copper-Catalyzed Methoxylation of Tetra-*ortho*-Brominated Azobenzene^a^

Entry	Variation from optimized conditions	Yield (%)^b^
1	none	100
2	2.5 equiv NaOMe instead of 5.0 equiv	55
3	6.5 equiv NaOMe instead of 5.0 equiv	79
4	3.2 equiv HCO_2_Me instead of 1.6 equiv	82
5	CuCl_2_ instead of CuCl	80
6	Cu(OTf)_2_ instead of CuCl	66
7	No CuCl	18

a Reactions run with **2a** (0.16 mmol).

b Isolated yields.

With optimized conditions for both steps in hand, we explored the scope of substrates, including symmetrical and asymmetrical azobenzenes and a broad range of functionalities. Indeed, unsubstituted azobenzene gave quantitative conversion to the desired product **3a** (Table 3). With regard to the C–H activation step, good to excellent yields were obtained in the presence of different substituents, including alkyl groups (**2b** and **2c**), halogens (**2e**-**2g**), carboxylic acids (**2i** and **2k**) and esters (**2k**-**2m**). Only in the case of ether **2d**, we found that the reaction results in decomposition if Cu(OTf)_2_ is present, and it is necessary to carry out the bromination in its absence, albeit with the separate addition of H_2_O. The main limitation found while exploring the scope of the reaction was its incompatibility with the Boc protecting group. Nonetheless, the trifluoroacetyl group could be used instead to protect amines with very good results (**2n**-**2p**).^8e^ We next moved to assessing the methoxylation reaction with the previously obtained tetra-*ortho*-brominated azobenzenes and found that in most cases the functionalities were well tolerated, although a benzylic amine derivative (**3o**) was obtained in lower yield. However, all other substrates provided the expected products in moderate to excellent yields. For instance, alkyl and ether groups gave excellent results (**3b**-**d**). Halogenated azobenzenes were also well-tolerated (**3e**-**g**), although a lower reaction temperature was required for brominated and iodinated substrates to avoid methoxylation of the *para* positions. Benzylic and propionic acids, as well as esters, gave the desired products with very good yields (**3i**-**m**). Amines protected as trifluoroacetamides were also compatible and yielded the corresponding tetra-methoxylated azobenzenes in moderate to good yields (**3n**-**p**). It is worth noting that previous examples of tetra-*ortho*-methoxylated azobenzenes bearing amide groups had only been obtained with overall yields under 1%.^6ac^ Moreover, **3a**, **3b** and **3m** have only been reported once in 38%, 6% and 18% yield, respectively.^8f^ However, with this new protocol, these are obtained in overall yields of 99%, 65% and 69% from the same precursors. Most importantly, this strategy has allowed us to prepare a wide range of unprecedented tetra-*ortho*-methoxylated azobenzenes (**3c**, **3e**, **3h**-**3l** and **3n**-**3p**).

**Table 3:**
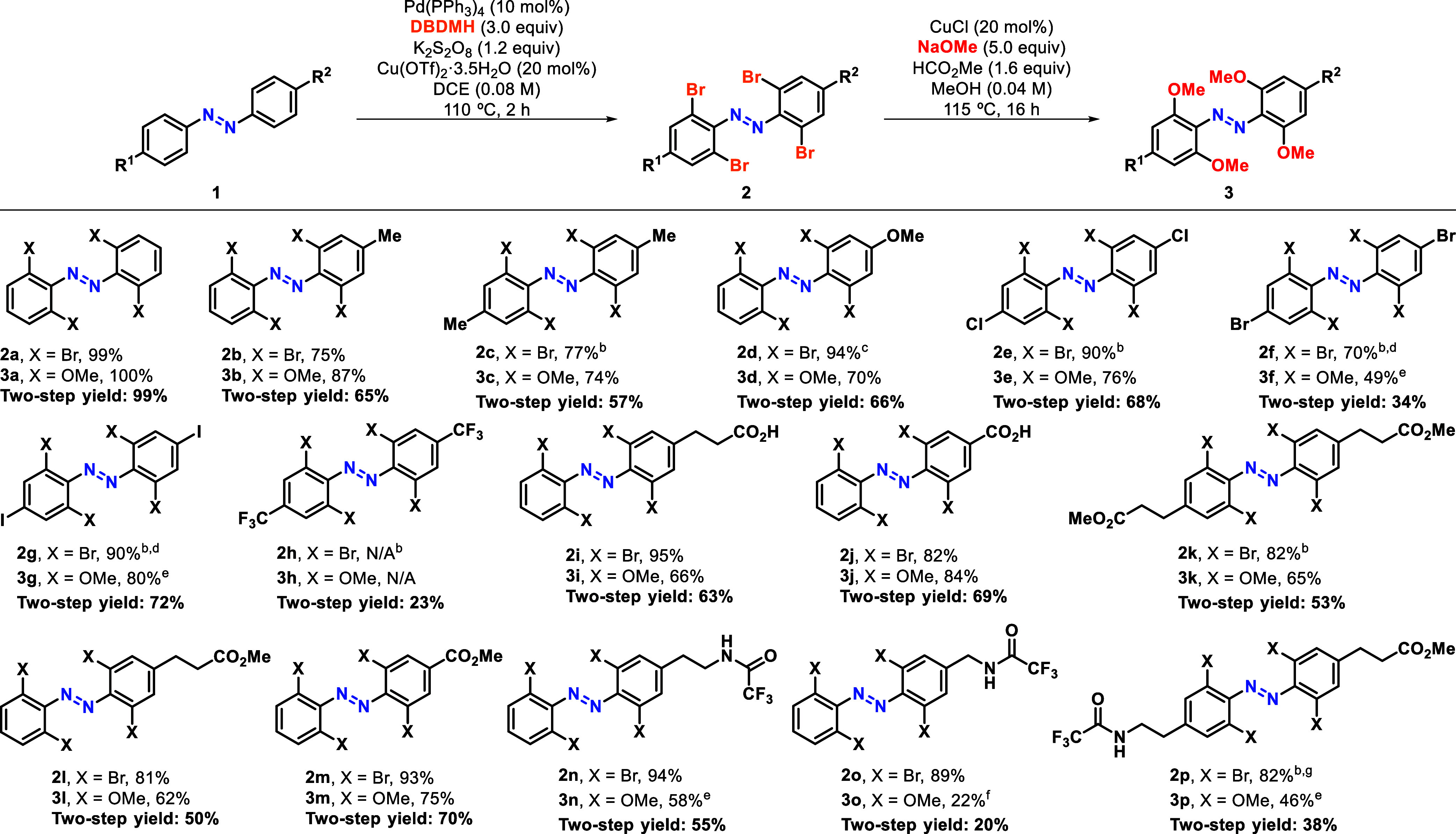
Scope of Palladium-Catalyzed C–H Bromination and Subsequent Copper-Catalyzed Methoxylation of Azobenzenes to Yield Tetra-*ortho*-Methoxylated Azobenzenes^a^^b^^c^^d^^e^^f^

a All yields are isolated. Brominations run at 0.59 mmol scale and methoxylations at 0.16 mmol scale.

b Reaction run with Cu(OTf)_2_·3.5H_2_O (40 mol %).

c Reaction run without Cu(OTf)_2_ and with added H_2_O (3.5 equiv).

d Reaction run for 16 h.

e Reaction run at 90 °C.

f Reaction run at 70 °C. ^g^Reaction run at 2.06 mmol scale.

Photocharacterization of all tetra-*ortho*-methoxylated azobenzenes revealed that electron-poor azobenzenes showed a higher population of the *Z* isomer at the photostationary state (PSS) after illumination with red light (650 nm) (Table 4).

**Table 4:**
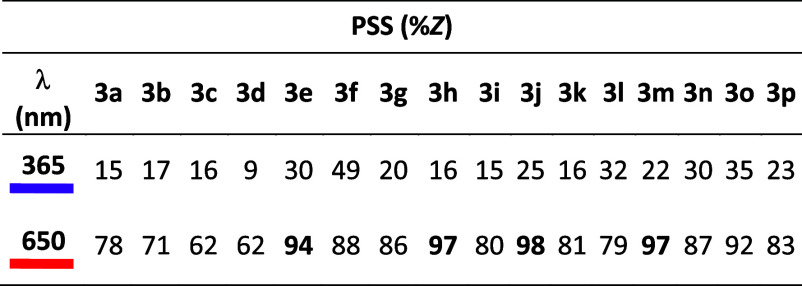
Photostationary State Isomer Ratios of Tetra-*ortho*-Methoxylated Azobenzenes^a^

a Ratios determined by HPLC, reading at an isosbestic point (ca. 450 nm).

**Scheme 2:**
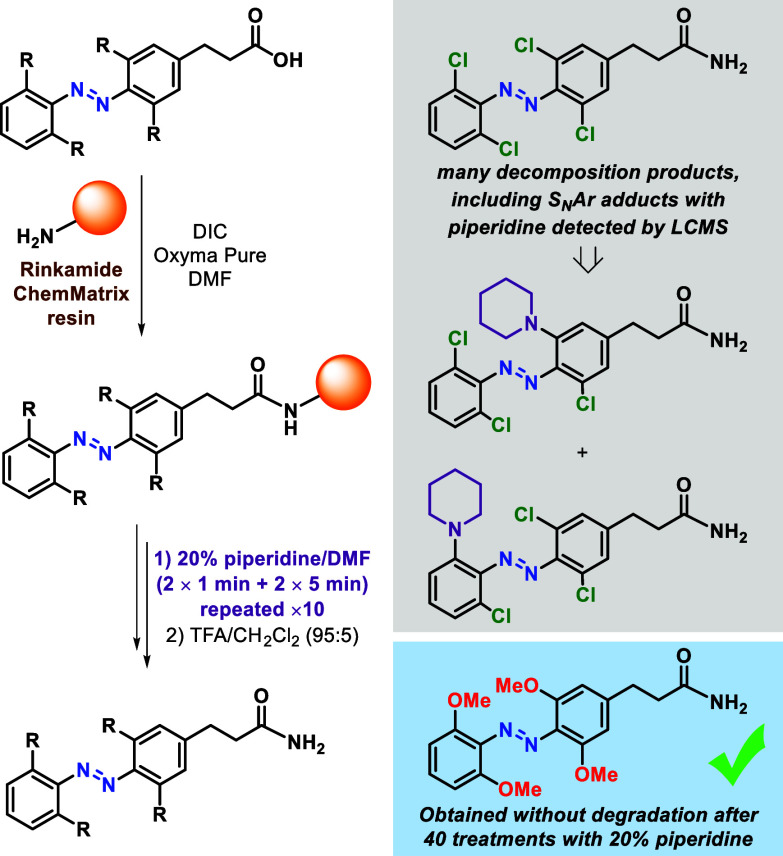
Solid Phase Peptide Synthesis (SPPS) Robustness Test of Tetra-*ortho*-Substituted Azobenzenes

Next, we assessed the robustness of these methoxylated azobenzenes when compared to their chlorinated analogues. In order to do so, propionic acid derivatives bearing the different tetra-*ortho*-substituted azobenzenes were bound to RinkAmide ChemMatrix^15^ resin and subsequently treated repeatedly with piperidine to mimic the most common Fmoc-deprotection conditions used during SPPS (Scheme 2). In total, 10 cycles of the usual deprotection protocol (2 × 1 min + 2 × 5 min) using 20% piperidine in DMF were performed, adding to a total of 40 treatments with piperidine. To our delight, the methoxylated azobenzene recovered after cleavage was identical to the one obtained in a control experiment where only attachment to the resin and subsequent cleavage were performed (see Figure S3).^10^ As expected, the chlorinated analogue showed new species that had formed during the piperidine treatments, among which, S_N_Ar products of chloride substitution with piperidine were detected (see Figure S2). However, many of the newly formed byproducts could not be identified. The susceptibility of tetra-*ortho*-fluorinated building blocks toward S_N_Ar under similar conditions has also been reported.^6c^ This result paves the way to the development of a general protocol for the synthesis of azobenzene-containing visible-light-operated peptides using standard SPPS conditions.

In conclusion, the synthesis of a broad range of tetra-*ortho*-methoxyazobenzenes has been achieved using a two-step protocol with good to excellent overall yields. The sequence involves the initial palladium and copper catalyzed C–H bromination of azobenzene and subsequent copper(I) catalyzed methoxylation of the brominated adducts. Overall, this strategy grants access to a large variety of functionalities that will facilitate the study and application of these scaffolds. Moreover, mechanistic studies on the palladium-catalyzed C–H bromination reaction of azobenzenes have shed light on its requirements and has allowed expanding its substrate scope by using a copper(II) cocatalyst. Finally, the superior robustness of *ortho*-methoxylated azobenzenes for SPPS applications when compared to their halogenated analogues has been demonstrated by repeated treatments under conventional Fmoc-deprotection conditions using piperidine. Applications in peptide synthesis incorporating tetra-methoxylated azobenzene building blocks are currently underway in our laboratory.

## Data Availability

The data underlying this study are available in the published article and its Supporting Information.

## Supporting Information Available

Full optimization tables, control experiments, proposed mechanism, detailed synthetic procedures, NMR spectra, HPLC-MS analysis of SPPS robustness test, UV/vis spectra, and photostationary states analysis (PDF)
