# Supplementary material for: Synthesis of Tetra-ortho-Methoxylated Azobenzene Photoswitches via Sequential Catalytic C–H Activation and Methoxylation
Source: J Org Chem. 2024 Nov 8;89(23):17141–6. doi: 10.1021/acs.joc.4c01554 (PMC11629381; doi:10.1021/acs.joc.4c01554)
Supplement: Supplementary file 1 — jo4c01554_si_001.pdf [file jo4c01554_si_001.pdf]

Synthesis of Tetra-*ortho*-Methoxylated Azobenzene Photoswitches via Sequential Catalytic C−H Activation and Methoxylation

Albert Ruiz-Soriano, Lara Lamelza, Elena Pizzamiglio, Xavier Just-Baringo*

Laboratori de Química Orgànica, Facultat de Farmàcia, Universitat de Barcelona, 08028 Barcelona (Spain)
*e-mail: xavier.just@ub.edu

Table of Contents

**1.**	**General Information** ……………………	S1
**2.**	**Experimental Section** ……………………	S2
**2.1.**	**Palladium-Catalysed C−H Bromination** ……………………	S2
2.1.1.	Full Optimization Table ……………………	S2
2.1.2.	Amount of Water in Hydrated Copper(II) Triflate ……………………	S3
2.1.3.	Control Experiments on the Effect of $Cu(OTf)_2$ ……………………	S4
2.1.4.	Radical Scavenging ……………………	S5
2.1.5.	Proposed Mechanism ……………………	S5
**2.2.**	**Copper-catalysed Methoxylation** ……………………	S6
2.2.1.	Full Optimization Table ……………………	S6
2.2.2.	Control reaction with tetra-*ortho*-chloroazobenzene ……………………	S6
2.2.3.	Radical Scavenging ……………………	S7
**2.3.**	**Synthesis and Characterization** ……………………	S7
**3.**	**NMR Spectra** ……………………	S33
**4.**	**Solid Phase Peptide Synthesis (SPPS) Robustness Test** ……………………	S121
**4.1.**	**Tetra-*ortho*-Chlorinated Azobenzene Robustness Test** ……………………	S122
**4.2.**	**Tetra-*ortho*-Methoxylated Azobenzene Robustness Test** ……………………	S123
**5.**	**Photocharacterisation** ……………………	S124
**5.1**	**UV-Vis Spectra of Photostationary States (PSS)** ……………………	S124
**5.2**	**Isomer Ratios** ……………………	S128

1. General Information

All solvents and reagents used were purchased from commercial suppliers and used without further purification. ^{1}H-NMR spectra were obtained at room temperature on a Bruker 400 MHz spectrometer. ^{13}C-NMR spectra were obtained at 100 MHz. All NMR spectra were processed using MestReNova NMR software. Chemical shifts are reported in parts per million (ppm) and coupling constants (J) are reported in Hz. Splitting patterns are reported as follows: singlet (s), doublet (d), triplet (t), quadruplet

(q), quintuplet (quint), doublet of doublets (dd), doublet of doublets of doublets (ddd), multiplet (m), etc. NMR signals were assigned using the appropriate 2D NMR experiments (*i.e.* HSQC and/or HMBC when necessary). HPLC analysis was performed on a Waters Alliance 2695 separation module coupled to a Waters 2996 photodiode detector (PDA) and to an electrospray ionization source Waters ACQUITY QDa detector, using the MassLynx 4.1 software for data acquisition and a XSelect CSH C18 OBD column. The flow rate was 0.6 $mL \cdot min^{-1}$, and MeCN (0.1% formic acid) and H_2O (0.1% formic acid) were used as solvents. The elution runtime was 3.5 min at 50 °C. All manipulations between irradiations and analysis by HPLC-MS or UV-Vis were carried out in a dark room. TLC analysis was carried out on aluminium sheets coated with silica gel and visualized using UV light. High-resolution mass spectrometry (HRMS) using ESI-TOF and low-resolution EI-MS experiments were performed by Unitat de Cromatografia de Gasos-Espectrometria de Masses Aplicada, Centres Científics i Tecnològics de la Univeristat de Barcelona (CCiTUB). Irradiation with red light (650 nm, 369 $W \cdot m^{-2}$) was performed using a PAUL red LED lamp by GenIUL, S. L. Irradiation with UV light (365 nm) was performed with a TLC visualization lamp.

2. Experimental Section

2.1. Palladium-Catalysed C−H Bromination

2.1.1. Full Optimization Table

Table S1. Optimization of the palladium-catalysed C−H *ortho*-bromination of azobenzene.[a]

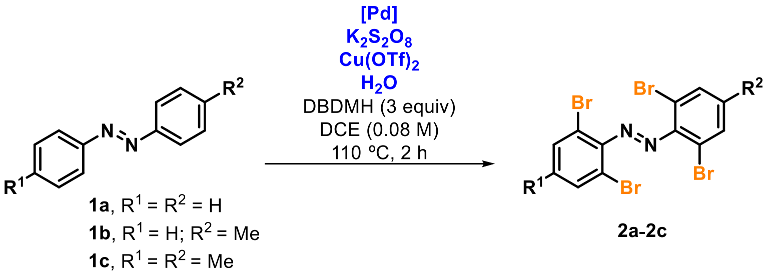

entry	SM	[Pd] (mol%)	$K_2S_2O_8$ (equiv)	[Cu] (mol%)	H_2O (equiv)	Yield (%)[b]
**1**	**1a**	**$Pd(PPh_3)_4$ (10)**	1.2	**20**	**0.7**[c]	**99**
**2**	**1a**	$Pd(PPh_3)_4$ (10)	1.2	40	1.4[c]	92
**3**	**1a**	$Pd(PPh_3)_4$ (5)	1.2	20	0.7[c]	86
**4**	**1a**	$Pd(PPh_3)_4$ (5)	1.2	40	1.4[c]	68
**5**	**1a**	$Pd(OAc)_2$ (10)	1.2	20	0.7[c]	50
**6**	**1a**	$Pd(OAc)_2$ (10)	1.2	40	1.4[c]	75
**7**	**1a**	-	1.2	40	1.4[c]	0
**8**	**1a**	$Pd(OAc)_2$ (10)	1.2	40[d]	N/A[e]	35
**9**	**1a**	$Pd(PPh_3)_4$ (10)	1.2	-	0.7[f]	90
**10**	**1a**	$Pd(PPh_3)_4$ (10)	1.2	-	N/A[e]	79
**11**	**1a**	$Pd(PPh_3)_4$ (10)	-	20	0.7[c]	87
**12**[g]	**1a**	$Pd(PPh_3)_4$ (10)	-	-	0.7[f]	85
**13**[g]	**1a**	$Pd(PPh_3)_4$ (10)	1.2	20	-	0

**14**[g]	**1a**	$Pd(PPh_3)_4$ (10)	1.2	-	-	0
**15**[g]	**1a**	$Pd(PPh_3)_4$ (10)	-	-	-	0
**16**	**1b**	**$Pd(PPh_3)_4$ (10)**	1.2	**20**	**0.7**[c]	**75**
**17**	**1b**	$Pd(PPh_3)_4$ (10)	1.2	40	1.4[c]	60
**18**	**1c**	$Pd(PPh_3)_4$ (10)	1.2	20	0.7[c]	67
**19**	**1c**	**$Pd(PPh_3)_4$ (10)**	1.2	**40**	**1.4**[c]	**77**

[a]Reactions run with **1a-c** (0.20 mmol). [b]Isolated yields. [c]H_2O added as part of the hydrated copper(II) salt, $Cu(OTf)_2 \cdot 3.5H_2O$. [d]Anhydrous $Cu(OTf)_2$ was used instead. [e]DCE from an unsealed bottle was used. [f]H_2O added separately via microsyringe. [g]Reactions run using anhydrous reagents and solvent under a nitrogen atmosphere.

2.1.2. Amount of Water in Hydrated Copper(II) Triflate

The amount of water used in these experiments was determined by the amount present in hydrated $Cu(OTf)_2$, which gave excellent results when compared to the anhydrous reagent. Moreover, it is a very convenient way to add the necessary amount of water to the reaction mixture. Control experiments were run to determine the amount of water that dry copper(II) triflate will absorb if exposed to air or will be present after re-crystallization. In a typical experiment, a sample of commercially available dry copper(II) triflate (109 mg, 0.30 mmol) was left in a vial open to air. After 16 h it had turned from a white powder (Figure S1), to a blue one (127 mg). Longer times under this conditions did not result in a further increase of mass. The obtained blue solid was dried in a vacuum oven at 100 °C for 16 h, yielding again a white solid (108 mg). The mass difference between the hydrated and dried samples of $Cu(OTf)_2$ corresponds to 3.5 equivalents with respect to copper.

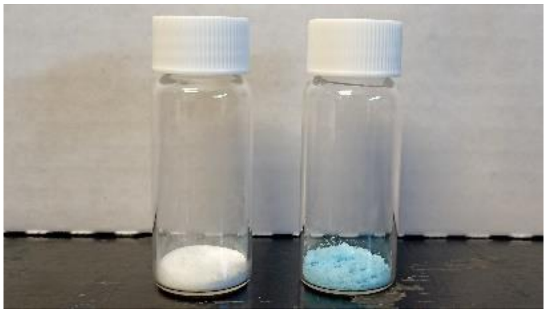

Figure S1. Pictures of anhydrous $Cu(OTf)_2$ (left) and $Cu(OTf)_2 \cdot 3.5H_2O$ (right).

Copper(II) triflate from different sources was used in control reactions to assess the difference in performance (Table S2). These results show again that water is key for a reliable outcome of the transformation and that using $Cu(OTf)_2$ that has been stored under air is an ideal source of both copper and the right amount of water. Alternatively, water can be added to the reaction mixture if using dry $Cu(OTf)_2$ is preferred (entries 1 and 2). Using non-anhydrous solvents proved to be a non-reliable source of the water required for the reaction (entry 3). In our hands, best results were obtained when incorporating water to the reaction mixture as part of the hydrated copper salt, either as obtained after recrystallization without thorough drying[1] (entry 4) or hydrated after exposure to air as described above (entry 5).

[1] C. L. Jenkins, J. K. Kochi, *J. Am. Chem. Soc.*, 1972, **94**, 843.

Table S2. Control experiments for the C−H *ortho*-bromination of azobenzene to assess the effect of the hydration of copper(II) triflate and the amount of water present.[a]

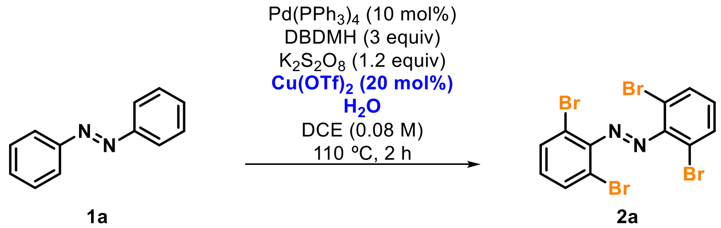

entry	$Cu(OTf)_2$ source	added H_2O (equiv vs. Cu)	Yield[b] (%)
**1**	anhydrous salt	3.5	87
**2**	anhydrous salt	7.0	83
**3**	anhydrous salt	-	45
**4**	recrystallized from non-dried MeCN	-	94
**5**	anhydrous salt exposed to air overnight	-	98

[a]Reactions run with **1a** (0.20 mmol) using reagents and solvents stored under air. [b]Isolated yields.

2.1.3. <u>Control Experiments on the Amount of $Cu(OTf)_2$ Used</u>

In order to gain further insight into the key C−H bromination reaction, different control experiments were performed to understand the role of $Cu(OTf)_2$ in benzylic substrates (Scheme S1).

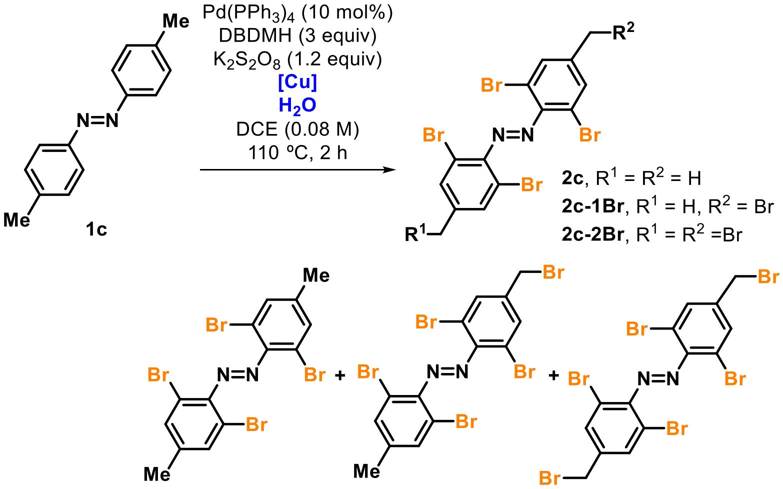

Conditions	2c	2c-1Br	2c-2Br
**No [Cu]**[a]	56%	35%	9%
**$Cu(OTf)_2 \cdot 3.5H_2O$ (20 mol%)**	81%	9%	10%
**$Cu(OTf)_2 \cdot 3.5H_2O$ (40 mol%)**	96%	1%	3%

[a]H_2O (0.7 equiv) added separately

^{1}H-NMR ratios

Scheme S1. Control experiments on the effect of copper on the C−H bromination of azobenzenes bearing benzylic positions.

2.1.4. Radical Scavenging

A control experiment in the presence of TEMPO showed that it suppressed the formation of the product, an observation consistent with the expected formation of radical bromine from DBDMH under the reaction conditions (Scheme S2).

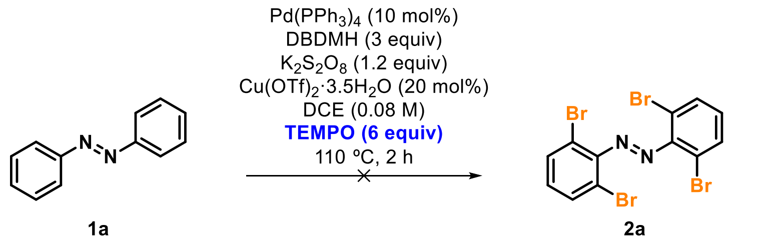

Scheme S2. Control C-H bromination reaction in the presence of theradical scavenger TEMPO.

2.1.5. Proposed Mechanism

The above experiments, along with the role of copper in avoiding benzylic bromination, prompts us to propose a mechanism in which copper(II) intercepts bromine radicals to generate a copper(III) bromide species **III** able to transmetalate with palladium(II) intermediate **I** and oxidise it to generate palladium(IV) complex **II** (Scheme S3).

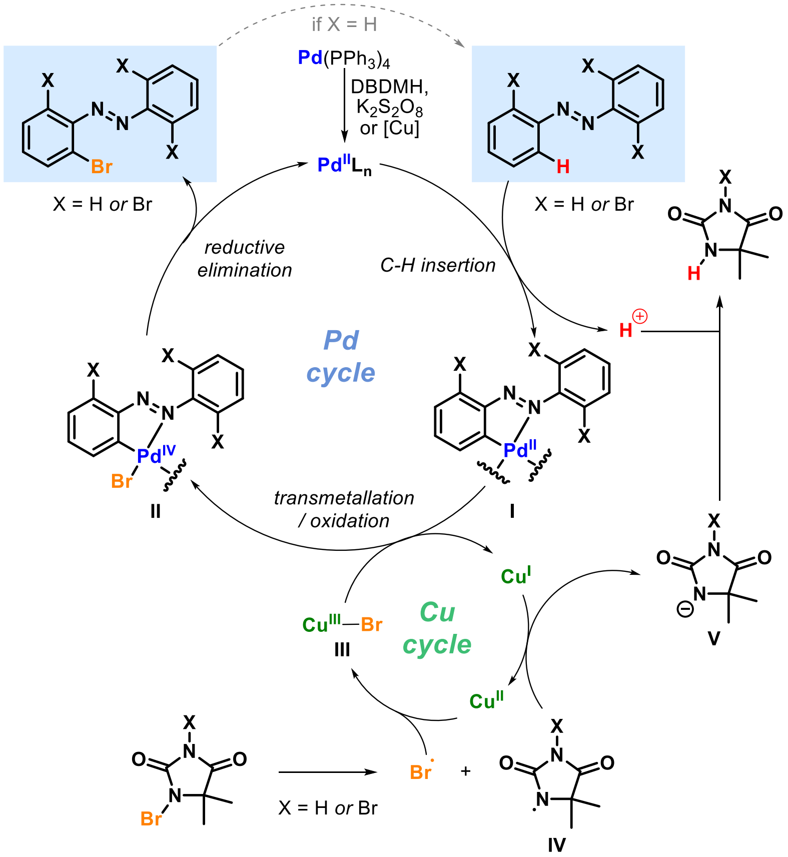

Scheme S3. Proposed mechanism for the palladium- and copper-catalysed C−H *ortho*-bromination of azobenzenes.

Similar copper(III) bromide species have been proposed as key intermediates in bromination reactions using copper(II) salts and other N−Br reagents as the source of bromine atoms.[2] Intermediate **II** can furnish the desired C−Br bond by reductive elimination. During the transmetalation/oxidation process, copper(III) would reduce to copper(I), which can react with *N*-centered radical **IV** formed from DBDMH, thus regenerating copper(II) in the process.

2.2. Copper-catalysed methoxylation

2.2.1. Full Optimization Table

Table S3. Optimization of the copper-catalysed methoxylation of tetra-*ortho*-brominated azobenzene.[a]

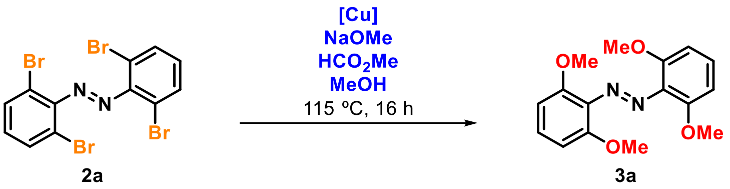

entry	[Cu] (mol%)	NaOMe (equiv)	HCO_2Me (equiv)	MeOH [M]	Yield (%)[b]
**1**	CuCl (20)	2.5	1.6	0.16	16
**2**	CuCl (20)	2.5	1.6	0.08	52
**3**	CuCl (20)	2.5	1.6	0.04	55
**4**	CuCl (40)	2.5	1.6	0.04	42
**5**	**CuCl (20)**	**5.0**	**1.6**	**0.04**	**100**
**6**	CuCl (20)	6.5	1.6	0.04	79
**7**	CuCl (20)	5.0	3.2	0.04	82
**8**	$CuCl_2$ (20)	5.0	1.6	0.04	80
**9**	$Cu(OTf)_2$ (20)	5.0	1.6	0.04	66
**10**	-	5.0	1.6	0.04	18

[a]Reactions run with **2a** (0.16 mmol). [b]Isolated yields.

2.2.2. Control reaction with tetra-*ortho*-chloroazobenzene

A control experiment using optimized conditions with tetra-*ortho*-chloroazobenzene was performed to assess the performance of chlorinated azobenzenes as alternative substrates towards methoxylated azobenzenes. However, the desired tetra-*ortho*-methoxylated azobenzene was not detected (Scheme S4).

[2] Liu, T.; Myers, M. C.; Yu, J.-Q. *Angew. Chem. Int. Ed.* **2017**, *56*, 306-309.

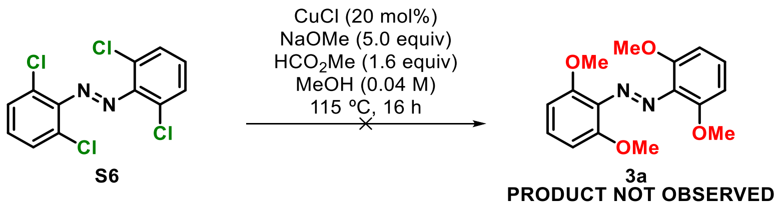

Scheme S4. Control methoxylation reaction of tetra-*ortho*-chloroazobenzene under optimized condition for tetra-*ortho*-brominated substrates.

2.2.3. Radical Scavenging

In order to assess the formation of radical species during the methoxylation reaction, a control experiment with added TEMPO was performed (Scheme S5). Although the expected product was obtained in high yield, it suffered a significant drop from the optimized reaction in the absence of the nitroxyl radical. This might be attributed to the competing reactivity of TEMPO with the copper(I) catalyst,[3] but is not indicative of radical species being involved in the mechanism of the reaction.

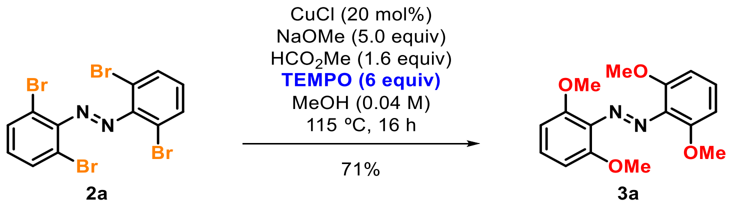

Scheme S5. Control methoxylation reaction in the presence of the radical scavenger TEMPO.

2.3. Synthesis and Characterization

General procedure A: Synthesis of symmetric azobenzenes via oxidative dimerization with manganese dioxide.[4]

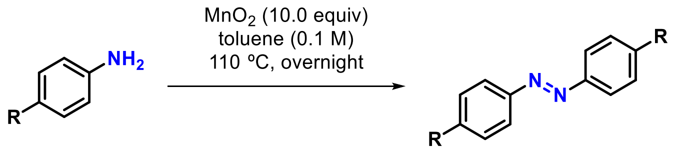

To a three-neck round bottom flask loaded with a solution of the aniline (1.0 equiv) in toluene (0.1 M) was added MnO_2 (10.0 equiv), which had been previously activated in the oven overnight (100 °C). The resulting mixture was stirred vigorously at reflux using an aluminium block and followed by TLC (hexane/EtOAc, 50:50). Once the TLC showed full conversion of the starting material, the mixture was allowed to cool down to room temperature. The reaction mixture was filtered through Celite®, washed with EtOAc and the filtrate was concentrated under reduced pressure. The crude product was purified by silica column chromatography.

[3] J. M. Hoover, B. L. Ryland, S. S. Stahl, *J. Am. Chem. Soc.*, 2013, **135**, 2357.
[4] X. Wang, X. Wang, C. Xia, L. Wu, *Green Chem.*, 2019, **21**, 4189.

General Procedure B: Synthesis of asymmetric azobenzenes via Bayer-Mills reaction.

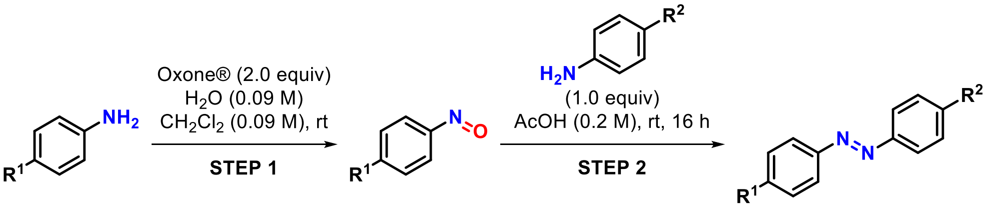

STEP 1: Synthesis of nitroso derivatives[5]

A solution of aniline in CH_2Cl_2 (1.0 equiv, 0.09 M) was added to a round bottom flask containing a solution of Oxone® in H_2O (2.0 equiv, 0.09 M). The mixture was stirred vigorously with an oval stirring bar at room temperature. The progress of the reaction was checked by TLC (CH_2Cl_2/MeOH, 90:10). The solution gradually turned green as the desired nitroso compound formed. Once the TLC showed full conversion, the layers were separated, and the aqueous layer was extracted with CH_2Cl_2 (3 × 60 mL). The combined organic layers were washed with 1 N aqueous HCl (60 mL), saturated aqueous $NaHCO_3$ (60 mL), H_2O (60 mL) and brine (60 mL). Then, the organic phase was dried with anhydrous Na_2SO_4 and concentrated under reduced pressure. The crude thus obtained was checked by ^{1}H-NMR to confirm clean full conversion into the nitroso intermediate and was used without purification.

STEP 2: Bayer-Mills reaction[6]

To a three-neck round bottom flask loaded with a stirring solution of the crude nitroso compound in acetic acid (1.0 equiv, 0.2 M) at room temperature under N_2, the aniline (1.0 equiv) was added portion-wise. After stirring for 16 h, the solvent was removed under reduced pressure and the crude product was purified by silica flash column chromatography.

General procedure C: Synthesis of tetra-*ortho*-brominated azobenzenes via C-H activation.

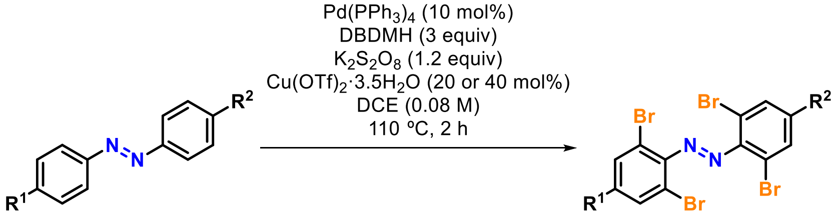

To a thick-wall glass reaction tube charged with azobenzene (0.59 mmol), $Pd(PPh_3)_4$ (68 mg, 0.059 mmol, 0.1 equiv), DBDMH (506 mg, 1.77 mmol, 3.0 equiv), $K_2S_2O_8$ (191 mg, 0.71 mmol, 1.2 equiv) and $Cu(OTf)_2·3.5H_2O$ (0.12 mmol, 20 mol%; or 0.24 mmol, 40 mol%) was added DCE (7.5 mL) before the tube was sealed and the mixture stirred at 110 °C in an oil bath. After 2 h, the mixture was allowed to cool to room temperature. The reaction mixture was filtered over Celite®, washed with EtOAc and the filtrate was concentrated under reduced pressure. The crude product was purified by silica gel column chromatography to obtain the title compound.

[5] B. Priewisch, K. Rück-Braun, *J. Org. Chem.*, 2005, **70**, 2350.
[6] X. Just-Baringo, A. Yeste-Vázquez, J. Moreno-Morales, C. Ballesté-Delpierre, E. Vila, J.; Giralt, *Chem. Eur. J.*, 2021, **27**, 12987.

General procedure D: Synthesis of tetra-*ortho*-methoxylated azobenzenes via a copper-catalysed methoxylation.

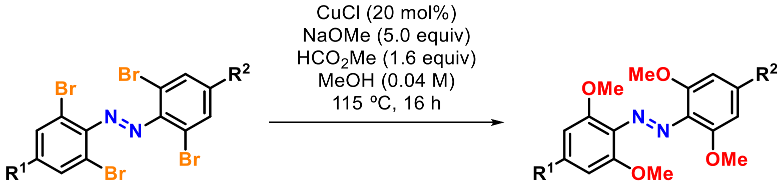

To a thick-wall glass reaction tube charged with brominated azobenzene (0.16 mmol), CuCl (3.2 mg, 0.032 mmol, 20 mol%) and NaOMe (43 mg, 0.8 mmol, 5.0 equiv) and methyl formate (16 µL, 0.26 mmol, 1.6 equiv) was added MeOH (4 mL, 0.04 M) before the tube was sealed and the mixture stirred at 115 °C in an aluminium block. After 16 h the mixture was allowed to cool to room temperature. The reaction mixture was filtered over celite® with EtOAc, and the filtrate was concentrated under reduced pressure. The crude product was purified by silica gel column chromatography to obtain the title compound.

Methyl 3-(4-aminophenyl)propionate (S1)

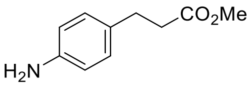

To a stirring solution of 3-(4-aminophenyl)propionic acid (3.00 g, 18.2 mmol) dissolved in MeOH (55 mL), *p*-toluenesulfonic acid monohydrate (3.75 g, 21.8 mmol) was added and the solution was stirred at reflux using an aluminium block for 16 h. The reaction was cooled down to room temperature and the volatiles were evaporated under reduced pressure. The crude product partitioned between a saturated aqueous solution of $NaHCO_3$ and CH_2Cl_2, the two phases were separated, and the aqueous phase was extracted with CH_2Cl_2 (2 x 50 mL). The organic phase was dried with anhydrous Na_2SO_4 and concentrated under reduced pressure to obtain the title compound as a brown solid (3.05 g, 93%).

The product thus obtained matched the one reported in the literature.[7]

Methyl 2-(4-aminophenyl)acetate (S2)

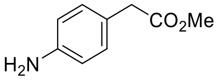

To a stirring solution of 2-(4-aminophenyl)acetic acid (2.50 g, 16.5 mmol) dissolved in MeOH (50 mL), *p*-toluenesulfonic acid monohydrate (3.40 g, 20.0 mmol) was added and the solution was stirred at reflux using an aluminium block for 16 h. The reaction was cooled down to room temperature and the volatiles were evaporated under reduced pressure. The crude product was partitioned between a saturated aqueous solution of $NaHCO_3$ and CH_2Cl_2, the two phases were separated, and the aqueous phase was extracted with CH_2Cl_2 (2 x 50 mL). The organic phase was dried with anhydrous Na_2SO_4 and concentrated under reduced pressure to obtain the title compound as a brown solid (2.40 g, 88%).

The product thus obtained matched the one reported in the literature.[8]

---

[7] S. Sasaki, S. Kitamura, N. Negoro, M. Suzuki, Y. Tsujihata, N. Suzuki, T. Santou, N. Kanzaki, M. Harada, Y. Tanaka, M. Kobayashi, N. Tada, M. Funami, T. Tanaka, Y. Yamamoto, K. Fukatsu, T. Yasuma, Y. Momose, *J. Med. Chem.*, 2011, **54**, 1365.
[8] J. W. Walton, J. M. Cross, T. Riedel, P. J. Dyson, *Org. Biomol. Chem.*, 2017, **15**, 9186.

N-(4-aminophenethyl)-2,2,2-trifluoroacetamide (S3)

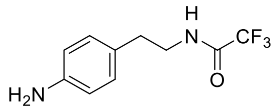

To a stirring solution of 4-(2-aminoethyl)aniline (4.0 mL, 30.0 mmol) in CH_2Cl_2 (150 mL) was added methyl trifluoroacetate (3.0 mL, 30.0 mmol). After 18 h at room temperature, the solvent was removed under reduced pressure and the crude product was purified by silica flash column chromatography (CH_2Cl_2/MeOH, 100:0 to 98:2), affording product the title compound as a yellow solid (6.80 g, 96%).

The product thus obtained matched the one reported in the literature.[3]

N-(4-aminobenzyl)-2,2,2-trifluoroacetamide (S4)

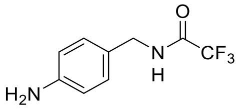

To a stirring solution of 4-(2-aminobenzyl)aniline (1.3 mL, 10.0 mmol) in CH_2Cl_2 (50 mL) we added the methyl trifluoroacetate (1.0 mL, 10.0 mmol). After 18 h at room temperature, the solvent was removed under reduced pressure and the crude product was purified by silica flash column chromatography (CH_2Cl_2/MeOH, 100:0 to 98:2), affording product the title compound as a yellow solid (2.10 g, 96%).

The product thus obtained matched the one reported in the literature.[9]

***tert*-Butyl (4-aminophenethyl)carbamate (S5)**

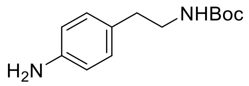

To a round bottom flask loaded with a stirring solution of 4-(2-aminoethyl)aniline (1.1 mL, 8.50 mmol) in THF (60 mL) at 0 °C was added dropwise a solution of Boc_2O (1.90 g, 8.90 mmol) in THF (34 mL). After the addition, the ice bath was removed, and the mixture was stirred at room temperature. After 2 h, H_2O (10 mL) was added, and the clear solution obtained was concentrated under vacuum. More THF was added to the residue to co-evaporate any remaining H_2O. The crude product was purified by silica flash column chromatography (hexane/EtOAc, 100:0 to 80:20), affording the title compound as a yellow solid (1.40 g, 72%).

The product thus obtained matched the one reported in the literature.[10]

[9] R. Kileci-Ksoll, C. Winklhofer, W. Steglich, *Synthesis*, 2010, **13**, 2287.
[10] M. Schönberger, M. Althaus, M. Fronius, W. Clauss, D. Trauner, *Nat. Chem.*, 2014, **6**, 712.

(*E*)-1,2-Bis(2,6-dichlorophenyl)diazene (S6)

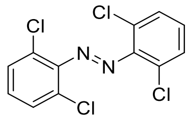

To a thick-wall glass reaction tube charged with azobenzene (1.65 mmol), $Pd(PPh_3)_4$ (190 mg, 0.17 mmol, 0.1 equiv), TCCA (767 mg, 3.30 mmol, 2.0 equiv), $K_2S_2O_8$ (460 mg, 1.98 mmol, 1.2 equiv) was added DCE (22 mL) before the tube was sealed and the mixture stirred at 110 °C in an oil bath. After 2 h, the mixture was allowed to cool to room temperature. The reaction mixture was filtered over Celite®, washed with EtOAc and the filtrate was concentrated under reduced pressure. The crude product was purified by silica gel column chromatography (hexane/DCM, 2:8) to obtain the title compound as a red solid (443 mg, 84%).

The product thus obtained matched the one reported in the literature.[11]

(*E*)-3-(3,5-Dichloro-4-((2,6-dichlorophenyl)diazenyl)phenyl)propanoic acid (S7)

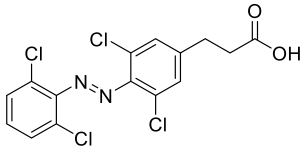

To a thick-wall glass reaction tube charged with (*E*)-3-(4-(phenyldiazenyl)phenyl)propionic acid (**1i**) (500 mg, 2.00 mmol), $Pd(OAc)_2$ (45 mg, 0.20 mmol) and NCS (1.60 g, 12.00 mmol) under N_2 was added acetic acid (80 mL) before the tube was sealed and the mixture stirred at 80 °C in an oil bath. After stirring for 20 hours, the mixture was allowed to cool to room temperature. Volatiles were removed under vacuum and the residue was dissolved in EtOAc (130 mL). The solution was washed with H_2O (8 × 130 mL), dried ($MgSO_4$) and concentrated. The crude product was purified by silica gel column chromatography (hexane/EtOAc 100:0 to 50:50). The title product was obtained as a red solid (646 mg, 82%).

The product thus obtained matched the one reported in the literature.[6]

1-(4-Methylphenyl)-2-phenyldiazene (1b)

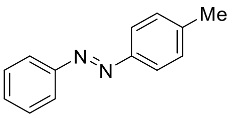

Prepared following general procedure B, using aniline (1.0 mL, 11.0 mmol) in CH_2Cl_2 (125 mL) and Oxone® (6.76 g, 22.0 mmol) in H_2O (125 mL). This afforded nitrosobenzene as a brownish green solid. The resulting crude was checked by ^{1}H-NMR to confirm clean full conversion into the nitroso compound and it was used immediately in the next step without further purification, using nitrosobenzene (770 mg, 7.19 mmol) in acetic acid (35 mL) and *p*-toluidine (770 mg, 7.19 mmol). Purification of the crude product by silica flash column chromatography (hexane) afforded the title compound as an orange solid (977 mg, 27%). During ^{1}H-NMR analysis, only the *trans* isomer was observed.

[11] Q. Liu, X. Luo, S. Wei, Y. Wang, J. Zhu, Y. Liu, F. Quan, M. Zhang, C. Xia, *Tetrahedron Lett.*, 2019, **60**, 1715.

^{1}H-NMR (400 MHz, $CDCl_3$) δ 7.90 (m, $C_{Ar}H$, 2 H), 7.83 (d, J = 8.2 Hz, $C_{Ar}H$, 2 H), 7.56-7.41 (m, $C_{Ar}H$, 3 H), 7.31 (d, J = 8.2 Hz, $C_{Ar}H$, 2 H), 2.43 (s, CH_3, 3 H) ppm.

^{13}C{^{1}H}-NMR (100 MHz, $CDCl_3$) δ 152.9 (C_{Ar}N), 150.9 (C_{Ar}N), 141.7 ($C_{Ar}CH_3$), 130.8 (C_{Ar}H), 129.9 (C_{Ar}H), 129.2 (C_{Ar}H), 123.0 (C_{Ar}H), 122.9 (C_{Ar}H), 21.65 ($C_{Ar}CH_3$) ppm.

HRMS calcd for $C_{13}H_{13}N_2$ $[M+H]^+$: 197.1073, found 197.1078.

(*E*)-1,2-Bis(4-methylphenyl)diazene (1c)

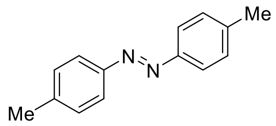

Prepared following general procedure A, using *p*-toluidine (400 mg, 3.73 mmol), MnO_2 (3.2 g, 37.3 mmol) and toluene (20 mL). The title compound was obtained as orange crystals (387 mg, 98%). During ^{1}H-NMR analysis, only the *trans* isomer was observed.

^{1}H-NMR (*trans* isomer) (400 MHz, $CDCl_3$) δ 7.81 (d, J = 8.2 Hz, $C_{Ar}H$, 4 H), 7.32 (d, J = 8.2 Hz, $C_{Ar}H$, 4 H), 2.43 (s, CH_3, 6 H) ppm.

^{13}C{^{1}H}-NMR (*trans* isomer) (100 MHz, $CDCl_3$) δ 150.9 (C_{Ar}N), 141.3 ($C_{Ar}CH_3$), 129.8 (C_{Ar}H), 122.8 (C_{Ar}H), 21.6 ($C_{Ar}CH_3$) ppm.

HRMS calcd for $C_{14}H_{15}N_2$ $[M+H]^+$: 211.1230, found 211.1230.

(*E*)-1,2-Bis(4-chlorophenyl)diazene (1e)

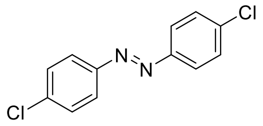

Prepared following general procedure A, using 4-chloroaniline (1.01 g, 7.96 mmol), MnO_2 (6.92 g, 79.6 mmol) and toluene (79 mL). This afforded the title compound as an orange solid (997 mg, 99%). During ^{1}H-NMR analysis, the product was observed as a mixture of *trans*/*cis* isomers (95:5).

^{1}H-NMR (*trans* isomer) (400 MHz, $CDCl_3$) δ 7.86 (d, J = 8.6 Hz, $C_{Ar}H$, 4 H), 7.49 (d, J = 8.6 Hz, $C_{Ar}H$, 4 H) ppm.

^{1}H-NMR (*cis* isomer) (400 MHz, $CDCl_3$) δ 7.26 (d, J = 8.6 Hz, $C_{Ar}H$, 4 H), 6.80 (d, J = 8.6 Hz, $C_{Ar}H$, 4 H) ppm.

^{13}C{^{1}H}-NMR (100 MHz, $CDCl_3$) δ 150.9 (C_{Ar}N), 137.4 (C_{Ar}Cl), 129.6 (C_{Ar}H), 124.3 (C_{Ar}H) ppm.

Low Res EI-MS calcd for $C_{12}H_8Cl_2N_2$ [M]: 250.0065, found 250.0. ESI did not provide a detectable molecular ion for HRMS analysis.

(*E*)-1,2-Bis(4-bromophenyl)diazene (1f)

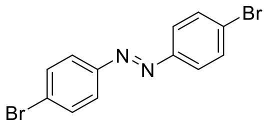

Prepared following general procedure A, using 4-bromoaniline (1.01 g, 5.88 mmol), MnO_2 (5.11 g, 58.8 mmol) and toluene (60 mL). This afforded the title compound as an orange solid (950 mg, 95%). During ^{1}H-NMR analysis, the product was observed as a mixture of *trans*/*cis* isomers (96:4).

^{1}H-NMR (*trans* isomer) (400 MHz, $CDCl_3$) δ 7.79 (d, *J* = 8.8 Hz, $C_{Ar}H$, 4 H), 7.65 (d, *J* = 8.8 Hz, $C_{Ar}H$, 4 H) ppm.

^{1}H-NMR (*cis* isomer) (400 MHz, $CDCl_3$) δ 7.42 (d, *J* = 8.8 Hz, $C_{Ar}H$, 4 H), 6.71 (d, *J* = 8.8 Hz, $C_{Ar}H$, 4 H) ppm.

^{13}C{^{1}H}-NMR (100 MHz, $CDCl_3$) δ 151.3 (C_{Ar}N), 132.5 (C_{Ar}H), 125.9 (C_{Ar}Br), 124.5 (C_{Ar}H). The *cis* isomer was not observed due to the small ratio and intensity of the corresponding signals.

HRMS calcd for $C_{12}H_9Br_2N_2$[M+H]$^+$: 338.9127, found 338.9134.

(*E*)-1,2-Bis(4-iodophenyl)diazene (1g)

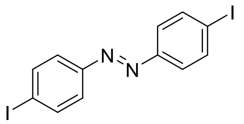

Prepared following general procedure A, using 4-iodoaniline (2.08 g, 9.50 mmol), MnO_2 (8.26 g, 95.0 mmol) and toluene (100 mL) for 2 h. The crude product was purified by filtration through Celite with the aid of warm CH_2Cl_2. The title compound was obtained as orange crystals (2.06 g, quant.). During ^{1}H-NMR analysis, the product was observed as a mixture of *trans*/*cis* isomers (96:4).

^{1}H-NMR (*trans* isomer) (400 MHz, $CDCl_3$) δ 7.87 (d, *J* = 8.4 Hz, $C_{Ar}H$, 4 H), 7.64 (d, *J* = 8.4 Hz, $C_{Ar}H$, 4 H) ppm.

^{1}H-NMR (*cis* isomer) (400 MHz, $CDCl_3$) δ 7.62 (d, *J* = 8.4 Hz, $C_{Ar}H$, 4 H), 6.60 (d, *J* = 8.4 Hz, $C_{Ar}H$, 4 H) ppm.

^{13}C{^{1}H}-NMR (*trans* isomer) (100 MHz, $CDCl_3$) δ 151.9 (C_{Ar}N), 138.6 (C_{Ar}H), 124.7 (C_{Ar}H), 98.3 (C_{Ar}I) ppm. The *cis* isomer was not observed due to the small ratio and intensity of the corresponding signals.

HRMS calcd for $C_{12}H_9I_2N_2$ [M+H]$^+$: 434.885, found 434.884.

(*E*)-1,2-Bis(4-(trifluoromethyl)phenyl)diazene (1h)

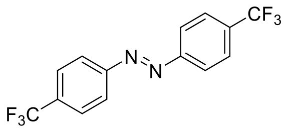

Prepared following general procedure A, using 4-(trifluoromethyl)aniline (1.01 g, 6.28 mmol), MnO_2 (5.46 g, 62.8 mmol) and toluene (63 mL). Purification of the crude product by silica flash column

chromatography (hexane) afforded the title compound as a yellow solid (498 mg, 50%). During ^{1}H-NMR analysis, only the *trans* isomer was observed.

^{1}H-NMR (*trans* isomer) (400 MHz, $CDCl_3$) δ 8.04 (d, *J* = 8.0 Hz, $C_{Ar}H$, 4 H), 7.81 (d, *J* = 8.0 Hz, $C_{Ar}H$, 4 H) ppm.

^{13}C{^{1}H}-NMR (*trans* isomer) (100 MHz, $CDCl_3$) δ 154.2 ($C_{Ar}N$), 133.1 (q, *J* = 32.3 Hz, $C_{Ar}CF_3$), 126.5 (q, *J* = 8.0 Hz, $C_{Ar}H$), 123.9 (q, *J* = 273.7 Hz, CF_3), 123.4 ($C_{Ar}H$) ppm.

^{19}F-NMR (376 MHz, $CDCl_3$) δ −62.7 ppm.

HRMS calcd for $C_{14}H_9F_6N_2$ $[M+H]^+$: 319.0664, found 319.0664.

(*E*)-3-(4-(Phenyldiazenyl)phenyl)propionic acid (1i)

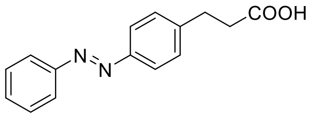

Prepared following general procedure B, using aniline (1.0 mL, 11.0 mmol) in CH_2Cl_2 (125 mL) and Oxone® (6.76 g, 22.0 mmol) in H_2O (125 mL). This afforded nitrosobenzene as a brownish green solid. The resulting crude was checked by ^{1}H-NMR to confirm clean full conversion into the nitroso compound and it was used immediately in the next step without further purification, using nitrosoaniline (578 mg, 5.40 mmol) in acetic acid (27 mL) and 3-(4-aminophenyl)propionic acid (892 mg, 5.40 mmol). The solvent was removed under reduced pressure and the crude product was purified by silica flash column chromatography (hexane/EtOAc, 100:0 to 50:50), affording the title compound as an orange solid (1.15 g, 41%). During ^{1}H-NMR analysis, the product was observed as a mixture of *trans*/*cis* isomers (89:11).

The product thus obtained matched the one reported in the literature.[3]

^{1}H-NMR (*trans* isomer) (400 MHz, $CDCl_3$) δ 7.89-7.92 (m, $C_{Ar}H$, 2 H), 7.86 (d, *J* = 8.4 Hz, $C_{Ar}H$, 2 *H*), 7.46-7.54 (m, $C_{Ar}H$, 3 H), 7.37 (d, *J* = 8.4 Hz, $C_{Ar}H$, 2 H), 3.05 (t, *J* = 7.6 Hz, $C_{Ar}CH_2CH_2CO_2H$, 2H), 2.75 (t, *J* = 7.6 Hz, $C_{Ar}CH_2CH_2CO_2H$, 2 H) ppm.

^{1}H-NMR (*cis* isomer) (400 MHz, $CDCl_3$) δ 7.29-7.22 (m, $C_{Ar}H$, 2 H), 7.15 (t, *J* = 7.6 Hz, $C_{Ar}H$, 1 *H*), 7.09 (d, *J* = 8.0 Hz, $C_{Ar}H$, 2 H), 6.84 (d, *J* = 8.4 Hz, $C_{Ar}H$, 2 H), 6.80 (d, *J* = 8.4 Hz, $C_{Ar}H$, 2 H), 2.90 (t, *J* = 7.8 Hz, $C_{Ar}CH_2CH_2CO_2H$, 2H), 2.63 (t, *J* = 7.8 Hz, $C_{Ar}CH_2CH_2CO_2H$, 2 H) ppm.

^{13}C{^{1}H}-NMR (*trans* isomer) (100 MHz, $CDCl_3$) δ 176.7 (CO_2H), 152.8 ($C_{Ar}N$), 151.5 ($C_{Ar}N$), 143.6 ($C_{Ar}CH_2$), 129.2 ($C_{Ar}H$), 129.2 ($C_{Ar}H$), 123.3 ($C_{Ar}H$), 122.9 ($C_{Ar}H$), 35.1 ($C_{Ar}CH_2CH_2CO_2H$), 30.6 ($C_{Ar}CH_2CH_2CO_2H$) ppm.

Dimethyl 2,2'-(diazene-1,2-diylbis(4,1-phenylene))(*E*)-dipropionate (1k)

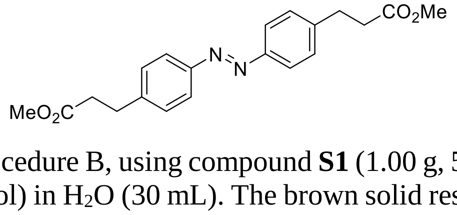

Prepared following general procedure B, using compound **S1** (1.00 g, 5.58 mmol) in CH_2Cl_2 (30 mL), and Oxone® (3.43 g, 11.2 mmol) in H_2O (30 mL). The brown solid residue was checked by ^{1}H-NMR to confirm clean full conversion into the nitroso compound and it was used immediately in the next step without further purification, using the nitroso derivative dissolved in acetic acid (20 mL), and compound **S1** (1.00 g, 5.58 mmol). The solvent was removed under reduced pressure and the crude

product was purified by silica gel flash chromatography (hexane/EtOAc, 8:2) to obtain an orange solid. The orange solid was recrystallized from hexane (15 mL) to obtain the title compound as orange crystals (831 mg, 42%). During ^{1}H-NMR analysis, only the *trans* isomer was observed.

^{1}H-NMR (*trans* isomer) (400 MHz, $CDCl_3$) δ 7.82 (d, *J* = 8.4 Hz, $C_{Ar}H$, 4 H), 7.32 (d, *J* = 8.4 Hz, $C_{Ar}H$, 4 H), 3.68 (s, CO_2CH_3, 6 H), 3.03 (t, *J* = 7.8 Hz, $C_{Ar}CH_2CH_2CO_2CH_3$, 4 H), 2.68 (t, *J* = 7.8 Hz, $C_{Ar}CH_2CH_2CO_2CH_3$, 4 H) ppm.

^{13}C{^{1}H}-NMR (*trans* isomer) (100 MHz, $CDCl_3$) δ 173.1 (CO_2CH_3), 151.4 ($C_{Ar}N$), 143.8 ($C_{Ar}CH_2$), 129.1 ($C_{Ar}H$), 123.0 ($C_{Ar}H$), 51.7 (CO_2CH_3), 35.4 ($C_{Ar}CH_2CH_2CO_2CH_3$), 30.8 ($C_{Ar}CH_2CH_2CO_2CH_3$) ppm.

HRMS calcd for $C_{20}H_{23}N_2O_4$ $[M+H]^+$: 355.1652, found 355.1653.

Methyl (*E*)-3-(4-(phenyldiazenyl)phenyl)propanoate (1l)

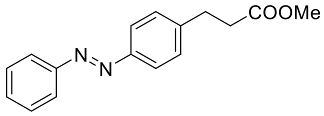

Prepared following general procedure B, using aniline (1.0 mL, 11.0 mmol) in CH_2Cl_2 (125 mL), and Oxone® (6.76 g, 22.0 mmol) in H_2O (125 mL). This afforded nitrosobenzene as a brownish green solid. The resulting crude was checked by ^{1}H-NMR to confirm clean full conversion into the nitroso compound and it was used immediately in the next step without further purification, using nitrosoaniline (390 mg, 3.60 mmol) in acetic acid (18 mL) and compound **S1** (601 mg, 3.60 mmol). The solvent was removed under reduced pressure and the crude product was purified by silica gel flash chromatography (hexane/EtOAc, 100:0 to 70:30) to afford the title compound as an orange solid (702 mg, 24%). During ^{1}H-NMR analysis, only the *trans* isomer was observed.

^{1}H-NMR (*trans* isomer) (400 MHz, $CDCl_3$) δ 7.91 (m, $C_{Ar}H$, 2 H), 7.86 (m, $C_{Ar}H$, 2 H), 7.54-7.44 (m, $C_{Ar}H$, 3 H), 7.35 (d, *J* = 8.8 Hz, $C_{Ar}H$, 2 H), 3.69 (s, CO_2CH_3, 3 H), 3.04 (t, *J* = 7.8 Hz, $C_{Ar}CH_2CH_2CO_2CH_3$, 2 H), 2.69 (t, *J* = 7.8 Hz, $C_{Ar}CH_2CH_2CO_2CH_3$, 2 H) ppm.

^{13}C{^{1}H}-NMR (*trans* isomer) (100 MHz, $CDCl_3$) δ 173.2 (CO_2Me), 152.8 ($C_{Ar}N$), 151.5 ($C_{Ar}N$), 144.0 (C_{Ar}), 131.0 ($C_{Ar}H$), 129.2 ($C_{Ar}H$), 129.2 ($C_{Ar}H$), 123.2 ($C_{Ar}H$), 122.9 ($C_{Ar}H$), 51.8 (CO_2CH_3), 35.5 ($C_{Ar}CH_2CH_2CO_2CH_3$), 30.9 ($C_{Ar}CH_2CH_2CO_2CH_3$) ppm.

HRMS calcd for $C_{16}H_{17}N_2O_2$ $[M+H]^+$: 269.1282, found 269.1285.

Methyl (*E*)-4-(phenyldiazenyl)benzoate (1m)

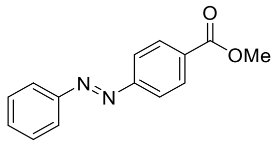

A solution of (*E*)-4-(phenyldiazenyl)benzoic acid (250 mg, 1.10 mmol) and *p*-toluenesulfonic acid monohydrate (230 mg, 1.30 mmol) in methanol (3.3 mL) was stirred at reflux in an oil bath for 18 h. Methanol was evaporated under reduced pressure and the crude product was suspended in a saturated solution of $NaHCO_3$ (25 mL) and extracted with CH_2Cl_2 (3 x 15 mL). The combined organic phases were dried with anhydrous Na_2SO_4 and concentrated under reduced pressure. The title compound was obtained as an orange solid (254 mg, 96%). During ^{1}H-NMR analysis, only the *trans* isomer was observed.

^{1}H-NMR (*trans* isomer) (400 MHz, $CDCl_3$) δ 8.20 (d, *J* = 8.8 Hz, $C_{Ar}H$, 2 H), 7.97-7.94 (m, $C_{Ar}H$, 4 H), 7.57-7.48 (m, $C_{Ar}H$, 3 H), 3.96 (s, CO_2CH_3, 3 H) ppm.

^{13}C{^{1}H}-NMR (*trans* isomer) (100 MHz, $CDCl_3$) δ 166.7 (CO_2Me), 155.3 (C_{Ar}N), 152.7 (C_{Ar}N), 132.0 (C_{Ar}), 131.9 (C_{Ar}H), 130.8 (C_{Ar}H), 129.3 (C_{Ar}H), 123.3 (C_{Ar}H), 122.8 (C_{Ar}H), 52.5 (CO_2CH_3) ppm.

HRMS calcd for $C_{14}H_{13}N_2O_2$ [M+H]$^+$: 241.0972, found 241.0967.

(*E*)-2,2,2-Trifluoro-*N*-(4-(phenyldiazenyl)phenethyl)acetamide (1n)

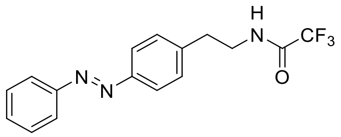

Prepared following general procedure B, using aniline (1.0 mL, 11.0 mmol) in CH_2Cl_2 (125 mL) and Oxone® (6.76 g, 22.0 mmol) in H_2O (125 mL). This afforded nitrosobenzene as a brownish green solid. The resulting crude was checked by ^{1}H-NMR to confirm clean full conversion into the nitroso compound and it was used immediately in the next step without further purification, using nitrosobenzene (351 mg, 3.27 mmol) in acetic acid (16 mL) and *N*-(4-aminophenethyl)-2,2,2-trifluoroacetamide (**S3**) (760 mg, 3.27 mmol). Purification of the crude product by silica flash column chromatography (hexane/EtOAc, 100:0 to 90:10) afforded the title compound as an orange solid (560 mg, 15%). During ^{1}H-NMR analysis, the product was observed as a mixture of *trans*/*cis* isomers (87:13).

^{1}H-NMR (*trans* isomer) (400 MHz, $CDCl_3$) δ 7.94-7.88 (m, $C_{Ar}H$, 4 H), 7.56-7.48 (m, $C_{Ar}H$, 3 H), 7.34 (d, *J* = 8.4 Hz, $C_{Ar}H$, 2 H), 6.44 (bs, $C_{Ar}CH_2CH_2NH$, 1 H), 3.66 (q, *J* = 6.8 Hz, $C_{Ar}CH_2CH_2NH$, 2 H), 2.97 (t, *J* = 6.8 Hz, $C_{Ar}CH_2CH_2NH$, 2 H) ppm.

^{1}H-NMR (*cis* isomer) (400 MHz, $CDCl_3$) δ 7.29-7.22 (m, $C_{Ar}H$, 2 H), 7.16 (t, *J* = 7.4 Hz, $C_{Ar}H$, 1 H), 7.06 (d, *J* = 8.4 Hz, $C_{Ar}H$, 2 H), 6.86-6.78 (m, $C_{Ar}H$, 4 H), 6.32 (bs, $C_{Ar}CH_2CH_2NH$, 1 H), 3.56 (q, *J* = 6.8 Hz, $C_{Ar}CH_2CH_2NH$, 2 H), 2.82 (t, *J* = 6.8 Hz, $C_{Ar}CH_2CH_2NH$, 2 H) ppm.

^{13}C{^{1}H}-NMR (*trans* isomer) (100 MHz, $CDCl_3$) δ 157.4 (q, *J* = 36.9 Hz, $NHCOCF_3$), 152.7 (C_{Ar}), 151.8 (C_{Ar}), 140.8 (C_{Ar}), 131.2 (C_{Ar}H), 129.5 (C_{Ar}H), 129.2 (C_{Ar}H), 123.4 (C_{Ar}H), 122.9 (C_{Ar}H), 115.8 (q, *J* = 286 Hz, $NHCOCF_3$), 41.0 ($C_{Ar}CH_2CH_2NH$), 34.9 ($C_{Ar}CH_2CH_2NH$), ppm.

^{13}C{^{1}H}-NMR (*cis* isomer) (100 MHz, $CDCl_3$) δ 153.5 (C_{Ar}), 152.2 (C_{Ar}), 137.1 (C_{Ar}), 129.1 (C_{Ar}H), 128.9 (C_{Ar}H), 127.6 (C_{Ar}H), 121.3 (C_{Ar}H), 120.5 (C_{Ar}H), 40.8 ($C_{Ar}CH_2CH_2NH$), 34.6 ($C_{Ar}CH_2CH_2NH$), ppm. Neither C from the trifluoroacetamide group was observed for the *cis* isomer due to the small ratio and intensity of those signals.

^{19}F-NMR (376 MHz, $CDCl_3$) δ –75.9 ppm.

HRMS calcd for $C_{16}H_{15}F_3N_3O$ [M+H]$^+$: 322.1162, found 322.1158.

(*E*)-2,2,2-Trifluoro-*N*-(4-(phenyldiazenyl)benzyl)acetamide (1o)

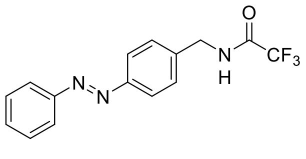

Prepared following general procedure B, using aniline (1.0 mL, 11.0 mmol) in CH_2Cl_2 (125 mL) and Oxone® (6.76 g, 22.0 mmol) in H_2O (125 mL). This afforded nitrosobenzene as a brownish green solid. The resulting crude was checked by ^{1}H-NMR to confirm clean full conversion into the nitroso compound and it was used immediately in the next step without further purification, using nitrosobenzene (197 mg, 1.84 mmol) in acetic acid (10 mL), and compound **S4** (402 mg, 1.84 mmol). Purification of the crude product by silica flash column chromatography (hexane/EtOAc, 100:0 to 90:10) afforded the title compound as an orange solid (471 mg, 14%). During ^{1}H-NMR analysis, the product was observed as a mixture of *trans*/*cis* isomers (97:3).

^{1}H-NMR (*trans* isomer) (400 MHz, $CDCl_3$) δ 7.95-7.90 (m, $C_{Ar}H$, 4 H), 7.56-7.47 (m, $C_{Ar}H$, 3 H), 7.44 (d, J = 8.0 Hz, $C_{Ar}H$, 2 H), 6.63 (bs, $C_{Ar}CH_2NH$, 1 H), 4.62 (d, J = 6.0 Hz, $C_{Ar}CH_2NH$, 2 H) ppm.

^{13}C{^{1}H}-NMR (*trans* isomer) (100 MHz, $CDCl_3$) δ 157.4 (q, J = 37.0 Hz, NH*C*OCF_3), 152.7 (C_{Ar}N), 152.6 (C_{Ar}N), 138.6 (C_{Ar}), 131.4 (C_{Ar}H), 129.3 (C_{Ar}H), 128.8 (C_{Ar}H), 123.6 (C_{Ar}H), 123.1 (C_{Ar}H), 116.0 (q, J = 287 Hz, NHCOCF_3), 43.7 ($C_{Ar}CH_2$NH) ppm.

^{19}F-NMR (376 MHz, $CDCl_3$) δ –75.7 ppm.

HRMS calcd for $C_{15}H_{13}F_3N_3O$ $[M+H]^+$: 308.1005, found 308.1006.

Methyl (*E*)-3-(4-((4-(2-(2,2,2-trifluoroacetamido)ethyl)phenyl)diazenyl)phenyl)propanoate (1p)

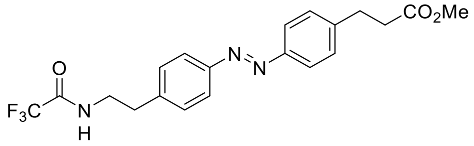

Prepared following general procedure B, using compound **S1** (1.00 g, 5.58 mmol) in CH_2Cl_2 (30 mL), and Oxone® (3.40 g, 11.2 mmol) in H_2O (30 mL). This afforded methyl 3-(4-nitrosophenyl)propanoate as a brownish green solid. The resulting crude was checked by ^{1}H-NMR to confirm clean full conversion into the nitroso intermediate and it was used immediately in the next step without further purification, using methyl 3-(4-nitrosophenyl)propanoate (844 mg, 4.37 mmol) in acetic acid (22 mL), and compound **S3** (1.00 g, 4.37 mmol). Purification of the crude product by silica flash column chromatography (hexane/EtOAc, 100:0 to 85:15) afforded the title compound as an orange solid (967 mg, 43%). During ^{1}H-NMR analysis, the product was observed as a mixture of *trans*/*cis* isomers (88:12).

^{1}H-NMR (*trans* isomer) (400 MHz, $CDCl_3$) δ 7.89-7.82 (m, $C_{Ar}H$, 4 H), 7.37-7.30 (m, $C_{Ar}H$, 4 H), 6.48 (s, $CH_2NHCOCF_3$, 1 H), 3.68 (s, CO_2CH_3, 3 H), 3.66 (m, $C_{Ar}CH_2CH_2NH$, 2 H), 3.03 (t, J = 7.8 Hz, $C_{Ar}CH_2CH_2CO_2CH_3$, 2 H), 2.97 (t, J = 7.2 Hz, $C_{Ar}CH_2CH_2NH$, 2 H), 2.68 (t, J = 7.8 Hz, $C_{Ar}CH_2CH_2CO_2CH_3$, 2 H) ppm.

^{1}H-NMR (*cis* isomer) (400 MHz, $CDCl_3$) δ 7.07 (d, J = 8.4 Hz, $C_{Ar}H$, 4 H), 6.80 (d, J = 8.4 Hz, $C_{Ar}H$, 2 H), 6.77 (d, J = 8.4 Hz, $C_{Ar}H$, 2 H), 6.48 (s, $CH_2NHCOCF_3$, 1 H), 3.67-3.62 (m, CO_2CH_3, 3 H), 3.57 (q, J = 7.0 Hz, $C_{Ar}CH_2CH_2NH$, 2 H), 2.88 (t, J = 7.6 Hz, $C_{Ar}CH_2CH_2CO_2CH_3$, 2 H), 2.83 (t, J = 7.0 Hz, $C_{Ar}CH_2CH_2NH$, 2 H), 2.58 (t, J = 7.6 Hz, $C_{Ar}CH_2CH_2CO_2CH_3$, 2 H) ppm.

^{13}C{^{1}H}-NMR (*trans* isomer) (100 MHz, $CDCl_3$) δ 173.3 (CO_2CH_3), 157.4 (q, J = 37.0 Hz, $NHCOCF_3$), 151.8 (C_{Ar}), 151.3 (C_{Ar}), 144.1 (C_{Ar}), 140.8 (C_{Ar}), 129.6 ($C_{Ar}H$), 129.2 ($C_{Ar}H$), 123.4 (C_{Ar}), 123.2 (C_{Ar}), 115.9 (q, J = 286 Hz, $NHCOCF_3$), 51.9 (CO_2CH_3), 41.0 ($C_{Ar}CH_2CH_2NH$), 35.5 ($C_{Ar}CH_2CH_2CO_2CH_3$), 35.0 ($C_{Ar}CH_2CH_2NH$), 30.9 ($C_{Ar}CH_2CH_2CO_2CH_3$) ppm.

^{13}C{^{1}H}-NMR (*cis* isomer) (100 MHz, $CDCl_3$) δ 173.2 (CO_2CH_3), 152.3 (C_{Ar}), 151.6 (C_{Ar}), 140.3 (C_{Ar}), 137.0 (C_{Ar}), 129.2 ($C_{Ar}H$), 128.7 ($C_{Ar}H$), 121.1 (C_{Ar}), 121.0 (C_{Ar}), 51.8 (CO_2CH_3), 40.8 ($C_{Ar}CH_2CH_2NH$), 35.4 ($C_{Ar}CH_2CH_2CO_2CH_3$), 34.6 ($C_{Ar}CH_2CH_2NH$), 30.5 ($C_{Ar}CH_2CH_2CO_2CH_3$) ppm. Neither C from the trifluoroacetamide group was observed for the *cis* isomer due to the small ratio and intensity of those signals.

^{19}F-NMR (376 MHz, $CDCl_3$) δ –75.9 ppm.

HRMS calcd for $C_{20}H_{21}F_3N_3O_3$ $[M+H]^+$: 408.1530, found 408.1529.

(*E*)-1,2-Bis(2,6-dibromophenyl)diazene (2a)

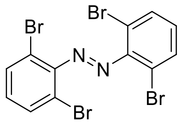

Prepared following general procedure C, using commercially available azobenzene (36 mg, 0.20 mmol) and $Cu(OTf)_2$·$3.5H_2O$ (20 mol%). Purification of the crude product by silica flash column chromatography (hexane) afforded the title compound as a red solid (94 mg, 99%). During ^{1}H-NMR analysis, only the *trans* isomer was observed.

^{1}H-NMR (*trans* isomer) (400 MHz, $CDCl_3$) δ 7.69 (d, J = 8.0 Hz, $C_{Ar}H$, 4 H), 7.11 (t, J = 8.0 Hz, $C_{Ar}H$, 2 H) ppm.

^{13}C{^{1}H}-NMR (*trans* isomer) (100 MHz, $CDCl_3$) δ 149.0 ($C_{Ar}N$), 133.5 ($C_{Ar}H$), 130.5 ($C_{Ar}H$), 116.1 ($C_{Ar}Br$) ppm.

HRMS calcd for $C_{12}H_7Br_4N_2$ $[M+H]^+$: 494.7337, found 494.7341.

(*E*)-1-(2,6-Dibromo-4-methylphenyl)-2-(2,6-dibromophenyl)diazene (2b)

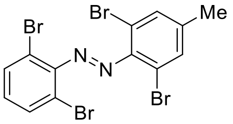

Prepared following general procedure C, using **1b** (116 mg, 0.59 mmol) and $Cu(OTf)_2$·$3.5H_2O$ (20 mol%). Purification of the crude product by silica flash column chromatography (hexane) afforded the title compound as a red solid (227 mg, 75%). During ^{1}H-NMR analysis, the product was observed as a mixture of *trans*/*cis* isomers (95:5).

^{1}H-NMR (*trans* isomer) (400 MHz, $CDCl_3$) δ 7.67 (d, J = 8.0 Hz, $C_{Ar}H$, 2 H), 7.52 (s, $C_{Ar}H$, 2 H), 7.08 (t, J = 8.0 Hz, $C_{Ar}H$, 1 H), 2.38 (s, $C_{Ar}CH_3$, 3 H).

^{1}H-NMR (*cis* isomer) (400 MHz, $CDCl_3$) δ 7.49 (d, J = 8.0 Hz, $C_{Ar}H$, 2 H), 7.31 (s, $C_{Ar}H$, 2 H), 6.99 (t, J = 8.0 Hz, $C_{Ar}H$, 1 H), 2.31 (s, $C_{Ar}CH_3$, 3 H).

^{13}C{^{1}H}-NMR (*trans* isomer) (100 MHz, $CDCl_3$) δ 149.4 ($C_{Ar}N$), 146.3 ($C_{Ar}N$), 141.8 ($C_{Ar}C$), 134.2 ($C_{Ar}H$), 133.7 ($C_{Ar}H$), 130.2 ($C_{Ar}H$), 116.4 ($C_{Ar}Br$), 115.8 ($C_{Ar}Br$), 20.7 (CH_3) ppm.

HRMS calcd for $C_{13}H_9Br_4N_2$ $[M+H]^+$: 508.7494, found 508.7474.

(*E*)-1,2-Bis(2,6-dibromo-4-methylphenyl)diazene (2c)

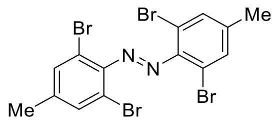

Prepared following general procedure C, using **1c** (124 mg, 0.59 mmol) and $Cu(OTf)_2 \cdot 3.5H_2O$ (40 mol%). Purification of the crude product by silica flash column chromatography (hexane) afforded the title compound as a red solid (240 mg, 77%). During ^{1}H-NMR analysis, only the *trans* isomer was observed.

^{1}H-NMR (*trans* isomer) (400 MHz, $CDCl_3$) δ 7.50 (s, $C_{Ar}H$, 4 H), 2.38 (s, $C_{Ar}CH_3$, 6 H).

^{13}C{^{1}H}-NMR (*trans* isomer) (100 MHz, $CDCl_3$) δ 146.7 (C_{Ar}N), 141.3 (C_{Ar}C), 134.0 (C_{Ar}H), 116.1 (C_{Ar}Br), 20.7 (CH_3).

HRMS calcd for $C_{14}H_{11}Br_4N_2$ $[M+H]^+$: 522.7650, found 522.7637.

(*E*)-1-(2,6-Dibromo-4-methoxyphenyl)-2-(2,6-dibromophenyl)diazene (2d)

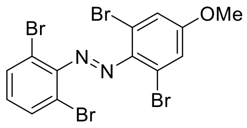

Prepared following general procedure C, using commercially available 4-methoxyazobenzene (125 mg, 0.59 mmol) in the absence of $Cu(OTf)_2 \cdot 3.5H_2O$. Purification of the crude product by silica flash column chromatography (hexane) afforded the title compound as a red solid (293 mg, 94%). During ^{1}H-NMR analysis, the product was observed as a mixture of *trans*/*cis* isomers (66:34).

^{1}H-NMR (*trans* isomer) (400 MHz, $CDCl_3$) δ 7.66 (d, *J* = 8.0 Hz, $C_{Ar}H$, 2 H), 7.27 (s, $C_{Ar}H$, 2 H), 7.07 (t, *J* = 8.0 Hz, $C_{Ar}H$, 1 H), 3.88 (s, OCH_3, 3 H) ppm.

^{1}H-NMR (*cis* isomer) (400 MHz, $CDCl_3$) δ 7.50 (d, *J* = 8.0 Hz, $C_{Ar}H$, 2 H), 7.05 (s, $C_{Ar}H$, 2 H), 6.99 (t, *J* = 8.0 Hz, $C_{Ar}H$, 1 H), 3.80 (s, OCH_3, 3 H) ppm.

^{13}C{^{1}H}-NMR (*trans* isomer) (100 MHz, $CDCl_3$) δ 160.4 (C_{Ar}O), 149.8 (C_{Ar}N), 141.7 (C_{Ar}N), 133.2 (C_{Ar}H), 129.8 (C_{Ar}H), 119.5 (C_{Ar}H), 118.7 (C_{Ar}Br), 115.5 (C_{Ar}Br), 56.3 (OCH_3) ppm. During ^{13}C-NMR analysis only the *trans* isomer was observed, provably due to thermal isomerization of the sample before the experiment.

HRMS calcd for $C_{13}H_9Br_4N_2O$ $[M+H]^+$: 524.7443, found 524.7444.

(*E*)-1,2-Bis(2,6-dibromo-4-chlorophenyl)diazene (2e)

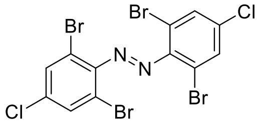

Prepared following general procedure C, using **1e** (148 mg, 0.59 mmol) and $Cu(OTf)_2 \cdot 3.5H_2O$ (40 mol%). Purification of the crude product by silica flash column chromatography (hexane) afforded the title compound as a pink solid (301 mg, 90%). During ^{1}H-NMR analysis, only the *trans* isomer was observed.

^{1}H-NMR (*trans* isomer) (400 MHz, $CDCl_3$) δ 7.72 (s, $C_{Ar}H$, 4 H) ppm.

^{13}C{^{1}H}-NMR (*trans* isomer) (101 MHz, $CDCl_3$) δ 147.4 (C_{Ar}N), 135.7 (C_{Ar}Cl), 133.2 (C_{Ar}H), 116.7 (C_{Ar}Br) ppm.

HRMS calcd for $C_{12}H_5Br_4Cl_2N_2$ $[M+H]^+$: 562.6558, found 562.6566.

(*E*)-1,2-Bis(2,4,6-tribromophenyl)diazene (2f)

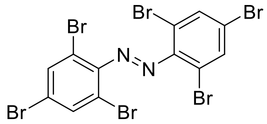

Prepared following general procedure C, using **1f** (200 mg, 0.59 mmol) and $Cu(OTf)_2{\cdot}3.5H_2O$ (40 mol%) overnight. Purification of the crude product by silica flash column chromatography (hexane) afforded the title compound as a purple solid (270 mg, 70%). During ^{1}H-NMR analysis, only the *trans* isomer was observed.

^{1}H-NMR (*trans* isomer) (400 MHz, $CDCl_3$) δ 7.86 (s, $C_{Ar}H$, 4 H) ppm.

^{13}C{^{1}H}-NMR (*trans* isomer) (100 MHz, $CDCl_3$) δ 147.8 (C_{Ar}N), 135.9 (C_{Ar}H), 123.3 (*p*-C_{Ar}Br), 116.9 (*o*-C_{Ar}Br) ppm.

HRMS shows a very low intensity signal, which shows a higher than expected error for the least intense isotopic peaks: calcd for $C_{12}H_5Br_6N_2$$[M+H]^+$: 650.5547, found 650.5855. However, the central and more intense isotopic peaks are within the acceptable error (<10 ppm):

m/z	Calc. m/z	Diff. (ppm)
650.5855	650.5547	47.3
652.5511	652.5527	2.45
654.5509	654.5507	0.31
656.5479	656.5487	1.22
658.5445	658.5467	3.34
660.5513	660.5447	9.99

(*E*)-1,2-Bis(2,6-dibromo-4-iodophenyl)diazene (2g)

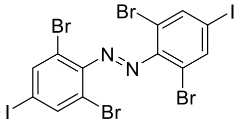

Prepared following general procedure C, using **1g** (256 mg, 0.59 mmol) and $Cu(OTf)_2{\cdot}3.5H_2O$ (40 mol%) overnight. Purification of the crude product by silica flash column chromatography (hexane/EtOAc, 100:0 to 95:5) afforded the title compound as a purple solid (399 mg, 90%). During ^{1}H-NMR analysis, only the *trans* isomer was observed.

^{1}H-NMR (*trans* isomer) (400 MHz, $CDCl_3$) δ 8.04 (s, $C_{Ar}H$, 4 H) ppm.

^{13}C{^{1}H}-NMR (*trans* isomer) (100 MHz, $CDCl_3$) δ 148.49 (C_{Ar}N), 141.56 (C_{Ar}H), 116.90 (C_{Ar}Br), 94.49 (C_{Ar}I).

HRMS calcd for $C_{12}H_5Br_4I_2N_2$ $[M+H]^+$: 746.5270, found 746.5251.

(*E*)-3-(3,5-Dibromo-4-((2,6-dibromophenyl)diazenyl)phenyl)propanoic acid (2i)

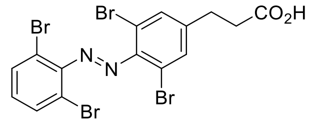

Prepared following general procedure C, using **1i** (150 mg, 0.59 mmol) and $Cu(OTf)_2{\cdot}3.5H_2O$ (20 mol%). Purification of the crude product by silica flash column chromatography (hexane/EtOAc 90:10 + 1% AcOH to 60:40 + 1% AcOH) afforded the title compound as a red solid (315 mg, 95%). During ^{1}H-NMR analysis, only the *trans* isomer was observed.

^{1}H-NMR (*trans* isomer) (400 MHz, DMSO-d_6) δ 12.25 (bs, CO_2H, 1 H), 7.88 (d, *J* = 8.0 Hz, $C_{Ar}H$, 2 H), 7.79 (s, $C_{Ar}H$, 2 H), 7.34 (t, *J* = 8.0 Hz, $C_{Ar}H$, 1 H), 2.90 (t, *J* = 7.4 Hz, $C_{Ar}CH_2CH_2CO_2H$, 2 H), 2.65 (t, *J* = 7.4 Hz, $C_{Ar}CH_2CH_2CO_2H$, 2 H).

^{13}C{^{1}H}-NMR (*trans* isomer) (100 MHz, DMSO-d_6) δ 173.4 (CO_2H), 148.1 (C_{Ar}), 146.0 (C_{Ar}), 145.7 (C_{Ar}), 133.6 ($C_{Ar}H$), 131.7 ($C_{Ar}H$), 115.5 ($C_{Ar}Br$), 115.1 ($C_{Ar}Br$), 34.3 ($C_{Ar}CH_2CH_2CO_2H$), 29.2 ($C_{Ar}CH_2CH_2CO_2H$) ppm.

HRMS calcd for $C_{15}H_9Br_4N_2O_2$ $[M+H]^+$: 564.7403, found 564.7414.

(*E*)-3-(3,5-Dibromo-4-((2,6-dibromophenyl)diazenyl)benzoic acid (2j)

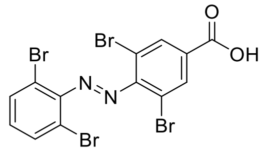

Prepared following general procedure C, using commercially available 4-(phenylazo)benzoic acid (134 mg, 0.59 mmol) and $Cu(OTf)_2{\cdot}3.5H_2O$ (20 mol%). Purification of the crude product by silica flash column chromatography (hexane/EtOAc 90:10 + 1% acetic acid to hexane/EtOAc 60:40 + 1% acetic acid) afforded the title compound as a red solid (263 mg, 82%). During ^{1}H-NMR analysis, the product was observed as a mixture of *trans*/*cis* isomers (89:11).

^{1}H-NMR (*trans* isomer) (400 MHz, DMSO-d_6) δ 13.8 (bs, CO_2H, 1 H), 8.40 (s, $C_{Ar}H$, 2 H), 7.92 (d, *J* = 8.0 Hz, $C_{Ar}H$, 2 H), 7.15 (t, *J* = 8.0 Hz, $C_{Ar}H$, 1 H) ppm.

^{1}H-NMR (*cis* isomer) (400 MHz, DMSO-d_6) δ 13.82 (bs, CO_2H, 1 H), 8.07 (s, $C_{Ar}H$, 2 H), 7.88 (d, *J* = 8.0 Hz, $C_{Ar}H$, 2 H), 7.34 (t, *J* = 8.0 Hz, $C_{Ar}H$, 1 H) ppm.

^{13}C{^{1}H}-NMR (*trans* isomer) (100 MHz, DMSO-d_6) δ 164.5 (CO_2H), 151.0 ($C_{Ar}N$), 147.7 ($C_{Ar}N$), 134.1 ($C_{Ar}H$), 134.1 ($C_{Ar}H$), 133.7 ($C_{Ar}C$), 132.6 ($C_{Ar}H$), 115.9 ($C_{Ar}Br$), 115.1 ($C_{Ar}Br$) ppm. The *cis* isomer was not observed due to the small ratio and intensity of the corresponding signals.

HRMS calcd for $C_{13}H_5Br_4N_2O_2$ $[M-H]^-$: 536.7090, found 536.7102.

Dimethyl 3,3'-(diazene-1,2-diylbis(3,5-dibromo-4,1-phenylene))(*E*)-dipropionate (2k)

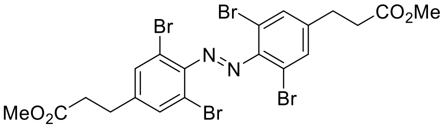

Prepared following general procedure C, using **1k** (209 mg, 0.59 mmol) and $Cu(OTf)_2{\cdot}3.5H_2O$ (40 mol%). Purification of the crude product by silica flash column chromatography (hexane/EtOAc 95:5

to 60:40) afforded the title compound as a red solid (332 mg, 82%). During ^{1}H-NMR analysis, only the *trans* isomer was observed.

^{1}H-NMR (*trans* isomer) (400 MHz, $CDCl_3$) δ 7.54 (s, $C_{Ar}H$, 4 H), 3.71 (s, CO_2CH_3, 6 H), 2.95 (t, J = 7.6 Hz, $C_{Ar}CH_2CH_2CO_2CH_3$, 4 H), 2.67 (t, J = 7.6 Hz, $C_{Ar}CH_2CH_2CO_2CH_3$, 4 H) ppm.

$^{13}C\{^1H\}$-NMR (*trans* isomer) (100 MHz, $CDCl_3$) δ 172.5 (CO_2CH_3), 147.2 ($C_{Ar}N$), 143.8 ($C_{Ar}CH_2$), 133.3 ($C_{Ar}H$), 116.2 ($C_{Ar}H$), 51.9 (CO_2CH_3), 34.9 ($C_{Ar}CH_2CH_2CO_2CH_3$), 29.8 ($C_{Ar}CH_2CH_2CO_2CH_3$) ppm.

HRMS calcd for $C_{20}H_{19}Br_4N_2O_4$ $[M+H]^+$: 666.8073, found 666.8063.

Methyl (*E*)-3-(3,5-dibromo-4-((2,6-dibromophenyl)diazenyl)phenyl)propanoate (2l)

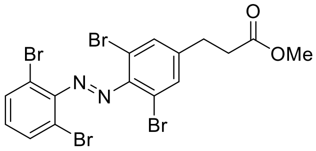

Prepared following general procedure C, using **1l** (158 mg, 0.59 mmol) and $Cu(OTf)_2·3.5H_2O$ (20 mol%). Purification of the crude product by silica flash column chromatography (hexane/EtOAc 90:10) afforded the title compound as a red solid (279 mg, 81%). During ^{1}H-NMR analysis, the product was observed as a mixture of *trans*/*cis* isomers (93:7).

^{1}H-NMR (*trans* isomer) (400 MHz, DMSO-d_6) δ 7.68 (d, J = 8.0 Hz, $C_{Ar}H$, 2 H), 7.55 (s, $C_{Ar}H$, 2 H), 7.09 (t, J = 8.0 Hz, $C_{Ar}H$, 1 H), 3.71 (s, CO_2CH_3, 3 H) 2.96 (t, J = 7.6 Hz, $C_{Ar}CH_2CH_2CO_2CH_3$, 2 H), 2.68 (t, J = 7.6 Hz, $C_{Ar}CH_2CH_2CO_2CH_3$, 2 H).

^{1}H-NMR (*cis* isomer) (400 MHz, DMSO-d_6) δ 7.49 (d, J = 8.0 Hz, $C_{Ar}H$, 2 H), 7.34 (s, $C_{Ar}H$, 2 H), 7.00 (t, J = 8.0 Hz, $C_{Ar}H$, 1 H), 3.67 (s, CO_2CH_3, 3 H) 2.89 (t, J = 7.6 Hz, $C_{Ar}CH_2CH_2CO_2CH_3$, 2 H), 2.62 (t, J = 7.6 Hz, $C_{Ar}CH_2CH_2CO_2CH_3$, 2 H).

$^{13}C\{^1H\}$-NMR (*trans* isomer) (100 MHz, DMSO-d_6) δ 172.6 (CO_2CH_3), 149.2 ($C_{Ar}N$), 147.1 ($C_{Ar}N$), 144.1 ($C_{Ar}CH_2$), 133.5 ($C_{Ar}H$), 133.4 ($C_{Ar}H$), 130.4 ($C_{Ar}H$), 116.5 ($C_{Ar}Br$), 115.9 ($C_{Ar}Br$), 52.0 (CO_2CH_3), 34.3 ($CH_2CO_2CH_3$), 29.2 (CCH_2) ppm. The *cis* isomer was not observed due to the small ratio and intensity of the corresponding signals.

HRMS calcd for $C_{16}H_{13}Br_4N_2O_2$ $[M+H]^+$: 580.7705, found 580.7710.

Methyl (*E*)-3-(3,5-dibromo-4-((2,6-dibromophenyl)diazenyl)benzoate (2m)

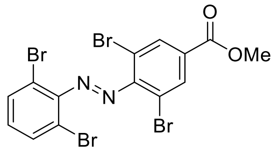

Prepared following general procedure C, using **1m** (142, 0.59 mmol) and $Cu(OTf)_2·3.5H_2O$ (20 mol%). Purification of the crude product by silica flash column chromatography (hexane/EtOAc 100:0 to 95:5) afforded the title compound as a red solid (304 mg, 93%). During ^{1}H-NMR analysis, only the *trans* isomer was observed.

^{1}H-NMR (*trans* isomer) (400 MHz, $CDCl_3$) δ 8.34 (s, $C_{Ar}H$, 2 H), 7.71 (d, J = 8.0 Hz, $C_{Ar}H$, 2 H), 7.13 (t, J = 8.0 Hz, $C_{Ar}H$, 1 H), 3.97 (s, CO_2CH_3, 3 H) ppm.

$^{13}C\{^{1}H\}$-NMR (*trans* isomer) (100 MHz, $CDCl_3$) δ 164.3 (CO_2CH_3), 152.3 ($C_{Ar}N$), 148.5 ($C_{Ar}N$), 134.4 ($C_{Ar}H$), 133.7 ($C_{Ar}H$), 131.8 ($C_{Ar}C$), 131.0 ($C_{Ar}H$), 116.6 ($C_{Ar}Br$), 115.4 ($C_{Ar}Br$), 53.03 (CO_2CH_3) ppm.

HRMS calcd for $C_{14}H_9Br_4N_2O_2$ $[M-H]^-$: 552.7392, found 552.7385.

(*E*)-*N*-(3,5-Dibromo-4-((2,6-dibromophenyl)diazenyl)phenethyl)-2,2,2-trifluoroacetamide (2n)

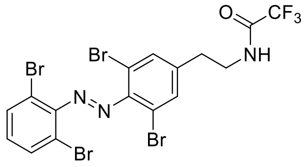

Prepared following general procedure C, using **1n** (189 mg, 0.59 mmol) and $Cu(OTf)_2·3.5H_2O$ (20 mol%). Purification of the crude product by silica flash column chromatography (hexane/EtOAc 100:0 to 90:10) afforded the title compound as a red solid (353 mg, 94%). During ^1H-NMR analysis, the product was observed as a mixture of *trans*/*cis* isomers (87:13).

^1H-NMR (*trans* isomer) (400 MHz, $CDCl_3$) δ 7.68 (d, J = 8.2 Hz, $C_{Ar}H$, 2 H), 7.54 (s, $C_{Ar}H$, 2 H), 7.11 (t, J = 8.2 Hz, $C_{Ar}H$, 1 H), 6.58 (bs, $NHCOCF_3$, 1 H), 3.64 (m, $CH_2CH_2NHCOCF_3$, 2 H), 2.92 (t, J = 7.2 Hz, $CH_2CH_2NHCOCF_3$, 2 H) ppm.

^1H-NMR (*cis* isomer) (400 MHz, $CDCl_3$) δ 7.49 (d, J = 8.0 Hz, $C_{Ar}H$, 2 H), 7.33 (s, $C_{Ar}H$, 2 H), 7.01 (t, J = 8.0 Hz, $C_{Ar}H$, 1 H), 6.46 (bs, $NHCOCF_3$, 1 H), 3.61-3.55 (m, $CH_2CH_2NHCOCF_3$, 2 H), 2.84 (t, J = 7.2 Hz, $CH_2CH_2NHCOCF_3$, 2 H) ppm.

$^{13}C\{^{1}H\}$-NMR (*trans* isomer) (100 MHz, $CDCl_3$) δ 157.6 (q, J = 36.9 Hz, $NHCOCF_3$), 149.1 ($C_{Ar}N$), 147.7 ($C_{Ar}N$), 141.1 ($C_{Ar}C$), 133.7 ($C_{Ar}H$), 133.5 ($C_{Ar}H$), 130.6 ($C_{Ar}H$), 116.6 ($C_{Ar}Br$), 116.0 ($C_{Ar}Br$), 115.8 (q, J = 286.0 Hz, $NHCOCF_3$), 40.8 ($CH_2CH_2NHCOCF_3$), 34.1 ($CH_2CH_2NHCOCF_3$) ppm.

$^{13}C\{^{1}H\}$-NMR (*cis* isomer) (100 MHz, $CDCl_3$) δ 157.5 (q, J = 36.9 Hz, $NHCOCF_3$), 149.6 ($C_{Ar}N$), 148.4 ($C_{Ar}N$), 140.6 ($C_{Ar}C$), 133.5 ($C_{Ar}H$), 133.4 ($C_{Ar}H$), 130.4 ($C_{Ar}H$) ppm. Distinct signals for $C_{Ar}Br$ and $NHCOCF_3$) were not observed, presumably due to overlap and/or the small ratio and intensity of those signals.

^{19}F-NMR (376 MHz, $CDCl_3$) δ –75.8 ppm.

HRMS calcd for $C_{16}H_{11}Br_4F_3N_3O$ $[M+H]^+$: 633.7582, found 633.7603.

(*E*)-*N*-(3,5-Dibromo-4-((2,6-dibromophenyl)diazenyl)benzyl)-2,2,2-trifluoroacetamide (2o)

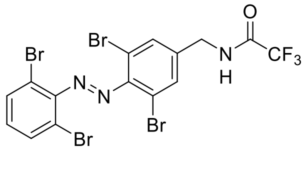

Prepared following general procedure C, using **1o** (181 mg, 0.59 mmol) and $Cu(OTf)_2·3.5H_2O$ (20 mol%). Purification of the crude product by silica flash column chromatography (hexane/EtOAc 100:0 to 90:10) afforded the title compound as a red solid (323 mg, 89%). During ^1H-NMR analysis, only the *trans* isomer was observed.

^1H-NMR (*trans* isomer) (400 MHz, $CDCl_3$) δ 7.69 (d, J = 8.0 Hz, $C_{Ar}H$, 2 H), 7.62 (s, $C_{Ar}H$, 2 H), 7.11 (t, J = 8.0 Hz, $C_{Ar}H$, 1 H), 6.91 (bs, $NHCOCF_3$, 1 H), 4.54 (d, J = 6.0 Hz, $CH_2NHCOCF_3$, 2 H) ppm.

$^{13}C\{^{1}H\}$-NMR (*trans* isomer) (100 MHz, $CDCl_3$) δ 157.6 (q, *J* = 37.6 Hz, NH*C*OCF_3), 148.9 (*C*$_{Ar}$N), 148.8 (*C*$_{Ar}$N), 138.9 (*C*$_{Ar}$C), 133.5 (*C*$_{Ar}$H), 132.8 (*C*$_{Ar}$H), 130.7 (*C*$_{Ar}$H), 116.5 (*C*$_{Ar}$Br), 116.0 (*C*$_{Ar}$Br), 115.8 (q, *J* = 287.8 Hz, NHCO*C*F_3), 42.5 (*C*H_2NHCOCF_3) ppm.

^{19}F-NMR (376 MHz, $CDCl_3$) δ –75.6 ppm.

HRMS calcd for $C_{15}H_9Br_4N_3F_3O$ $[M+H]^+$: 619.7426, found 619.7420.

Methyl (*E*)-3- (3,5-dibromo-4-((2,6-dibromo-4-(2-(2,2,2-trifluoroacetamido)ethyl)phenyl)diazenyl)phenyl)propanoate (2p)

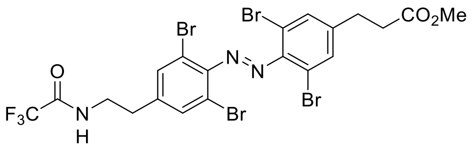

Prepared following general procedure C, using **1p** (839 mg, 2.06 mmol) and $Cu(OTf)_2$·$3.5H_2O$ (40 mol%). Purification of the crude product by silica flash column chromatography (hexane/EtOAc 80:20) afforded the title compound as a red solid (1.22 g, 82%). During ^{1}H-NMR analysis, the product was observed as a mixture of *trans*/*cis* isomers (75:25).

^{1}H-NMR (*trans* isomer) (400 MHz, $CDCl_3$) δ 7.55 (s, C$_{Ar}$*H*, 2 H), 7.52 (s, C$_{Ar}$*H*, 2 H), 6.59 (bs, N*H*COCF_3, 1 H), 3.70 (s, CO_2C*H$_3$*, 3 H), 3.66-3.56 (m, CH_2C*H$_2$*NHCOCF_3, 2 H), 2.96 (t, *J* = 7.6 Hz, C$_{Ar}$C*H$_2$*$CH_2CO_2CH_3$, 2 H), 2.92 (t, *J* = 7.2 Hz, C*H$_2$*CH_2NHCOCF_3, 2 H), 2.67 (t, *J* = 7.6 Hz, C$_{Ar}$$CH_2$C*H$_2$*$CO_2CH_3$, 2 H) ppm.

^{1}H-NMR (*cis* isomer) (400 MHz, $CDCl_3$) δ 7.34 (s, C$_{Ar}$*H*, 2 H), 7.33 (s, C$_{Ar}$*H*, 2 H), 6.50 (bs, N*H*COCF_3, 1 H), 3.67 (s, CO_2C*H$_3$*, 3 H), 3.66-3.56 (m, CH_2C*H$_2$*NHCOCF_3, 2 H), 2.90-2.81 (m, C$_{Ar}$C*H$_2$*$CH_2CO_2CH_3$ + C*H$_2$*CH_2NHCOCF, 4 H), 2.62 (t, *J* = 7.6 Hz, C$_{Ar}$$CH_2$C*H$_2$*$CO_2CH_3$, 2 H) ppm.

$^{13}C\{^{1}H\}$-NMR (*trans* isomer) (100 MHz, $CDCl_3$) δ 172.7 (*C*O_2CH_3), 157.6 (q, *J* = 36.8 Hz, NH*C*OCF_3), 147.9 (*C*$_{Ar}$N), 147.1 (*C*$_{Ar}$N), 144.1 (*C*$_{Ar}$C), 140.9 (*C*$_{Ar}$C), 133.6 (*C*$_{Ar}$H), 133.5 (*C*$_{Ar}$H), 116.4 (*C*$_{Ar}$Br), 116.3 (*C*$_{Ar}$Br), 115.8 (q, *J* = 286.1 Hz, NHCO*C*F_3), 52.1 (CO_2*C*H_3), 40.8 (CH_2*C*H_2NHCOCF_3), 35.0 (CH_2*C*$H_2CO_2CH_3$), 34.0 (*C*H_2CH_2NHCOCF_3), 29.9 (*C*$H_2CH_2CO_2CH_3$) ppm.

$^{13}C\{^{1}H\}$-NMR (*cis* isomer) (100 MHz, $CDCl_3$) δ 169.7 (*C*O_2CH_3), 148.5 (*C*$_{Ar}$N), 147.6 (*C*$_{Ar}$N), 143.7 (*C*$_{Ar}$C), 140.5 (*C*$_{Ar}$C), 133.3 (*C*$_{Ar}$H), 132.3 (*C*$_{Ar}$H), 116.7 (*C*$_{Ar}$Br), 116.2 (*C*$_{Ar}$Br), 52.0 (CO_2*C*H_3), 40.5 (CH_2*C*H_2NHCOCF_3), 34.8 (CH_2*C*$H_2CO_2CH_3$), 33.8 (*C*H_2CH_2NHCOCF_3), 29.7 (*C*$H_2CH_2CO_2CH_3$) ppm. Neither C from the trifluoroacetamide group was observed for the *cis* isomer due to the small ratio and intensity of those signals.

^{19}F-NMR (376 MHz, $CDCl_3$) δ −75.8 ppm.

HRMS calcd for $C_{20}H_{17}Br_4F_3N_3O_3$ $[M+H]^+$: 719.7950, found 719.7926.

(*E*)-1,2-Bis(2,6-dimethoxyphenyl)diazene (3a)

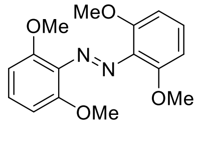

Prepared following general procedure D, starting from **2a** (80 mg, 0.16 mmol). Purification of the crude product by silica flash column chromatography (hexane/EtOAc, 80:20 to 40:60) afforded the title compound as a dark red solid (49 mg, 100%). During ^{1}H-NMR analysis, the product was observed as a mixture of *trans*/*cis* isomers (76:24).

^{1}H-NMR (*trans* isomer) (400 MHz, $CDCl_3$) δ 7.22 (t, *J* = 8.4 Hz, $C_{Ar}H$, 2 H), 6.66 (d, *J* = 8.4 Hz, $C_{Ar}H$, 4 H), 3.85 (s, OCH_3, 12 H) ppm.

^{1}H-NMR (*cis* isomer) (400 MHz, $CDCl_3$) δ 7.03 (t, *J* = 8.4 Hz, $C_{Ar}H$, 2 H), 6.42 (d, *J* = 8.4 Hz, $C_{Ar}H$, 4 H), 3.64 (s, OCH_3, 12 H) ppm.

^{13}C{^{1}H}-NMR (*trans* isomer) (100 MHz, $CDCl_3$) δ 152.4 ($C_{Ar}O$), 134.7 ($C_{Ar}N$), 129.4 ($C_{Ar}H$), 105.3 ($C_{Ar}H$), 56.7 (OCH_3) ppm.

^{13}C{^{1}H}-NMR (*cis* isomer) (100 MHz, $CDCl_3$) δ 150.1 ($C_{Ar}O$), 134.7 ($C_{Ar}N$), 128.2 ($C_{Ar}H$), 104.1 ($C_{Ar}H$), 55.6 (OCH_3) ppm.

HRMS calcd for $C_{16}H_{19}N_2O_4$ $[M+H]^+$: 303.1339, found 303.1342.

(*E*)-1-(2,6-Dimethoxy-4-methylphenyl)-2-(2,6-dimethoxyphenyl)diazene (3b)

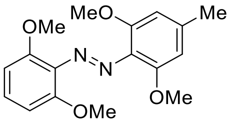

Prepared following general procedure D, starting from **2b** (82 mg, 0.16 mmol). Purification of the crude product by silica flash column chromatography (hexane/EtOAc, 70:30 to 40:60) afforded the title compound as a red solid (44 mg, 87%). During ^{1}H-NMR analysis, the product was observed as a mixture of *trans*/*cis* isomers (47:53).

^{1}H-NMR (*trans* isomer) (400 MHz, $CDCl_3$) δ 7.19 (t, *J* = 8.4 Hz, $C_{Ar}H$, 2 H), 6.65 (d, *J* = 8.4 Hz, $C_{Ar}H$, 2 H), 6.47 (s, $C_{Ar}H$, 2 H), 3.84 (s, OCH_3, 6 H), 3.83 (s, OCH_3, 6 H), 2.37 (s, $C_{Ar}CH_3$, 3 H) ppm.

^{1}H-NMR (*cis* isomer) (400 MHz, $CDCl_3$) δ 7.03 (t, *J* = 8.4 Hz, $C_{Ar}H$, 2 H), 6.42 (d, *J* = 8.4 Hz, $C_{Ar}H$, 2 H), 6.22 (s, $C_{Ar}H$, 2 H), 3.64 (s, OCH_3, 6 H), 3.61 (s, OCH_3, 6 H), 2.25 (s, $C_{Ar}CH_3$, 3 H) ppm.

^{13}C{^{1}H}-NMR (*trans* isomer) (100 MHz, $CDCl_3$) δ 152.6 ($C_{Ar}O$), 152.4 ($C_{Ar}O$), 140.4 ($C_{Ar}C$), 134.9 ($C_{Ar}N$), 132.6 ($C_{Ar}N$), 129.0 ($C_{Ar}H$), 106.1 ($C_{Ar}H$), 105.3 ($C_{Ar}H$), 56.7 (OCH_3), 56.6 (OCH_3), 22.5 ($C_{Ar}CH_3$) ppm.

^{13}C{^{1}H}-NMR (*cis* isomer) (100 MHz, $CDCl_3$) δ 150.0 ($C_{Ar}O$), 149.8 ($C_{Ar}O$), 138.6 ($C_{Ar}C$), 134.8 ($C_{Ar}N$), 132.3 ($C_{Ar}N$), 127.9 ($C_{Ar}H$), 104.9 ($C_{Ar}H$), 104.2 ($C_{Ar}H$), 55.6 (OCH_3), 55.5 (OCH_3), 22.2 ($C_{Ar}CH_3$) ppm.

HRMS calcd for $C_{17}H_{21}N_2O_4$ $[M+H]^+$: 317.1496, found 317.1493.

(*E*)-1,2-Bis(2,6-dimethoxyphenyl-4-methylphenyl)diazene (3c)

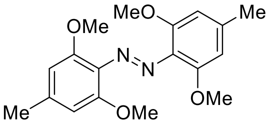

Prepared following general procedure D, starting from **2c** (84 mg, 0.16 mmol). Purification of the crude product by silica flash column chromatography (hexane/EtOAc, 70:30 to 50:50) afforded the title compound as a red solid (39 mg, 74%). During ^{1}H-NMR analysis, the product was observed as a mixture of *trans*/*cis* isomers (88:12).

^{1}H-NMR (*trans* isomer) (400 MHz, $CDCl_3$) δ 6.46 (s, $C_{Ar}H$, 4 H), 3.84 (s, OCH_3, 12 H), 2.36 (s, CCH_3, 6 H) ppm.

^{1}**H-NMR (*cis* isomer)** (400 MHz, $CDCl_3$) δ 6.23 (s, $C_{Ar}H$, 4 H), 3.62 (s, OCH_3, 12 H), 2.26 (s, CCH_3, 6 H) ppm.

^{13}**C{^{1}H}-NMR (*trans* isomer)** (100 MHz, $CDCl_3$) δ 152.6 ($C_{Ar}O$), 140.0 ($C_{Ar}C$), 132.5 ($C_{Ar}N$), 106.1 ($C_{Ar}H$), 56.6 (OCH_3), 22.5 ($C_{Ar}CH_3$).

^{13}**C{^{1}H}-NMR (*cis* isomer)** (100 MHz, $CDCl_3$) δ 149.8 ($C_{Ar}O$), 138.3 ($C_{Ar}C$), 132.8 ($C_{Ar}N$), 105.0 ($C_{Ar}H$), 55.6 (OCH_3), 22.2 ($C_{Ar}CH_3$).

HRMS calcd for $C_{18}H_{23}N_2O_4$ $[M+H]^+$: 331.1652, found 331.1654.

(*E*)-1-(2,6-Dimethoxy-4-methoxyphenyl)-2-(2,6-dimethoxyphenyl)diazene (3d)

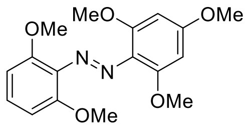

Prepared following general procedure D, starting from **2d** (85 mg, 0.16 mmol). Purification of the crude product by silica flash column chromatography (hexane/EtOAc, 60:40 to 40:60) afforded the title compound as a red solid (37 mg, 70%). During ^{1}H-NMR analysis, the product was observed as a mixture of *trans*/*cis* isomers (57:43).

^{1}**H-NMR (*trans* isomer)** (400 MHz, $CDCl_3$) δ 7.18 (t, J = 8.4 Hz, $C_{Ar}H$, 1 H), 6.65 (d, J = 8.4 Hz, $C_{Ar}H$, 2 H), 6.22 (s, $C_{Ar}H$, 2 H), 3.86 (s, OCH_3, 6 H), 3.86 (s, OCH_3, 3 H), 3.83 (s, OCH_3, 6 H) ppm.

^{1}**H-NMR (*cis* isomer)** (400 MHz, $CDCl_3$) δ 7.03 (t, J = 8.4 Hz, $C_{Ar}H$, 1 H), 6.44 (d, J = 8.4 Hz, $C_{Ar}H$, 2 H), 5.98 (s, $C_{Ar}H$, 2 H), 3.74 (s, OCH_3, 3 H), 3.65 (s, OCH_3, 6 H), 3.61 (s, OCH_3, 6 H), ppm.

^{13}**C{^{1}H}-NMR (*trans* isomer)** (100 MHz, $CDCl_3$) δ 161.7 (p-$C_{Ar}O$), 154.7 (o-$C_{Ar}O$), 152.4 (o-$C_{Ar}O$), 135.0 ($C_{Ar}N$), 128.9 ($C_{Ar}N$), 128.7 ($C_{Ar}H$), 105.4 ($C_{Ar}H$), 91.6 ($C_{Ar}H$), 56.7 (OCH_3), 55.7 (OCH_3), 55.6 (OCH_3).

^{13}**C{^{1}H}-NMR (*cis* isomer)** (100 MHz, $CDCl_3$) δ 160.3 (p-$C_{Ar}O$), 151.0 (o-$C_{Ar}O$), 149.9 (o-$C_{Ar}O$), 135.0 ($C_{Ar}N$), 129.4 ($C_{Ar}N$), 127.8 ($C_{Ar}H$), 104.3 ($C_{Ar}H$), 90.6 ($C_{Ar}H$), 56.7 (OCH_3), 55.6 (OCH_3), 55.4 (OCH_3).

HRMS calcd for $C_{17}H_{21}N_2O_5$ $[M+H]^+$: 333.1445, found 333.1456.

(*E*)-1,2-Bis(2,6-dimethoxyphenyl-4-chlorophenyl)diazene (3e)

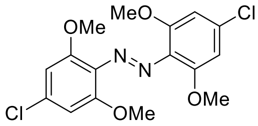

Prepared following general procedure D, starting from **2e** (91 mg, 0.16 mmol). Purification of the crude product by silica flash column chromatography (hexane/EtOAc, 90:10 to 60:40) afforded the title compound as a red solid (45 mg, 76%). During ^{1}H-NMR analysis, the product was observed as a mixture of *trans*/*cis* isomers (83:17).

^{1}**H-NMR (*trans* isomer)** (400 MHz, $CDCl_3$) δ 6.67 (s, $C_{Ar}H$, 4 H), 3.84 (s, OCH_3, 12 H).

^{1}**H-NMR (*cis* isomer)** (400 MHz, $CDCl_3$) δ 6.45 (s, $C_{Ar}H$, 4 H), 3.66 (s, OCH_3, 12 H).

^{13}**C{^{1}H}-NMR (*trans* isomer)** (100 MHz, $CDCl_3$) δ 152.9 ($C_{Ar}O$), 135.3 (C_{Ar}), 133.9 (C_{Ar}), 106.0 ($C_{Ar}H$), 56.9 (OCH_3).

$^{13}C\{^1H\}$**-NMR (*cis* isomer)** (100 MHz, $CDCl_3$) δ 150.2 (C_{Ar}O), 133.9 (C_{Ar}), 132.8 (C_{Ar}), 105.1 (C_{Ar}H), 55.9 (OCH$_3$).

HRMS calcd for $C_{16}H_{17}Cl_2N_2O_4$ $[M+H]^+$: 371.0560, found 371.0567.

(*E*)-1,2-Bis(2,6-dimethoxyphenyl-4-bromophenyl)diazene (3f)

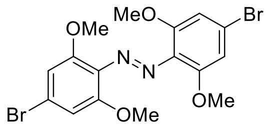

Prepared following general procedure D, starting from **2f** (105 mg, 0.16 mmol) at 90 °C. Purification of the crude product by silica flash column chromatography (hexane/EtOAc, 90:10 to 60:40) afforded the title compound as a red solid (36 mg, 49%). During ^{1}H-NMR analysis, the product was observed as a mixture of *trans*/*cis* isomers (46:54).

^1H**-NMR (*trans* isomer)** (400 MHz, $CDCl_3$) δ 6.82 (s, C$_{Ar}$*H*, 4 H), 3.84 (s, OCH_3, 12 H).

^1H**-NMR (*cis* isomer)** (400 MHz, $CDCl_3$) δ 6.60 (s, C$_{Ar}$*H*, 4 H), 3.66 (s, OCH_3, 12 H).

$^{13}C\{^1H\}$**-NMR (*trans* isomer)** (100 MHz, $CDCl_3$) δ 152.9 (C_{Ar}O), 133.2 (C_{Ar}N), 123.0 (C_{Ar}Br), 109.0 (C_{Ar}H), 56.9 (OCH$_3$).

$^{13}C\{^1H\}$**-NMR (*cis* isomer)** (100 MHz, $CDCl_3$) δ 150.2 (C_{Ar}O), 133.2 (C_{Ar}N), 121.6 (C_{Ar}Br), 108.1 (C_{Ar}H), 55.9 (OCH$_3$).

HRMS calcd for $C_{16}H_{17}Br_2N_2O_4$ $[M+H]^+$: 458.9550, found 458.9549.

(*E*)-1,2-Bis(2,6-dimethoxyphenyl-4-iodophenyl)diazene (3g)

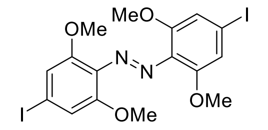

Prepared following general procedure D, starting from **2g** (112 mg, 0.16 mmol) at 90 °C. Purification of the crude product by silica flash column chromatography (hexane/EtOAc, 90:10 to 50:50) afforded the title compound as a red solid (71 mg, 80%). During ^{1}H-NMR analysis, the product was observed as a mixture of *trans*/*cis* isomers (92:8).

^1H**-NMR (*trans* isomer)** (400 MHz, $CDCl_3$) δ 7.00 (s, C$_{Ar}$*H*, 4 H), 3.83 (s, OCH_3, 12 H) ppm.

^1H**-NMR (*cis* isomer)** (400 MHz, $CDCl_3$) δ 6.78 (s, C_{Ar}H, 4 H), 3.65 (s, OCH_3, 12 H) ppm

$^{13}C\{^1H\}$**-NMR (*trans* isomer)** (100 MHz, $CDCl_3$) δ 152.6 (C_{Ar}O), 134.0 (C_{Ar}N), 115.1 (C_{Ar}H), 94.0 (C_{Ar}I), 57.0 (OCH$_3$).

$^{13}C\{^1H\}$**-NMR (*cis* isomer)** (100 MHz, $CDCl_3$) δ 150.1 (C_{Ar}O), 134.0 (C_{Ar}N), 114.1 (C_{Ar}H), 92.3 (C_{Ar}I), 56.0 (OCH$_3$).

HRMS calcd for $C_{16}H_{17}N_2I_2O_4$ $[M+H]^+$: 554.9272, found 554.9264.

(*E*)-1,2-Bis(2,6-dimethoxyphenyl-4-trifluoromethylphenyl)diazene (3h)

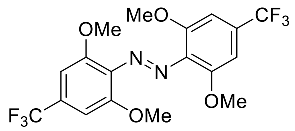

Prepared following general procedure C, starting from **1h** (188 mg, 0.59 mmol) and $Cu(OTf)_2 \cdot 3.5H_2O$ (40 mol%). Purification of the crude product by silica flash column chromatography (hexane) afforded the corresponding tetra-*ortho*-brominated azobenzene along with some inseparable impurities. The product thus obtained was used directly in the next step. The methoxylation step was performed following general procedure D, starting from the impure brominated intermediate. Purification of the crude methoxylated product by silica flash column chromatography (hexane/EtOAc, 90:10 to 85:15) afforded the title compound as a red solid (59 mg, 23% over two steps). During ^{1}H-NMR analysis, the product was observed as a mixture of *trans*/*cis* isomers (90:10).

^{1}H-NMR (*trans* isomer) (400 MHz, $CDCl_3$) δ 6.91 (s, $C_{Ar}H$, 4 H), 3.89 (s, OCH_3, 12 H) ppm.

^{1}H-NMR (*cis* isomer) (400 MHz, $CDCl_3$) δ 6.69 (s, $C_{Ar}H$, 4 H), 3.71 (s, OCH_3, 12 H) ppm.

^{13}C{^{1}H}-NMR (*trans* isomer) (100 MHz, $CDCl_3$) δ 152.5 ($C_{Ar}O$), 136.3 ($C_{Ar}N$), 131.7 (q, J = 32.6 Hz, CCF_3), 124.2 (q, J = 270.9 Hz, CF_3), 102.6 (q, J = 3.8 Hz, $C_{Ar}H$), 57.2 (OCH_3) ppm.

^{13}C{^{1}H}-NMR (*cis* isomer) (100 MHz, $CDCl_3$) δ 150.2 ($C_{Ar}O$), 136.3 ($C_{Ar}N$), 130.9 (q, J = 32.6 Hz, CCF_3), 121.4 (q, J = 270.9 Hz, CF_3), 101.8 (q, J = 3.8 Hz, $C_{Ar}H$), 56.3 (OCH_3) ppm.

^{19}F-NMR (376 MHz, $CDCl_3$) δ −62.8 ppm.

HRMS calcd for $C_{18}H_{17}F_6N_2O_4$ $[M+H]^+$: 439.1087, found 439.1084.

(*E*)-3-(3,5-Dimethoxy-4-((2,6-dimethoxyphenyl)diazenyl)phenyl)propanoic acid (3i)

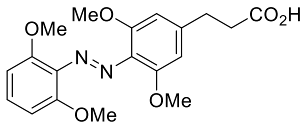

Prepared following general procedure D, starting from **2i** (91 mg, 0.16 mmol). Purification of the crude product by silica flash column chromatography (hexane/EtOAc, 70:30 to 0:100 + 1% AcOH) afforded the title compound as a very dark solid (40 mg, 66%). During ^{1}H-NMR analysis, only the *trans* isomer was observed. Some minor impurities are observed during NMR analysis, which could not be removed chromatographically.

^{1}H-NMR (*trans* isomer) (400 MHz, $CDCl_3$) δ 7.21 (t, J = 8.4 Hz, $C_{Ar}H$, 1 H), 6.65 (d, J = 8.4 Hz, $C_{Ar}H$, 2 H), 6.52 (s, $C_{Ar}H$, 2 H), 3.83 (s, OCH_3, 12 H), 2.96 (t, J = 8.0 Hz, $C_{Ar}CH_2CH_2CO_2H$, 2 H), 2.70 (t, J = 8.0 Hz, $C_{Ar}CH_2CH_2CO_2H$, 2 H) ppm. The carboxylic acid proton was not observed.

^{13}C{^{1}H}-NMR (*trans* isomer) (100 MHz, $CDCl_3$) δ 177.8 (CO_2H), 152.6 ($C_{Ar}O$), 152.3 ($C_{Ar}O$), 142.4 ($C_{Ar}C$), 134.6 ($C_{Ar}N$), 132.8 ($C_{Ar}N$), 129.2 ($C_{Ar}H$), 105.3 ($C_{Ar}H$), 105.2 ($C_{Ar}H$), 56.6 (OCH_3), 56.6 (OCH_3), 35.5 ($C_{Ar}CH_2CH_2CO_2H$), 31.4 ($C_{Ar}CH_2CH_2CO_2H$) ppm.

HRMS calcd for $C_{19}H_{21}N_2O_6$ $[M-H]^-$: 373.1405, found 373.1405.

(*E*)-3-(3,5-Dimethoxy-4-((2,6-dimethoxyphenyl)diazenyl)benzoic acid (3j)

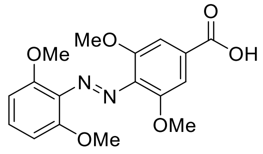

Prepared following general procedure D, starting from **2j** (87 mg, 0.16 mmol). Purification of the crude product by silica flash column chromatography (hexane/EtOAc, 50:50 + 1% AcOH to 0:100 + 1% AcOH) afforded the title compound as a red/orange solid (49 mg, 84%). During ^{1}H-NMR analysis, only the *trans* isomer was observed.

^{1}H-NMR (*trans* isomer) (400 MHz, DMSO-d_6) δ 7.33 (m, $C_{Ar}H$, 3 H), 6.80 (d, J = 8.4 Hz, $C_{Ar}H$, 2 H), 3.77 (s, OCH_3, 6 H), 3.74 (s, OCH_3, 6 H) ppm. A broadening of all the signals for the aromatic ring containing the carboxylic acid is observed. This led to the carboxylic acid proton not being observed and the singlet expected for the aromatic proton becoming a very broad signal at 7.33 ppm.

^{13}C{^{1}H}-NMR (*trans* isomer) (100 MHz, DSMO-d_6) δ 152.0 ($C_{Ar}O$), 150.8 (broad signal, $C_{Ar}O$) 149.3 ($C_{Ar}C$), 136.2 (broad signal, $C_{Ar}N$), 132.9 ($C_{Ar}N$), 130.5 ($C_{Ar}H$), 106.2 (broad signal, $C_{Ar}H$), 105.3 ($C_{Ar}H$), 56.2 (OCH_3), 56.2 (OCH_3) ppm. A broadening of all the signals for the aromatic ring containing the carboxylic acid is observed. This led to the carbonyl carbon not being observed.

HRMS calcd for $C_{17}H_{17}N_2O_6$ $[M-H]^-$: 345.1092, found 345.1106.

Dimethyl 3,3'-(diazene-1,2-diylbis(3,5-dimethoxy-4,1-phenylene))(*E*)-dipropionate (3k)

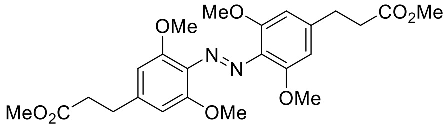

Prepared following general procedure D, starting from **2k** (107 mg, 0.16 mmol) under anhydrous conditions. Purification of the crude product by silica flash column chromatography (hexane/EtOAc, 80:20 to 50:50) afforded the title compound as a red solid (50 mg, 65%). During ^{1}H-NMR analysis, the product was observed as a mixture of *trans*/*cis* isomers (83:17).

^{1}H-NMR (*trans* isomer) (400 MHz, $CDCl_3$) δ 6.49 (s, $C_{Ar}H$, 4 H). 3.81 (s, OCH_3, 12 H), 3.67 (s, CO_2CH_3, 6 H), 2.94 (t, J = 7.8 Hz, $C_{Ar}CH_2CH_2CO_2CH_3$, 4 H), 2.64 (t, J = 7.8 Hz, $C_{Ar}CH_2CH_2CO_2CH_3$, 4 H) ppm.

^{1}H-NMR (*cis* isomer) (400 MHz, $CDCl_3$) δ 6.25 (s, $C_{Ar}H$, 4 H). 3.63 (s, CO_2CH_3, 6 H), 3.59 (s, OCH_3, 12 H), (s, CO_2CH_3, 6 H), 2.84 (t, J = 7.8 Hz, $C_{Ar}CH_2CH_2CO_2CH_3$, 4 H), 2.57 (t, J = 7.8 Hz, $C_{Ar}CH_2CH_2CO_2CH_3$, 4 H) ppm.

^{13}C{^{1}H}-NMR (*trans* isomer) (100 MHz, $CDCl_3$) δ 173.3 (CO_2CH_3), 152.6 ($C_{Ar}O$), 142.5 ($C_{Ar}C$), 133.1 ($C_{Ar}N$), 105.4 ($C_{Ar}H$), 56.7 (OCH_3), 51.8 (CO_2CH_3), 35.8 ($C_{Ar}CH_2CH_2CO_2CH_3$), 31.8 ($C_{Ar}CH_2CH_2CO_2CH_3$) ppm.

^{13}C{^{1}H}-NMR (*trans* isomer) (100 MHz, $CDCl_3$) δ 173.3 (CO_2CH_3), 149.9 ($C_{Ar}O$), 141.1 ($C_{Ar}C$), 133.2 ($C_{Ar}N$), 104.1 ($C_{Ar}H$), 55.6 (OCH_3), 51.7 (CO_2CH_3), 31.6 ($C_{Ar}CH_2CH_2CO_2CH_3$), 29.8 ($C_{Ar}CH_2CH_2CO_2CH_3$) ppm.

HRMS calcd for $C_{24}H_{31}N_2O_8$ $[M+H]^+$: 475.2075, found 475.2060.

Methyl (*E*)-3-(3,5-dimethoxy-4-((2,6-dimethoxyphenyl)diazenyl)phenyl)propanoate (3l)

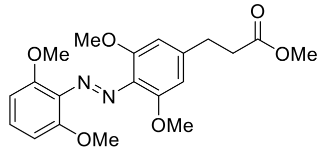

Prepared following general procedure D, starting from **2l** (93 mg, 0.16 mmol) under anhydrous conditions. Purification of the crude product by silica flash column chromatography (hexane/EtOAc, 100:0 to 50:50) afforded the title compound as a red solid (39 mg, 62%). During ^{1}H-NMR analysis, the product was observed as a mixture of *trans*/*cis* isomers (69:31).

^{1}H-NMR (*trans* isomer) (400 MHz, $CDCl_3$) δ 7.19 (t, *J* = 8.4 Hz, $C_{Ar}H$, 2 H), 6.64 (d, *J* = 8.4 Hz, $C_{Ar}H$, 2 H), 6.50 (s, $C_{Ar}H$, 2 H), 3.82 (s, OCH_3, 12 H), 3.67 (s, CO_2CH_3, 3 H), 2.95 (t, *J* = 8.0 Hz, $C_{Ar}CH_2CH_2CO_2CH_3$, 2 H), 2.65 (t, *J* = 8.0 Hz, $C_{Ar}CH_2CH_2CO_2CH_3$, 2 H) ppm.

^{1}H-NMR (*cis* isomer) (400 MHz, CDCl3) δ 7.02 (t, *J* = 8.4 Hz, $C_{Ar}H$, 2 H), 6.41 (d, *J* = 8.4 Hz, $C_{Ar}H$, 2 H), 6.24 (s, $C_{Ar}H$, 2 H), 3.61 (m, OCH_3 + CO_2CH_3, 15 H), 2.83 (t, *J* = 8.0 Hz, $C_{Ar}CH_2CH_2CO_2CH_3$, 2 H), 2.56 (t, *J* = 8.0 Hz, $C_{Ar}CH_2CH_2CO_2CH_3$, 2 H) ppm.

^{13}C{^{1}H}-NMR (*trans* isomer) (100 MHz, $CDCl_3$) δ 173.3 (CO_2CH_3), 152.6 ($C_{Ar}O$), 152.4 ($C_{Ar}O$), 142.7 ($C_{Ar}C$), 134.7 ($C_{Ar}N$), 133.1 ($C_{Ar}N$), 129.2 ($C_{Ar}H$), 105.3 ($C_{Ar}H$), 104.1 ($C_{Ar}H$), 56.7 (OCH_3), 56.6 (OCH_3), 51.8 (CO_2CH_3), 35.8 ($C_{Ar}CH_2CH_2CO_2CH_3$), 31.8 ($C_{Ar}CH_2CH_2CO_2CH_3$) ppm.

^{13}C{^{1}H}-NMR (*cis* isomer) (100 MHz, $CDCl_3$) δ 173.2 (CO_2CH_3), 150.0 ($C_{Ar}O$), 150.0 ($C_{Ar}O$), 141.1 ($C_{Ar}C$), 134.7 ($C_{Ar}N$), 132.9 ($C_{Ar}N$), 128.1 ($C_{Ar}H$), 105.3 ($C_{Ar}H$), 104.1 ($C_{Ar}H$), 55.6 (OCH_3), 55.6 (OCH_3), 51.7 (CO_2CH_3), 35.7 ($C_{Ar}CH_2CH_2CO_2CH_3$), 31.5 ($C_{Ar}CH_2CH_2CO_2CH_3$) ppm.

HRMS calcd for $C_{20}H_{25}N_2O_6$ $[M+H]^+$: 389.1707, found 389.1707.

Methyl (*E*)-3-(3,5-dimethoxy-4-((2,6-dimethoxyphenyl)diazenyl)benzoate (3m)

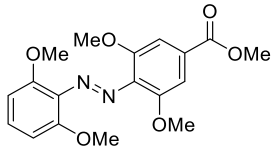

Prepared following general procedure D starting from **2m** (89 mg, 0.16 mmol) under anhydrous conditions. Purification of the crude product by silica flash column chromatography (hexane/EtOAc, 80:20 to 40:60) afforded the title compound as a red solid (57 mg, 75%). During ^{1}H-NMR analysis, the product was observed as a mixture of *trans*/*cis* isomers (80:20).

^{1}H-NMR (*trans* isomer) (400 MHz, $CDCl_3$) δ 7.36 (s, $C_{Ar}H$, 2 H), 7.25 (t, *J* = 8.4 Hz, $C_{Ar}H$, 1 H), 6.67 (d, *J* = 8.4 Hz, $C_{Ar}H$, 2 H), 3.94 (s, CO_2CH_3, 3 H), 3.88 (s, OCH_3, 6 H), 3.86 (s, OCH_3, 6 H) ppm.

^{1}H-NMR (*cis* isomer) (400 MHz, $CDCl_3$) δ 7.13 (s, $C_{Ar}H$, 2 H), 7.04 (t, *J* = 8.4 Hz, $C_{Ar}H$, 1 H), 6.41 (d, *J* = 8.4 Hz, $C_{Ar}H$, 2 H), 3.87 (s, CO_2CH_3, 3 H), 3.70 (s, OCH_3, 6 H), 3.64 (s, OCH_3, 6 H) ppm.

^{13}C{^{1}H}-NMR (*trans* isomer) (101 MHz, $CDCl_3$) δ 166.7 (CO_2CH_3), 152.7 ($C_{Ar}O$), 151.6 ($C_{Ar}O$), 137.7 (C_{Ar}), 134.2 (C_{Ar}), 130.3 ($C_{Ar}H$), 129.4 (C_{Ar}), 106.6 ($C_{Ar}H$), 105.2 ($C_{Ar}H$), 56.8 (OCH_3), 56.7 (OCH_3), 52.5 (CO_2CH_3) ppm.

^{13}C{^{1}H}-NMR (*cis* isomer) (101 MHz, $CDCl_3$) δ 166.6 (CO_2CH_3), 150.0 ($C_{Ar}O$), 149.6 ($C_{Ar}O$), 137.8 (C_{Ar}), 134.5 (C_{Ar}), 130.1 ($C_{Ar}H$), 128.7 (C_{Ar}), 105.6 ($C_{Ar}H$), 104.1 ($C_{Ar}H$), 55.8 (OCH_3), 55.6 (OCH_3), 52.4 (CO_2CH_3) ppm.

HRMS calcd for $C_{18}H_{21}N_2O_6$ $[M+H]^+$: 361.1394, found 361.1392.

(*E*)-*N*-(3,5-Dimethoxy-4-((2,6-dimethoxyphenyl)diazenyl)phenethyl)-2,2,2-trifluoroacetamide (3n)

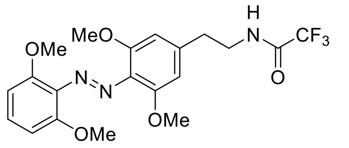

Prepared following general procedure D, starting from **2n** (102 mg, 0.16 mmol) at 90 °C under anhydrous conditions. Purification of the crude product by silica flash column chromatography (hexane/EtOAc, 90:10 to 40:60) afforded the title compound as a red solid (41 mg, 58%). During ^{1}H-NMR analysis, the product was observed as a mixture of *trans*/*cis* isomers (63:37). Some minor impurities are observed during NMR analysis, which could not be removed chromatographically.

^{1}H-NMR (*trans* isomer) (400 MHz, $CDCl_3$) δ 7.22 (t, *J* = 8.4 Hz, $C_{Ar}H$, 2 H), 6.74-6.60 (m, $C_{Ar}H$ + N*H*$COCF_3$, 3 H), 6.46 (s, $C_{Ar}H$, 2 H), 3.82 (s, OCH_3, 6 H), 3.80 (s, OCH_3, 6 H), 3.66-3.56 (m, $CH_2NHCOCF_3$, 2 H), 2.87 (t, *J* = 6.8 Hz, $C_{Ar}CH_2CH_2NH$, 2 H) ppm.

^{1}H-NMR (*cis* isomer) (400 MHz, $CDCl_3$) δ 7.04 (t, *J* = 8.4 Hz, $C_{Ar}H$, 2 H), 6.42 (d, *J* = 8.4 Hz, $C_{Ar}H$, 2 H), 6.22 (s, $C_{Ar}H$, 2 H), 3.62 (s, OCH_3, 6 H), 3.58 (s, OCH_3, 6 H), 3.57-3.49 (m, $CH_2NHCOCF_3$, 2 H), 2.76 (t, *J* = 6.8 Hz, $C_{Ar}CH_2CH_2NH$, 2 H) ppm.

^{13}C{^{1}H}-NMR (*trans* isomer) (100 MHz, $CDCl_3$) δ 157.3 (q, *J* = 36.6 Hz, $NHCOCF_3$), 152.6 ($C_{Ar}O$), 152.4 ($C_{Ar}O$), 139.6 (C_{Ar}), 139.6 (C_{Ar}), 134.4 (C_{Ar}), 129.5 ($C_{Ar}H$), 115.8 (q, *J* = 286 Hz, $NHCOCF_3$) 105.5 ($C_{Ar}H$), 105.2 ($C_{Ar}H$), 56.6 (OCH_3), 56.5 (OCH_3), 40.9 ($CH_2NHCOCF_3$), 35.5 ($C_{Ar}CH_2CH_2NH$) ppm.

^{13}C{^{1}H}-NMR (*cis* isomer) (100 MHz, $CDCl_3$) δ 157.2 (q, *J* = 36.6 Hz, $NHCOCF_3$), 150.1 ($C_{Ar}O$), 149.9 ($C_{Ar}O$), 138.3(C_{Ar}), 138.3(C_{Ar}), 133.2 (C_{Ar}), 128.3 ($C_{Ar}H$), 115.8 (q, *J* = 286 Hz, $NHCOCF_3$) 104.3 ($C_{Ar}H$), 104.0 ($C_{Ar}H$), 55.5 (OCH_3), 55.4 (OCH_3), 40.7 ($CH_2NHCOCF_3$), 35.3 ($C_{Ar}CH_2CH_2NH$) ppm.

^{19}F-NMR (376 MHz, $CDCl_3$) δ −75.9 ppm.

HRMS calcd for $C_{20}H_{23}F_3N_3O_5$ $[M+H]^+$: 442.1548, found 442.1585.

(*E*)-*N*-(3,5-Dimethoxy-4-((2,6-dimethoxyphenyl)diazenyl)benzyl)-2,2,2-trifluoroacetamide (3o)

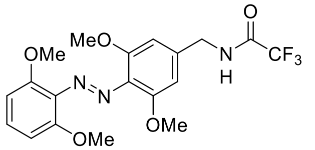

Prepared following general procedure D, starting from **2o** (100 mg, 0.16 mmol) at 70 °C under anhydrous conditions. Purification of the crude product by silica flash column chromatography (hexane/EtOAc, 80:20 to 50:50) afforded the title compound as a red solid (16 mg, 24%). During ^{1}H-NMR analysis, the product was observed as a mixture of *trans*/*cis* isomers (71:29). Some minor impurities are observed during NMR analysis, which could not be removed chromatographically.

^{1}H-NMR (*trans* isomer) (400 MHz, $CDCl_3$) δ 7.24 (t, *J* = 8.0 Hz, 1 H), 6.67 (d, *J* = 8.0 Hz, 2 H), 6.49 (s, 2 H), 4.44 (d, *J* = 5.6 Hz, 2 H), 3.83 (s, 6 H), 3.78 (s, 6 H) ppm. The amide proton was not observed.

^{1}H-NMR (*cis* isomer) (400 MHz, $CDCl_3$) δ 7.05 (t, *J* = 8.0 Hz, $C_{Ar}H$, 1 H), 6.43 (d, *J* = 8.0 Hz, 2 H), 6.27 (s, 2 H), 4.34 (d, *J* = 5.6 Hz, 2 H), 3.63 (s, 6 H), 3.58 (s, 6 H) ppm. The amide proton was not observed.

^{13}C{^{1}H}-NMR (*trans* isomer) (101 MHz, $CDCl_3$) δ 157.5 (q, *J* = 36.9 Hz, $NHCOCF_3$), 152.6 (C_{Ar}), 152.4 (C_{Ar}), 137.9 (C_{Ar}), 137.9 (C_{Ar}), 134.3 (C_{Ar}), 129.8 ($C_{Ar}H$), 116.1 (q, *J* = 285.9 Hz, $NHCOCF_3$), 105.3 ($C_{Ar}H$), 104.9 ($C_{Ar}H$), 56.7 (OCH_3), 56.6 (OCH_3), 44.2 ($CH_2NHCOCF_3$) ppm.

^{13}C{^{1}H}-NMR (*cis* isomer) (101 MHz, $CDCl_3$) δ 157.5 (q, *J* = 36.9 Hz, $NHCOCF_3$), 150.2 (C_{Ar}), 150.0 (C_{Ar}), 136.6 (C_{Ar}), 136.6 (C_{Ar}), 133.7 (C_{Ar}), 128.6 ($C_{Ar}H$), 116.1 (q, *J* = 285.9 Hz, $NHCOCF_3$), 104.3 ($C_{Ar}H$), 103.7 ($C_{Ar}H$), 55.7 (OCH_3), 55.5 (OCH_3), 44.0 ($CH_2NHCOCF_3$) ppm.

^{19}F-NMR (376 MHz, $CDCl_3$) δ −75.8 ppm.

HRMS calcd for $C_{19}H_{21}N_3F_3O_5$ $[M+H]^+$: 428.1428, found 428.1426.

Methyl (*E*)-3- (3,5-dimethoxy-4-((2,6-dimethoxy-4-(2-(2,2,2-trifluoroacetamido)ethyl)phenyl)diazenyl)phenyl)propanoate (3p)

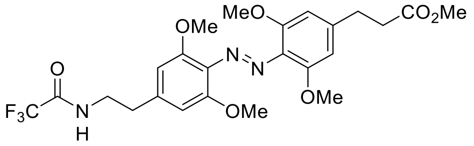

Prepared following general procedure D, starting from **2p** (116 mg, 0.16 mmol) at 90 °C under anhydrous conditions. Purification of the crude product by silica flash column chromatography (hexane/EtOAc 50:50) afforded the title compound as a red solid (39 mg, 47%). During ^{1}H-NMR analysis, the product was observed as a mixture of *trans*/*cis* isomers (55:45).

^{1}H-NMR (*trans* isomer) (400 MHz, $CDCl_3$) δ 6.70 (bs, $NHCOCF_3$, 1 H), 6.50 (s, $C_{Ar}H$, 2 H), 6.46 (s, $C_{Ar}H$, 2 H), 3.82 (s, OCH_3, 6 H), 3.80 (s, OCH_3, 6 H), 3.68 (s, CO_2CH_3, 3 H), 3.57-3.51 (m, $C_{Ar}CH_2CH_2NHCOCF_3$, 2 H), 2.95 (t, *J* = 7.8 Hz, $C_{Ar}CH_2CH_2CO_2CH_3$, 2 H), 2.88 (t, *J* = 6.8 Hz, $C_{Ar}CH_2CH_2NHCOCF_3$, 2 H), 2.65 (t, *J* = 7.8 Hz, $C_{Ar}CH_2CH_2CO_2CH_3$, 2 H) ppm.

^{1}H-NMR (*cis* isomer) (400 MHz, $CDCl_3$) δ 6.25 (s, $C_{Ar}H$, 2 H), 6.23 (s, $C_{Ar}H$, 2 H), 5.92 (bs, $NHCOCF_3$, 1 H), 3.63 (s, $C_{Ar}OCH_3$, 3 H), 3.60 (s, $C_{Ar}OCH_3$, 6 H), 3.58 (s, CO_2CH_3, 6 H), 3.47 (m, $C_{Ar}CH_2CH_2NHCOCF_3$, 2 H), 2.83 (t, *J* = 7.8 Hz, $C_{Ar}CH_2CH_2CO_2CH_3$, 2 H), 2.77 (t, *J* = 6.8 Hz, $C_{Ar}CH_2CH_2NHCOCF_3$, 2 H), 2.57 (t, *J* = 7.8 Hz, $C_{Ar}CH_2CH_2CO_2CH_3$, 2 H) ppm.

^{13}C{^{1}H}-NMR (*trans* isomer) (101 MHz, $CDCl_3$) δ 173.3 (CO_2CH_3), 157.4 (q, *J* = 37.0 Hz, $NHCOCF_3$), 152.7 ($C_{Ar}O$ x2), 143.0 ($C_{Ar}C$), 141.4 ($C_{Ar}C$), 133.4 ($C_{Ar}N$), 133.4 ($C_{Ar}N$), 115.9 (q, *J* = 287.0 Hz, $NHCOCF_3$), 105.7 ($C_{Ar}H$), 104.5 ($C_{Ar}H$), 56.7 (OCH_3), 56.6 (OCH_3), 51.9 (CO_2CH_3), 41.0 ($C_{Ar}CH_2CH_2NHCOCF_3$), 35.7 ($C_{Ar}CH_2CH_2CO_2CH_3$), 35.6 ($C_{Ar}CH_2CH_2NHCOCF_3$), 31.8 ($C_{Ar}CH_2CH_2CO_2CH_3$) ppm.

^{13}C{^{1}H}-NMR (*cis* isomer) (101 MHz, $CDCl_3$) δ 173.3 (CO_2CH_3), 157.4 (q, *J* = 37.0 Hz, $NHCOCF_3$), 150.1 ($C_{Ar}O$), 149.9 ($C_{Ar}O$), 139.6 ($C_{Ar}C$), 138.3 ($C_{Ar}C$), 133.1 ($C_{Ar}N$), 132.7 ($C_{Ar}N$), 115.9 (q, *J* = 287.0 Hz, $NHCOCF_3$), 105.4 ($C_{Ar}H$), 104.2 ($C_{Ar}H$), 55.6 (OCH_3), 55.5 (OCH_3), 51.8 (CO_2CH_3), 40.8 ($C_{Ar}CH_2CH_2NHCOCF_3$), 35.7 ($C_{Ar}CH_2CH_2CO_2CH_3$), 35.3 ($C_{Ar}CH_2CH_2NHCOCF_3$), 31.5 ($C_{Ar}CH_2CH_2CO_2CH_3$) ppm.

^{19}F-NMR (376 MHz, $CDCl_3$) δ −75.9 ppm.

HRMS calcd for $C_{24}H_{29}N_3F_3O_7$ $[M+H]^+$: 528.1952, found 528.1950.

3. NMR Spectra

1-(4-Methylphenyl)-2-phenyldiazene (1b)

^{1}H-NMR (400 MHz, $CDCl_3$)

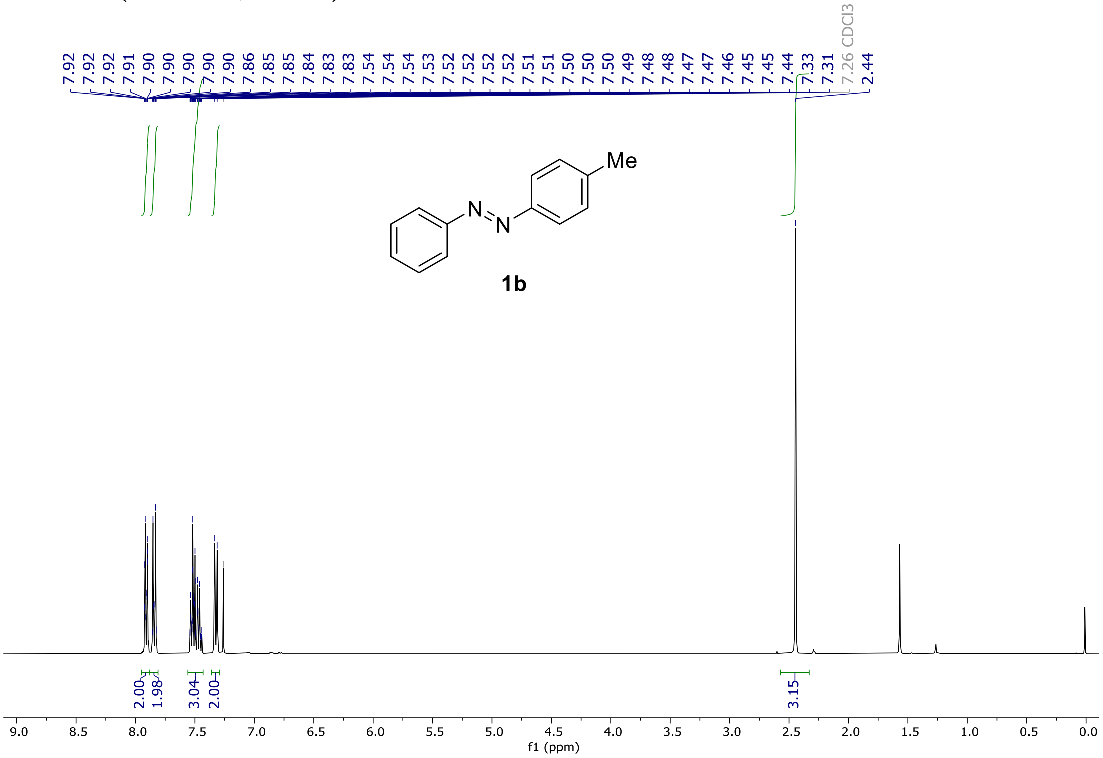

$^{13}C\{^{1}H\}$-NMR (100 MHz, $CDCl_3$)

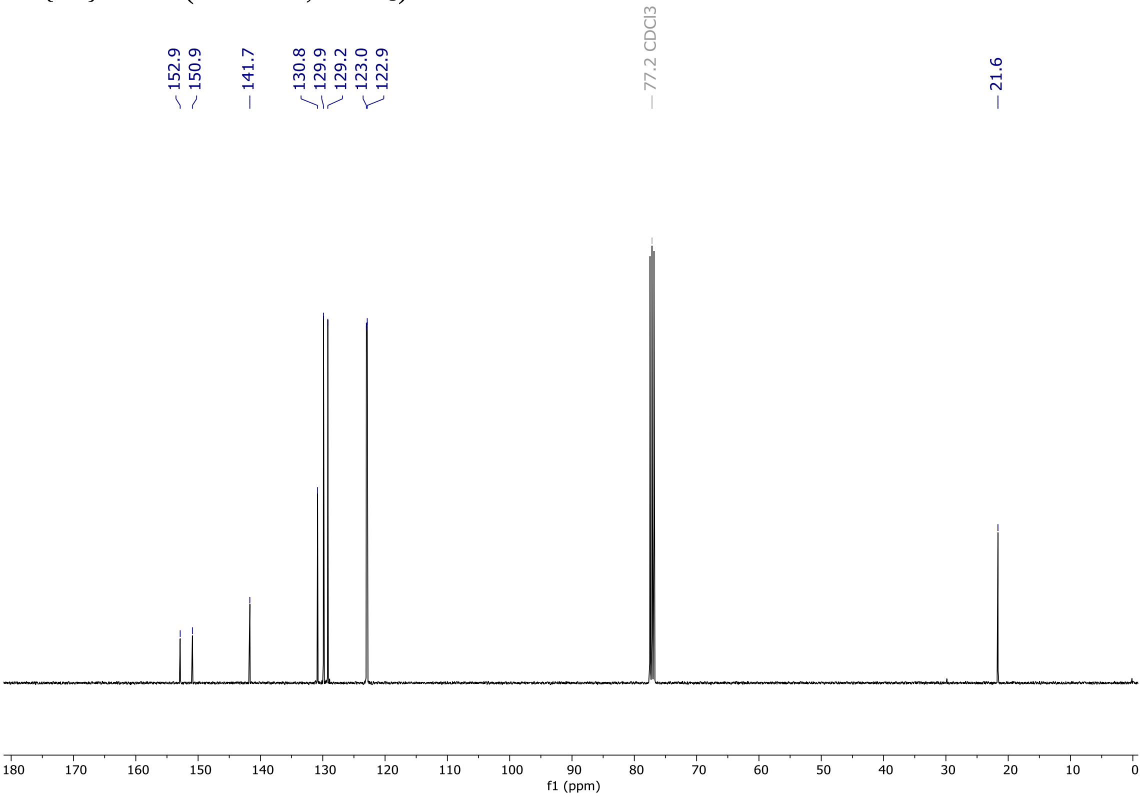

HSQC ($CDCl_3$)

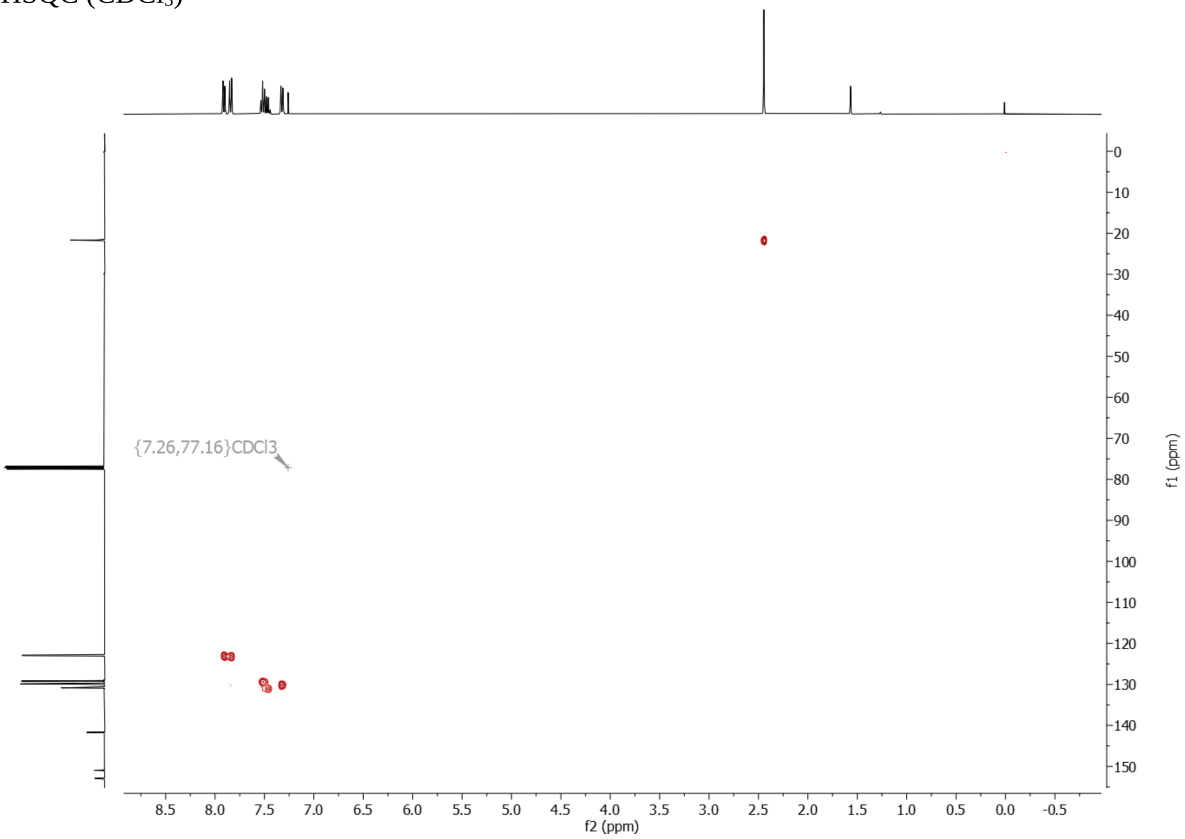

{7.26,77.16}CDCl3
0
10
20
30
40
50
60
70
80
90
100
110
120
130
140
150
f1 (ppm)
8.5
8.0
7.5
7.0
6.5
6.0
5.5
5.0
4.5
4.0
3.5
3.0
2.5
2.0
1.5
1.0
0.5
0.0
-0.5
f2 (ppm)

(*E*)-1,2-Bis(4-methylphenyl)diazene (1c)

^{1}H-NMR (400 MHz, $CDCl_3$)

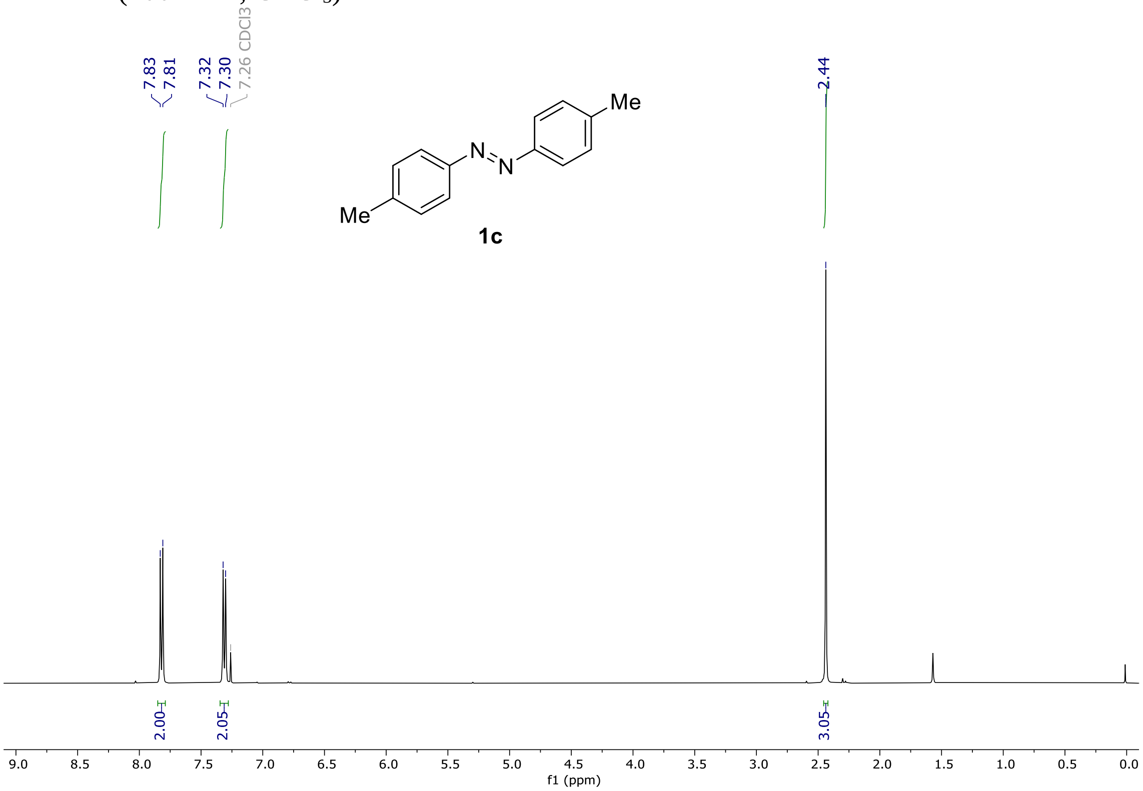

^{13}C{^{1}H}-NMR (100 MHz, $CDCl_3$)

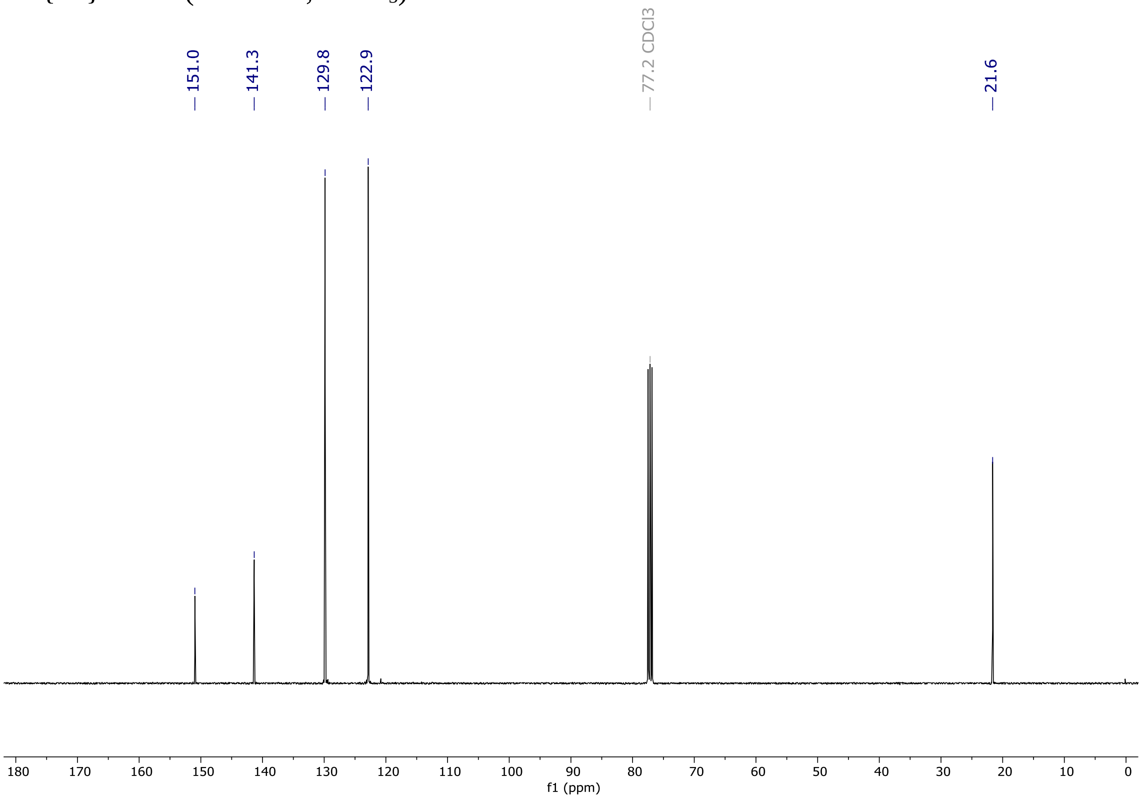

HSQC ($CDCl_3$)

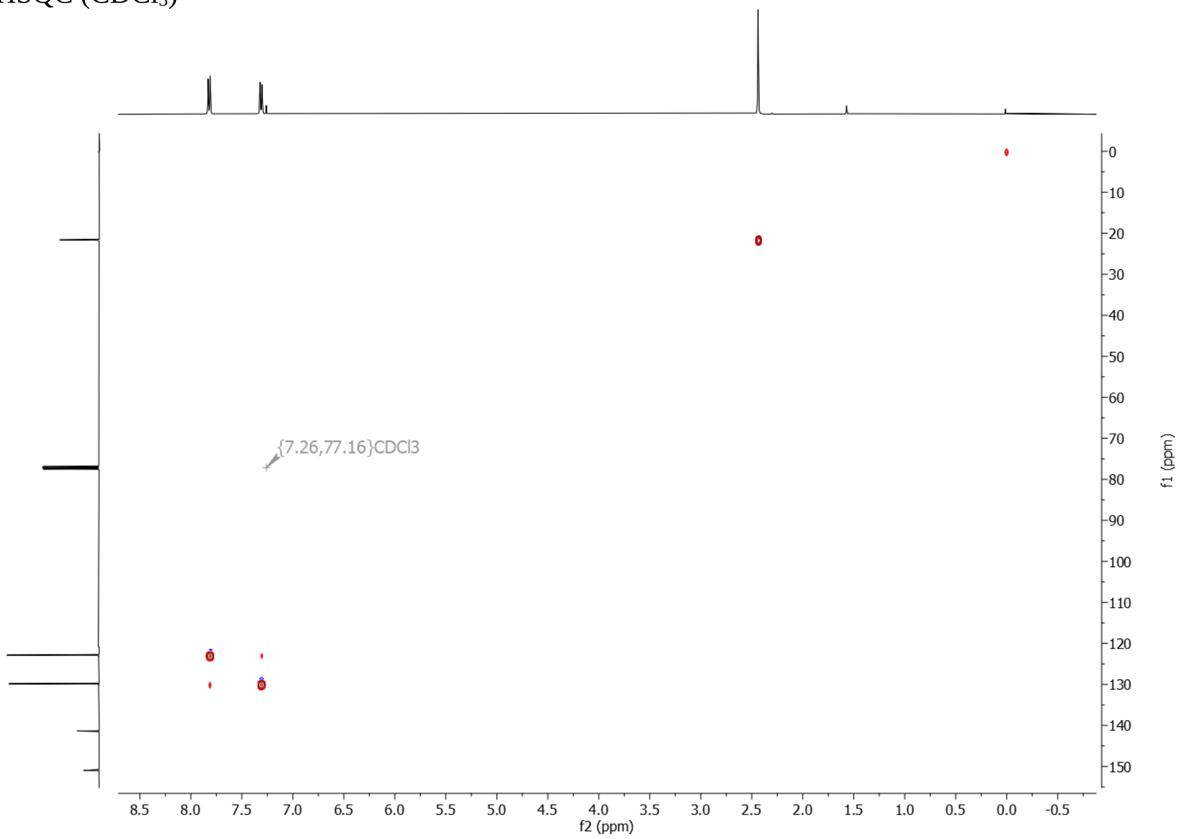

{7.26,77.16}CDCl3
8.5 8.0 7.5 7.0 6.5 6.0 5.5 5.0 4.5 4.0 3.5 3.0 2.5 2.0 1.5 1.0 0.5 0.0 -0.5
f2 (ppm)
0 10 20 30 40 50 60 70 80 90 100 110 120 130 140 150
f1 (ppm)

(*E*)-1,2-Bis(4-chlorophenyl)diazene (1e)

^{1}H-NMR (400 MHz, $CDCl_3$)

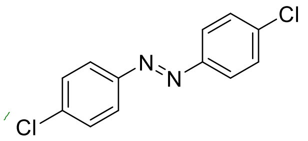

1e (*trans*/*cis*, 95:5)

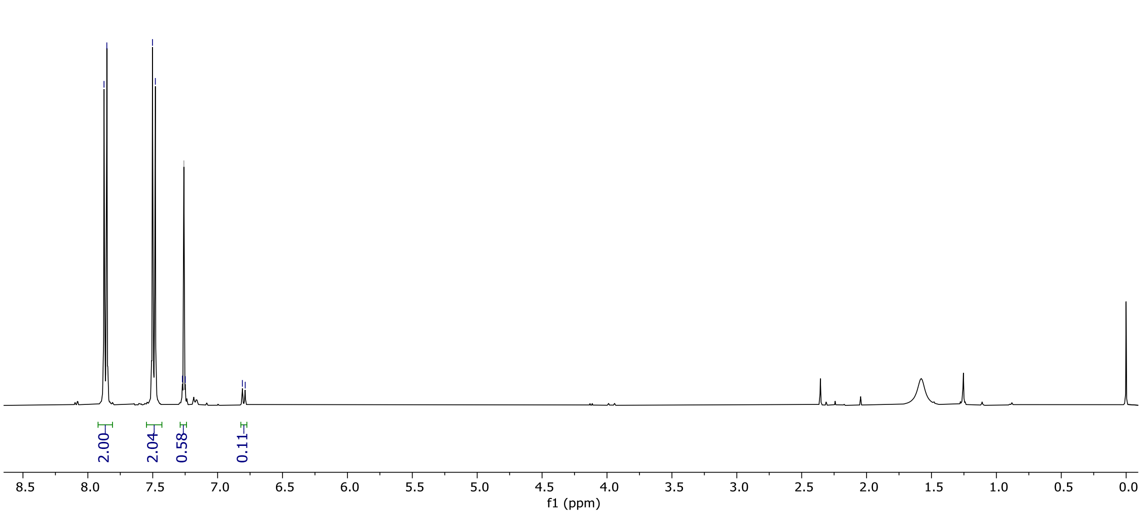

^{13}C{^{1}H}-NMR (100 MHz, $CDCl_3$)

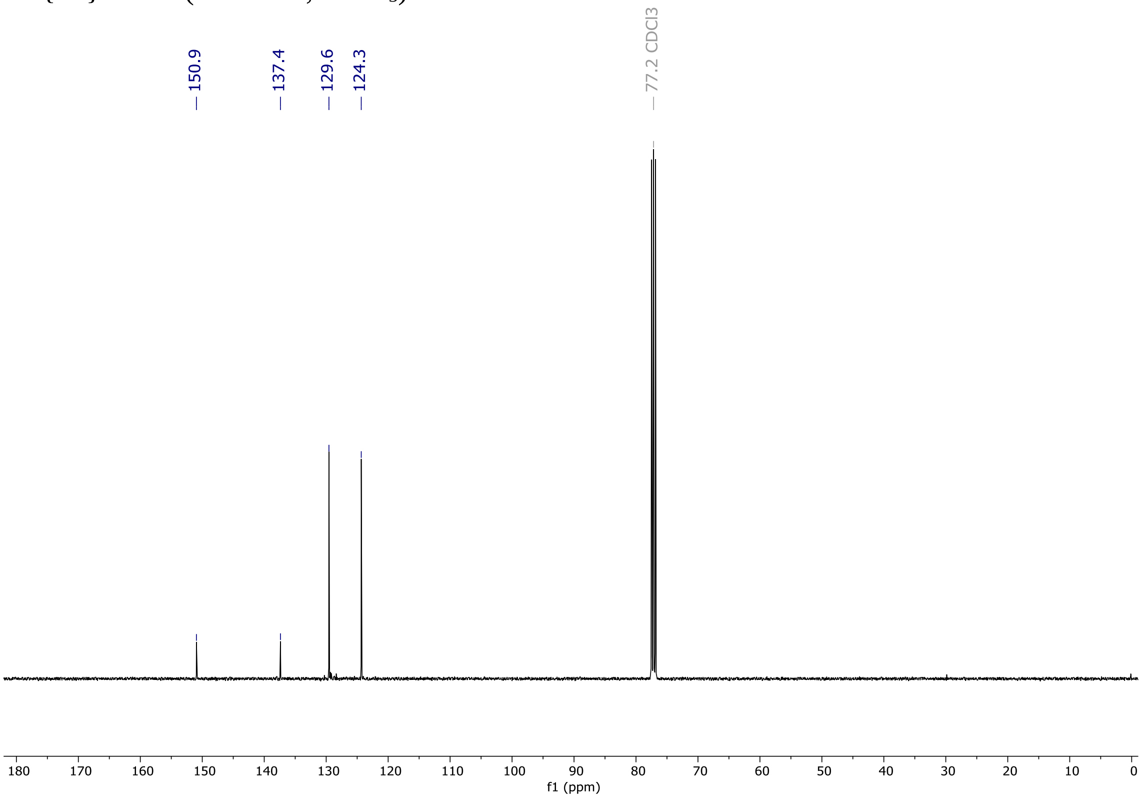

HSQC ($CDCl_3$)

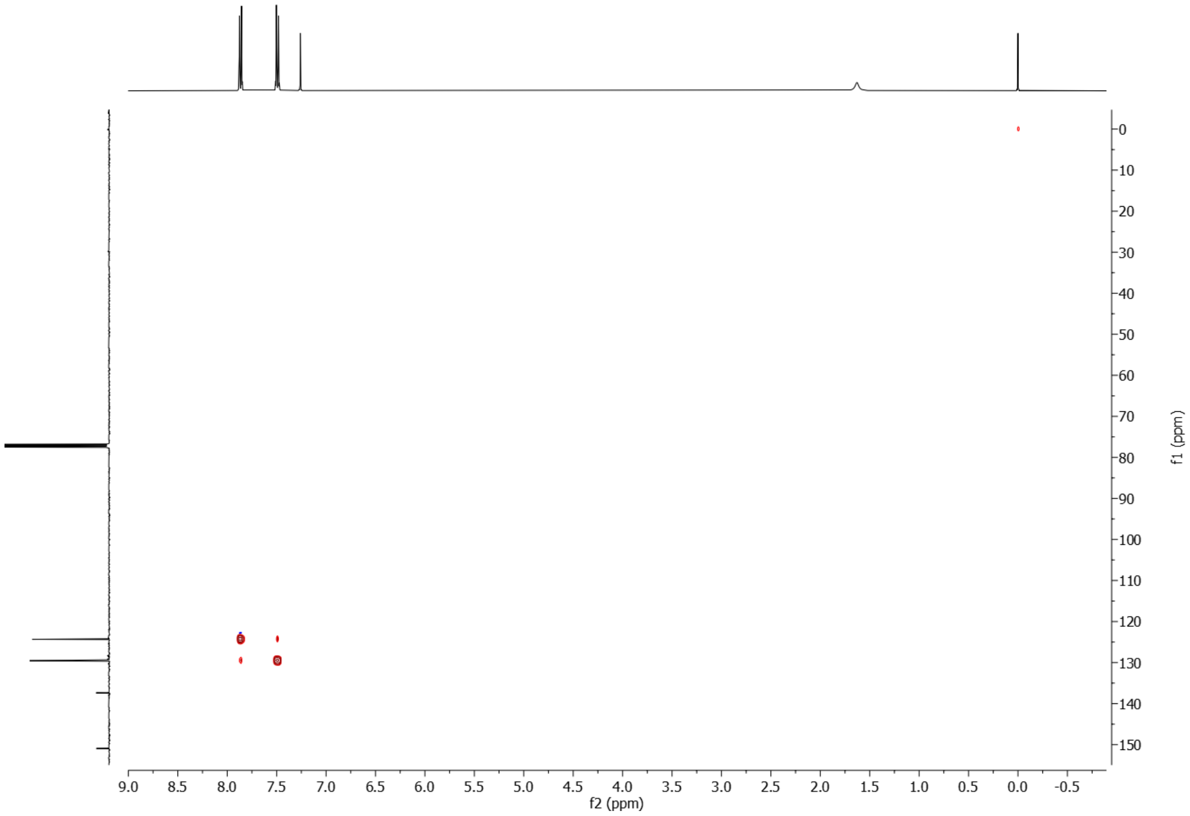

f1 (ppm)
f2 (ppm)
9.0
8.5
8.0
7.5
7.0
6.5
6.0
5.5
5.0
4.5
4.0
3.5
3.0
2.5
2.0
1.5
1.0
0.5
0.0
-0.5
0
10
20
30
40
50
60
70
80
90
100
110
120
130
140
150

(*E*)-1,2-Bis(4-bromophenyl)diazene (1f)

^{1}H-NMR (400 MHz, $CDCl_3$)

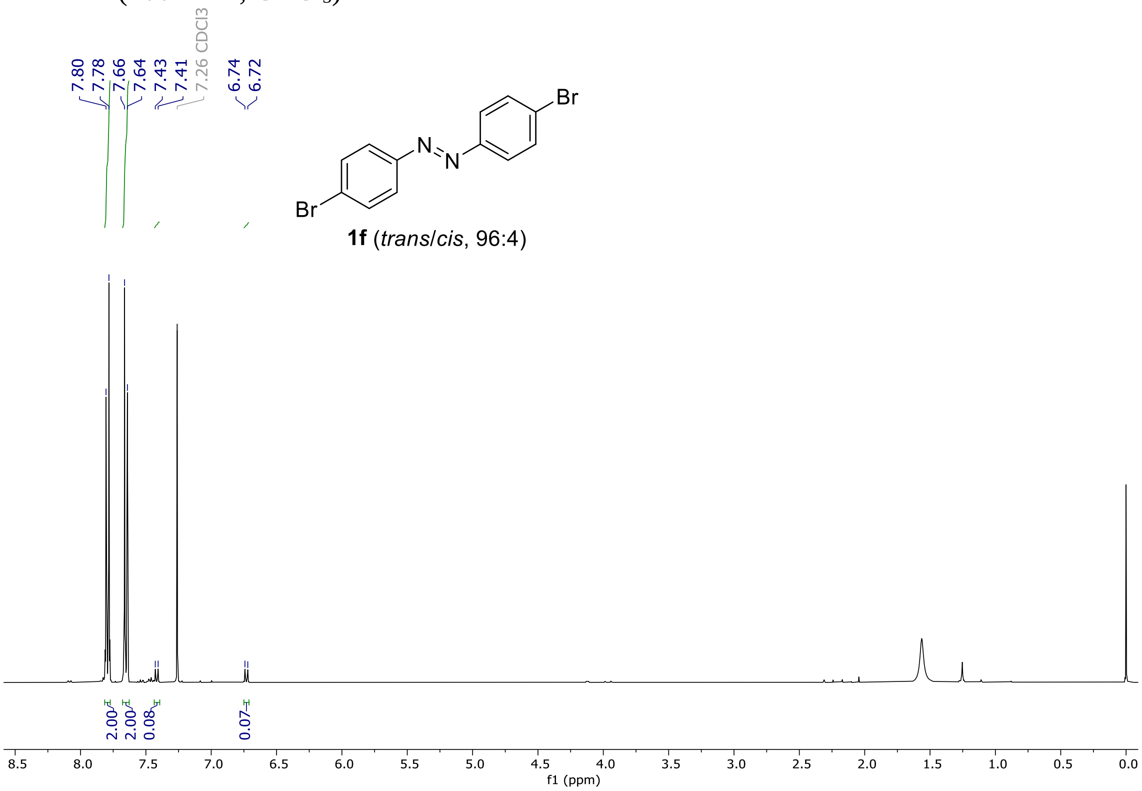

^{13}C{^{1}H}-NMR (100 MHz, $CDCl_3$)

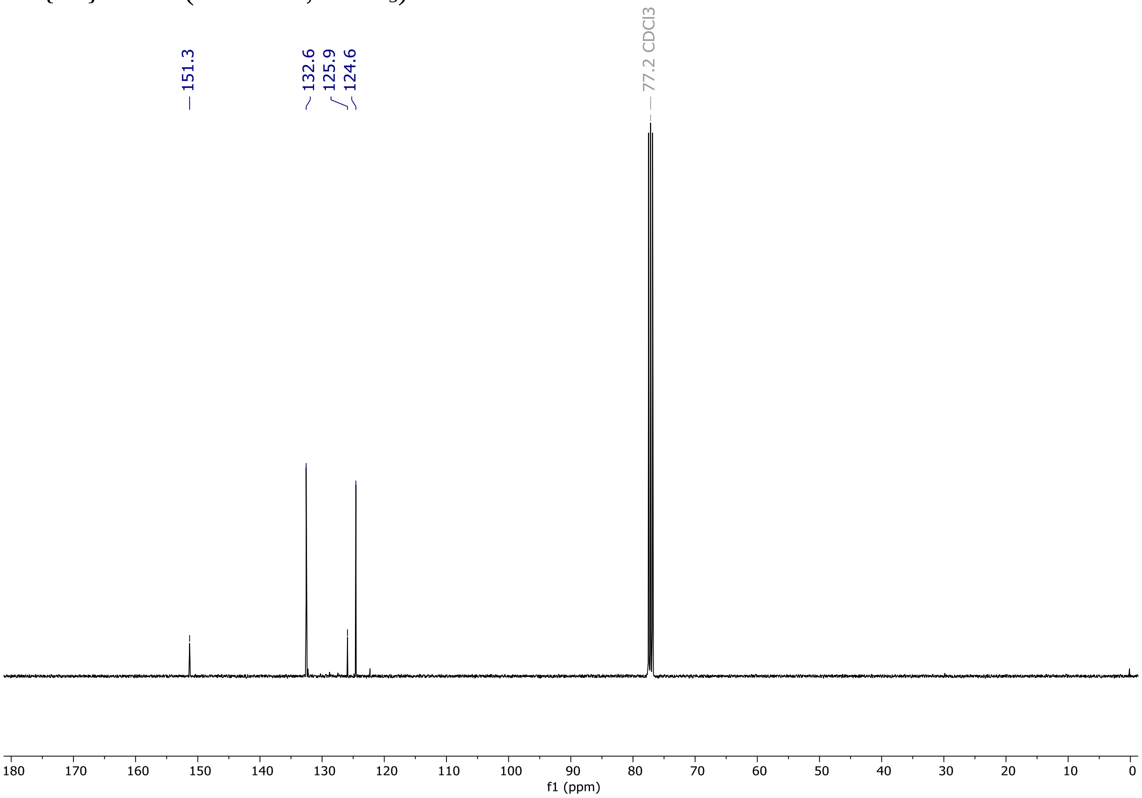

HSQC ($CDCl_3$)

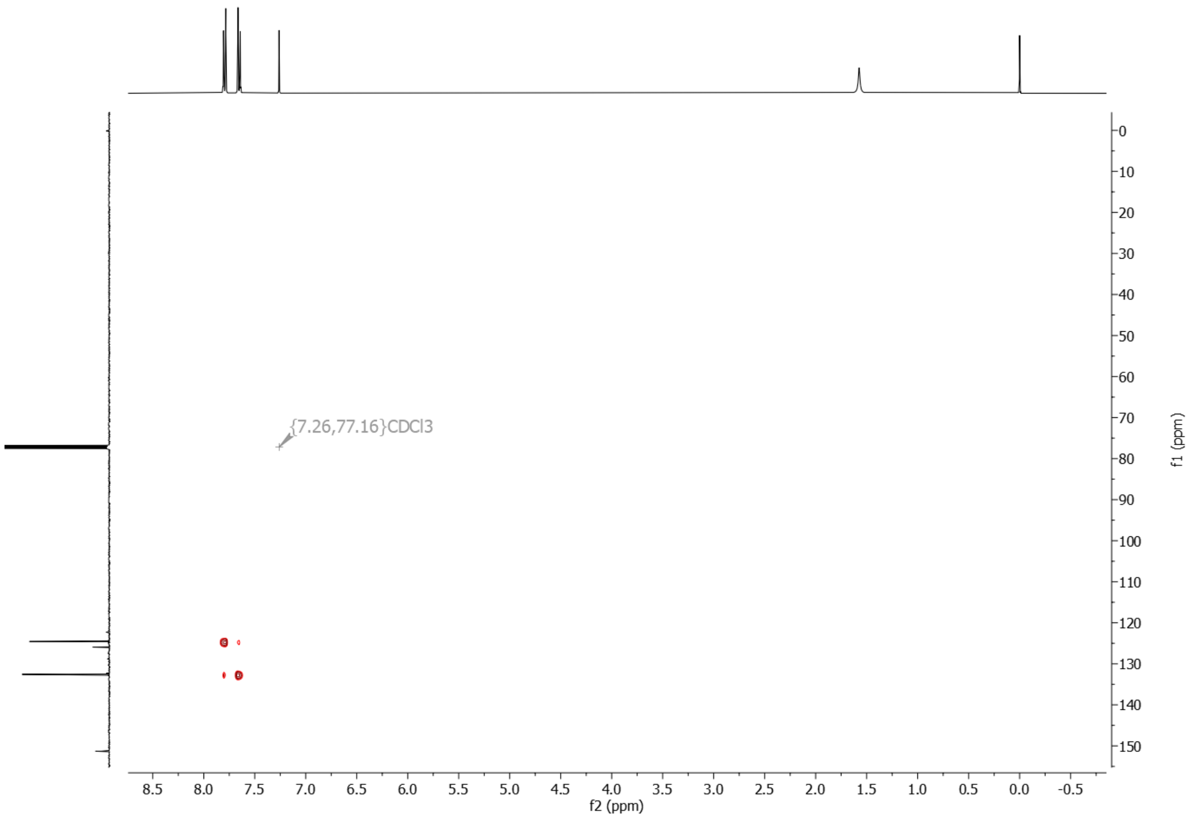

{7.26,77.16}CDCl3
0
10
20
30
40
50
60
70
80
90
100
110
120
130
140
150
f1 (ppm)
8.5
8.0
7.5
7.0
6.5
6.0
5.5
5.0
4.5
4.0
3.5
3.0
2.5
2.0
1.5
1.0
0.5
0.0
-0.5
f2 (ppm)

(*E*)-1,2-Bis(4-iodophenyl)diazene (1g)

^{1}H-NMR (400 MHz, $CDCl_3$)

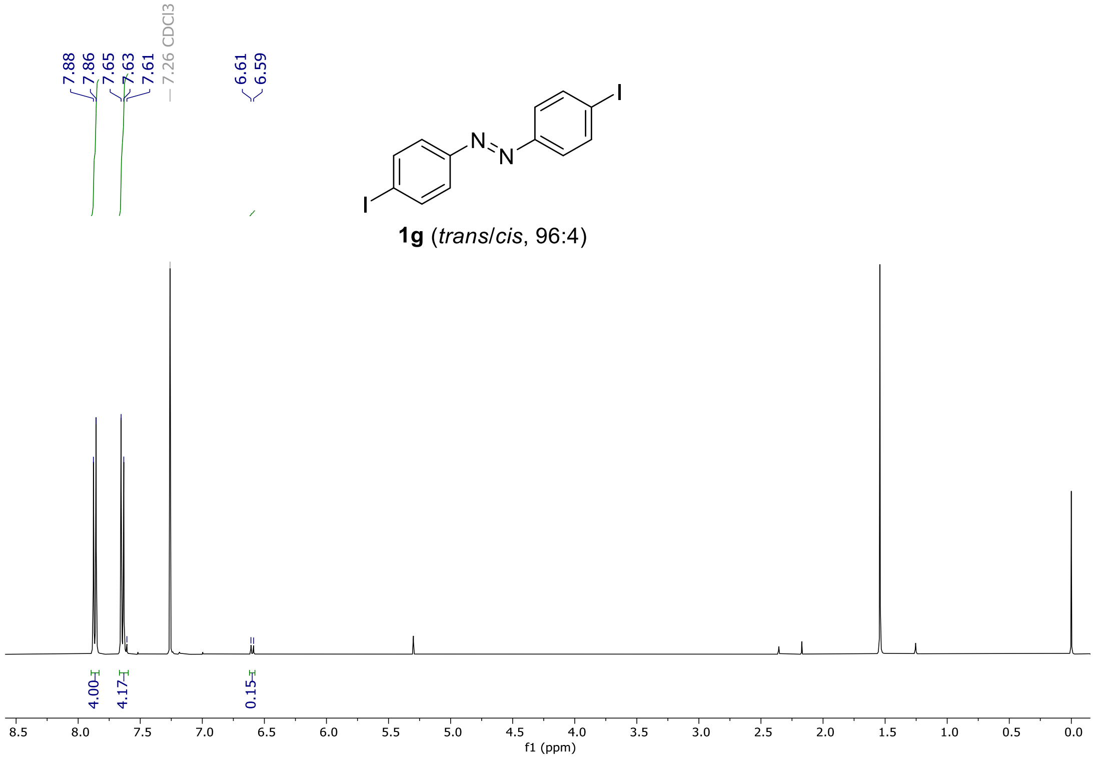

^{13}C{^{1}H}-NMR (100 MHz, $CDCl_3$)

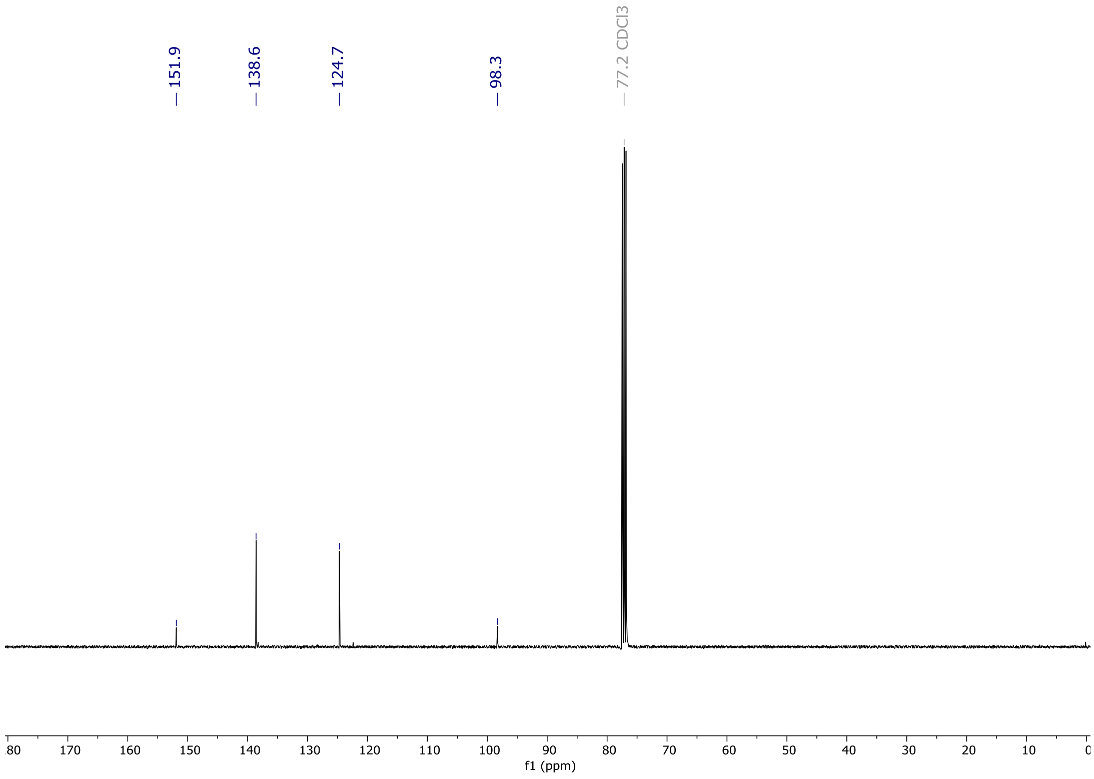

HSQC ($CDCl_3$)

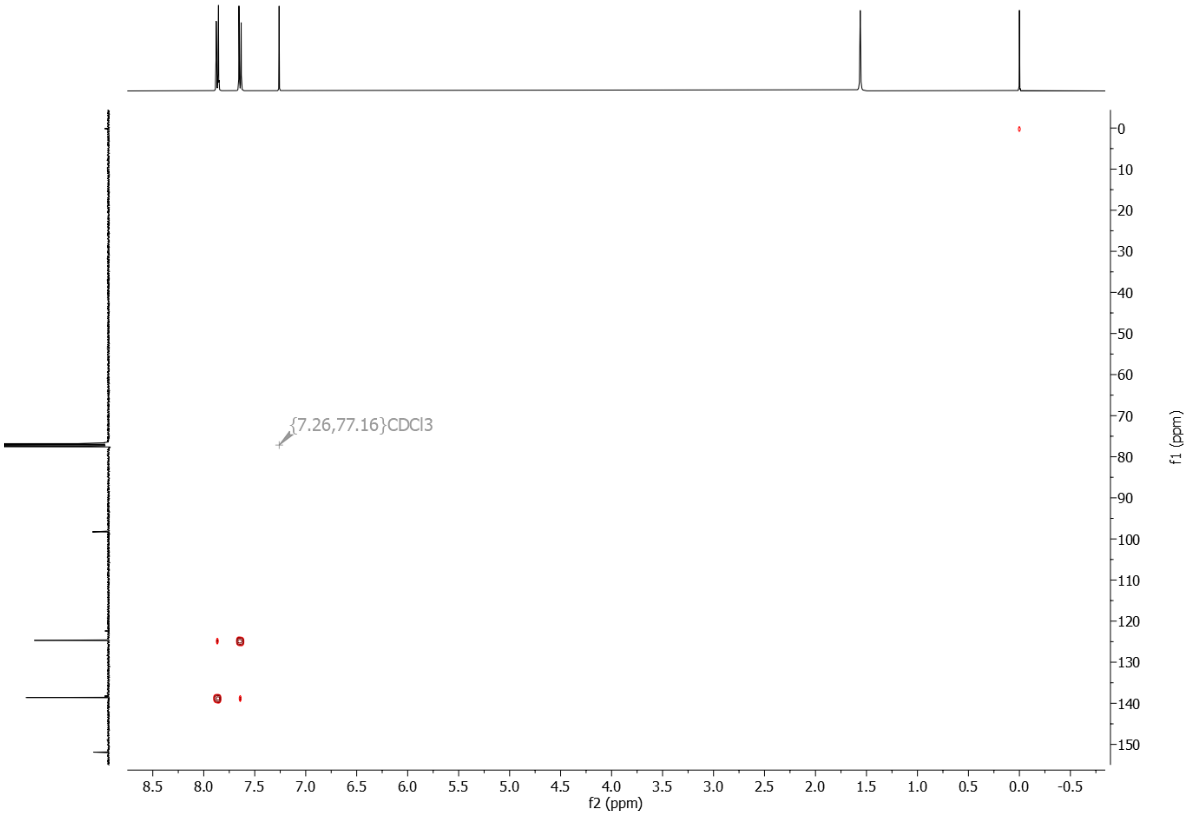
{7.26,77.16}CDCl3
8.5 8.0 7.5 7.0 6.5 6.0 5.5 5.0 4.5 4.0 3.5 3.0 2.5 2.0 1.5 1.0 0.5 0.0 -0.5
f2 (ppm)
0 10 20 30 40 50 60 70 80 90 100 110 120 130 140 150
f1 (ppm)

(*E*)-1,2-Bis(4-(trifluoromethyl)phenyl)diazene (1h)

^{1}H-NMR (400 MHz, $CDCl_3$)

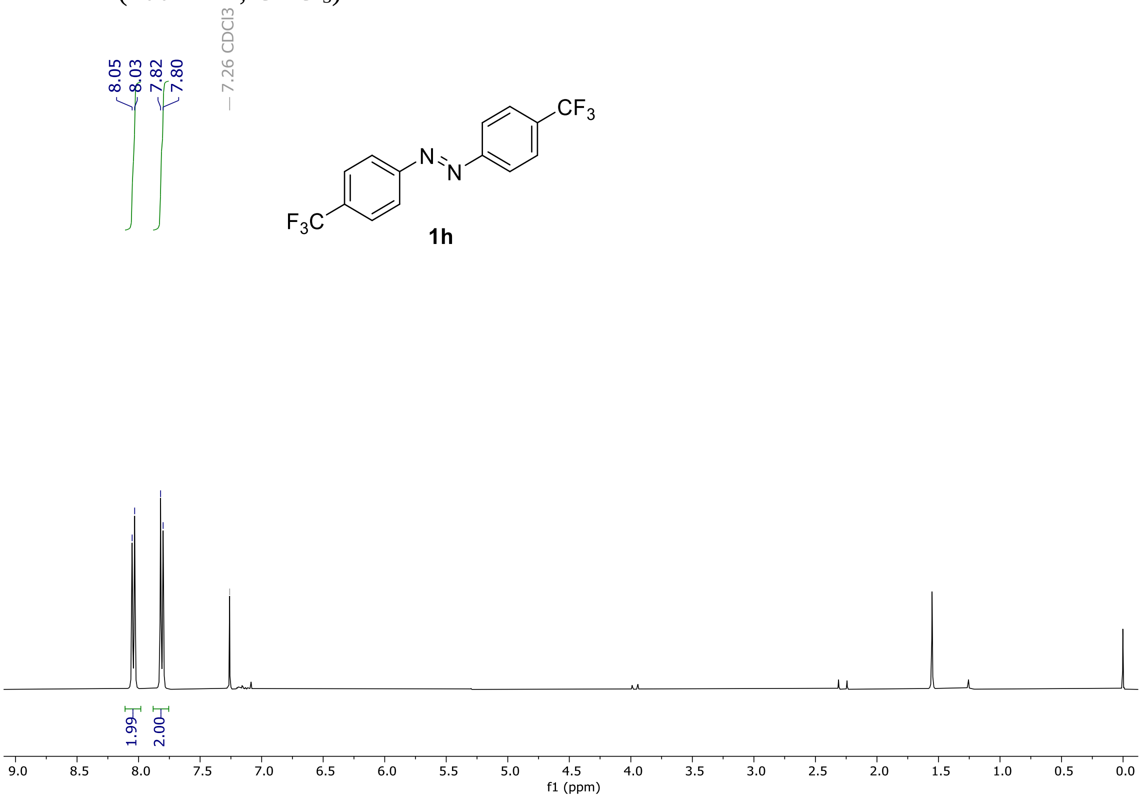

$^{13}C\{^{1}H\}$-NMR (100 MHz, $CDCl_3$)

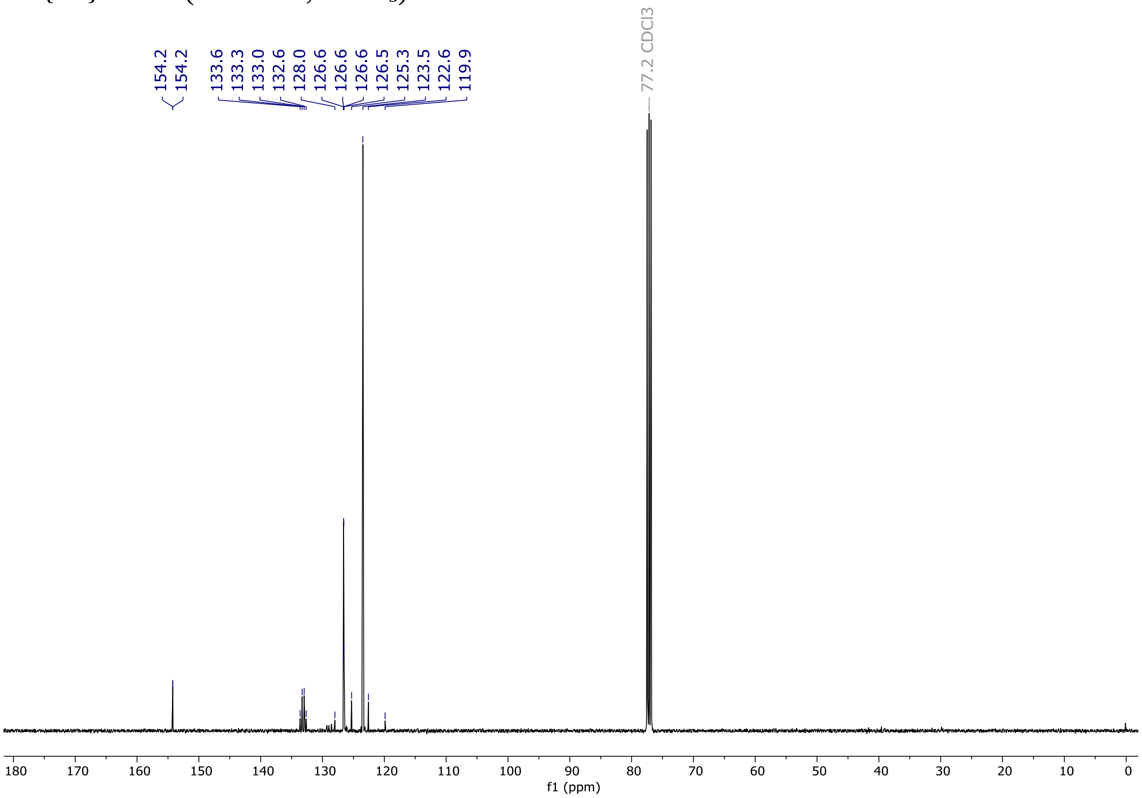

^{19}F-NMR (376 MHz, $CDCl_3$)

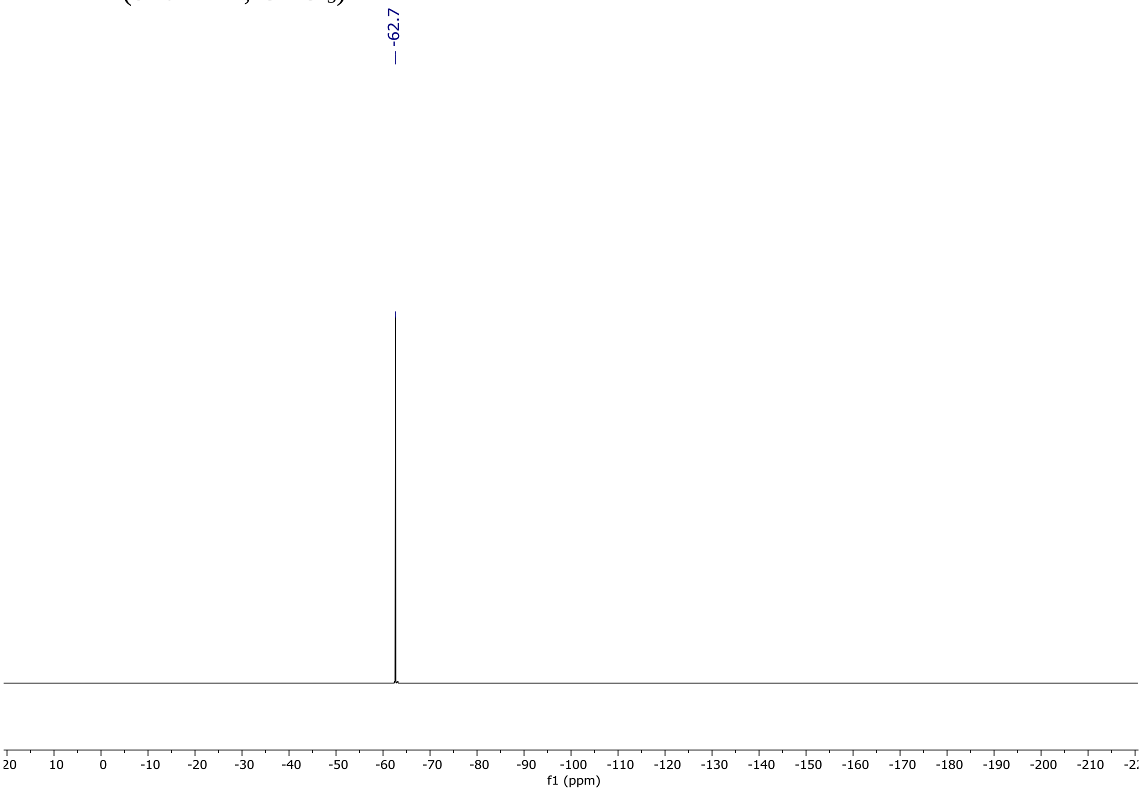

HSQC ($CDCl_3$)

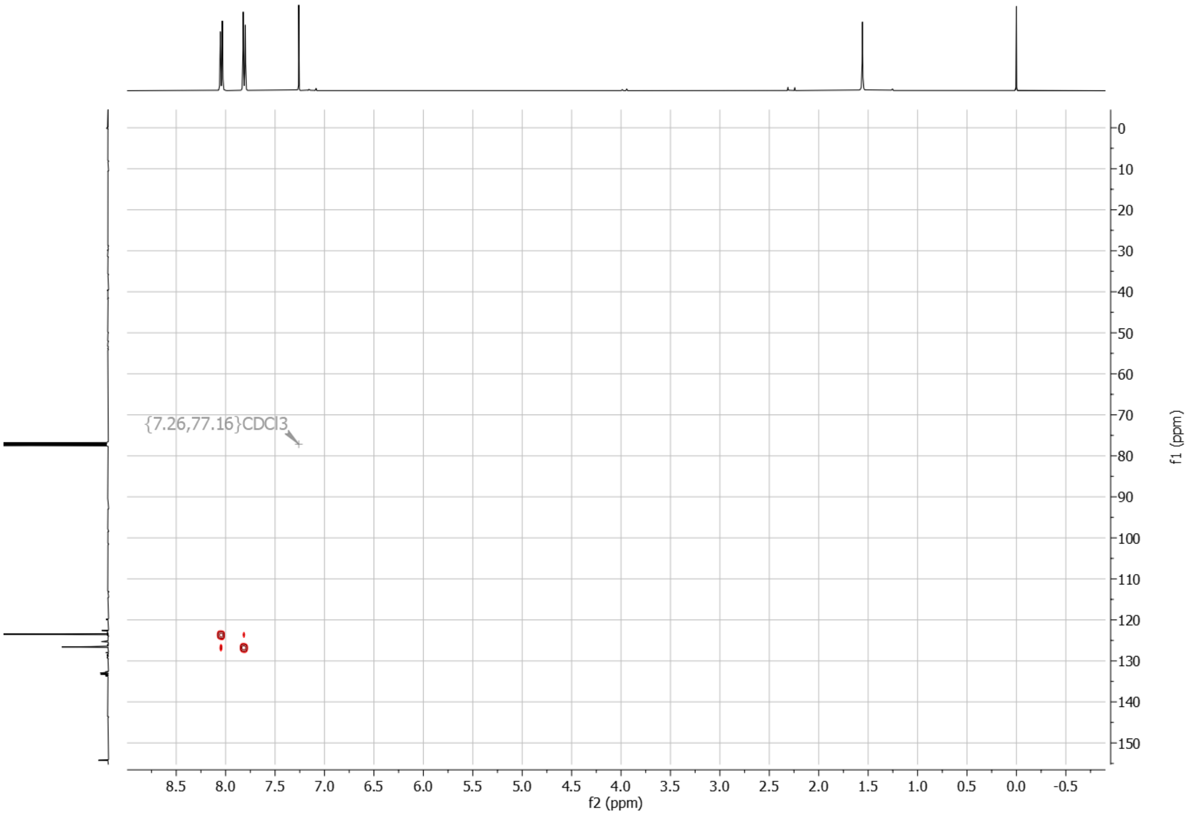

(*E*)-3-(4-(Phenyldiazenyl)phenyl)propionic acid (1i)

^{1}H-NMR (400 MHz, $CDCl_3$)

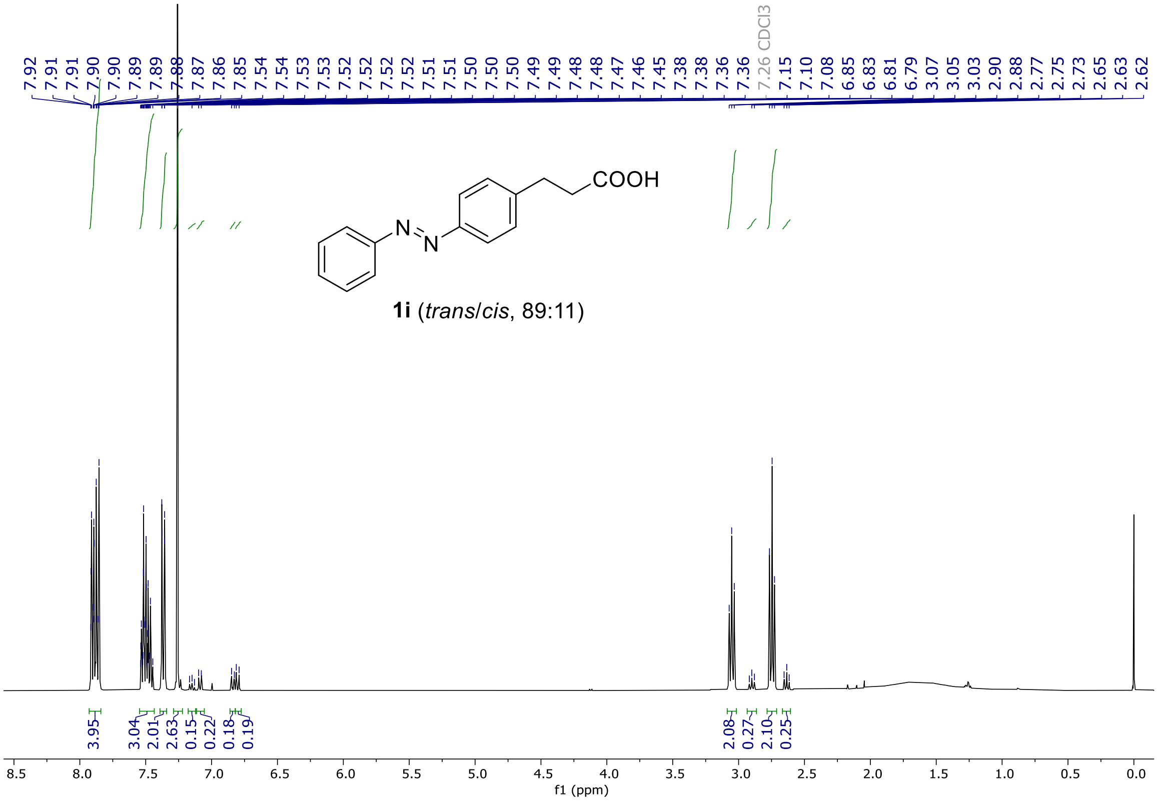

$^{13}C\{^{1}H\}$-NMR (100 MHz, $CDCl_3$)

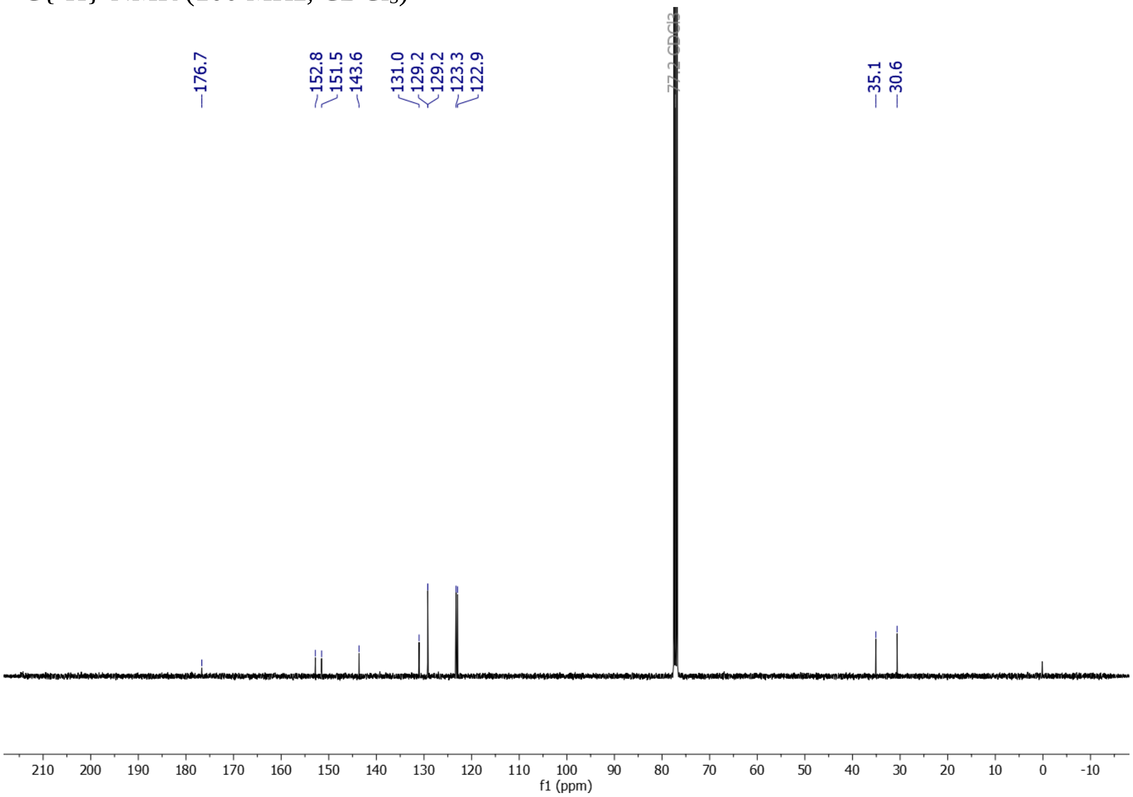

HSQC ($CDCl_3$)

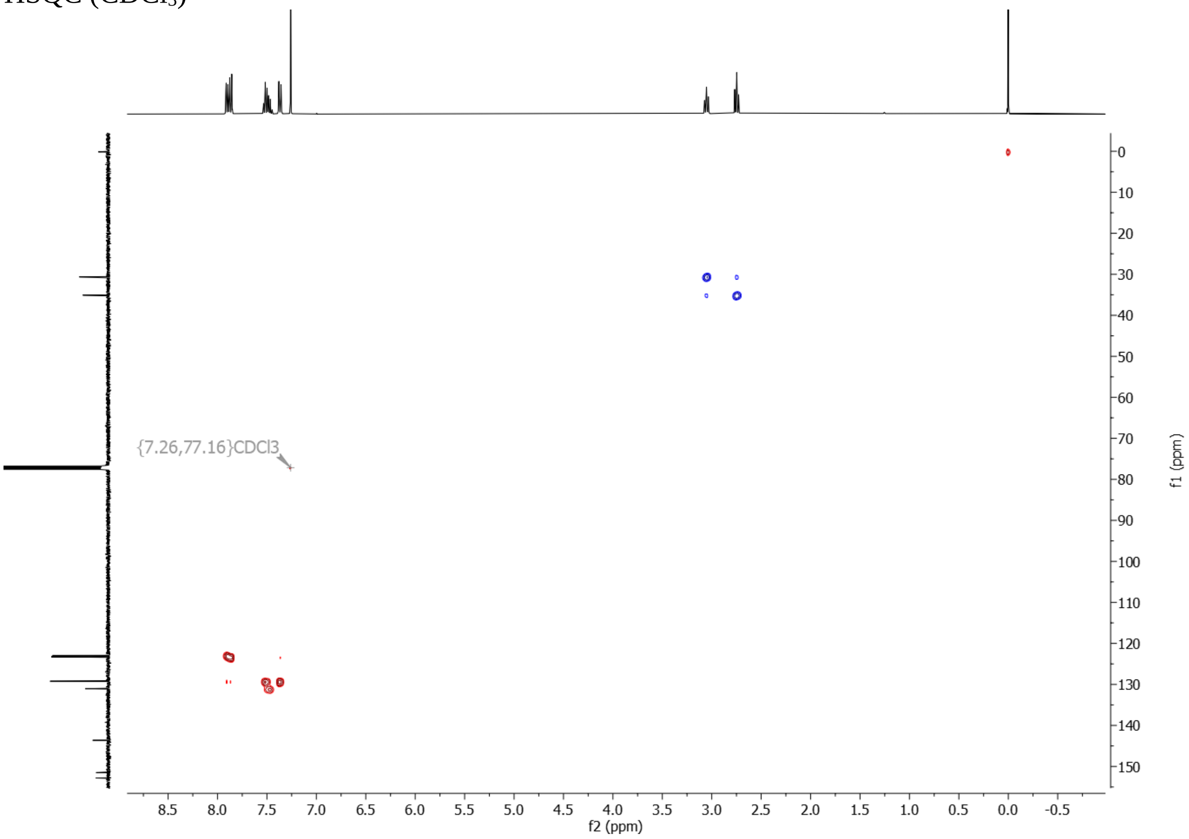

{7.26,77.16}CDCl3
f2 (ppm)
f1 (ppm)
8.5 8.0 7.5 7.0 6.5 6.0 5.5 5.0 4.5 4.0 3.5 3.0 2.5 2.0 1.5 1.0 0.5 0.0 -0.5
0 10 20 30 40 50 60 70 80 90 100 110 120 130 140 150

Dimethyl 2,2'-(diazene-1,2-diylbis(4,1-phenylene))(*E*)-dipropionate (1k)

^{1}H-NMR (400 MHz, $CDCl_3$)

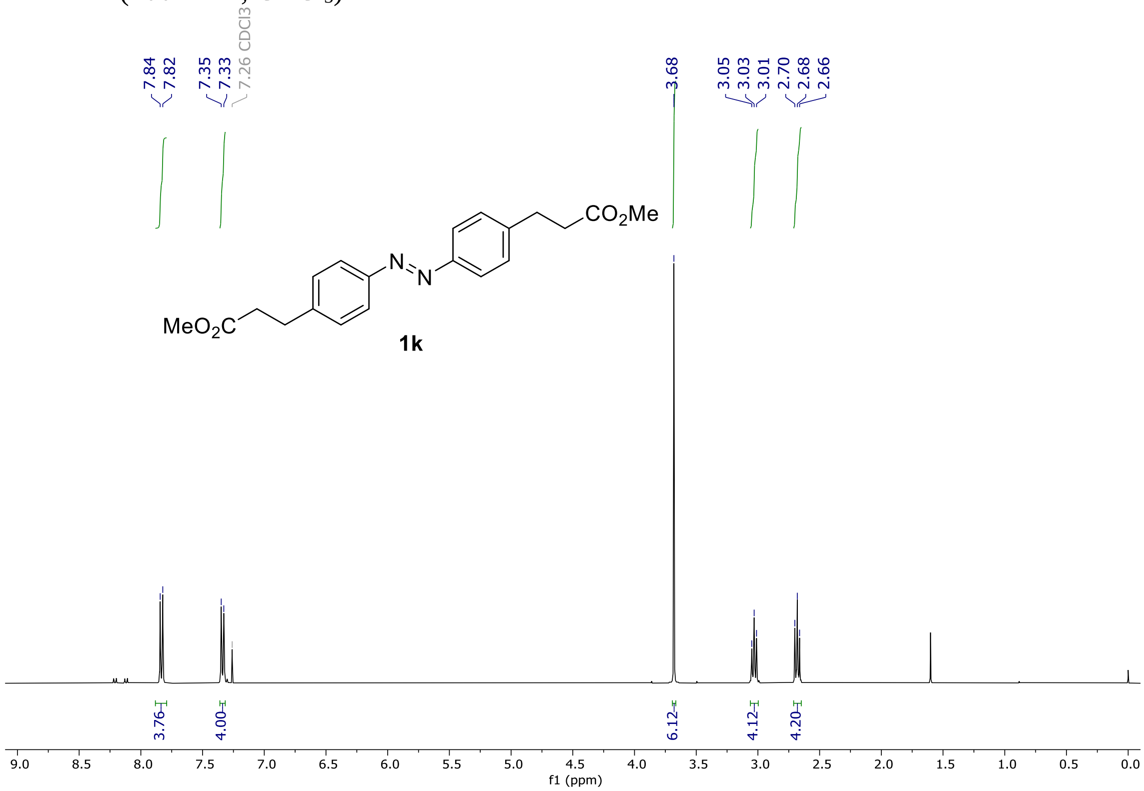

^{13}C{^{1}H}-NMR (100 MHz, $CDCl_3$)

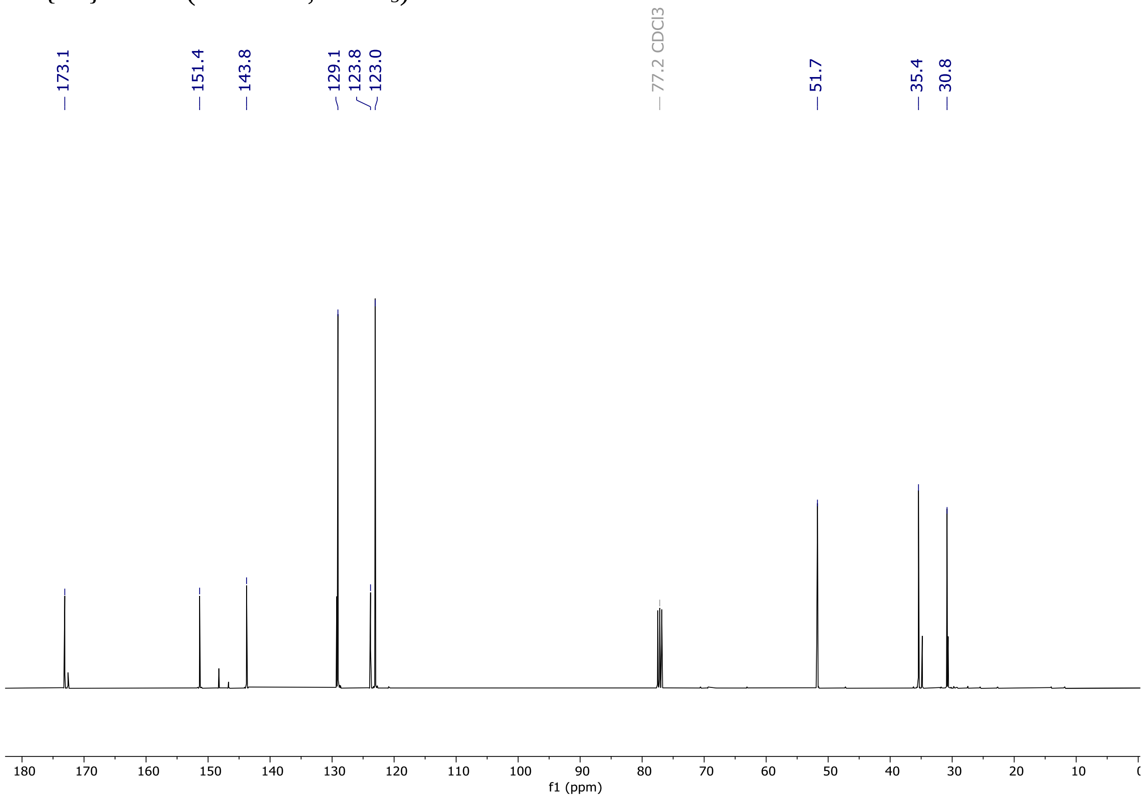

HSQC ($CDCl_3$)

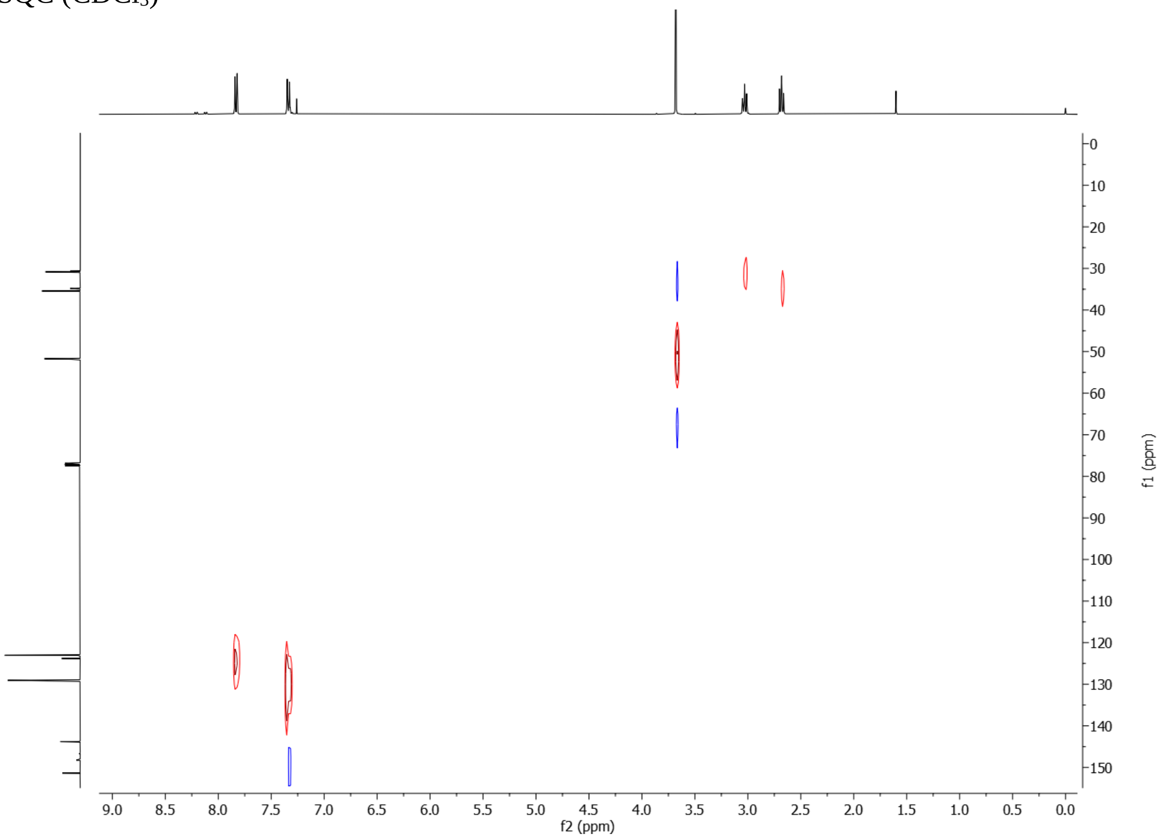

9.0
8.5
8.0
7.5
7.0
6.5
6.0
5.5
5.0
4.5
4.0
3.5
3.0
2.5
2.0
1.5
1.0
0.5
0.0
f2 (ppm)
0
10
20
30
40
50
60
70
80
90
100
110
120
130
140
150
f1 (ppm)

Methyl (*E*)-3-(4-(phenyldiazenyl)phenyl)propanoate (1l)

^{1}H-NMR (400 MHz, $CDCl_3$)

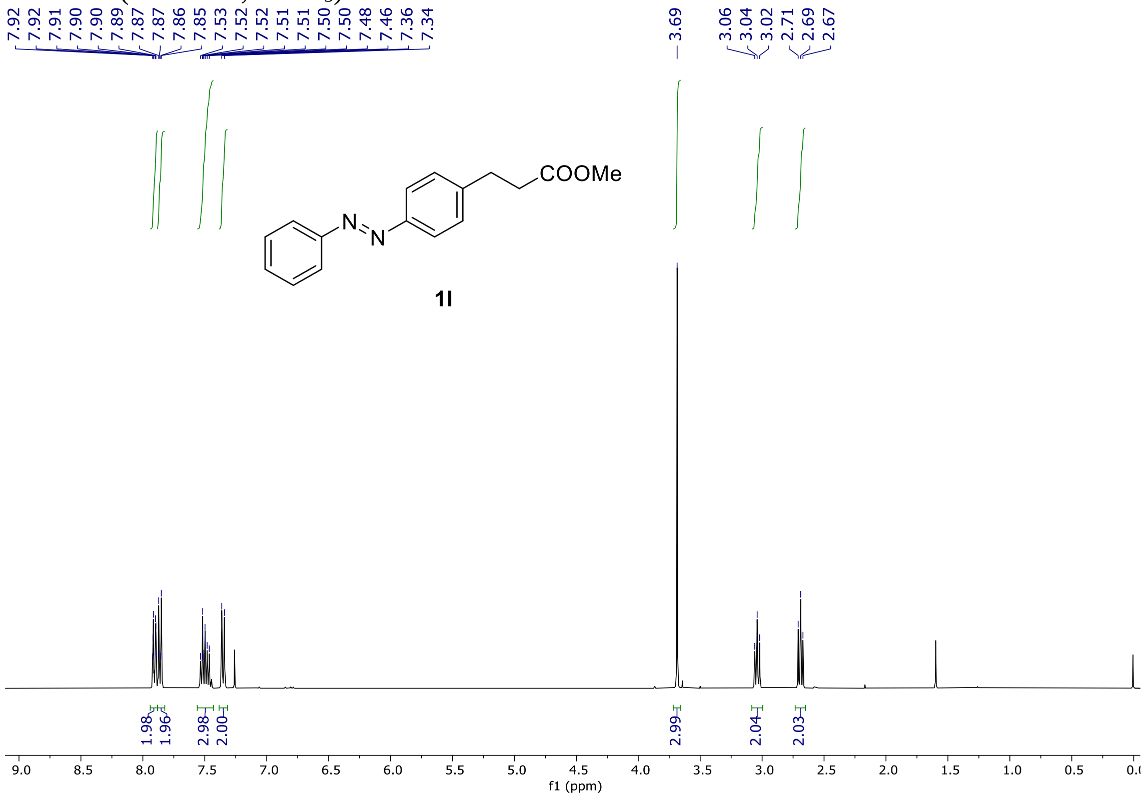

^{13}C{^{1}H}-NMR (100 MHz, $CDCl_3$)

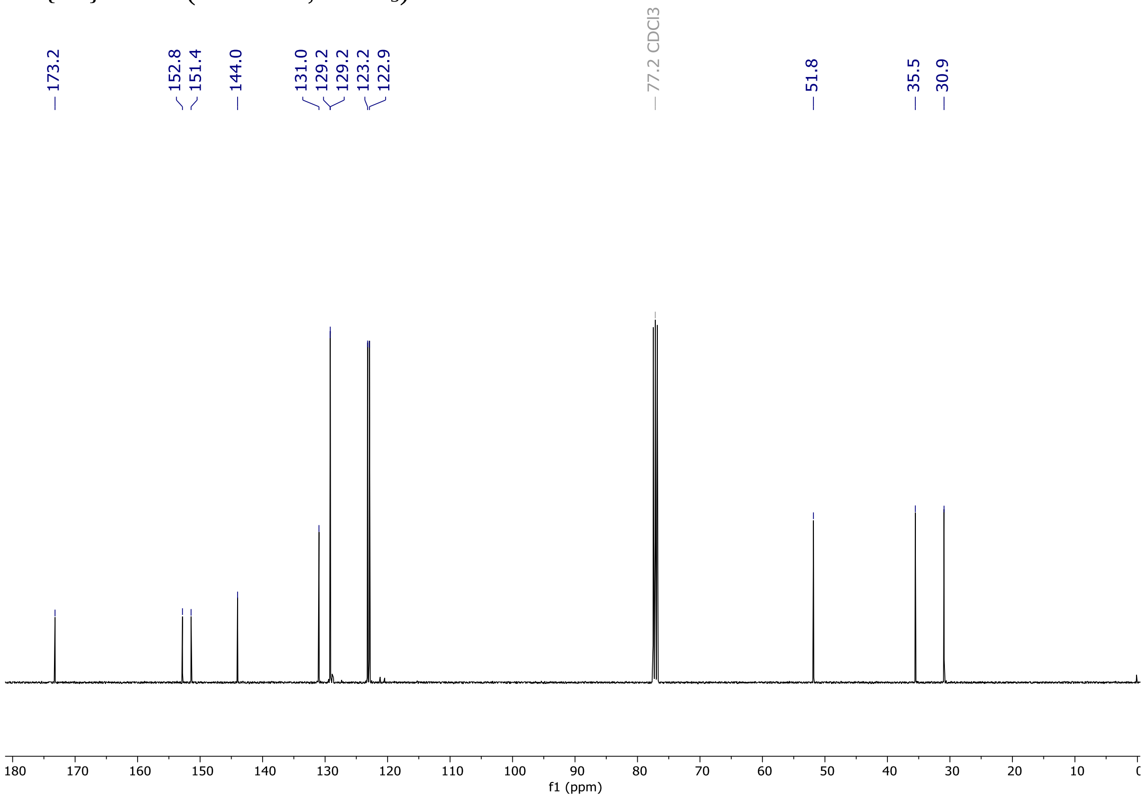

HSQC ($CDCl_3$)

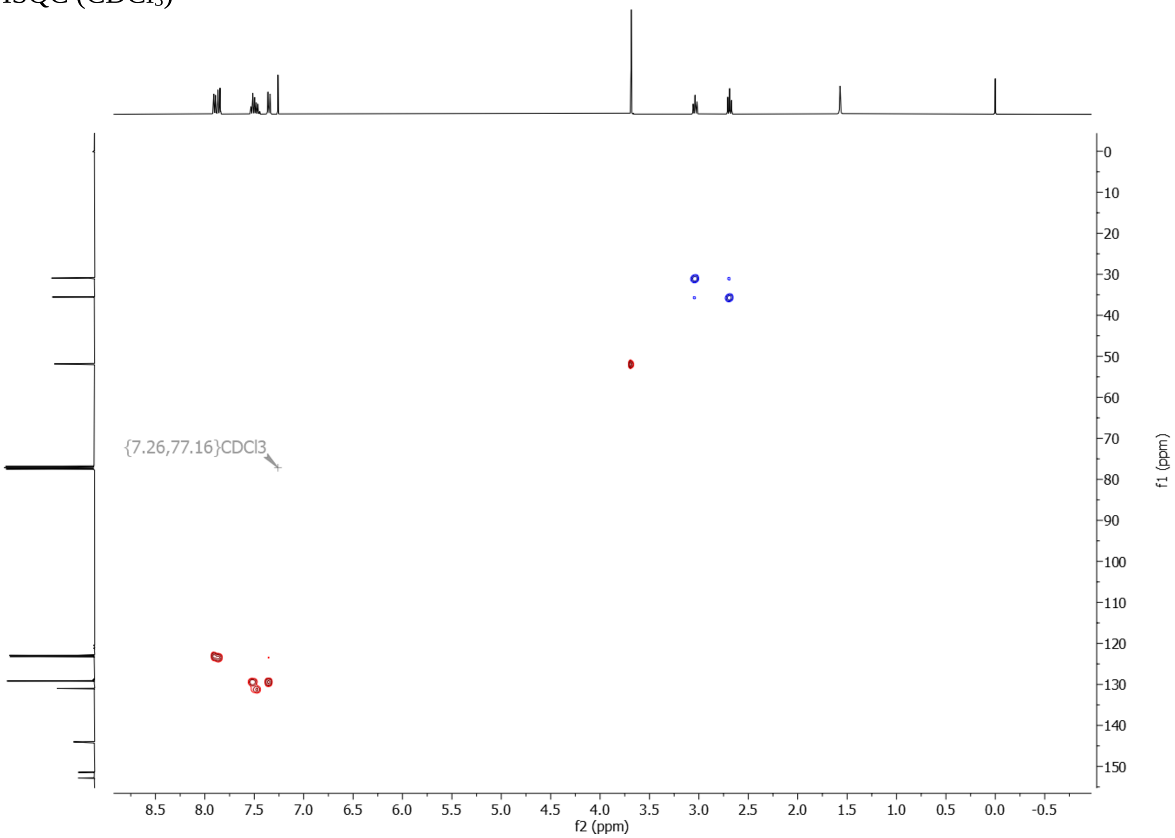

Methyl (*E*)-4-(phenyldiazenyl)benzoate (1m)

^{1}H-NMR (400 MHz, $CDCl_3$)

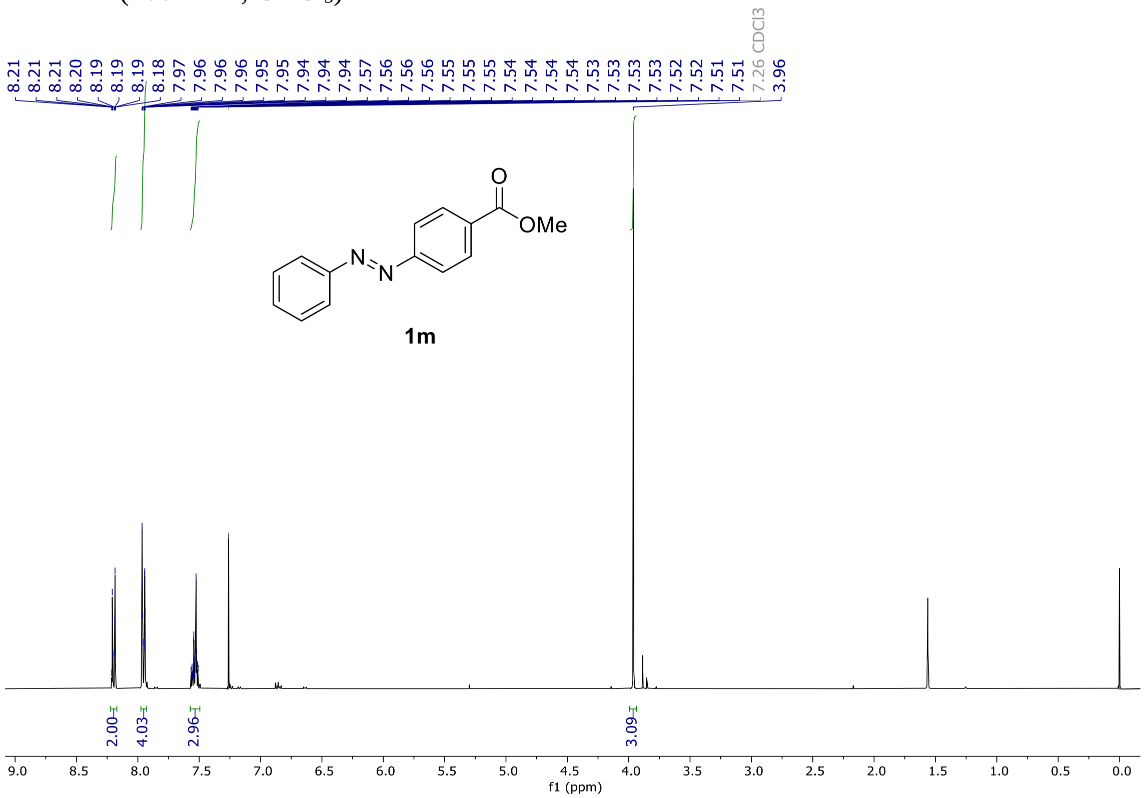

^{13}C{^{1}H}-NMR (100 MHz, $CDCl_3$)

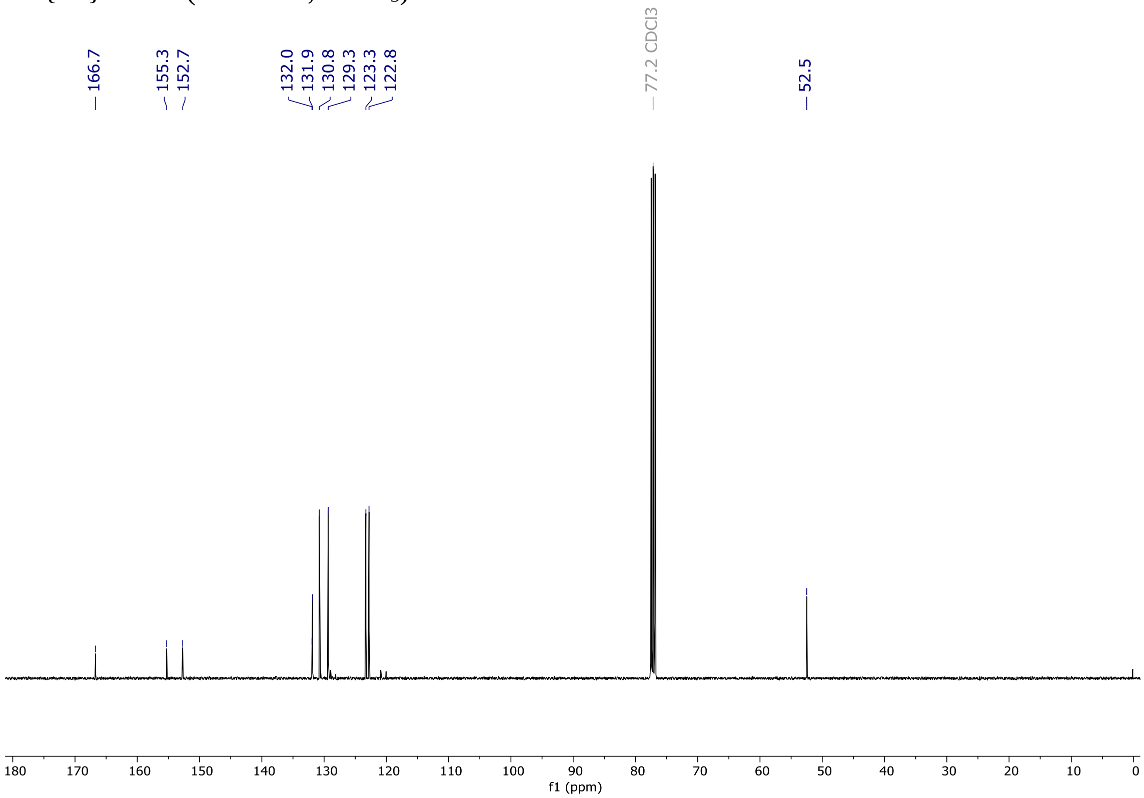

HSQC ($CDCl_3$)

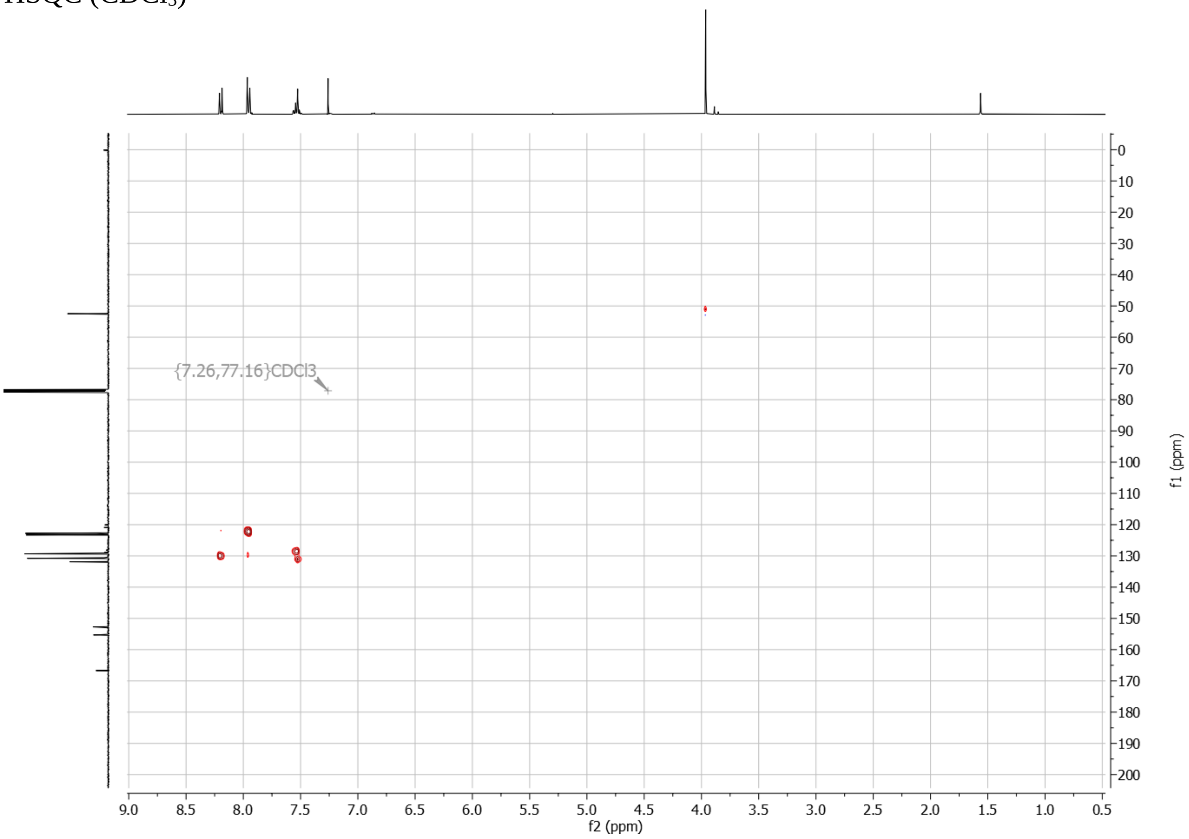

(*E*)-2,2,2-Trifluoro-*N*-(4-(phenyldiazenyl)phenethyl)acetamide (1n)

^{1}H-NMR (400 MHz, $CDCl_3$)

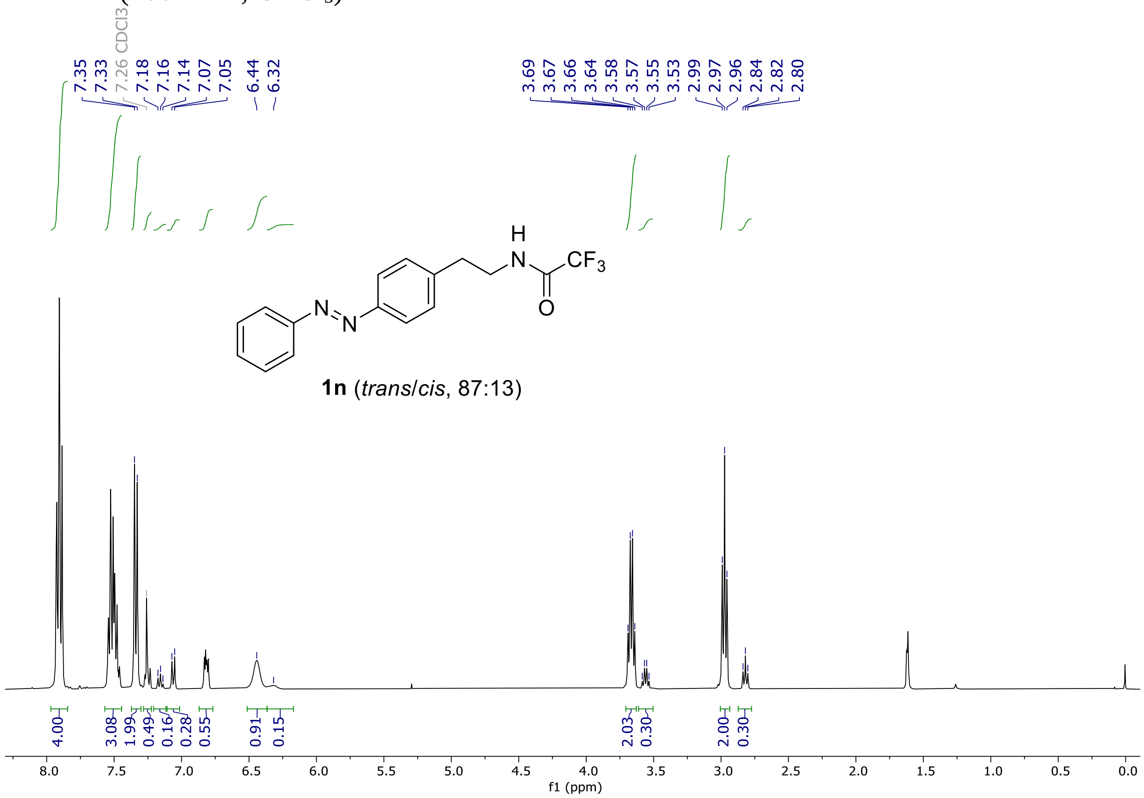

^{13}C{^{1}H}-NMR (100 MHz, $CDCl_3$)

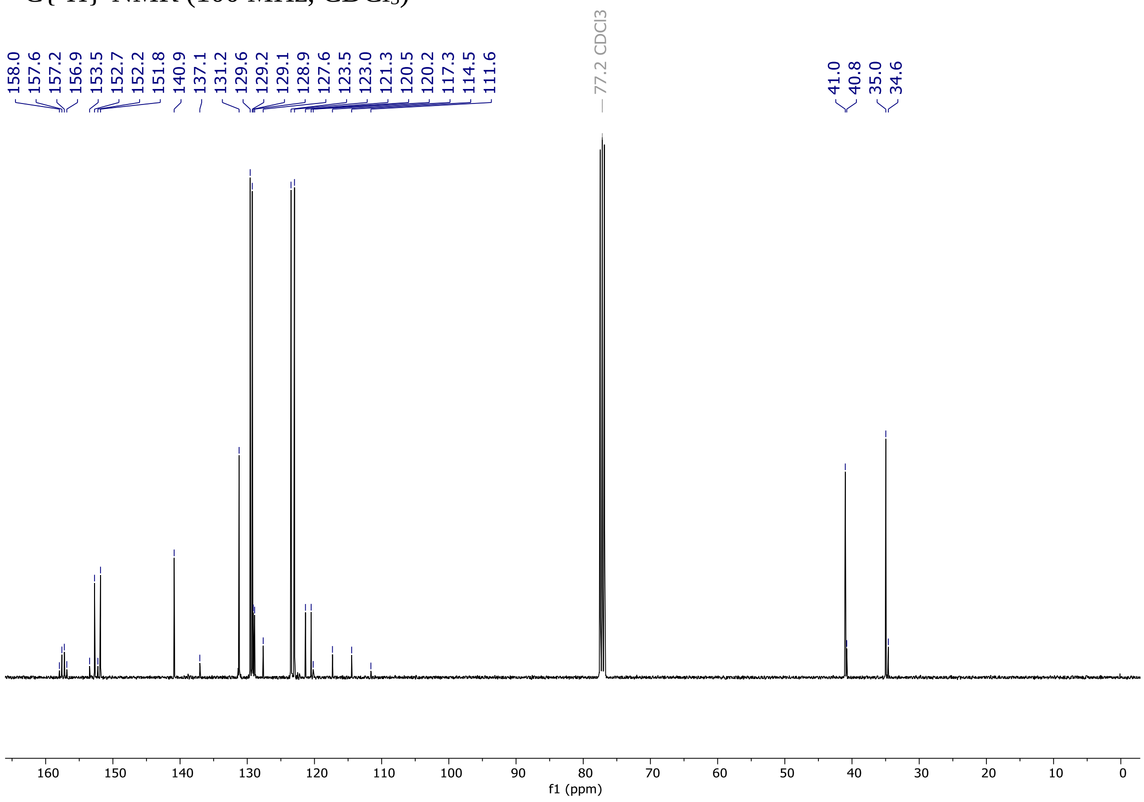

^{19}F-NMR (376 MHz, $CDCl_3$)

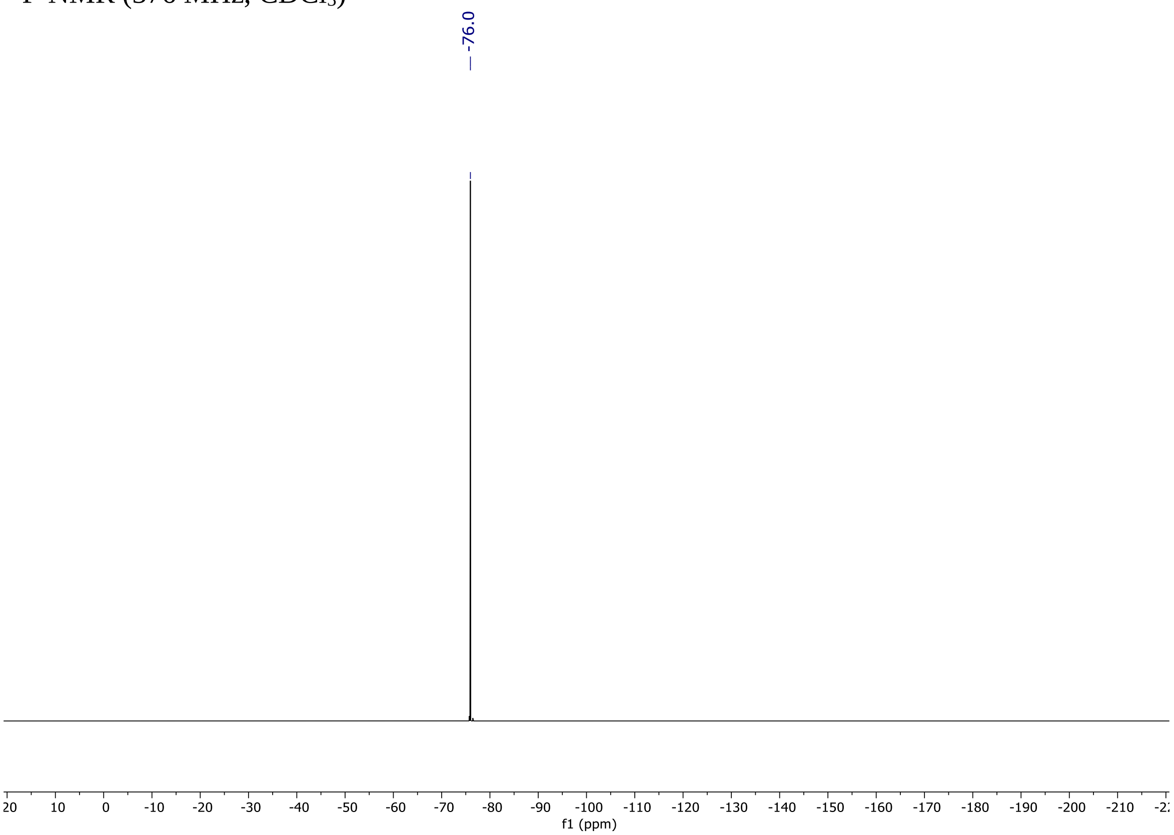

HSQC ($CDCl_3$)

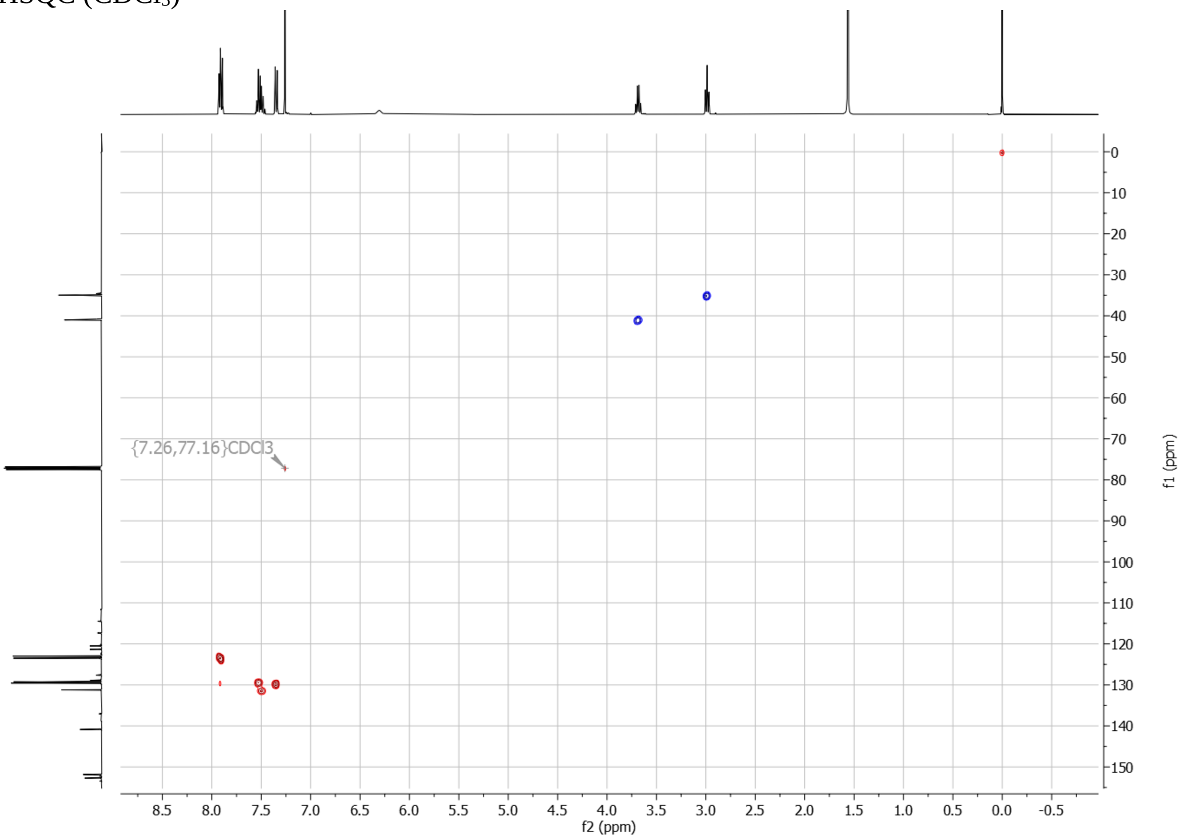

(*E*)-2,2,2-Trifluoro-*N*-(4-(phenyldiazenyl)benzyl)acetamide (1o)

^{1}H-NMR (400 MHz, $CDCl_3$)

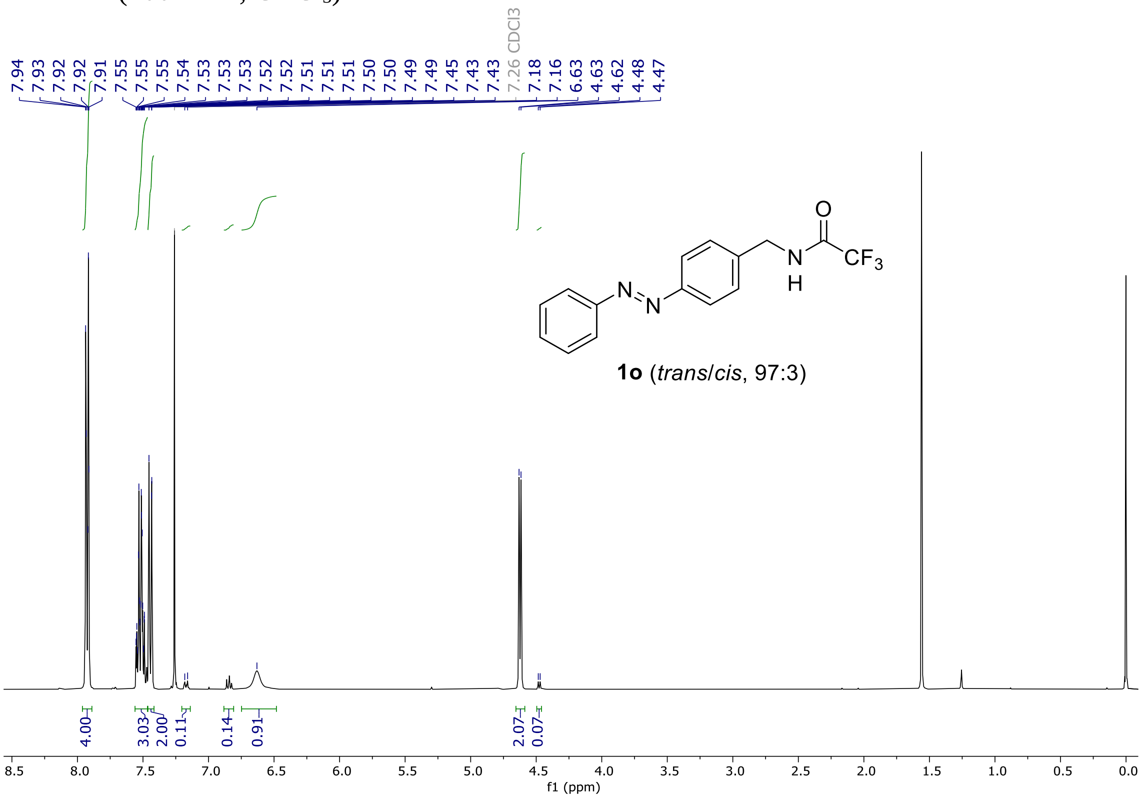

^{13}C{^{1}H}-NMR (100 MHz, $CDCl_3$)

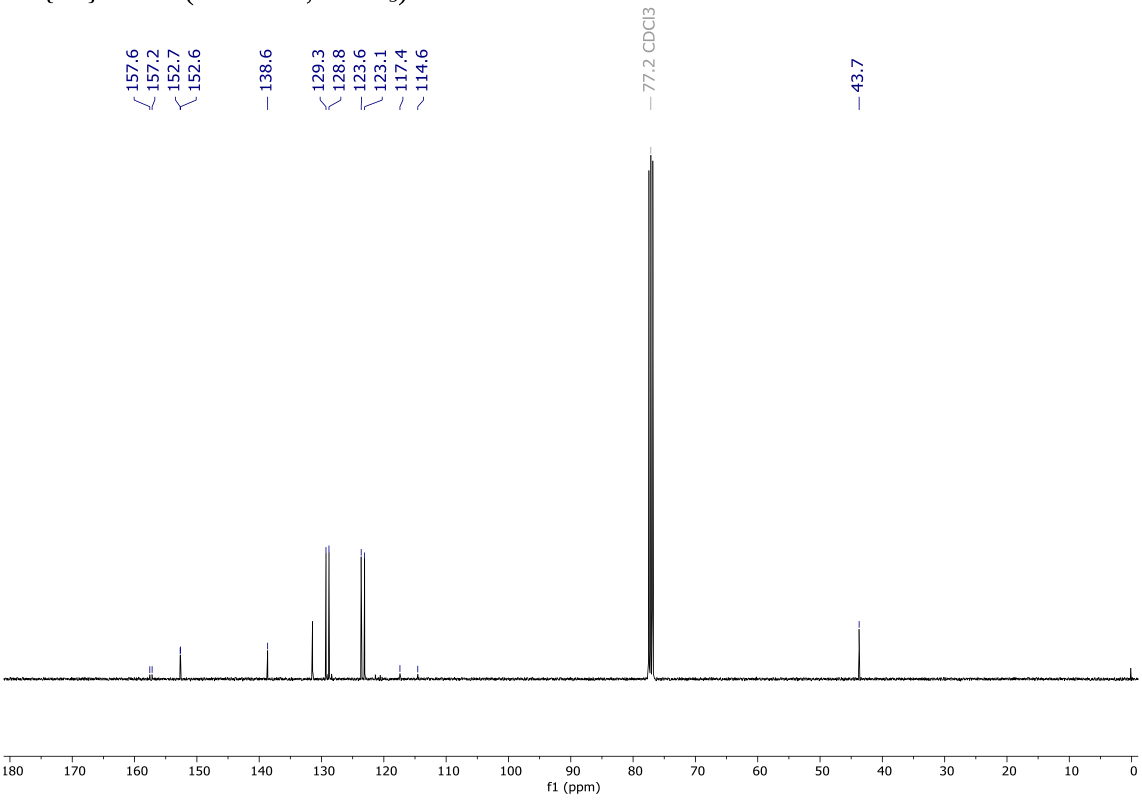

^{19}F-NMR (376 MHz, $CDCl_3$)

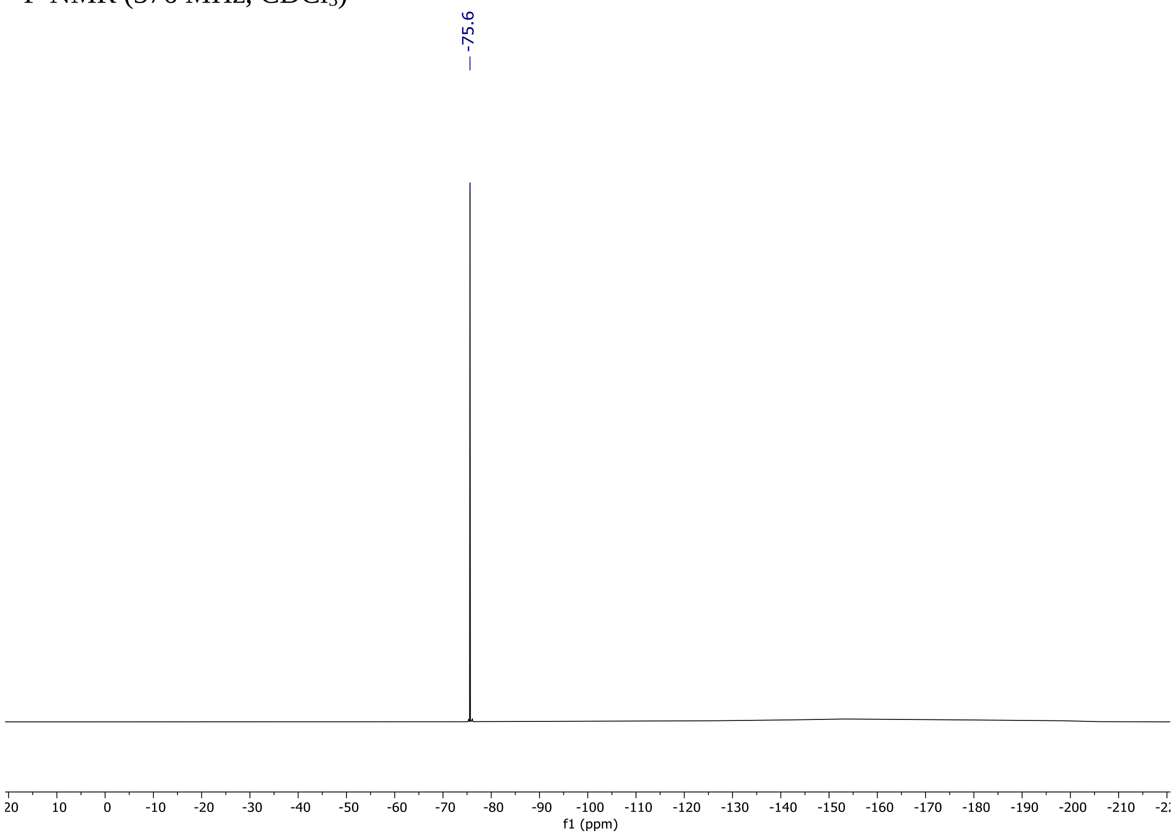

HSQC ($CDCl_3$)

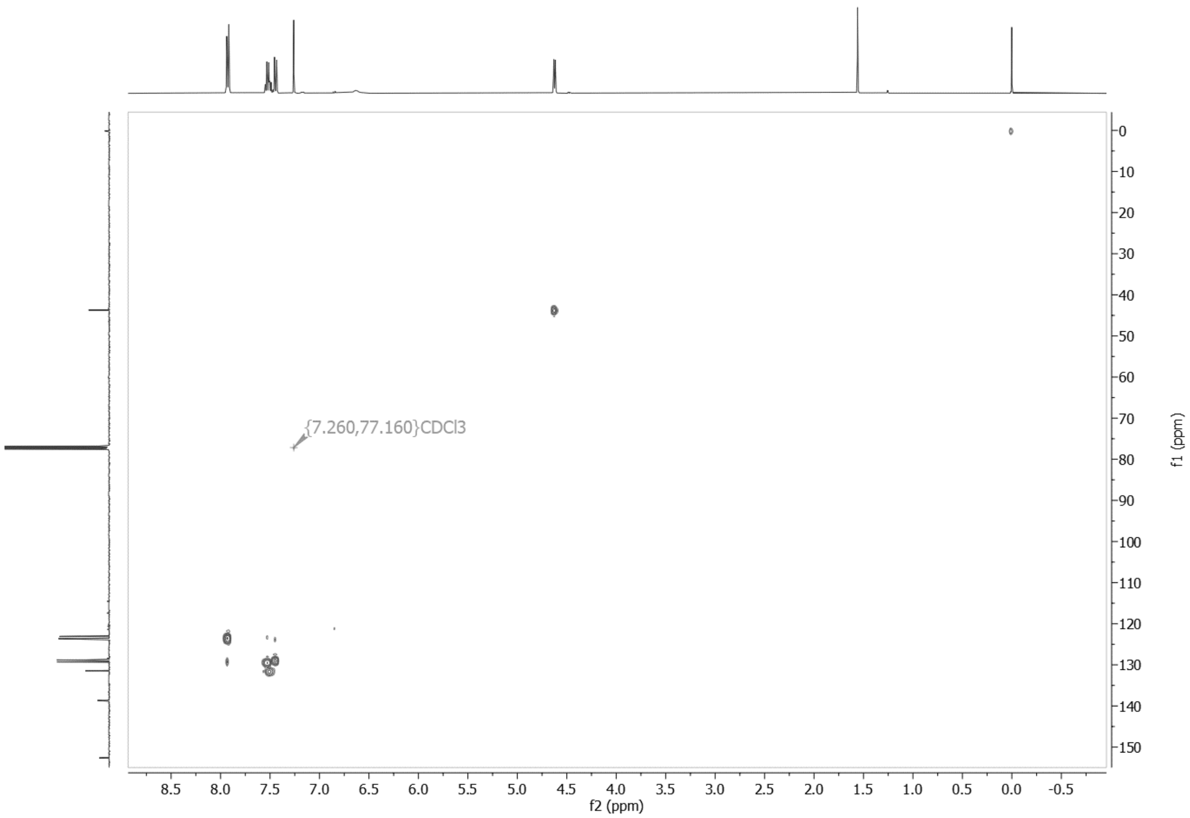

Methyl (*E*)-3-(4-((4-(2-(2,2,2-trifluoroacetamido)ethyl)phenyl)diazenyl)phenyl)propanoate (1p)

^{1}H-NMR (400 MHz, $CDCl_3$)

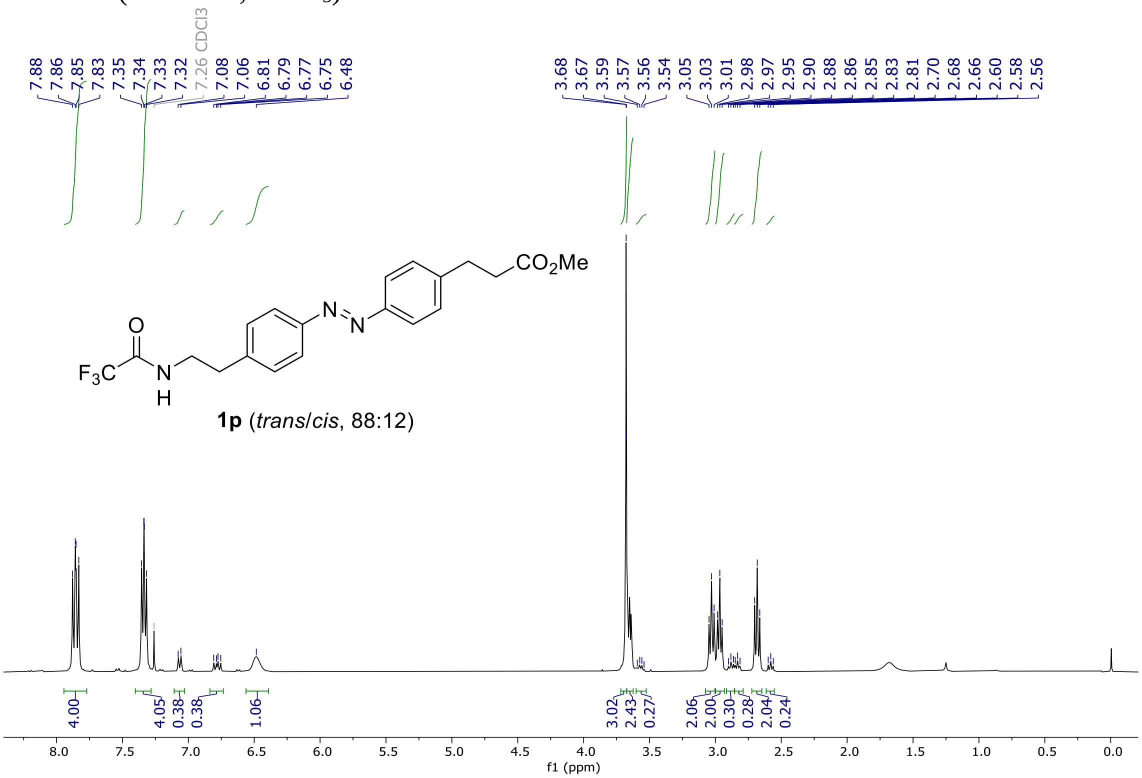

^{13}C{^{1}H}-NMR (100 MHz, $CDCl_3$)

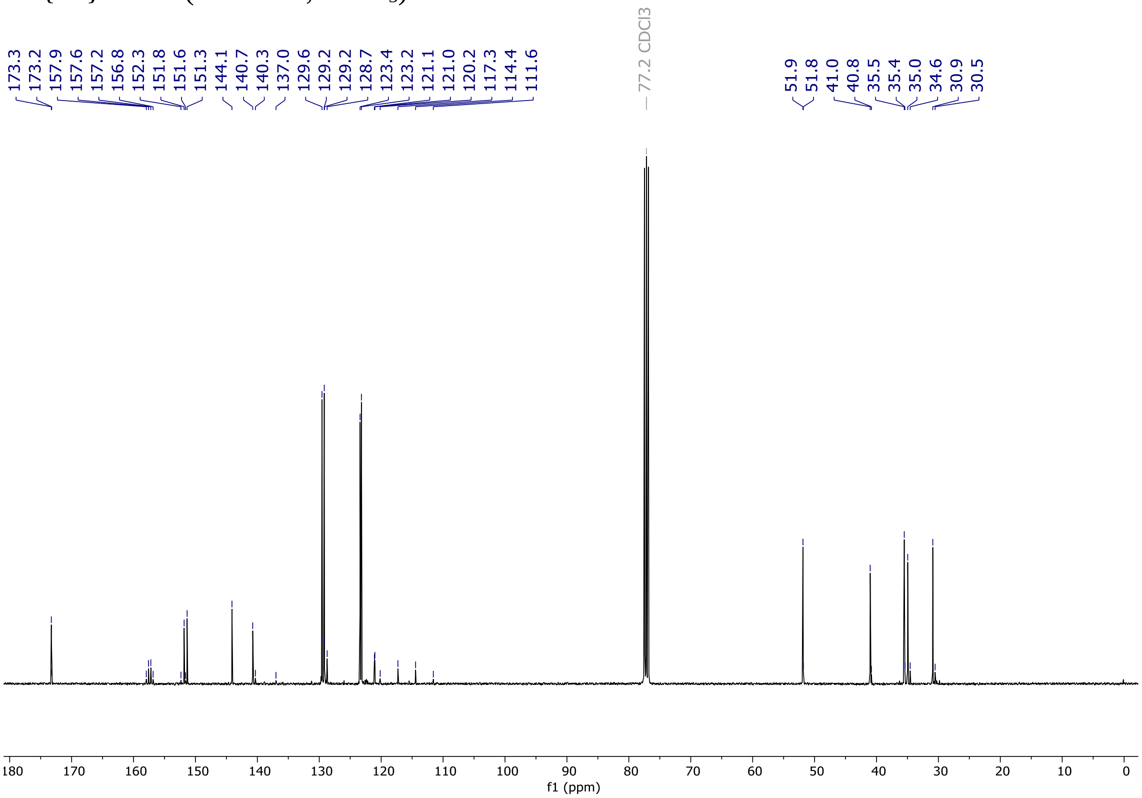

^{19}F-NMR (376 MHz, $CDCl_3$)

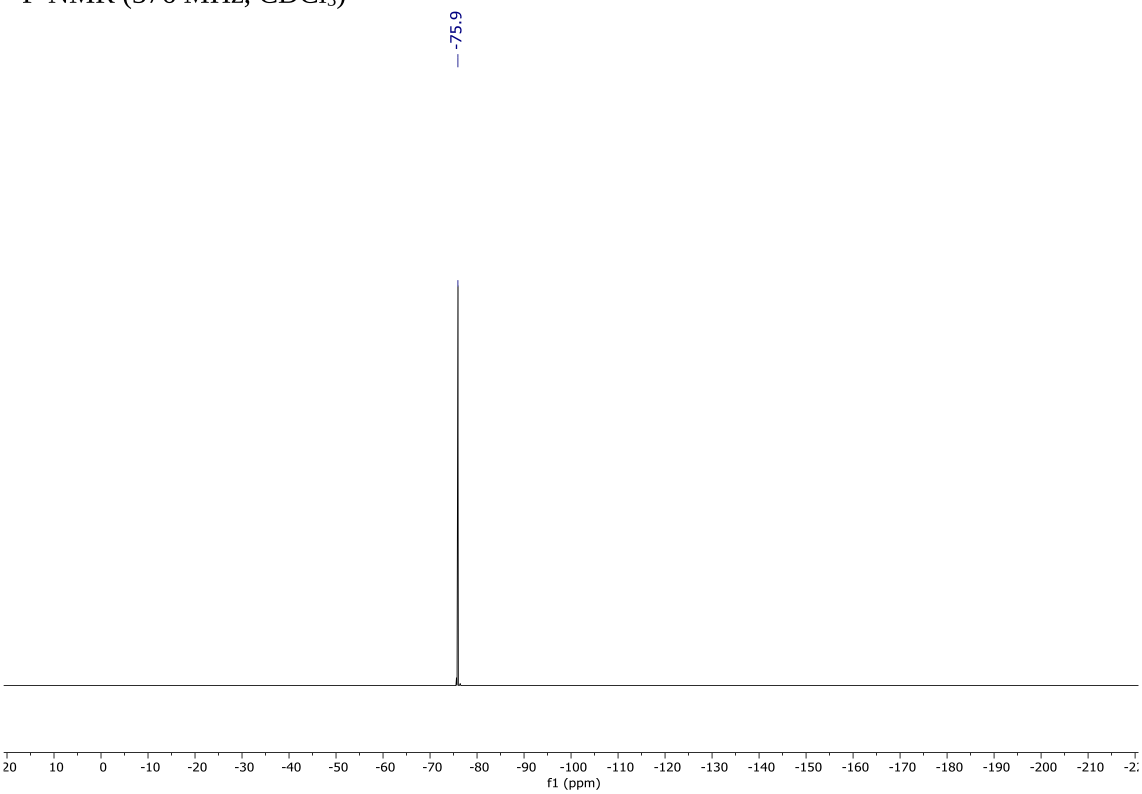

HSQC ($CDCl_3$)

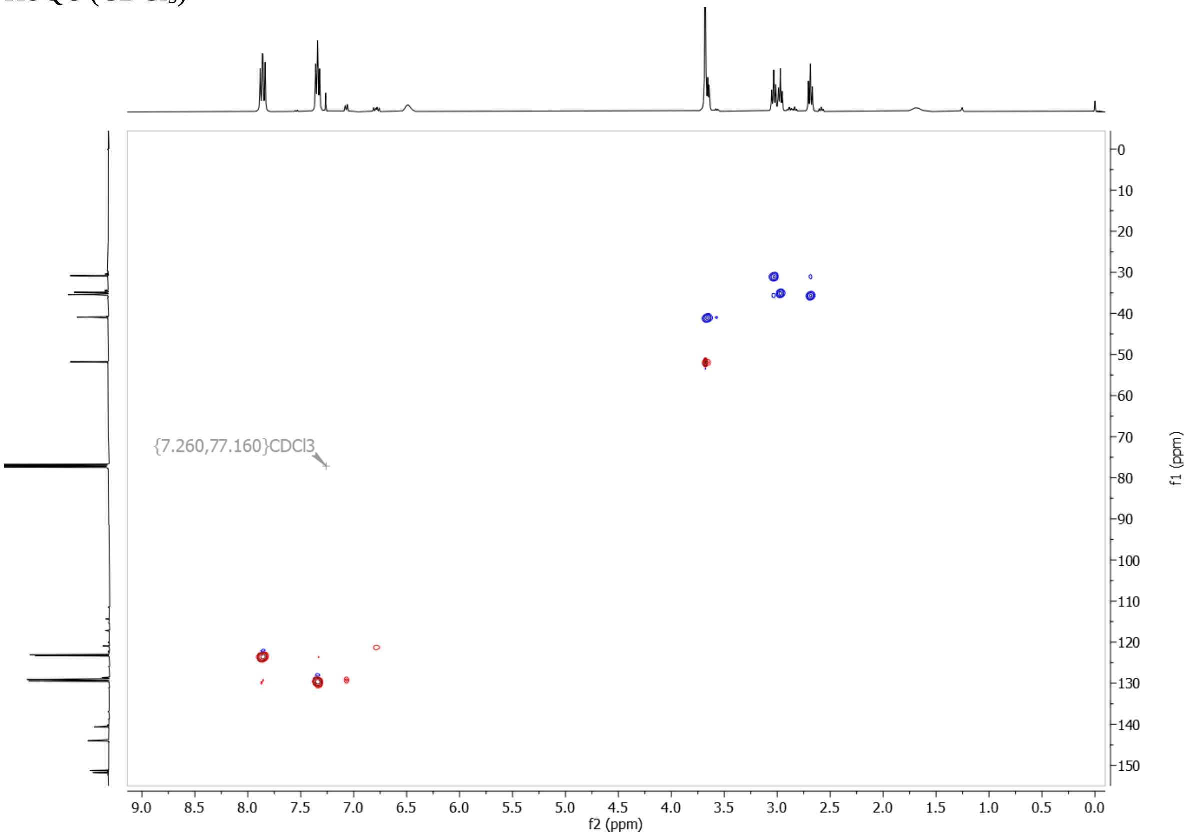

(*E*)-1,2-Bis(2,6-dibromophenyl)diazene (2a)

^{1}H-NMR (400 MHz, $CDCl_3$)

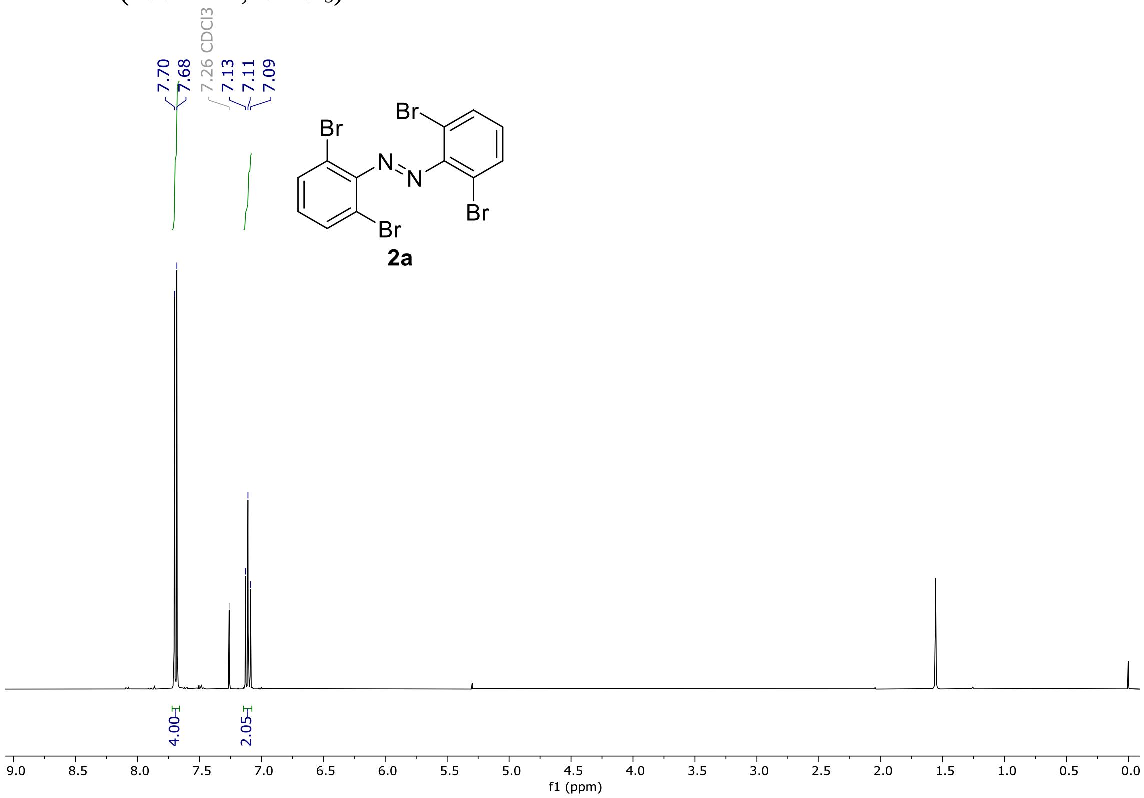

^{13}C{^{1}H}-NMR (100 MHz, $CDCl_3$)

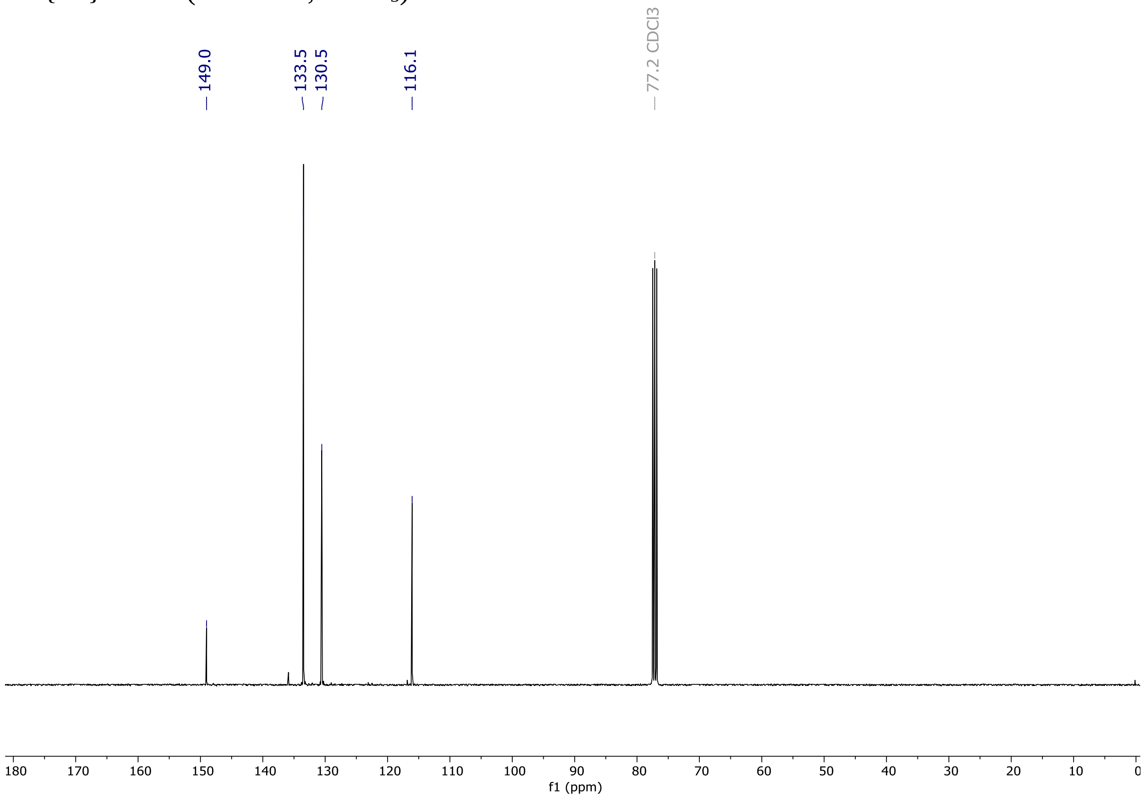

HSQC ($CDCl_3$)

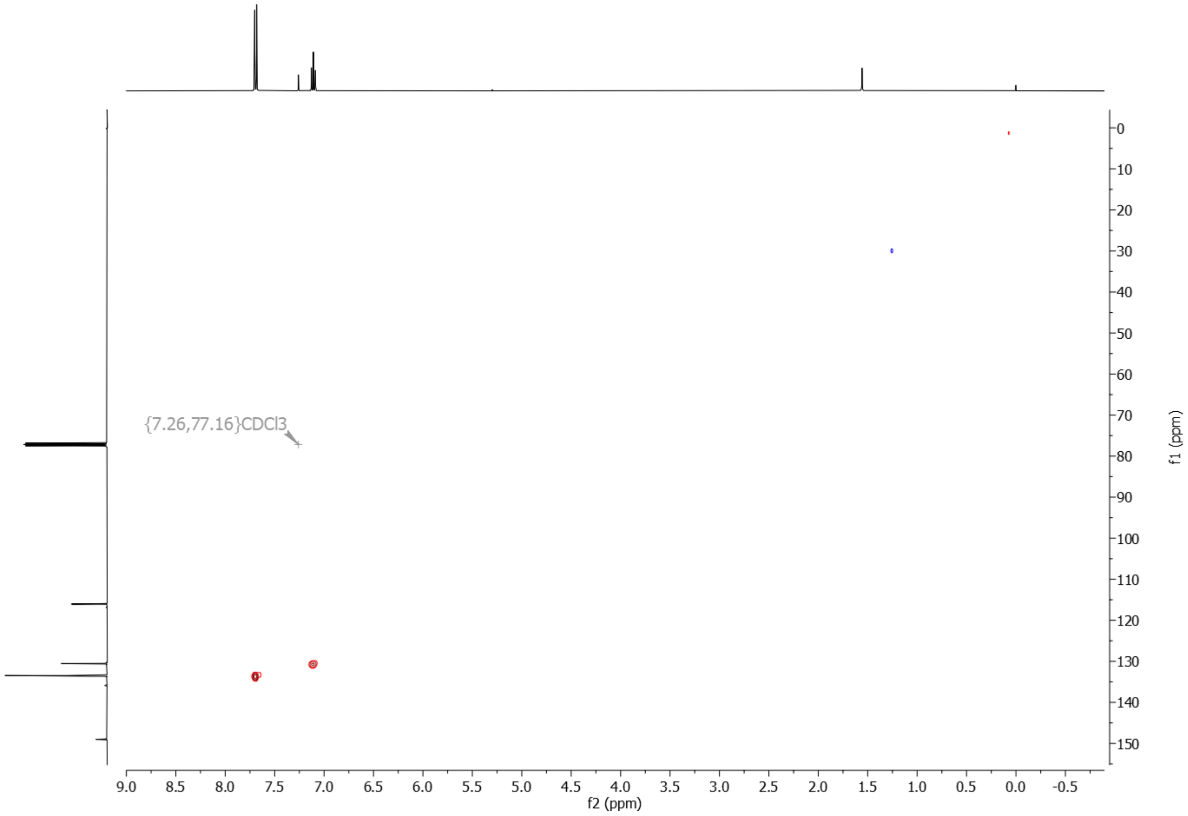

(*E*)-1-(2,6-dibromo-4-Methylphenyl)-2-(2,6-dibromophenyl)diazene (2b)

^{1}H-NMR (400 MHz, $CDCl_3$)

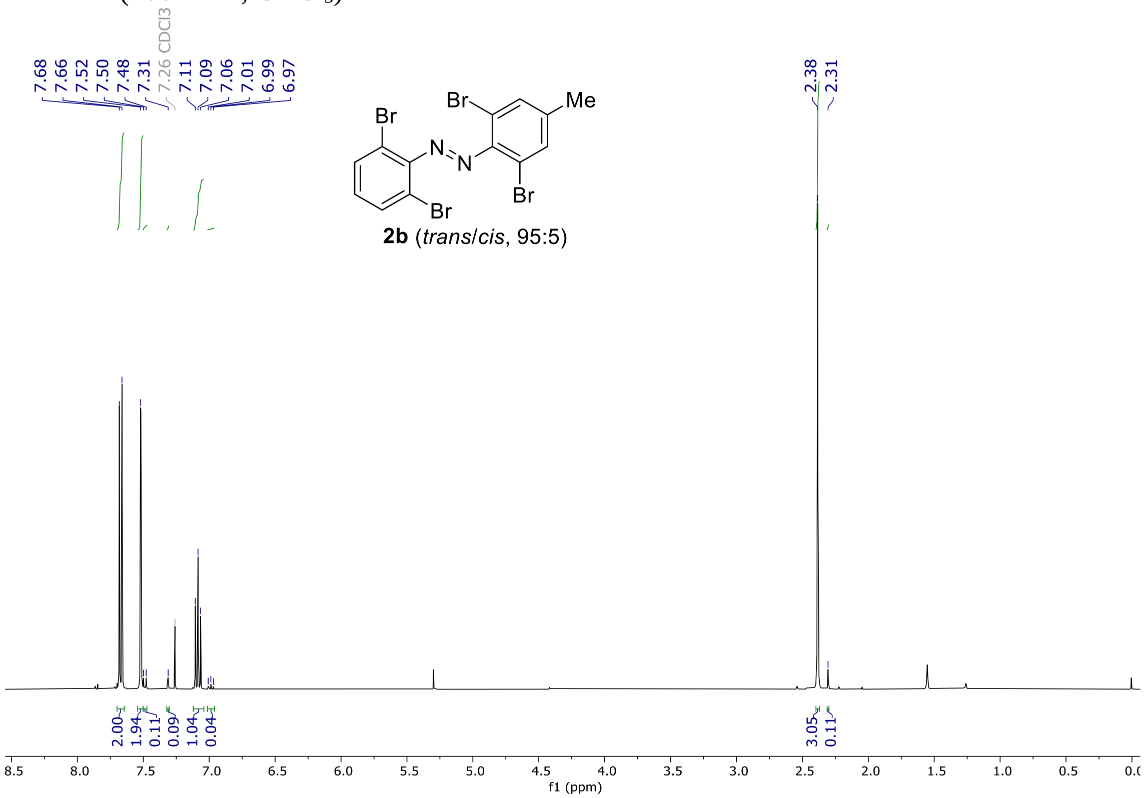

^{13}C{^{1}H}-NMR (100 MHz, $CDCl_3$)

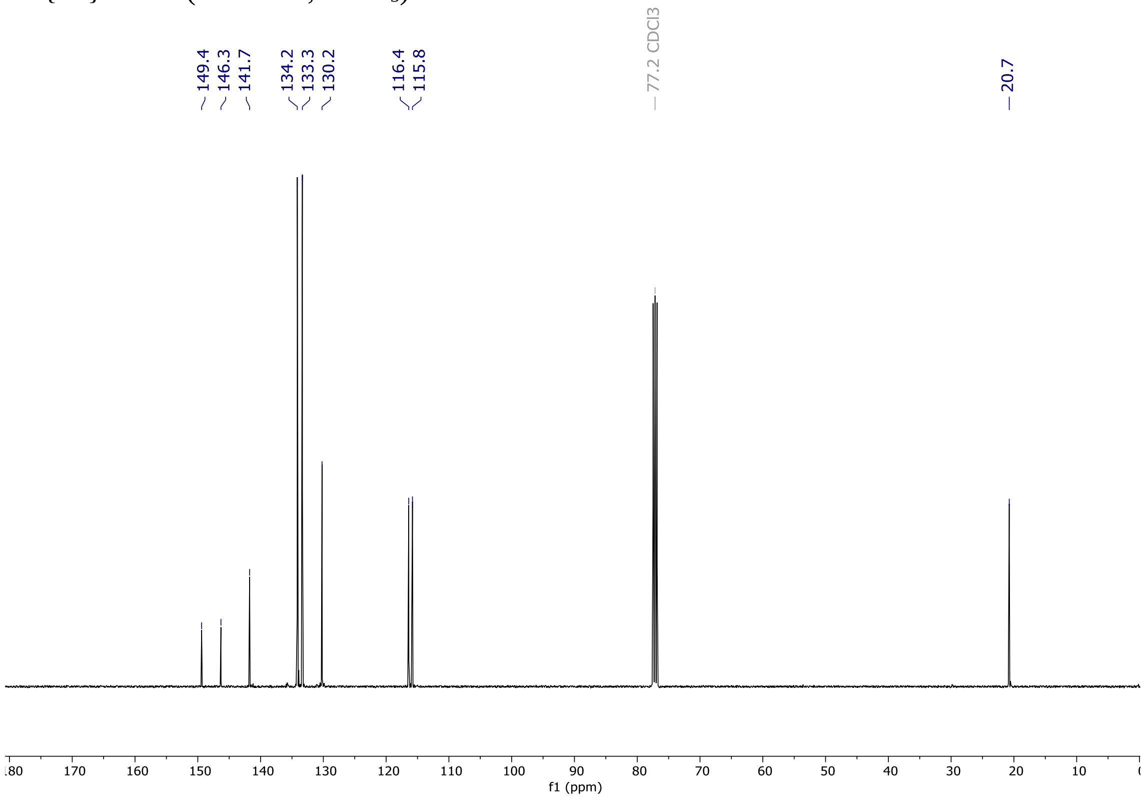

HSQC ($CDCl_3$)

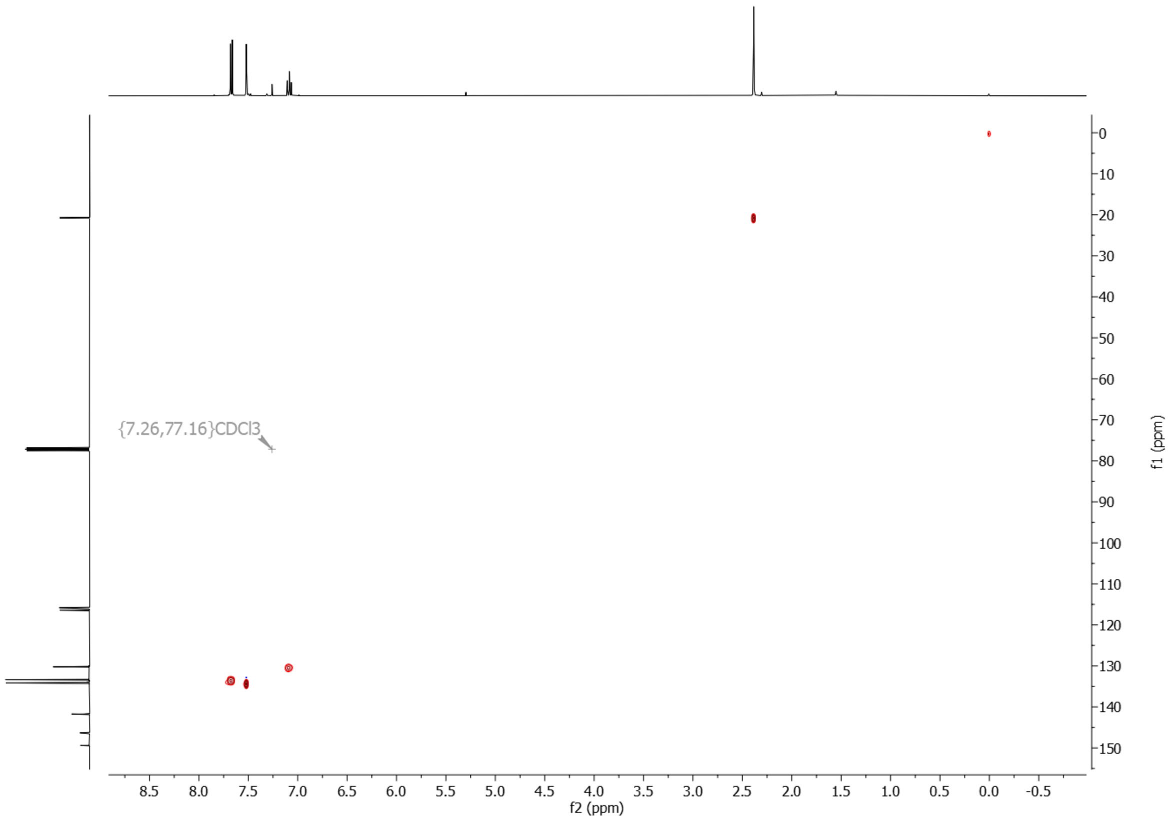

(*E*)-1,2-Bis(2,6-dibromo-4-methylphenyl)diazene (2c)

^{1}H-NMR (400 MHz, $CDCl_3$)

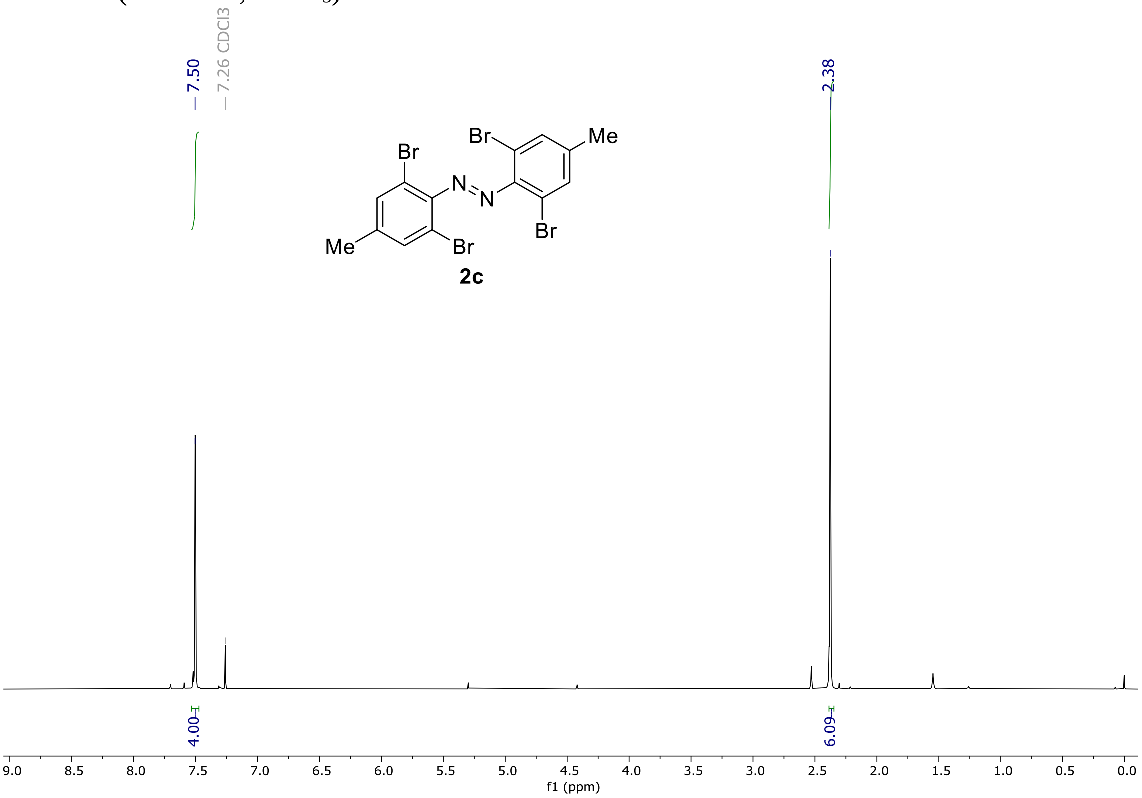

^{13}C{^{1}H}-NMR (100 MHz, $CDCl_3$)

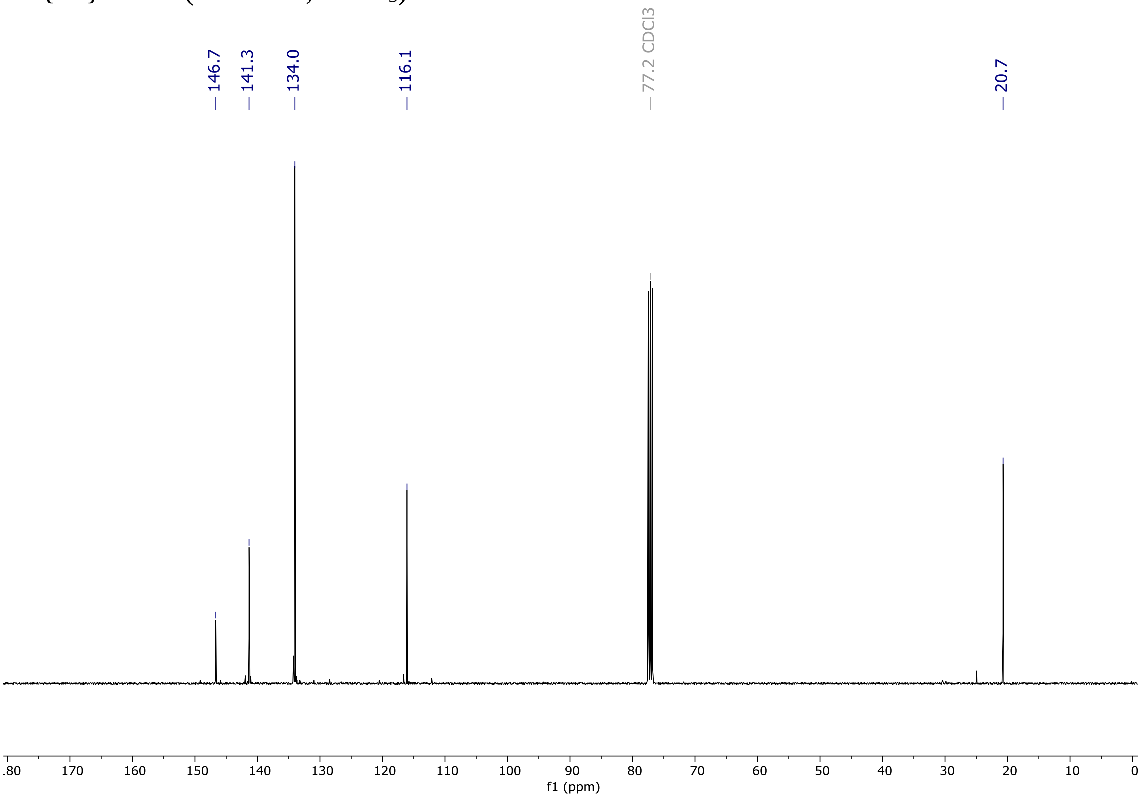

HSQC ($CDCl_3$)

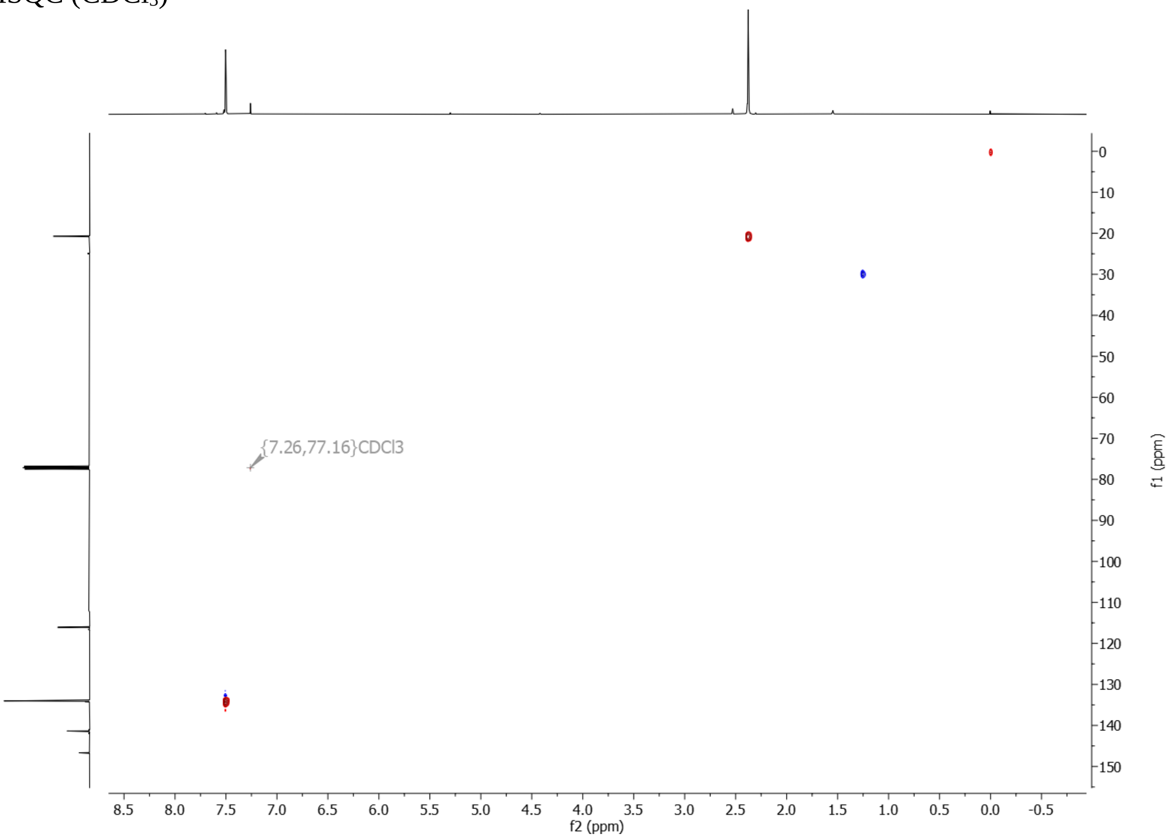

{7.26,77.16}CDCl3
8.5
8.0
7.5
7.0
6.5
6.0
5.5
5.0
4.5
4.0
3.5
3.0
2.5
2.0
1.5
1.0
0.5
0.0
-0.5
f2 (ppm)
0
10
20
30
40
50
60
70
80
90
100
110
120
130
140
150
f1 (ppm)

(*E*)-1-(2,6-Dibromo-4-methoxyphenyl)-2-(2,6-dibromophenyl)diazene (2d)

^{1}H-NMR (400 MHz, $CDCl_3$)

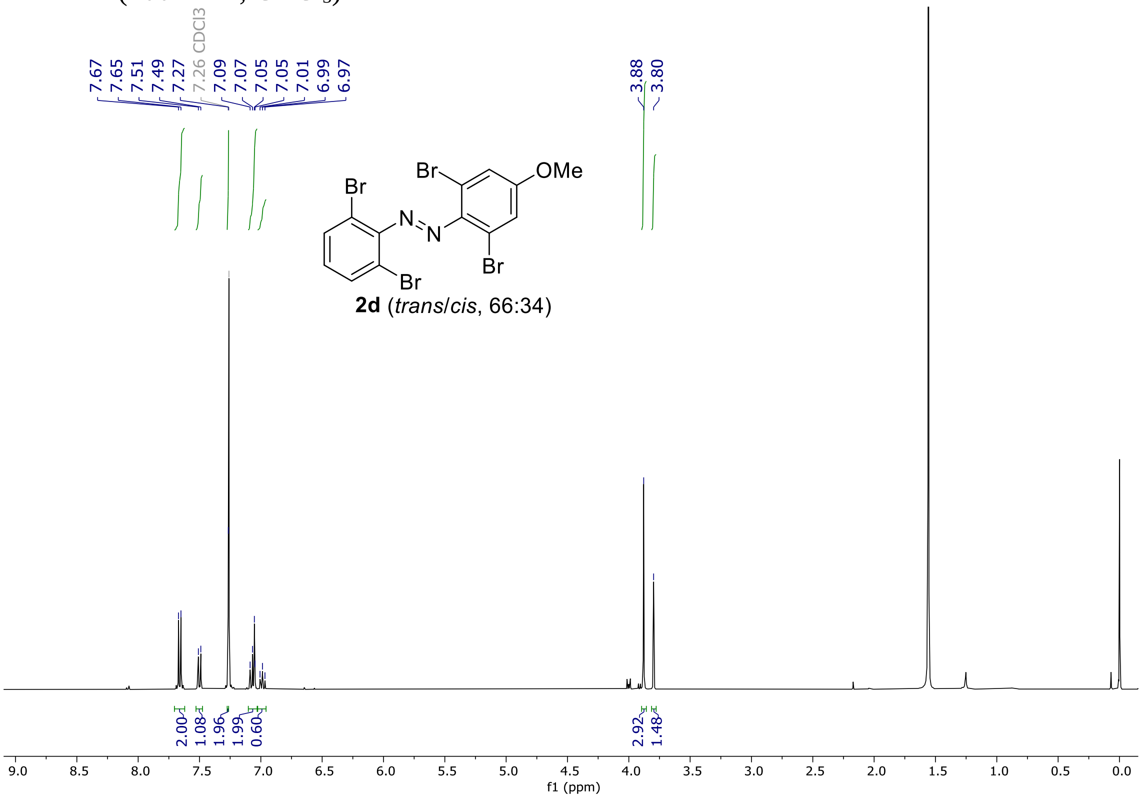

^{13}C{^{1}H}-NMR (100 MHz, $CDCl_3$)

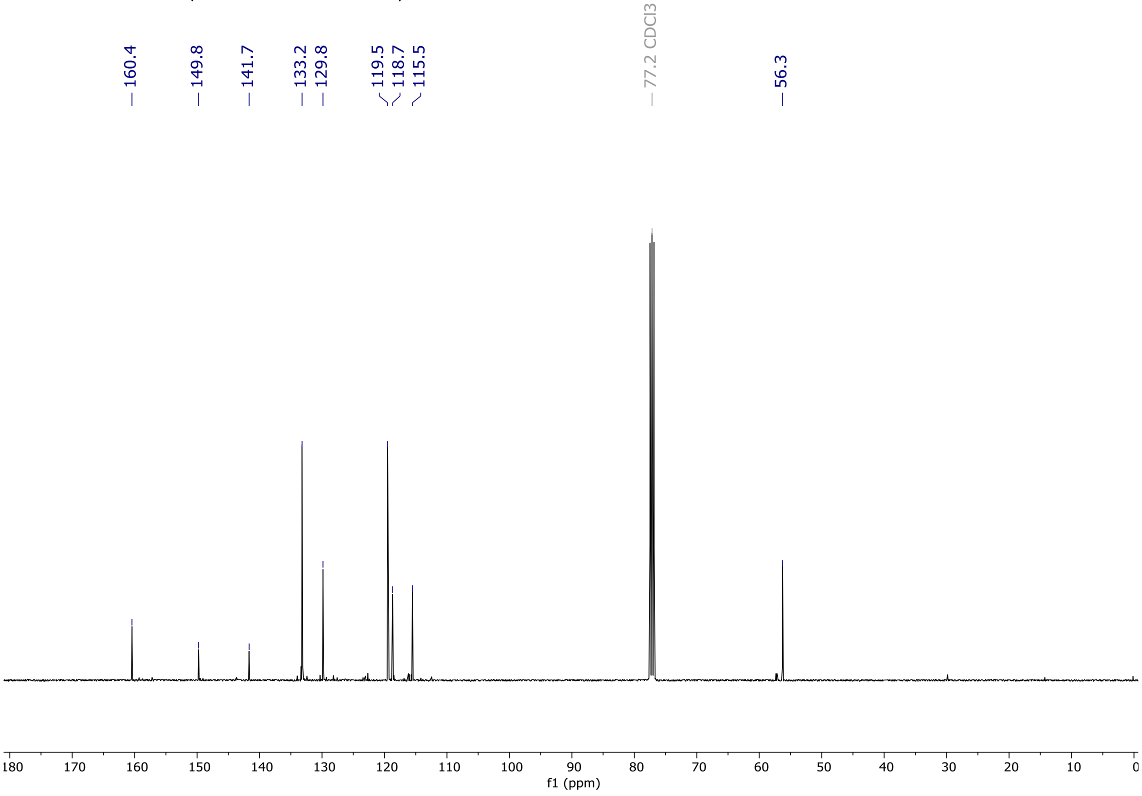

HSQC ($CDCl_3$)

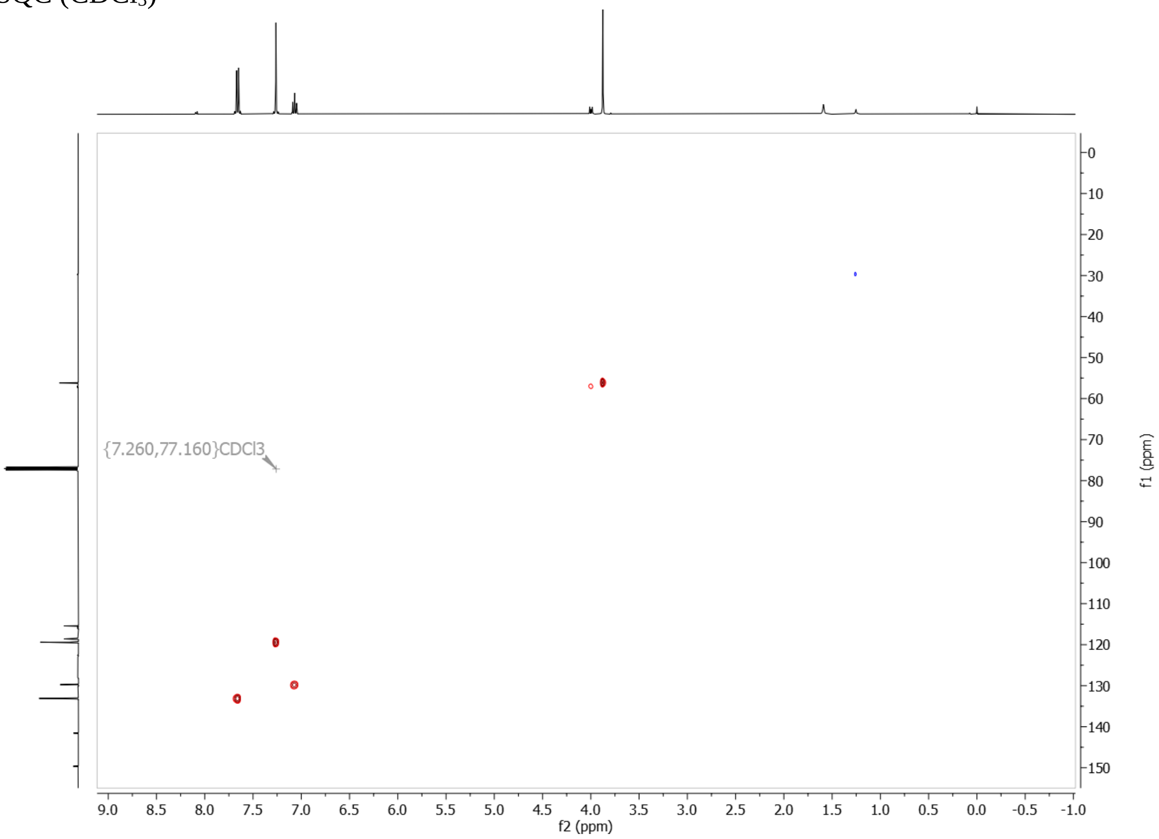

{7.260,77.160}CDCl3
0
10
20
30
40
50
60
70
80
90
100
110
120
130
140
150
f1 (ppm)
9.0
8.5
8.0
7.5
7.0
6.5
6.0
5.5
5.0
4.5
4.0
3.5
3.0
2.5
2.0
1.5
1.0
0.5
0.0
-0.5
-1.0
f2 (ppm)

(*E*)-1,2-Bis(2,6-dibromo-4-chlorophenyl)diazene (2e)

^{1}H-NMR (400 MHz, $CDCl_3$)

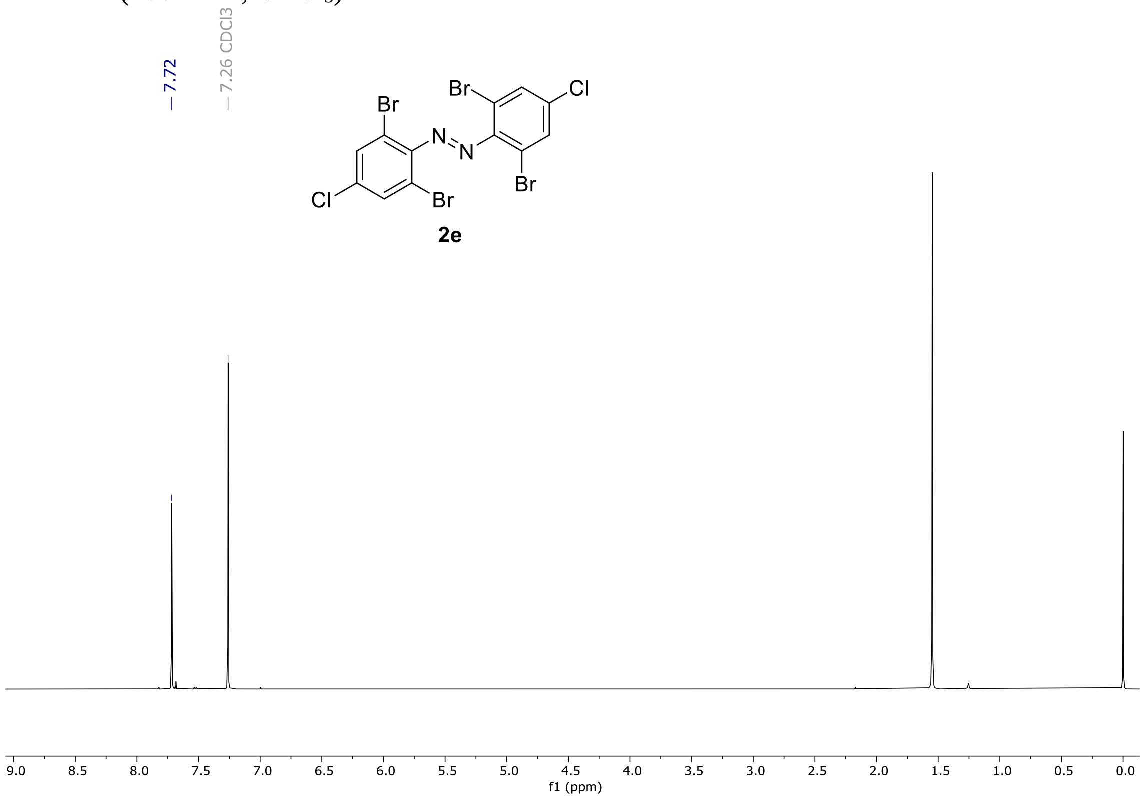

$^{13}C\{^{1}H\}$-NMR (100 MHz, $CDCl_3$)

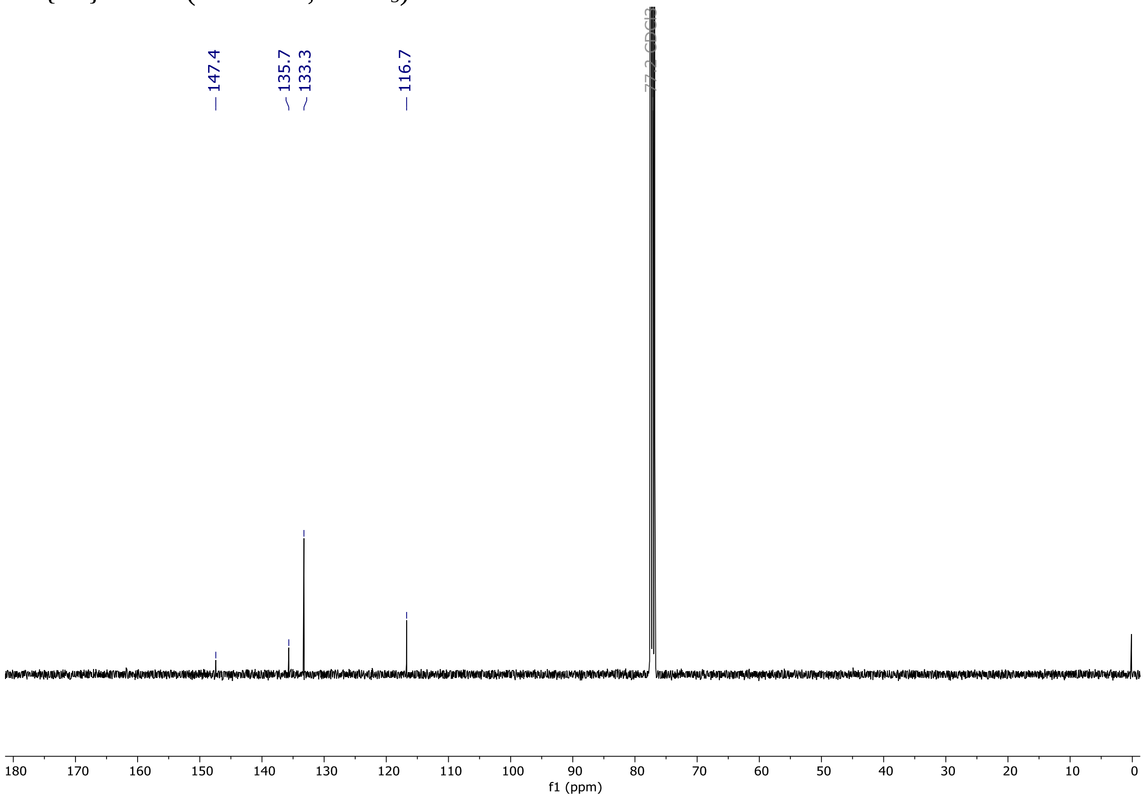

HSQC ($CDCl_3$)

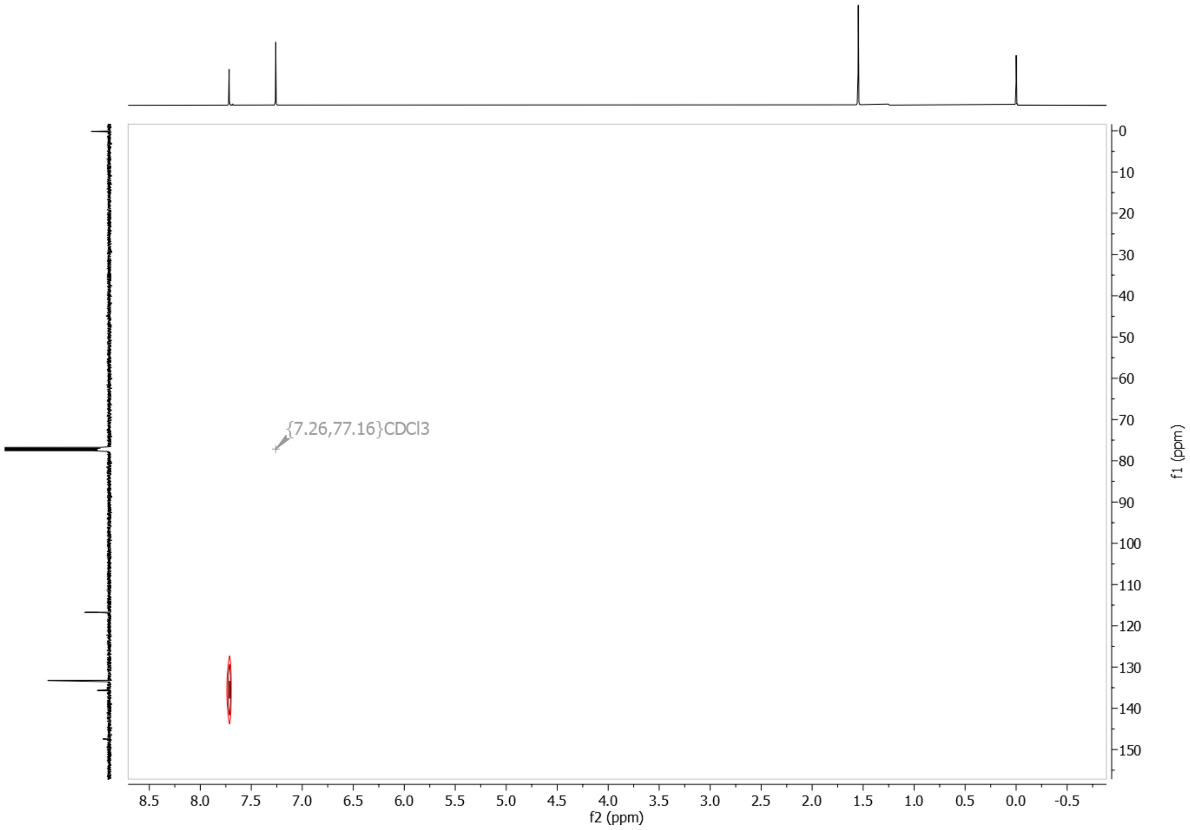

(*E*)-1,2-Bis(2,4,6-tribromophenyl)diazene (2f)

^{1}H-NMR (400 MHz, $CDCl_3$)

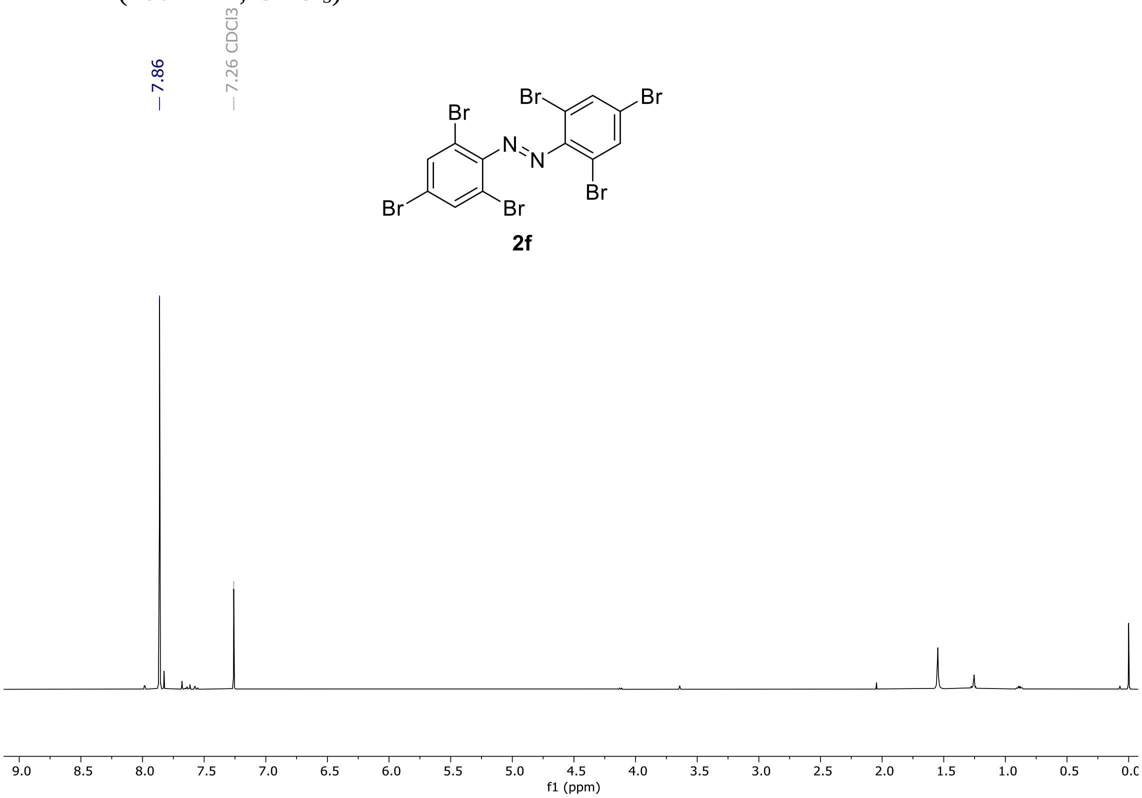

^{13}C{^{1}H}-NMR (100 MHz, $CDCl_3$)

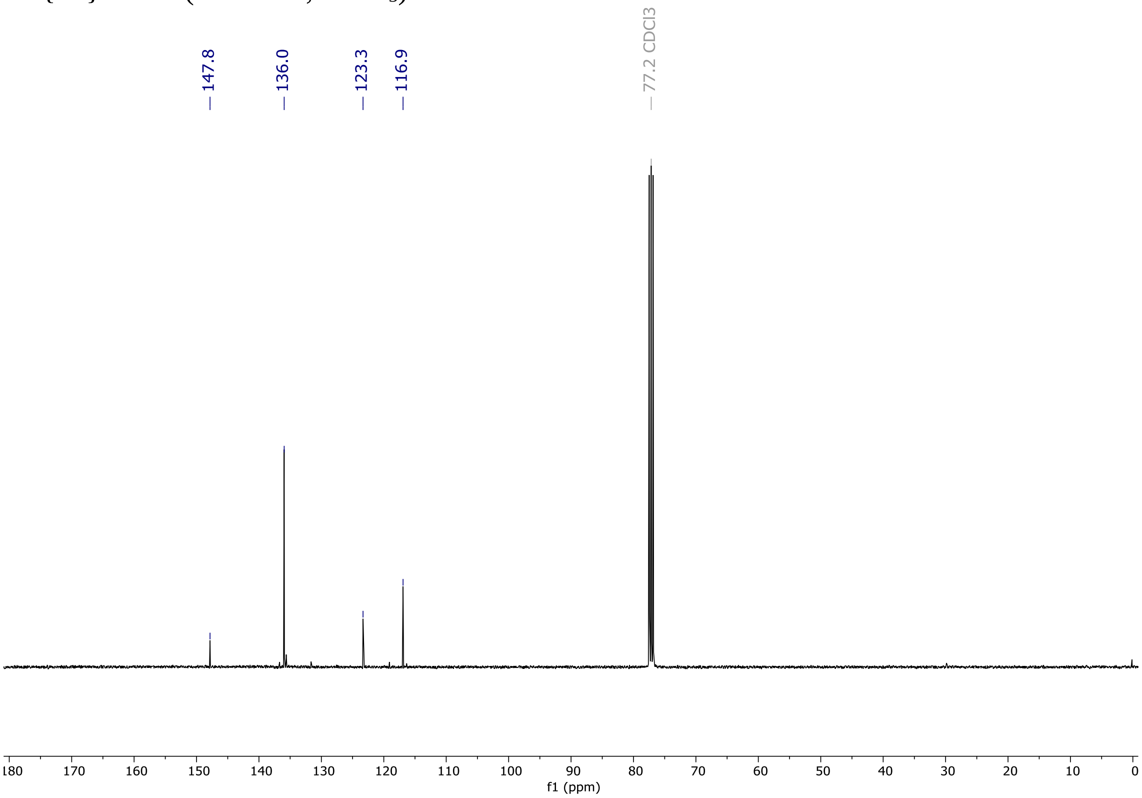

HSQC ($CDCl_3$)

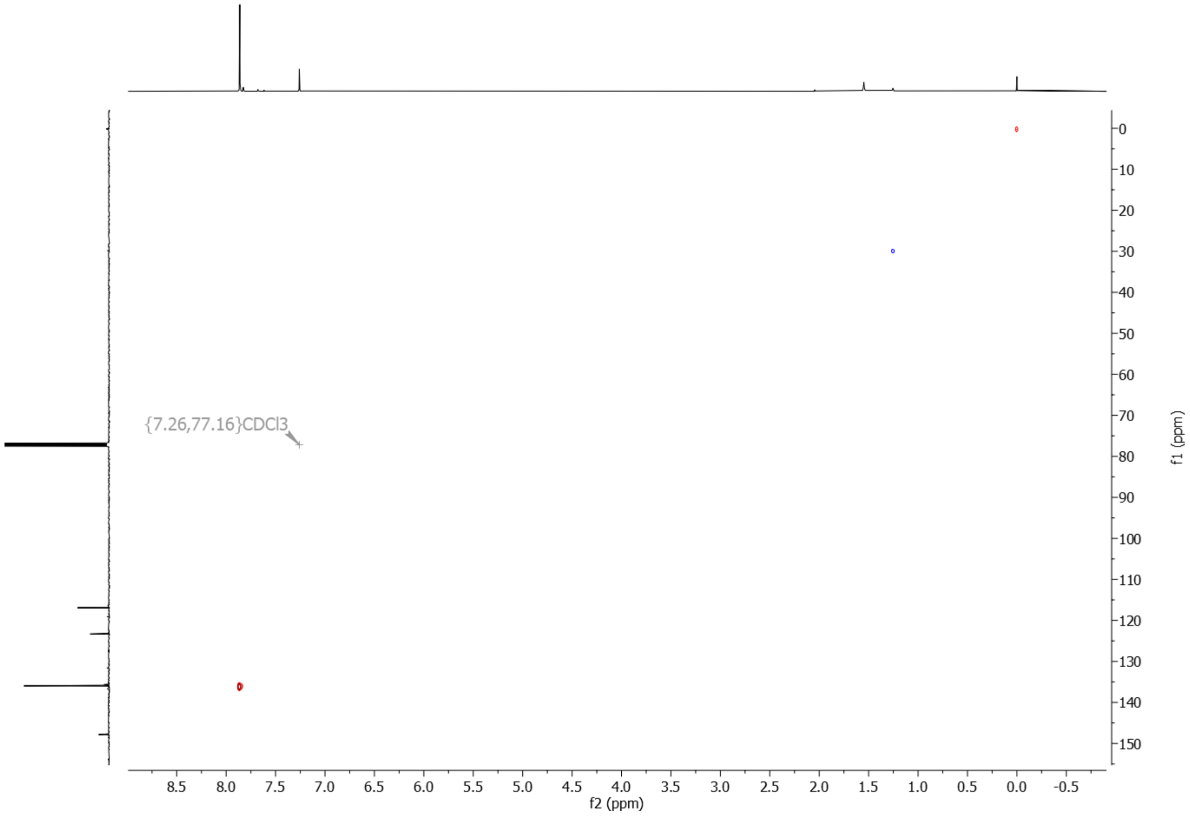

(*E*)-1,2-Bis(2,6-dibromo-4-iodophenyl)diazene (2g)

^{1}H-NMR (400 MHz, $CDCl_3$)

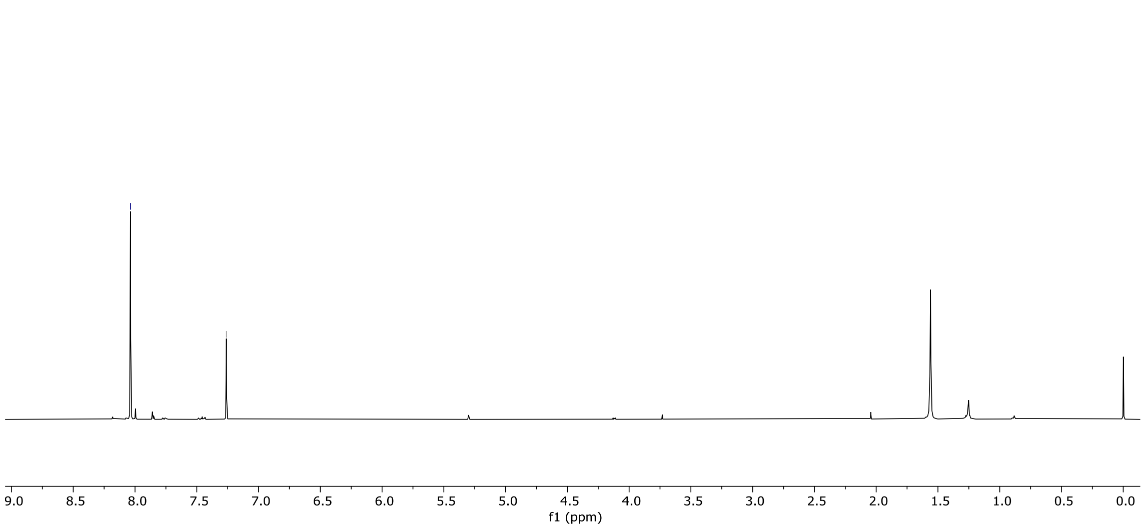

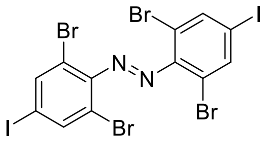

2g

^{13}C{^{1}H}-NMR (100 MHz, $CDCl_3$)

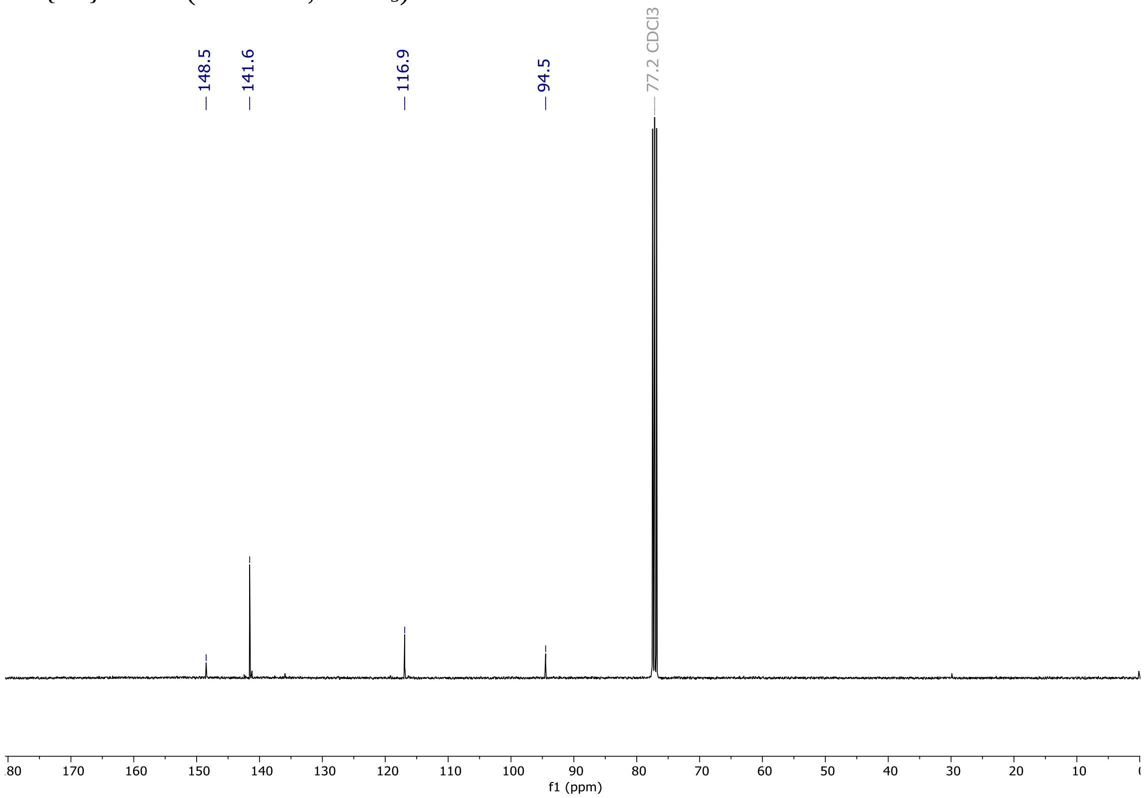

HSQC ($CDCl_3$)

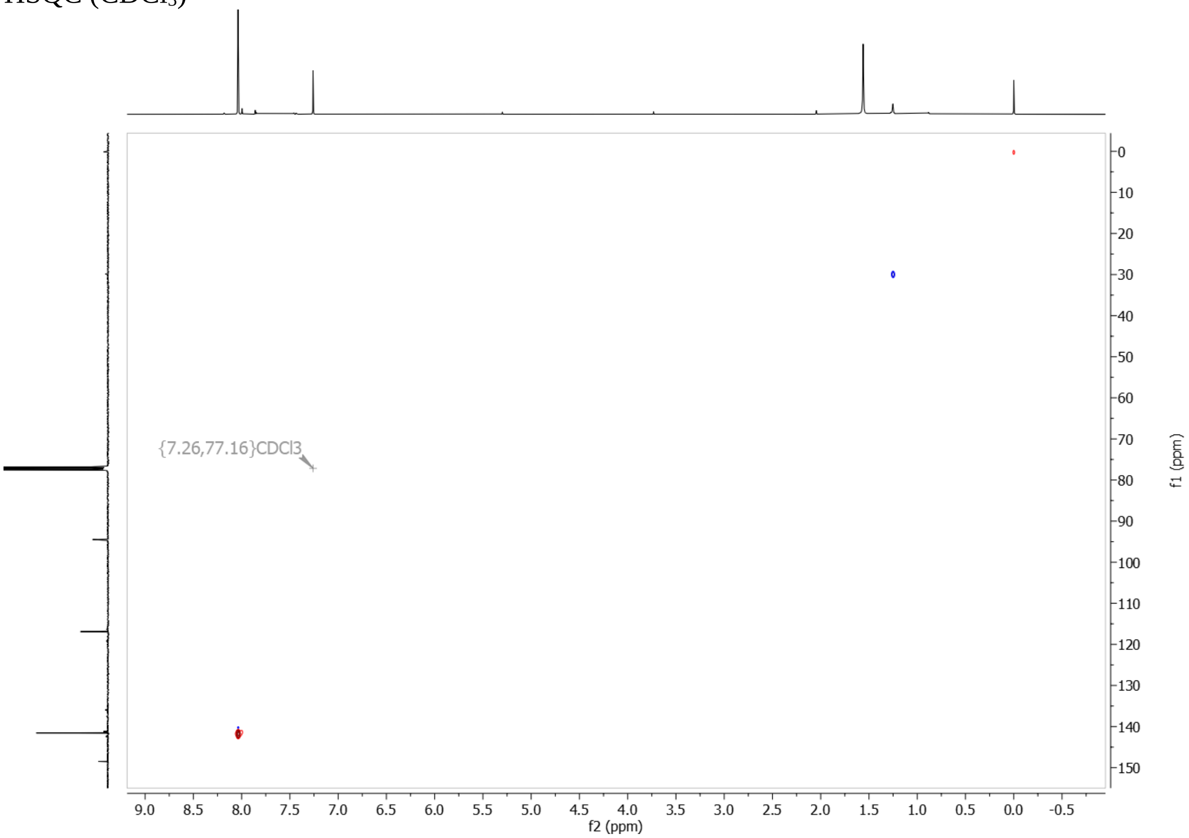

(*E*)-3-(3,5-Dibromo-4-((2,6-dibromophenyl)diazenyl)phenyl)propanoic acid (2i)

^{1}H-NMR (400 MHz, DMSO-d_6)

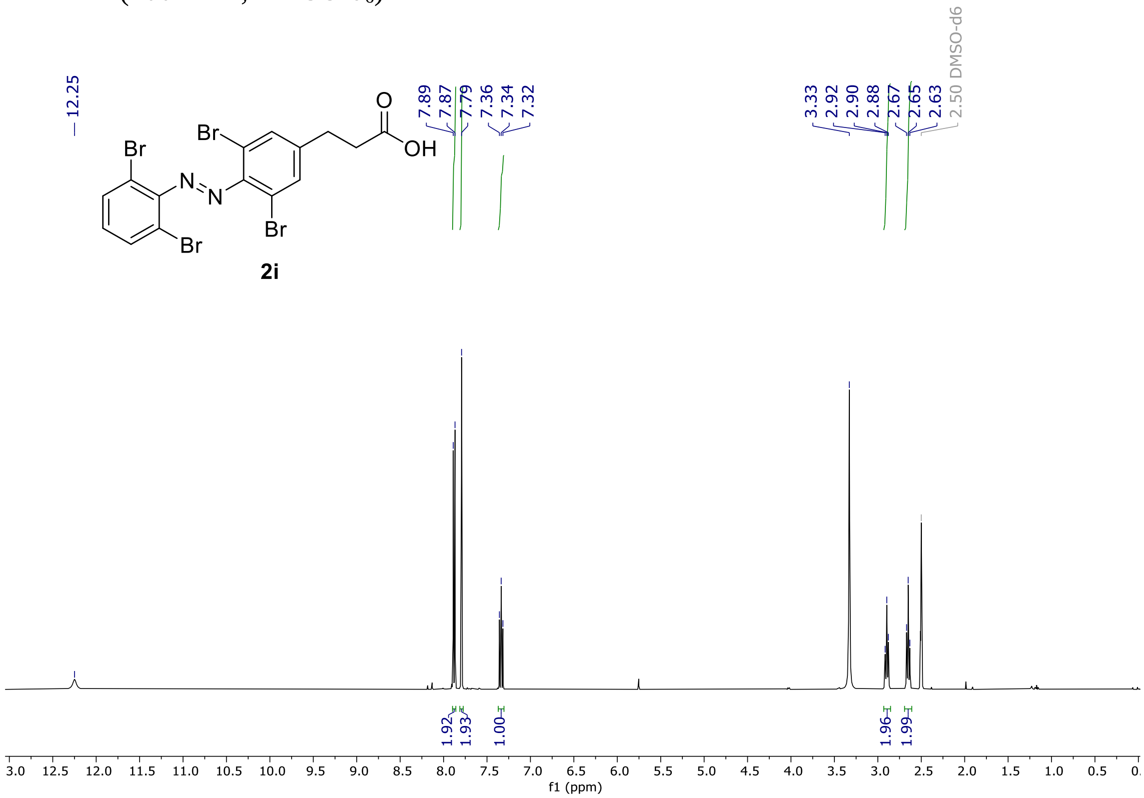

^{13}C{^{1}H}-NMR (100 MHz, DMSO-d_6)

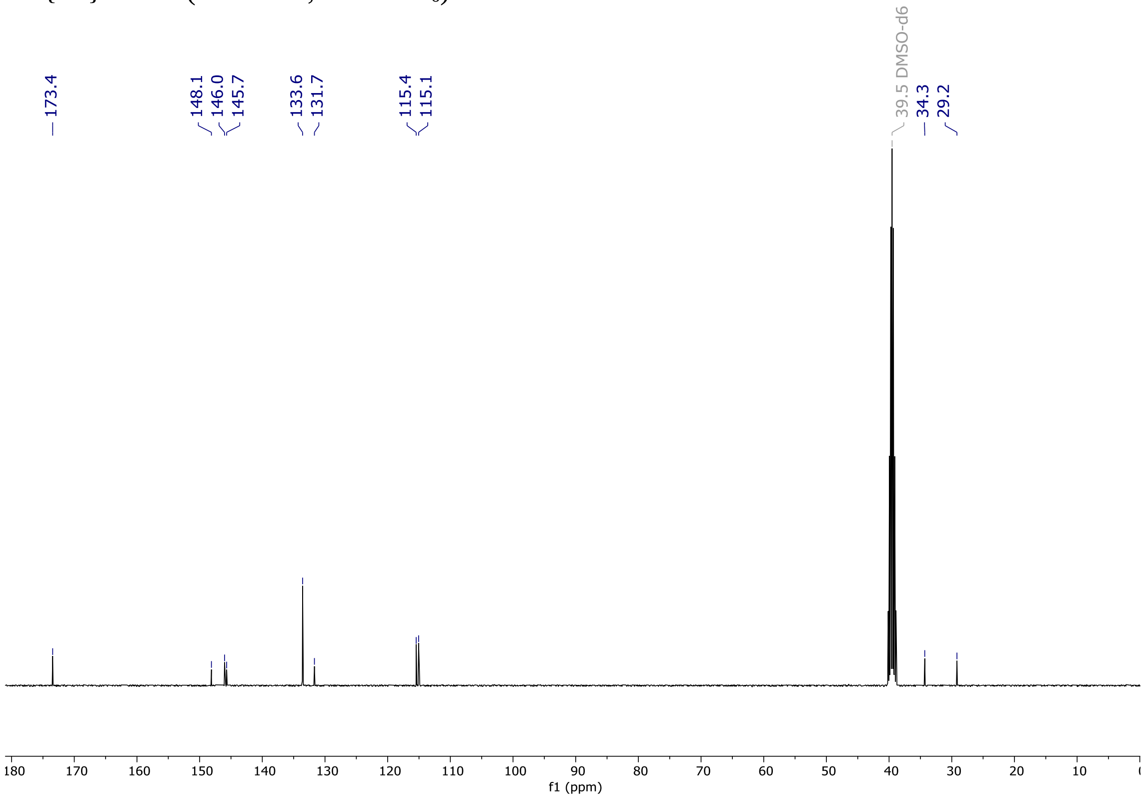

HSQC (DMSO-d_6)

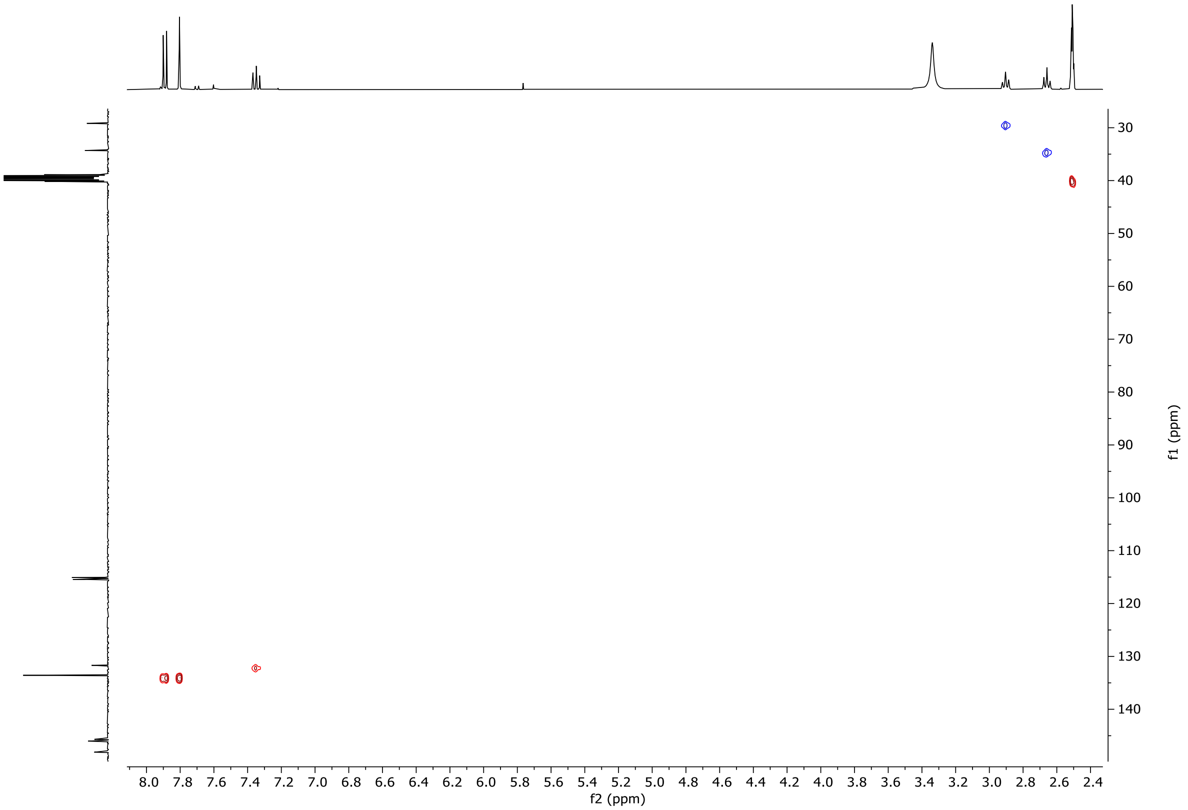

8.0 7.8 7.6 7.4 7.2 7.0 6.8 6.6 6.4 6.2 6.0 5.8 5.6 5.4 5.2 5.0 4.8 4.6 4.4 4.2 4.0 3.8 3.6 3.4 3.2 3.0 2.8 2.6 2.4
f2 (ppm)
30 40 50 60 70 80 90 100 110 120 130 140
f1 (ppm)

(*E*)-3-(3,5-Dibromo-4-((2,6-dibromophenyl)diazenyl)benzoic acid (2j)

^{1}H-NMR (400 MHz, DMSO-d$_6$)

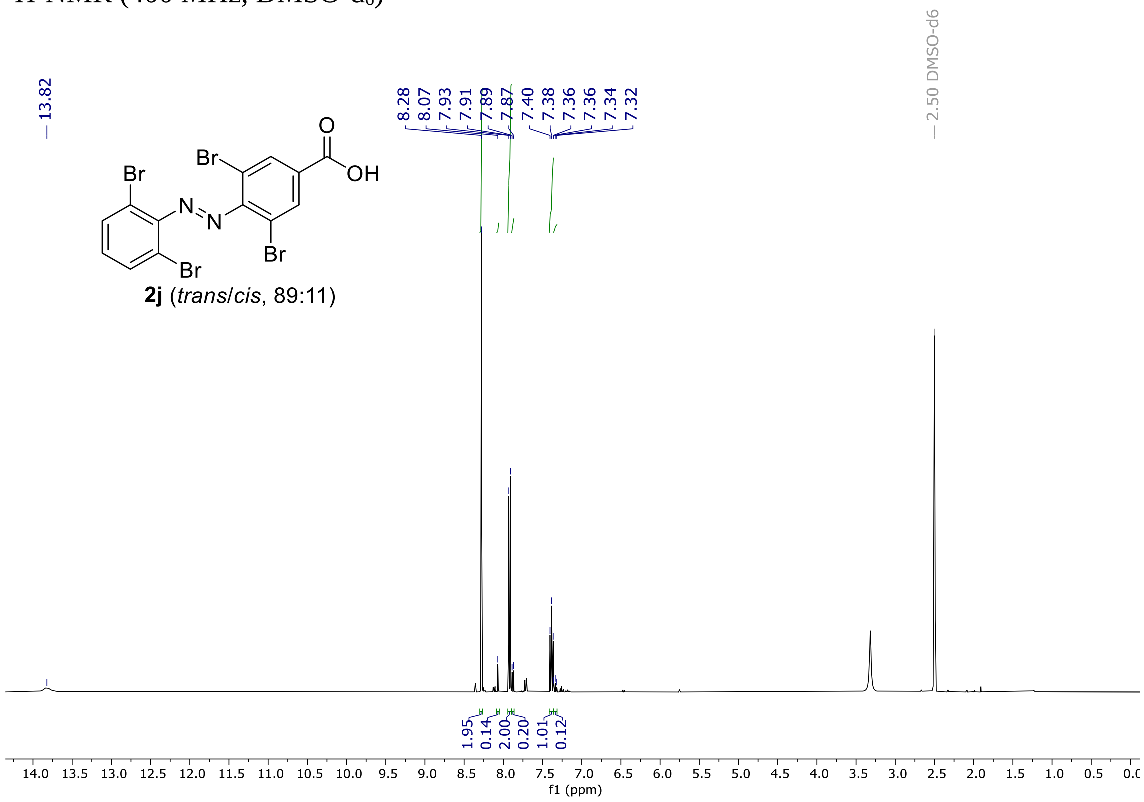

2j (*trans*/*cis*, 89:11)

^{13}C{^{1}H}-NMR (100 MHz, DMSO-d$_6$)

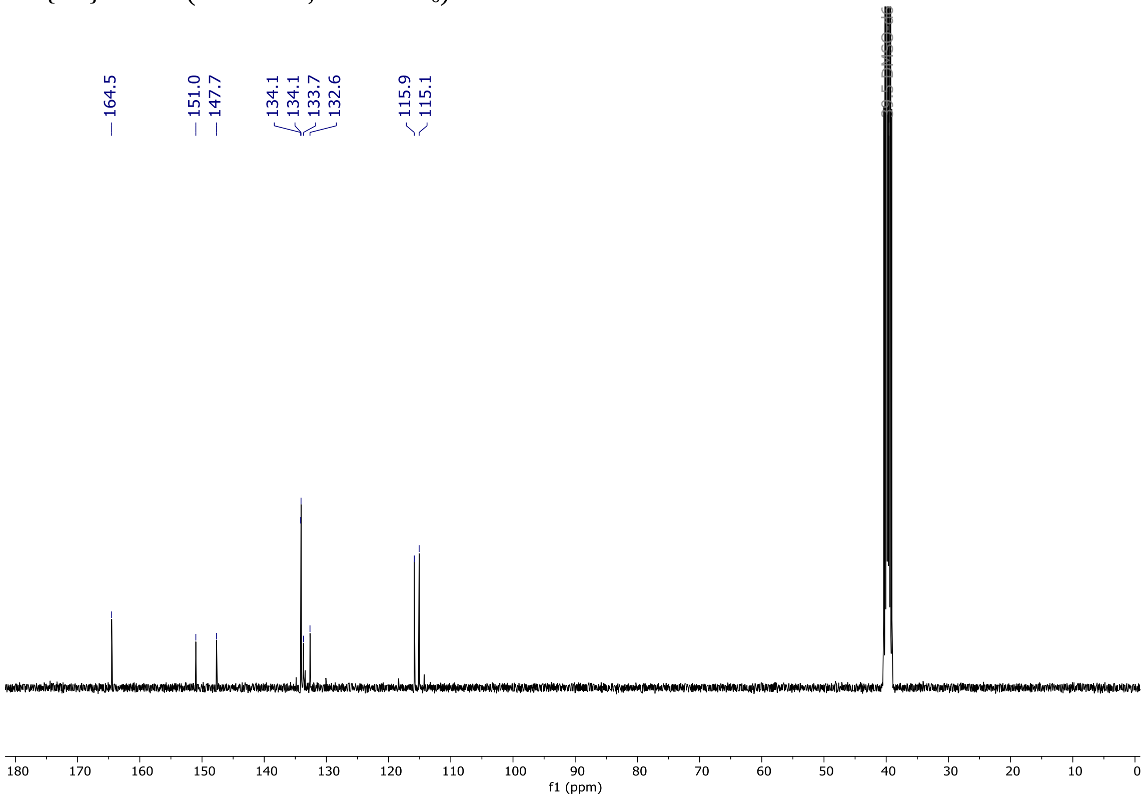

HMBC (DMSO-d_6)

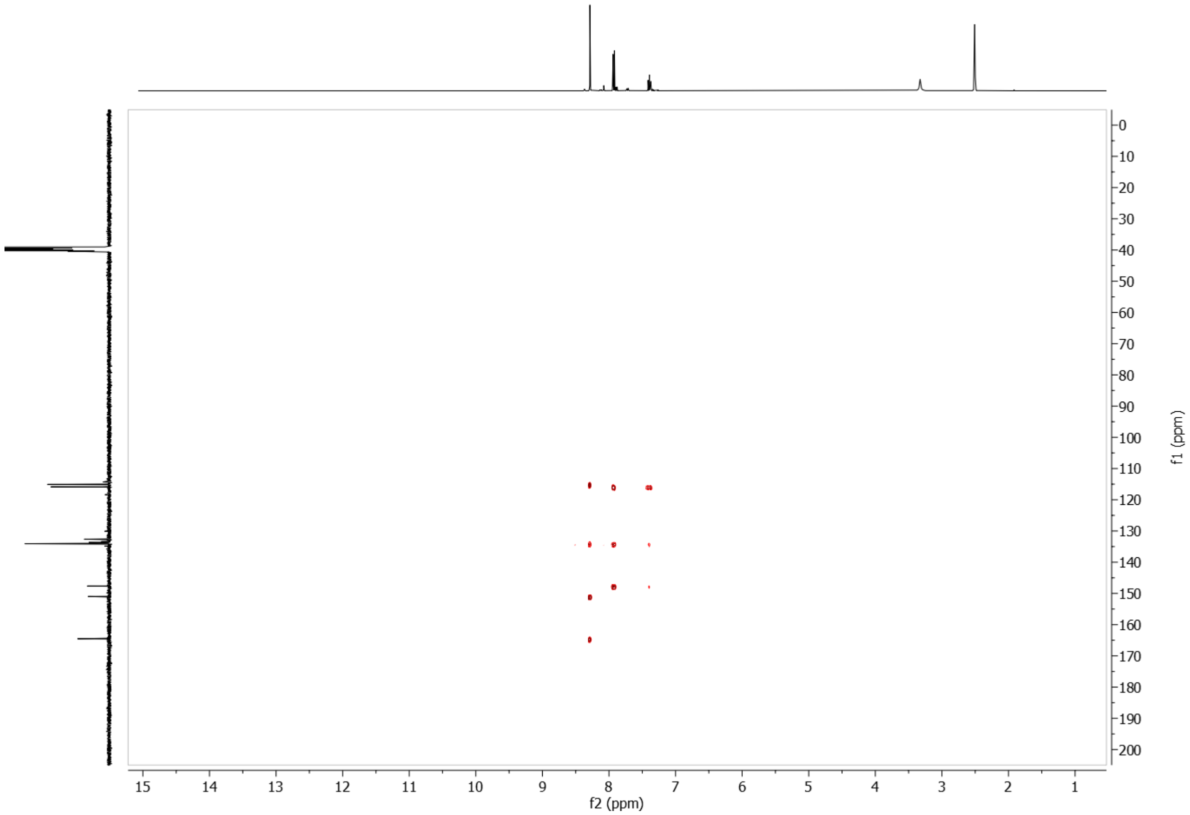

0
10
20
30
40
50
60
70
80
90
100
110
120
130
140
150
160
170
180
190
200
f1 (ppm)
15
14
13
12
11
10
9
8
7
6
5
4
3
2
1
f2 (ppm)

Dimethyl 3,3'-(diazene-1,2-diylbis(3,5-dibromo-4,1-phenylene))(*E*)-dipropionate (2k)

^{1}H-NMR (400 MHz, $CDCl_3$)

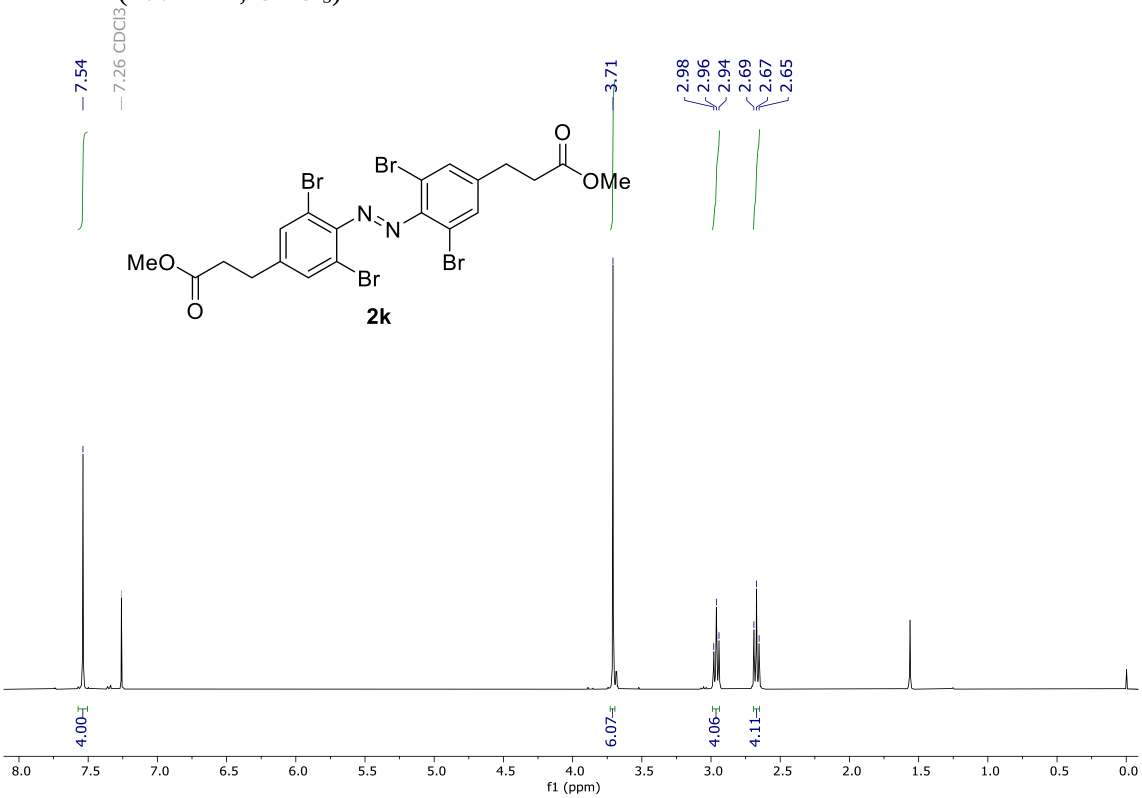

^{13}C{^{1}H}-NMR (100 MHz, $CDCl_3$)

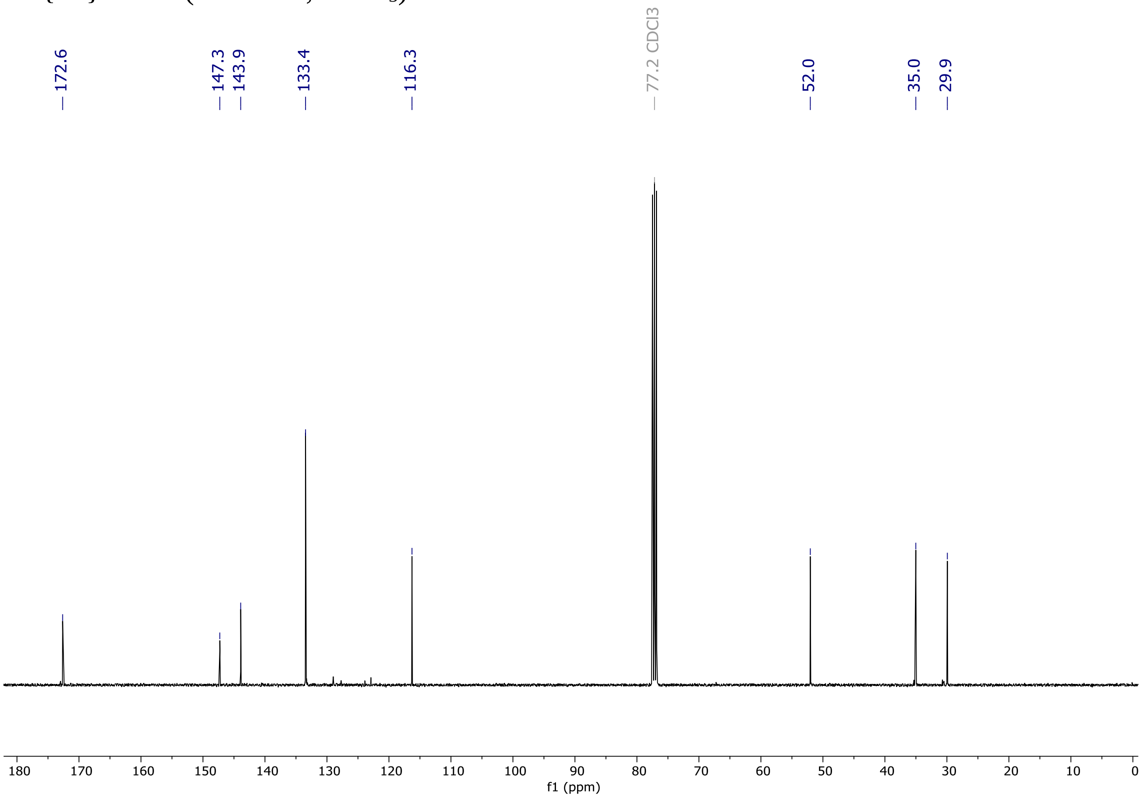

HSQC ($CDCl_3$)

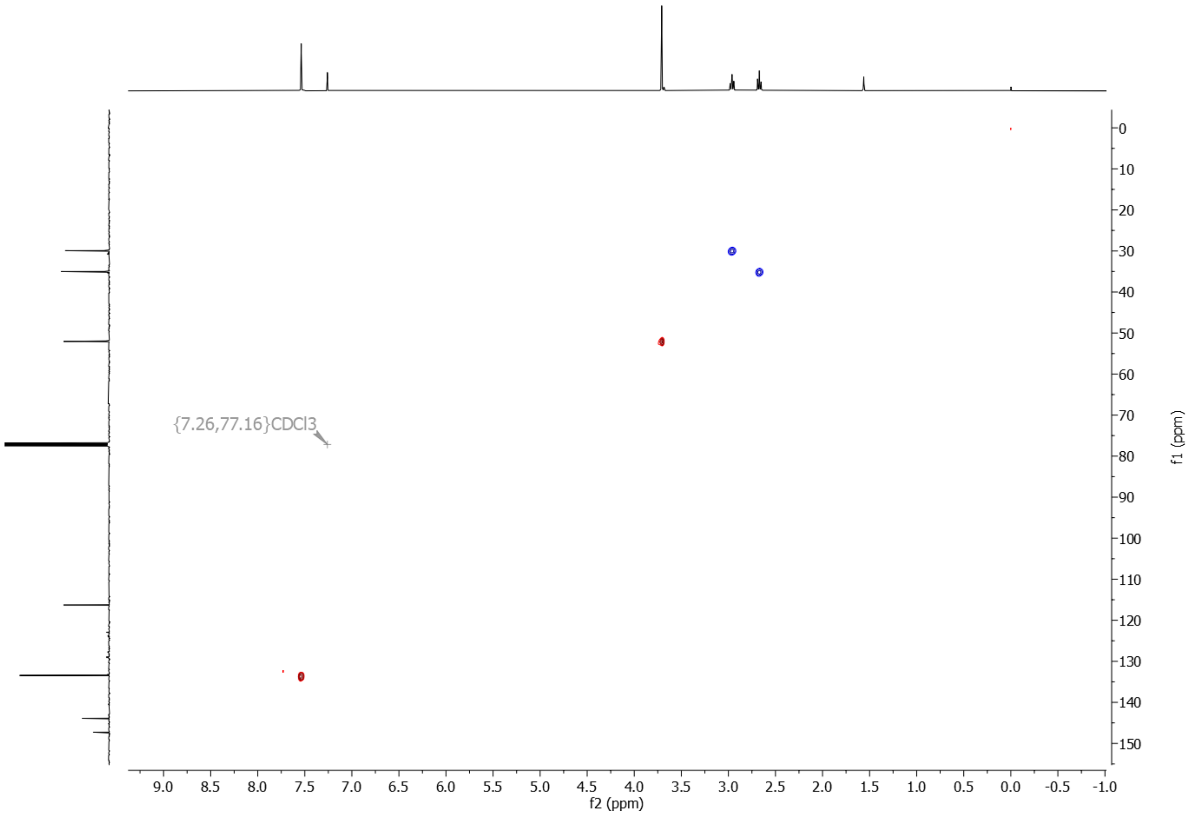

Methyl (*E*)-3-(3,5-dibromo-4-((2,6-dibromophenyl)diazenyl)phenyl)propanoate (2l)

^{1}H-NMR (400 MHz, $CDCl_3$)

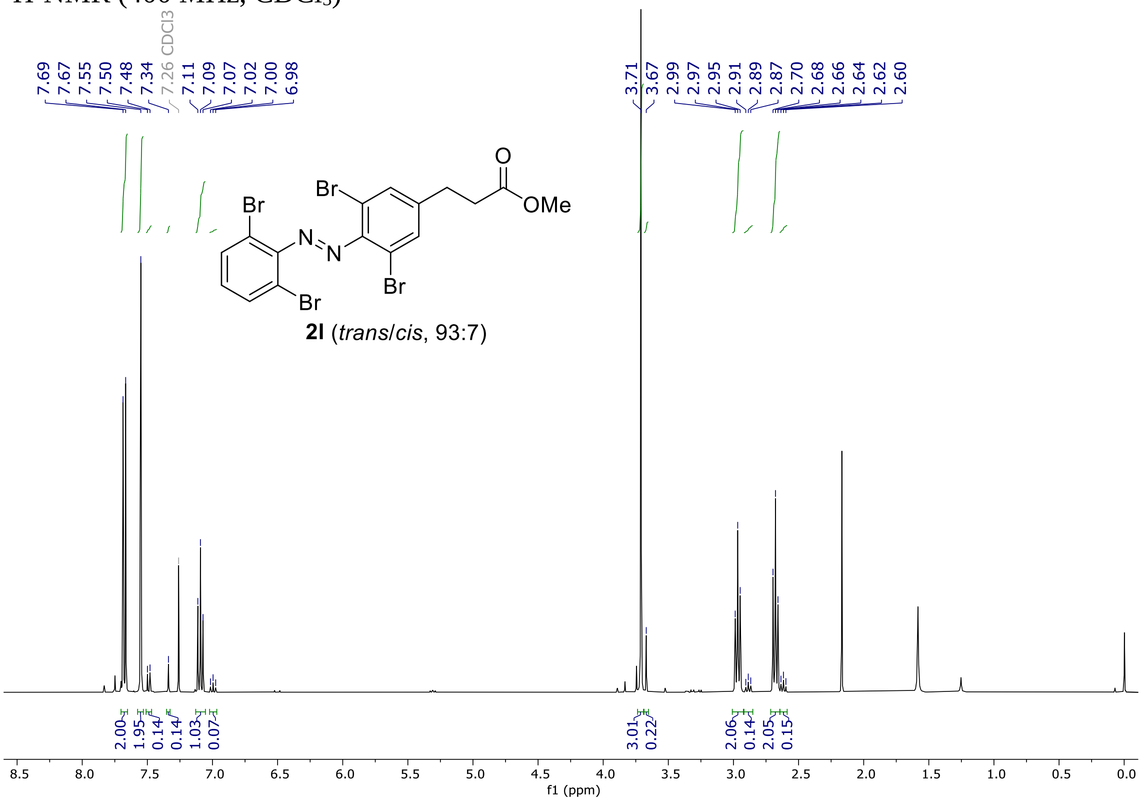

^{13}C{^{1}H}-NMR (100 MHz, $CDCl_3$)

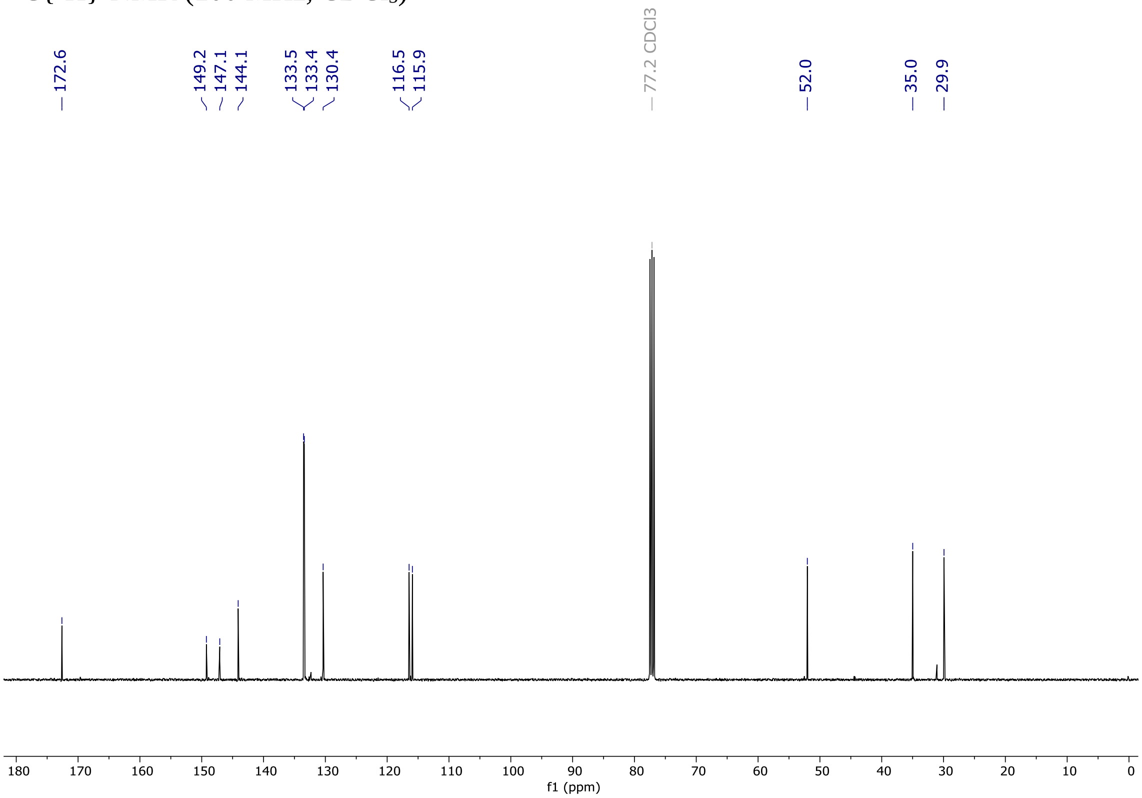

HSQC ($CDCl_3$)

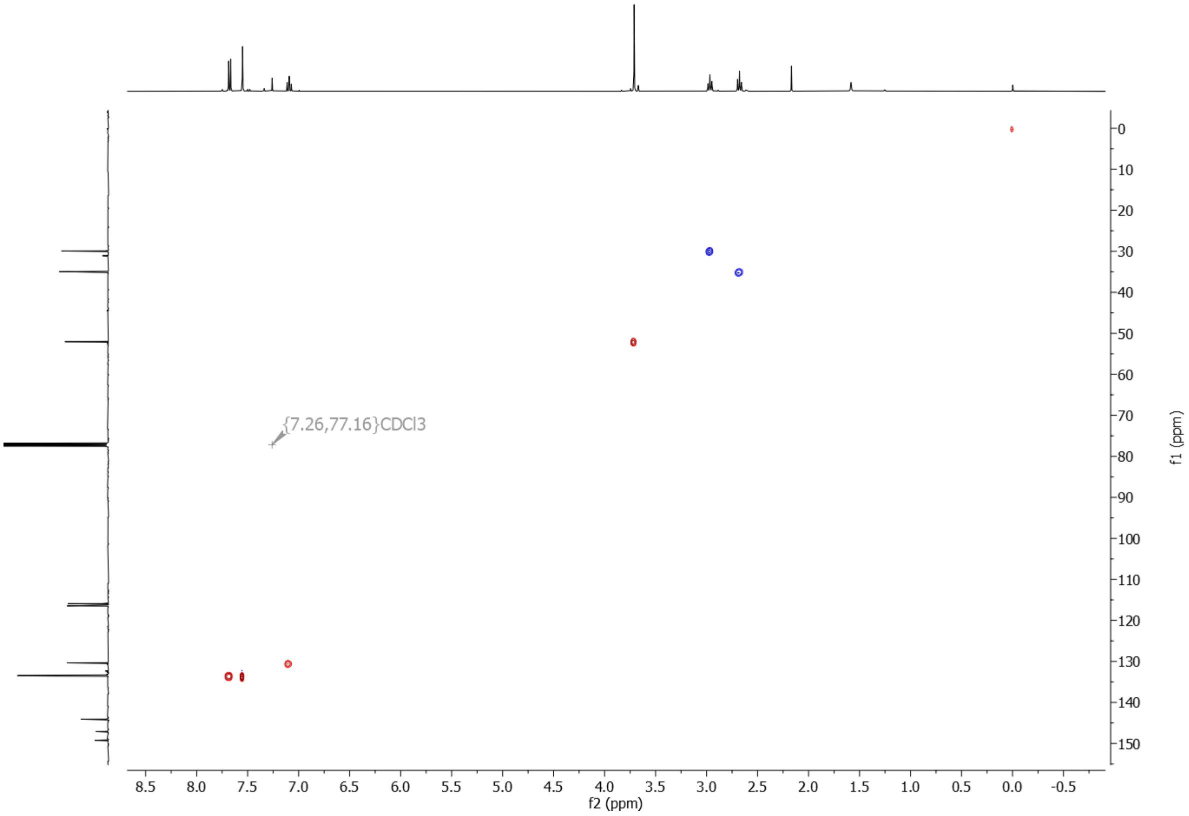

Methyl (*E*)-3-(3,5-dibromo-4-((2,6-dibromophenyl)diazenyl)benzoate (2m)

^{1}H-NMR (400 MHz, $CDCl_3$)

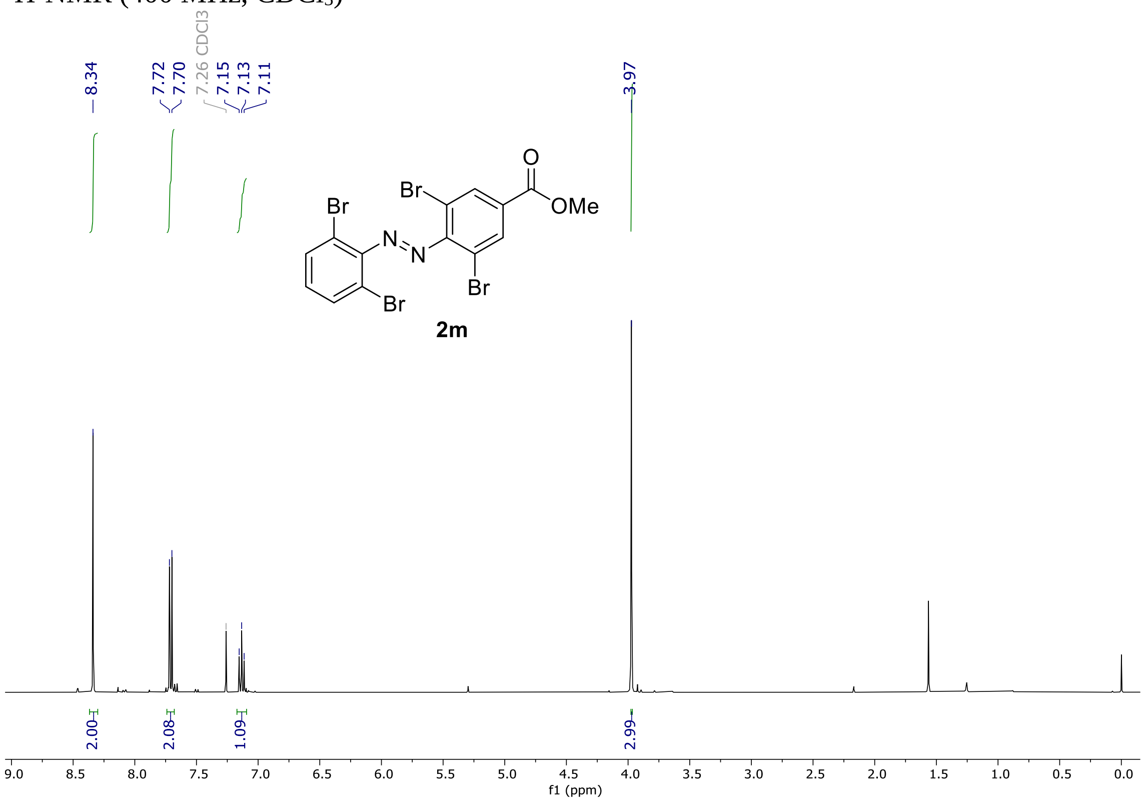

^{13}C{^{1}H}-NMR (100 MHz, $CDCl_3$)

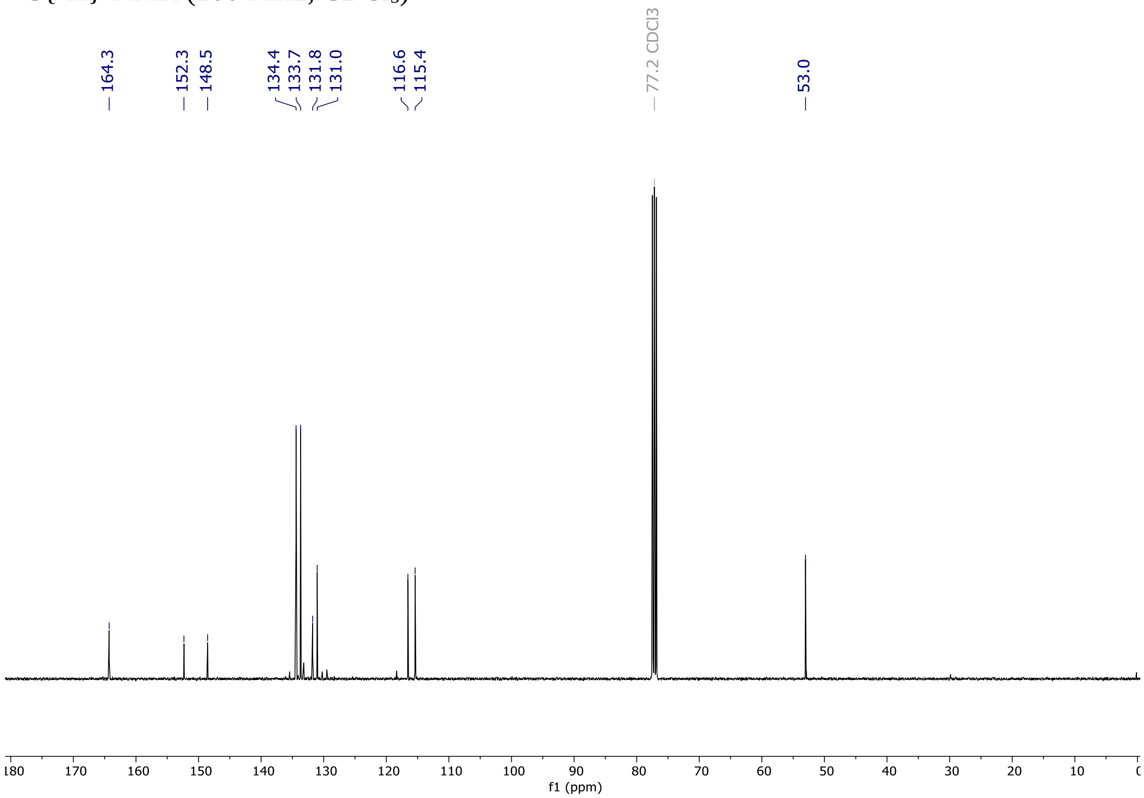

HSQC ($CDCl_3$)

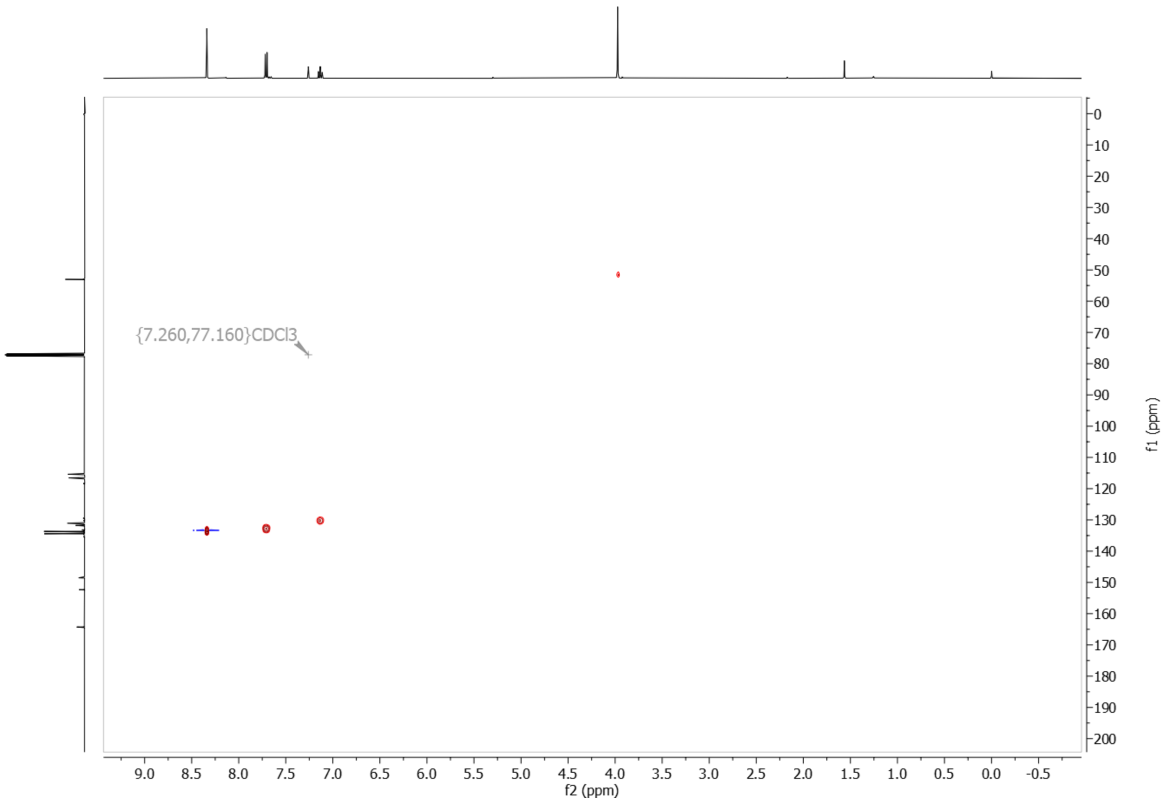

{7.260,77.160}CDCl3
0
10
20
30
40
50
60
70
80
90
100
110
120
130
140
150
160
170
180
190
200
f1 (ppm)
9.0
8.5
8.0
7.5
7.0
6.5
6.0
5.5
5.0
4.5
4.0
3.5
3.0
2.5
2.0
1.5
1.0
0.5
0.0
-0.5
f2 (ppm)

(*E*)-*N*-(3,5-Dibromo-4-((2,6-dibromophenyl)diazenyl)phenethyl)-2,2,2-trifluoroacetamide (2n)

^{1}H-NMR (400 MHz, $CDCl_3$)

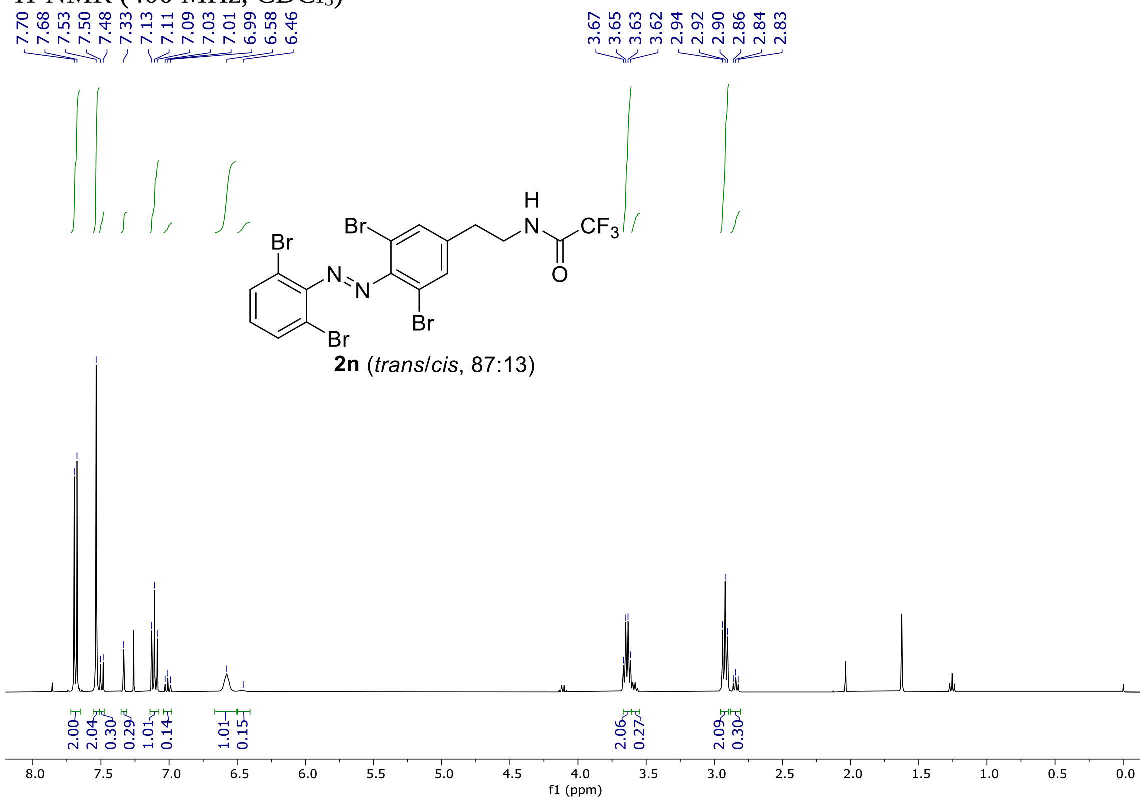

^{13}C{^{1}H}-NMR (100 MHz, $CDCl_3$)

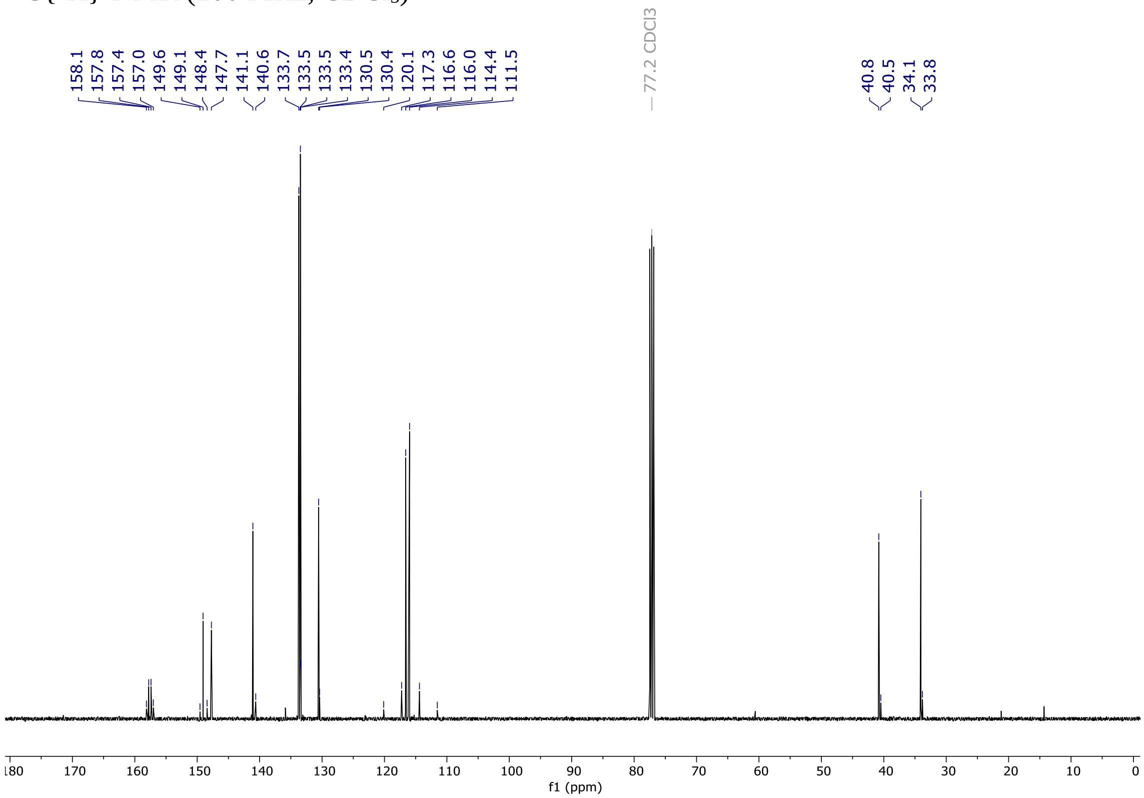

^{19}F-NMR (376 MHz, $CDCl_3$)

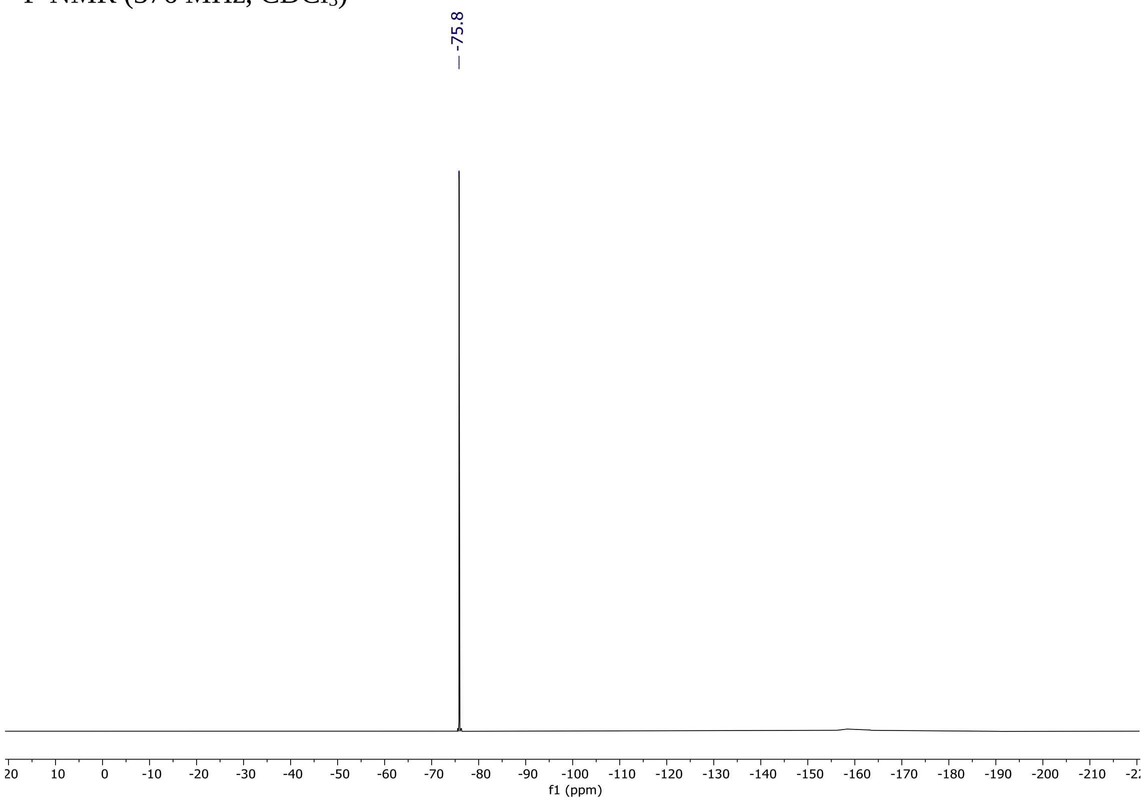

HSQC ($CDCl_3$)

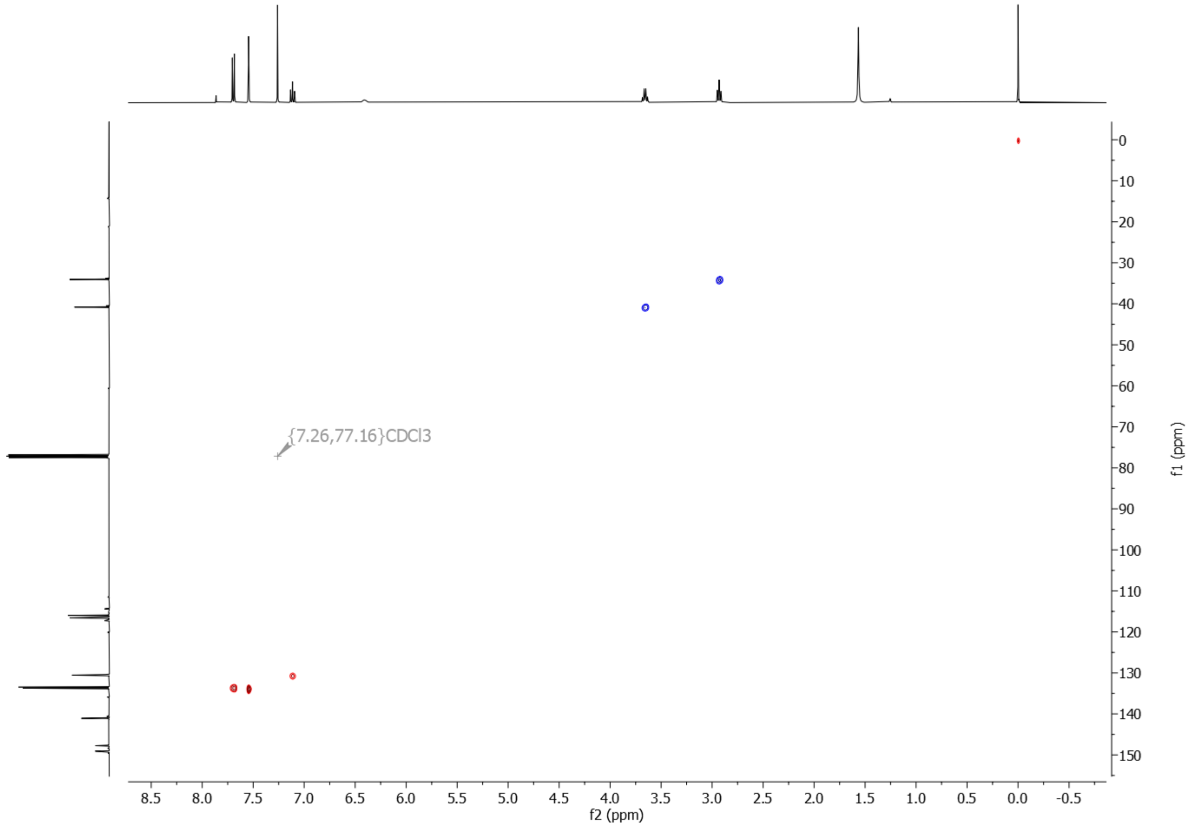

(*E*)-*N*-(3,5-Dibromo-4-((2,6-dibromophenyl)diazenyl)benzyl)-2,2,2-trifluoroacetamide (2o)

^{1}H-NMR (400 MHz, $CDCl_3$)

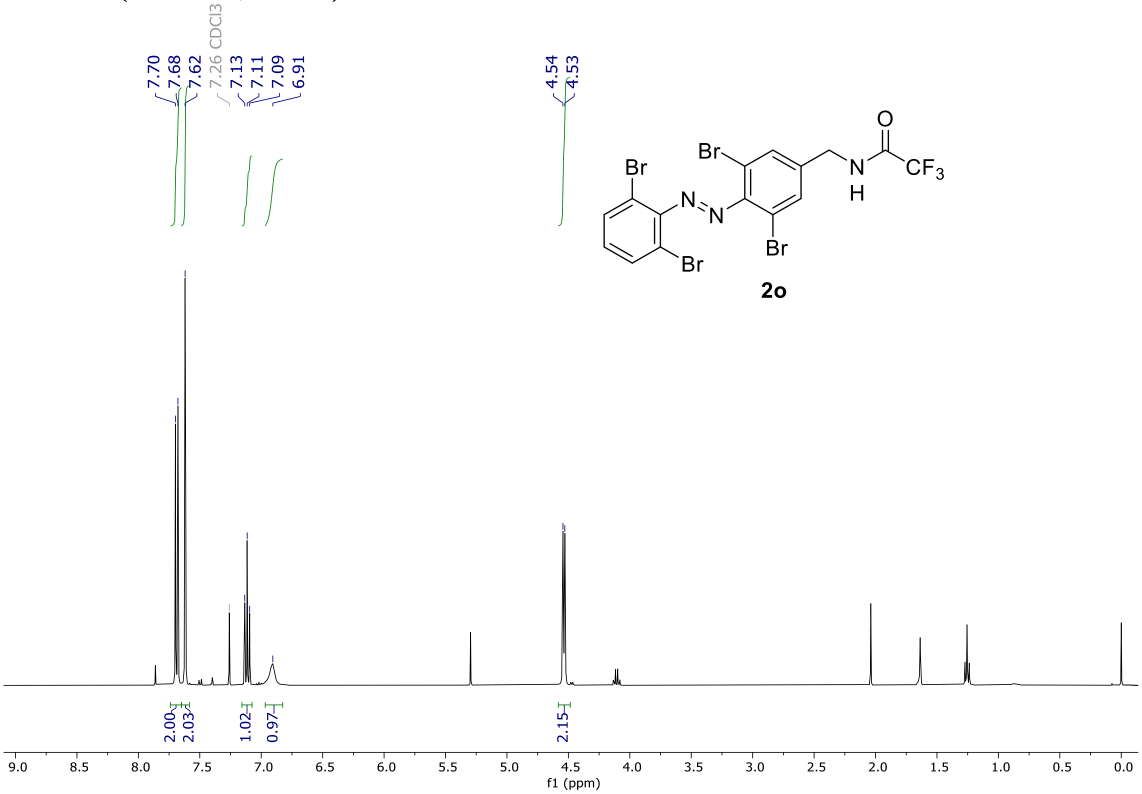

^{13}C{^{1}H}-NMR (100 MHz, $CDCl_3$)

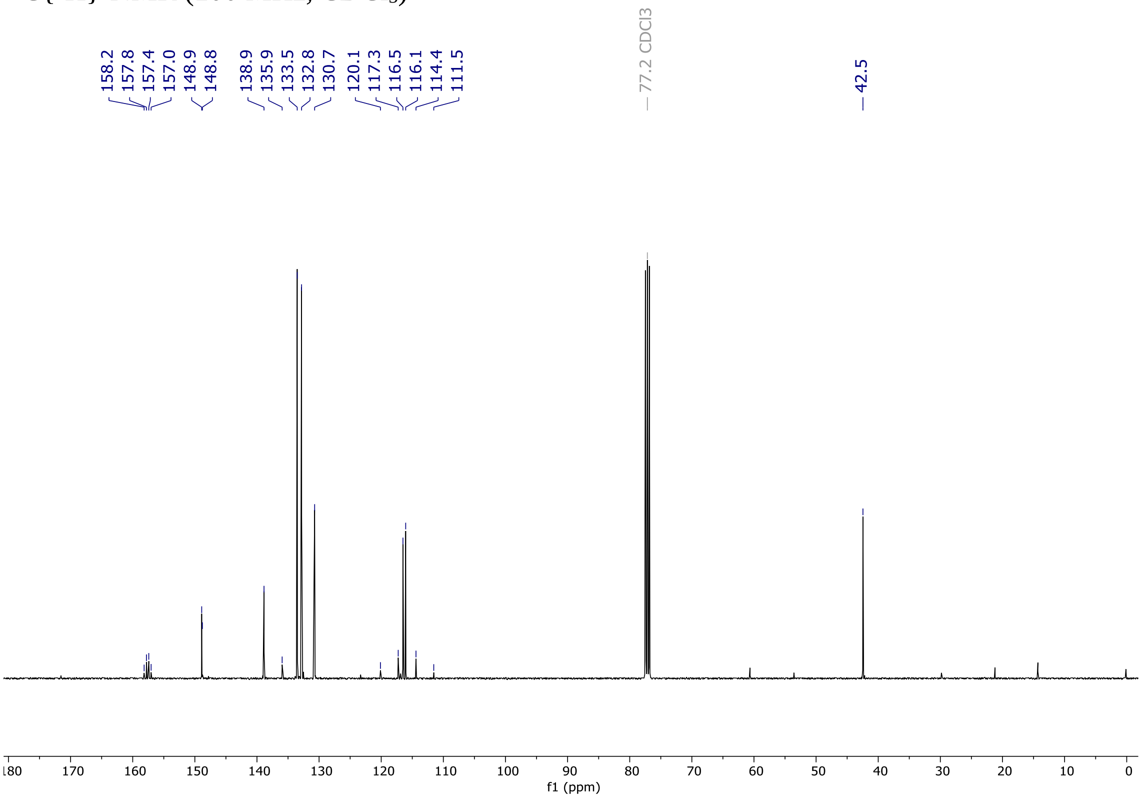

^{19}F-NMR (376 MHz, $CDCl_3$)

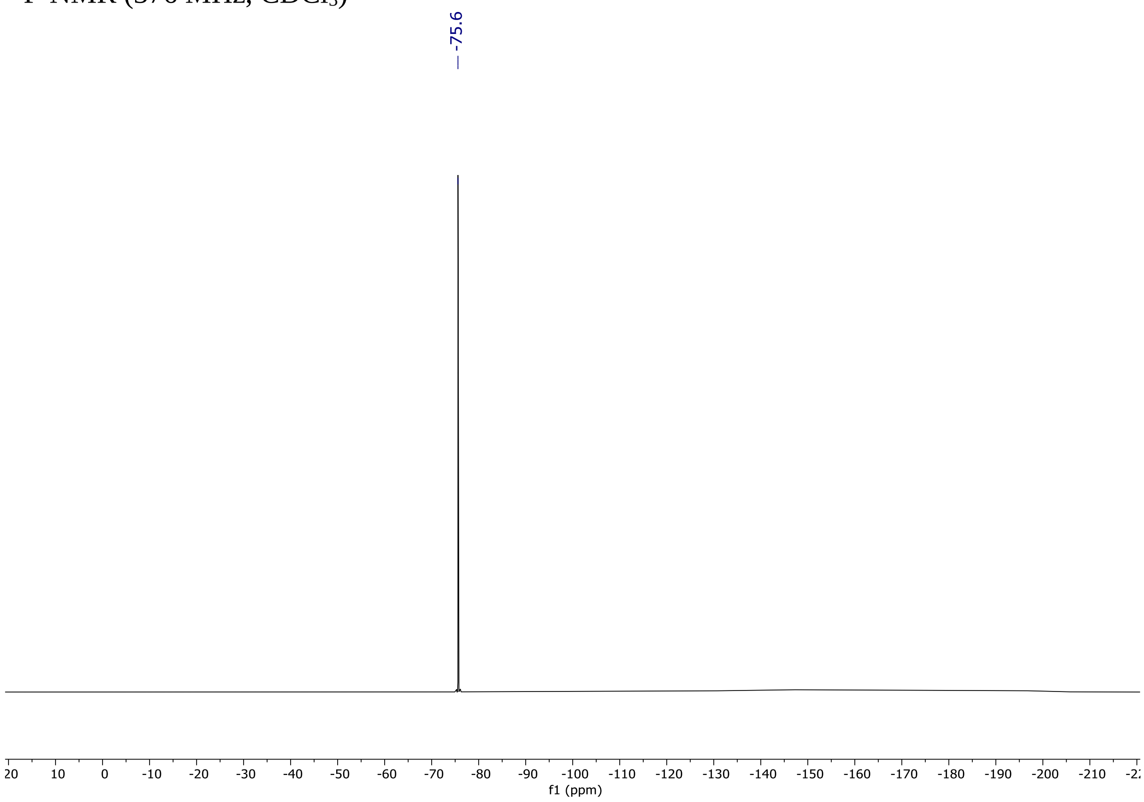

HSQC ($CDCl_3$)

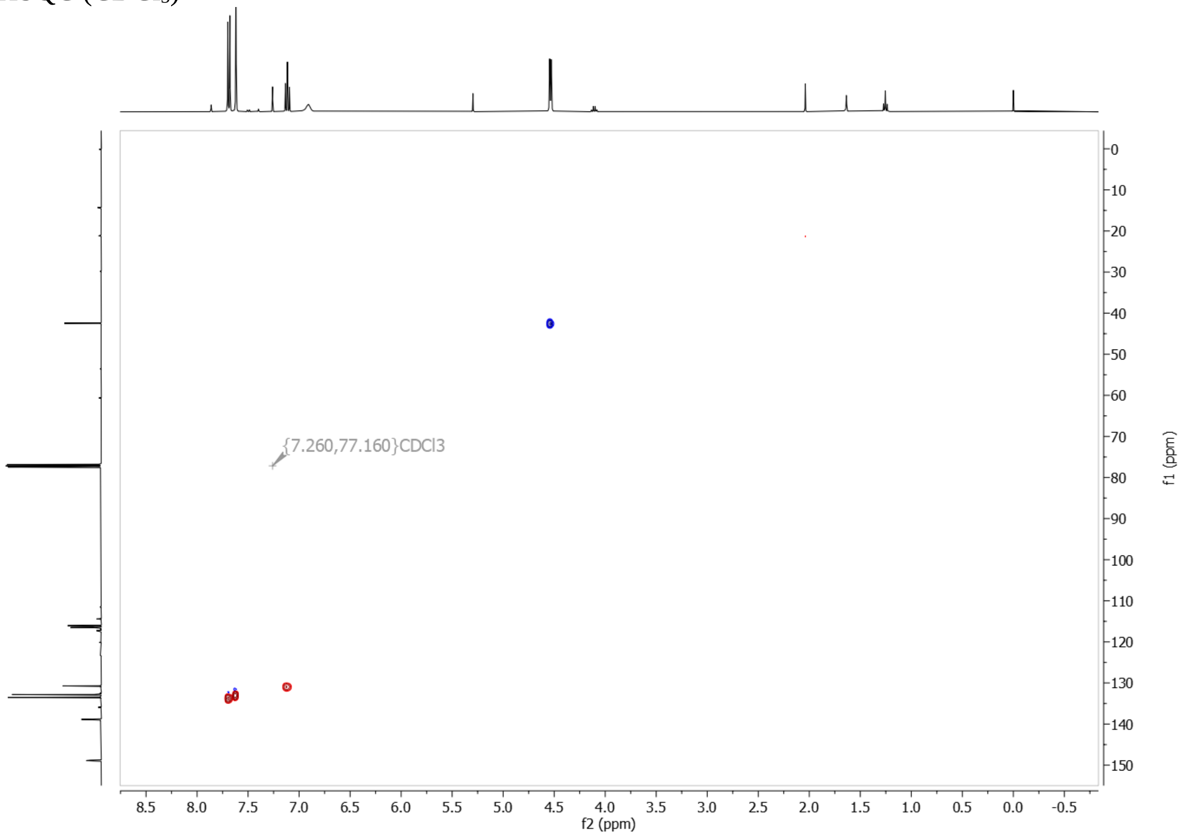

Methyl (*E*)-3-(3,5-Dibromo-4-((2,6-dibromo-4-(2-(2,2,2-trifluoroacetamido)ethyl)phenyl)diazenyl)phenyl)propanoate (2p)

^{1}H-NMR (400 MHz, $CDCl_3$)

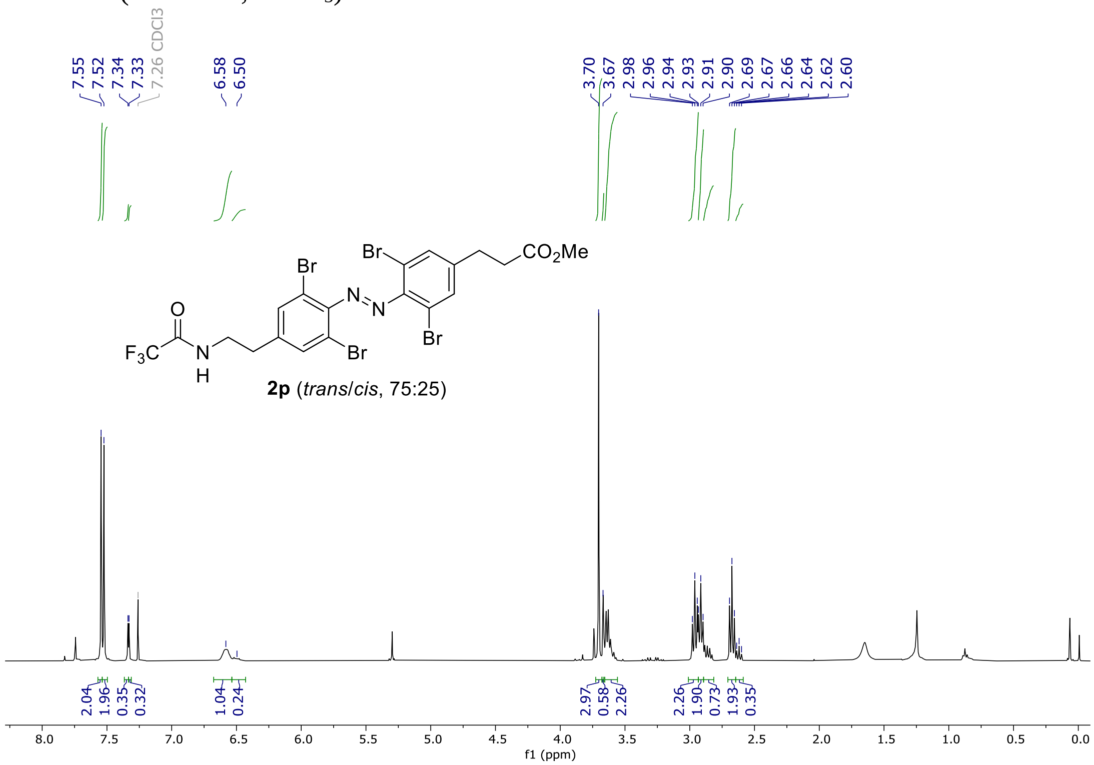

^{13}C{^{1}H}-NMR (100 MHz, $CDCl_3$)

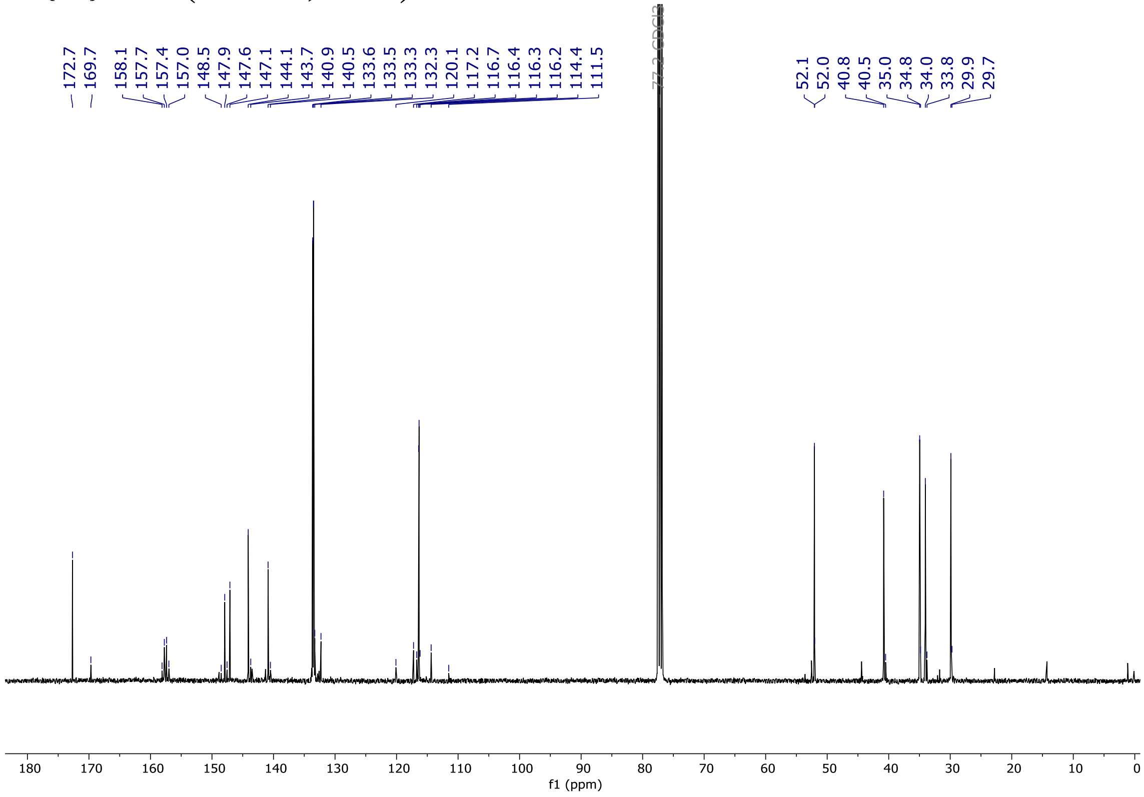

^{19}F-NMR (376 MHz, $CDCl_3$)

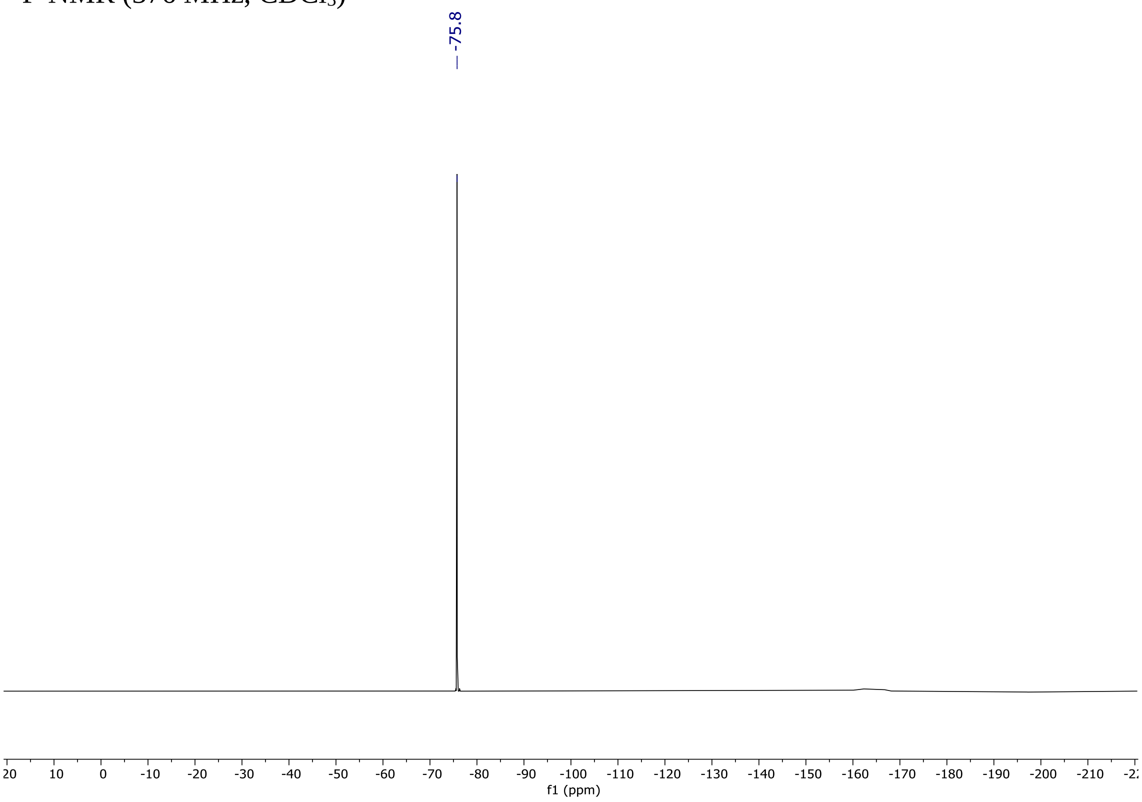

HSQC ($CDCl_3$)

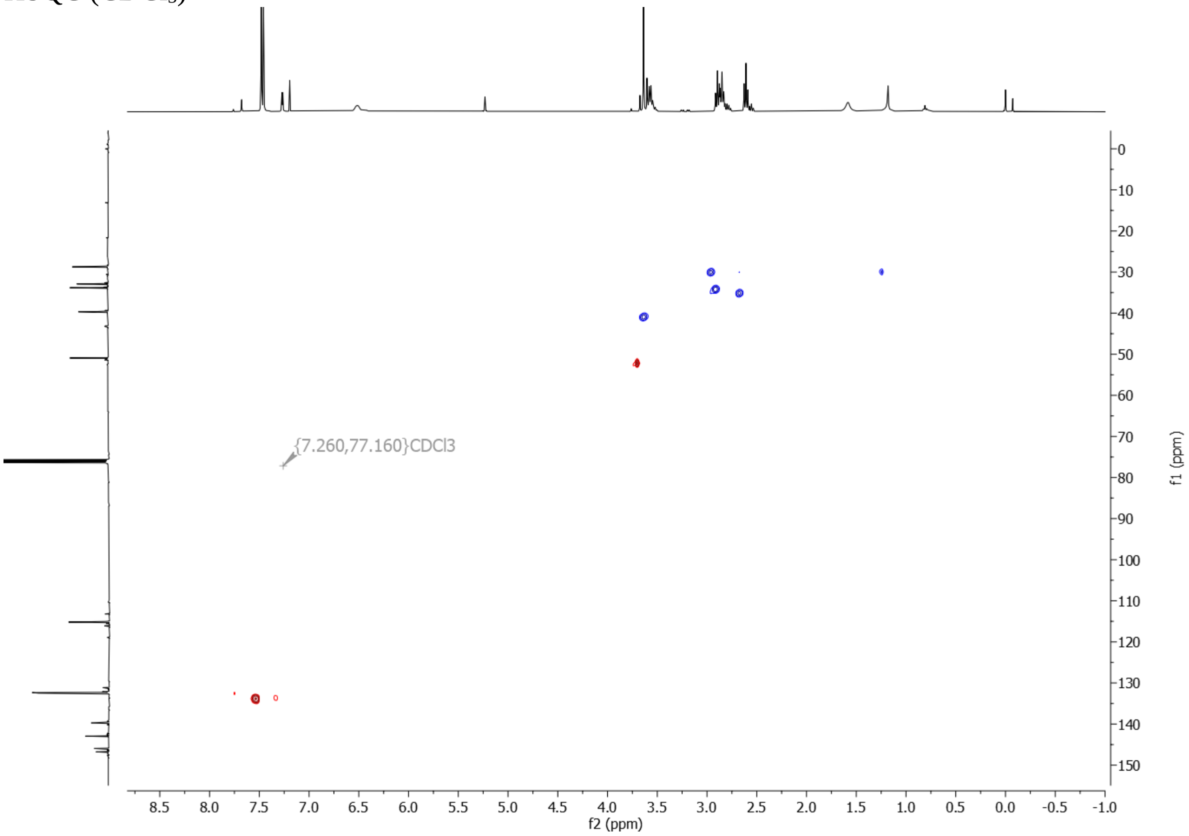

(*E*)-1,2-Bis(2,6-dimethoxyphenyl)diazene (3a)

^{1}H-NMR (400 MHz, $CDCl_3$)

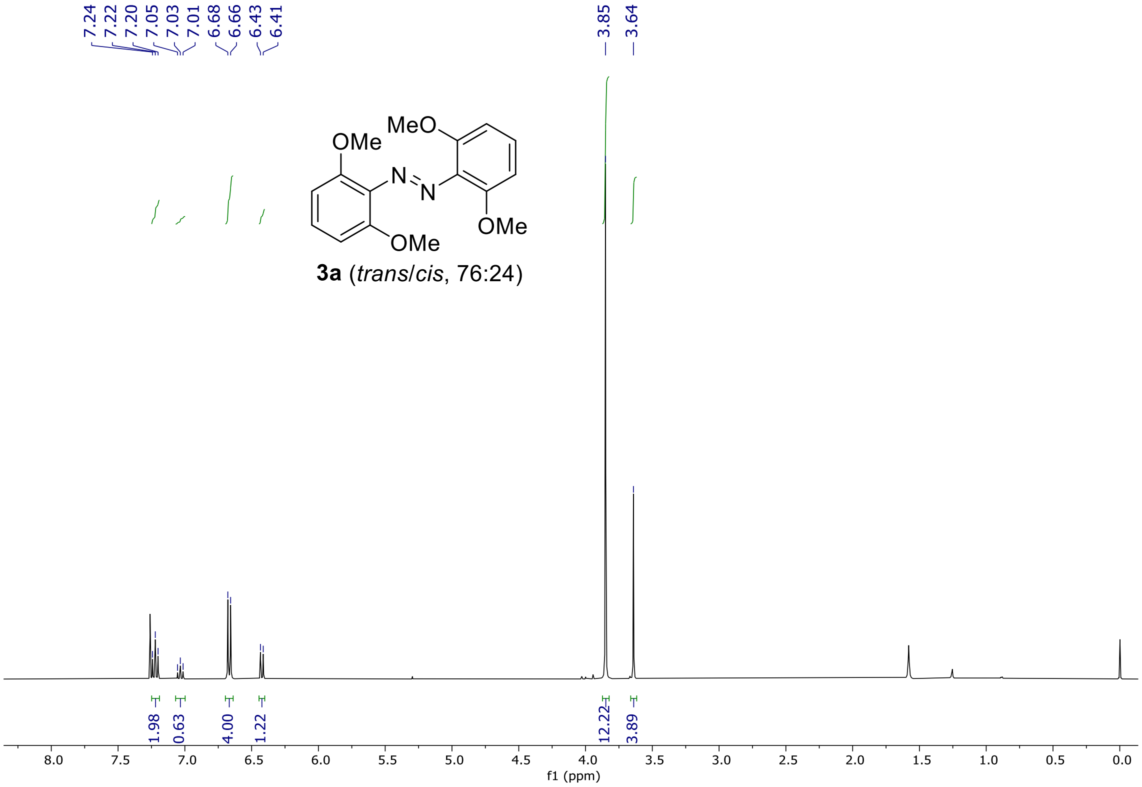

^{13}C{^{1}H}-NMR (100 MHz, $CDCl_3$)

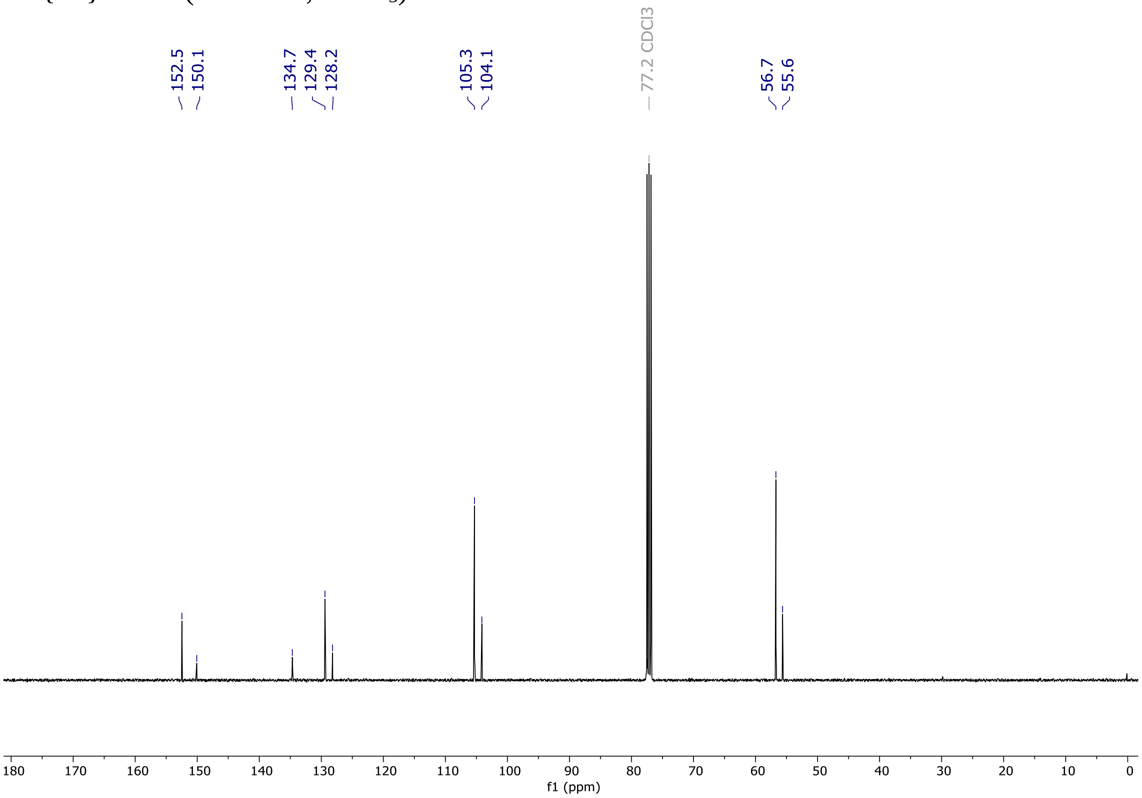

HSQC ($CDCl_3$)

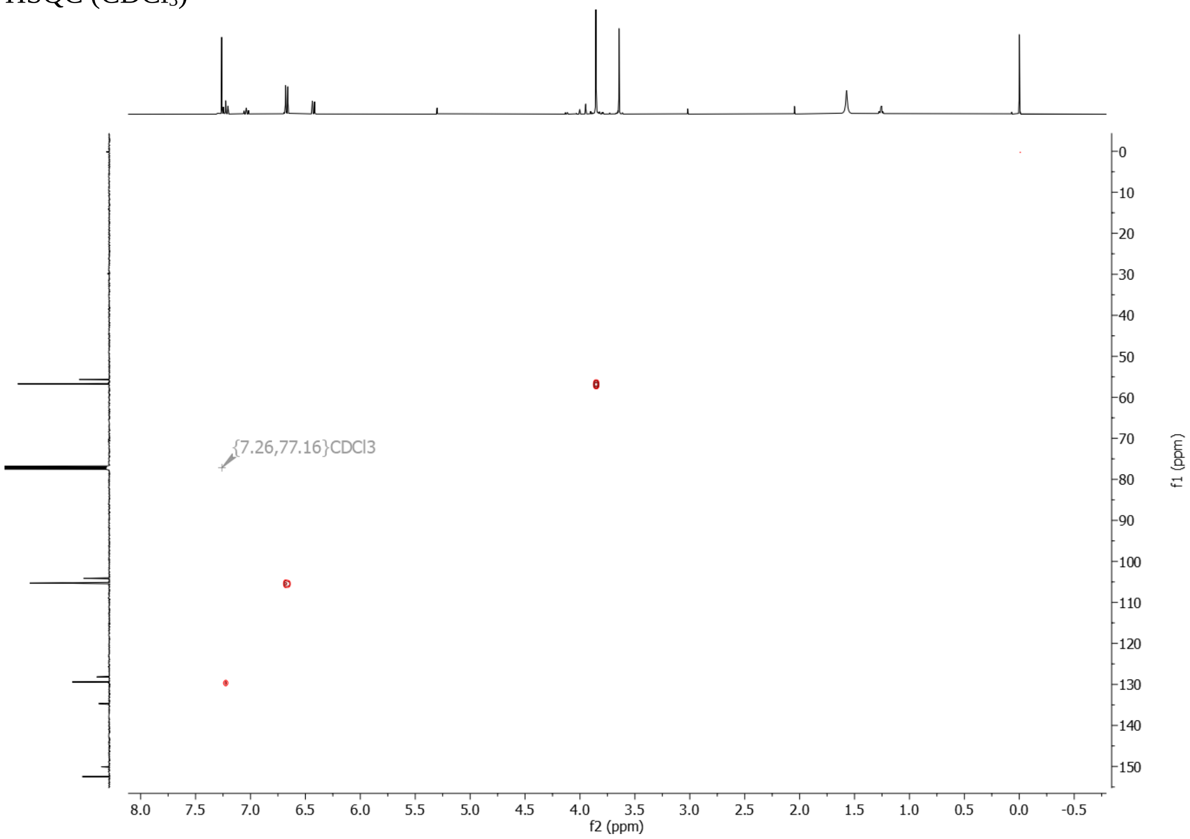

(*E*)-1-(2,6-Dimethoxy-4-methylphenyl)-2-(2,6-dimethoxyphenyl)diazene (3b)

^{1}H-NMR (400 MHz, $CDCl_3$)

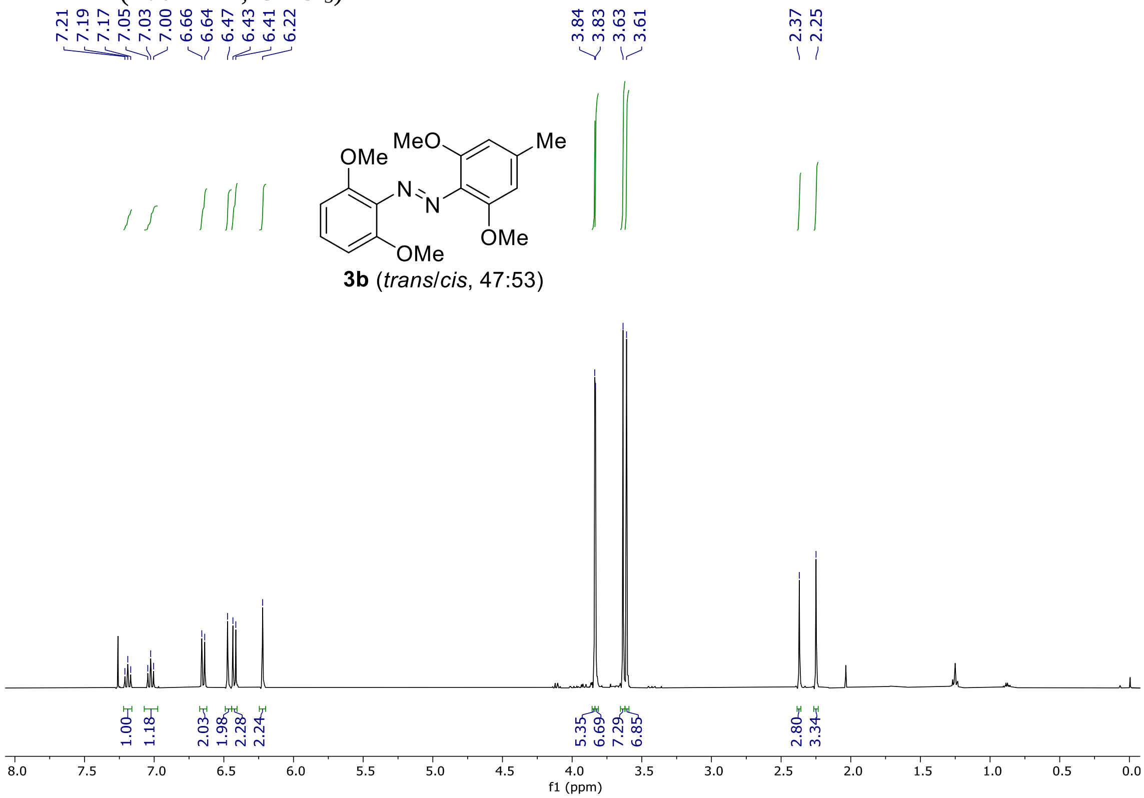

^{13}C{^{1}H}-NMR (100 MHz, $CDCl_3$)

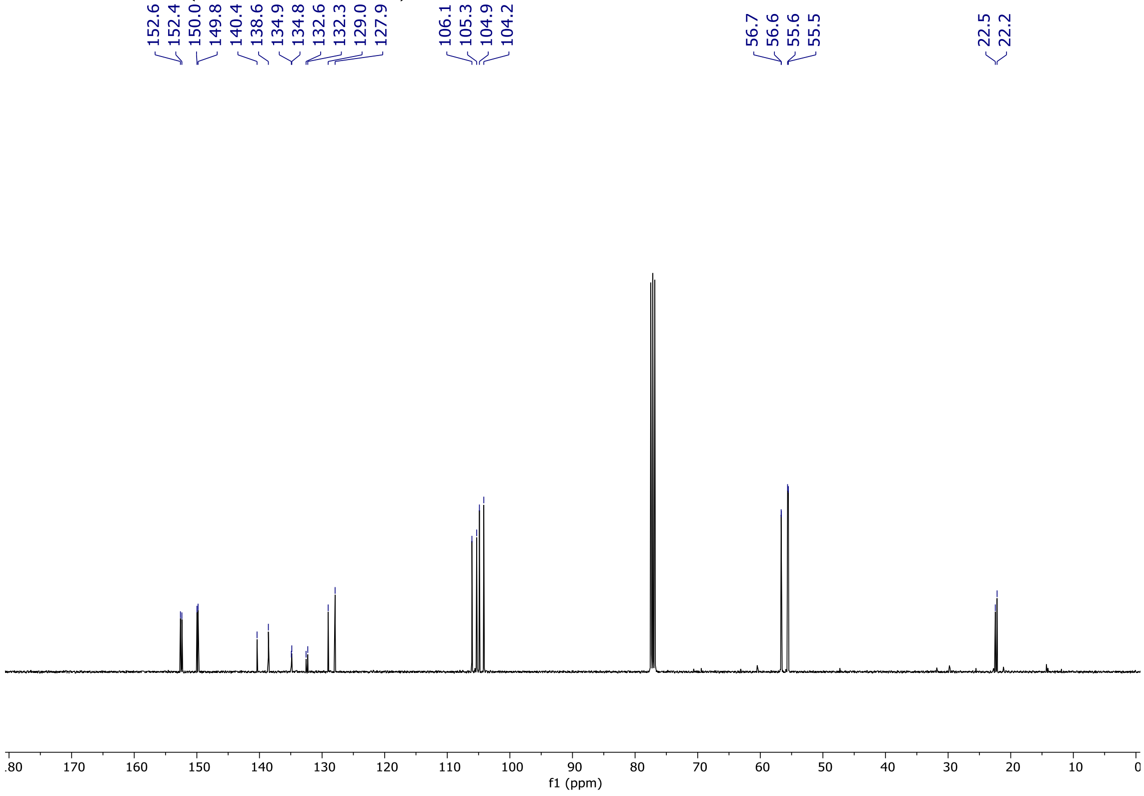

HSQC ($CDCl_3$)

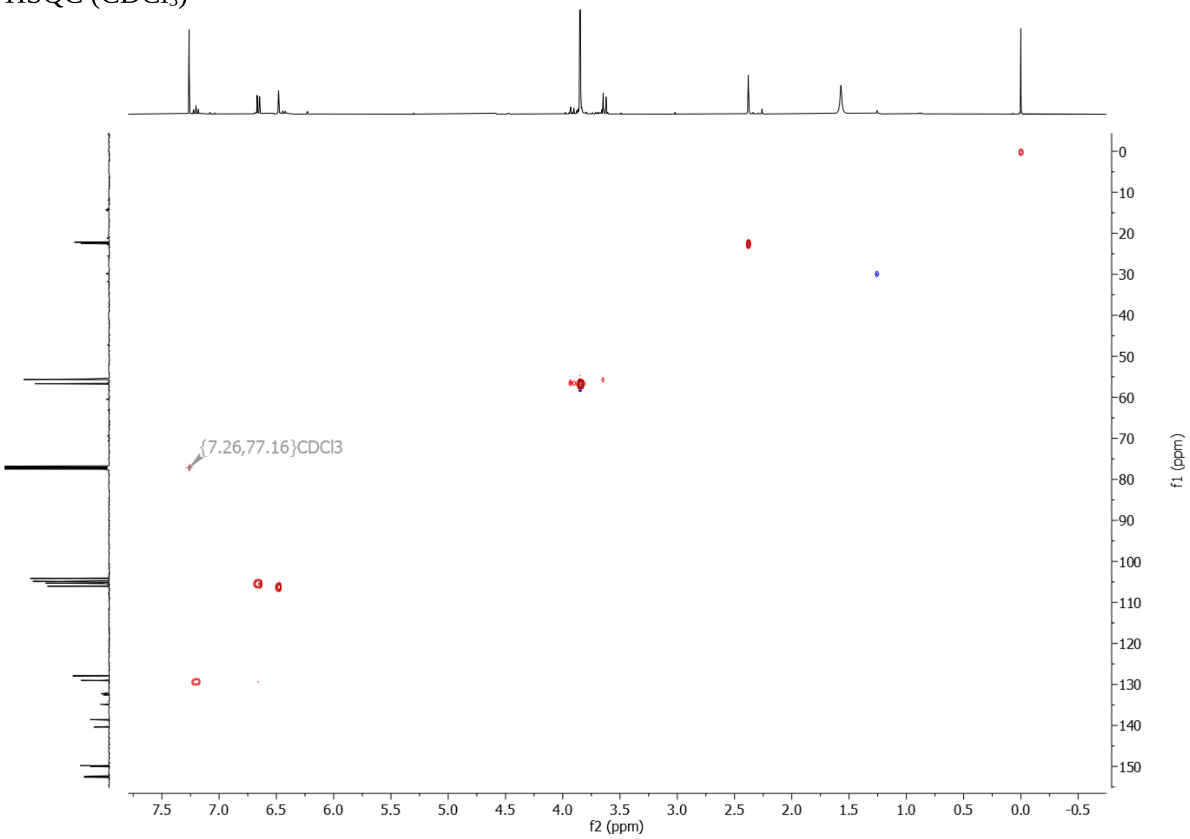

(*E*)-1,2-Bis(2,6-dimethoxyphenyl-4-methylphenyl)diazene (3c)

^{1}H-NMR (400 MHz, $CDCl_3$)

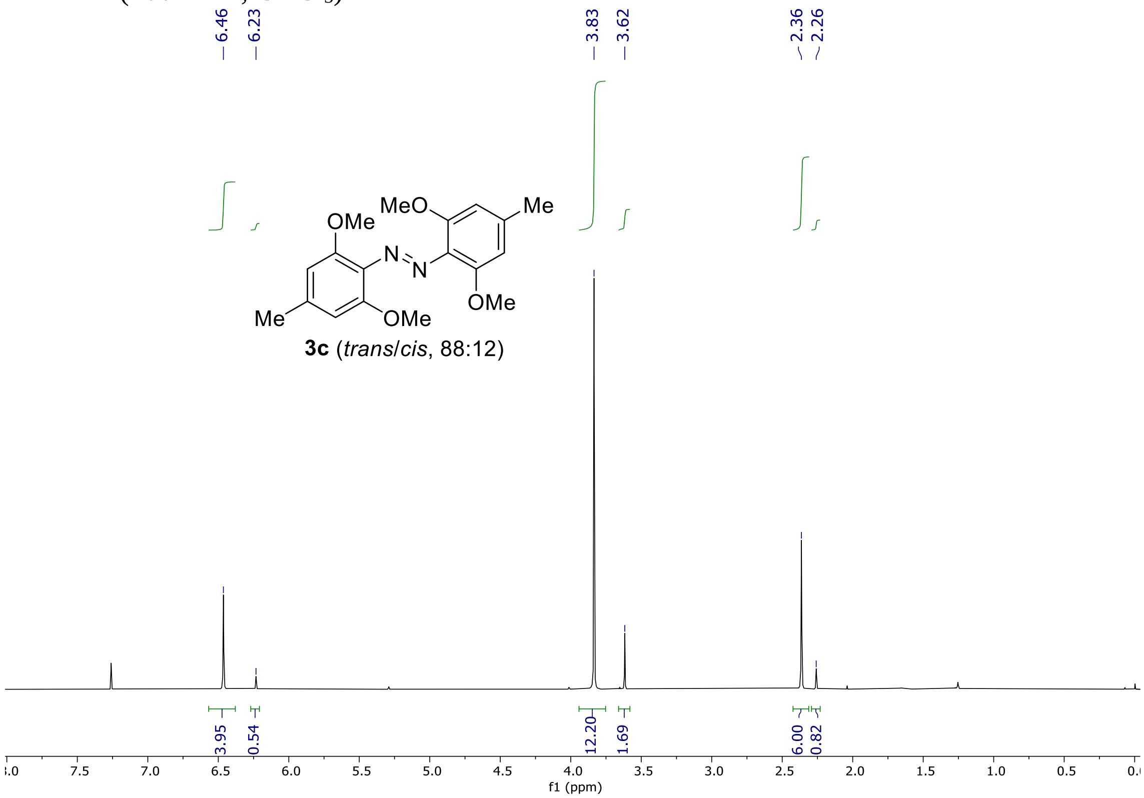

^{13}C{^{1}H}-NMR (100 MHz, $CDCl_3$)

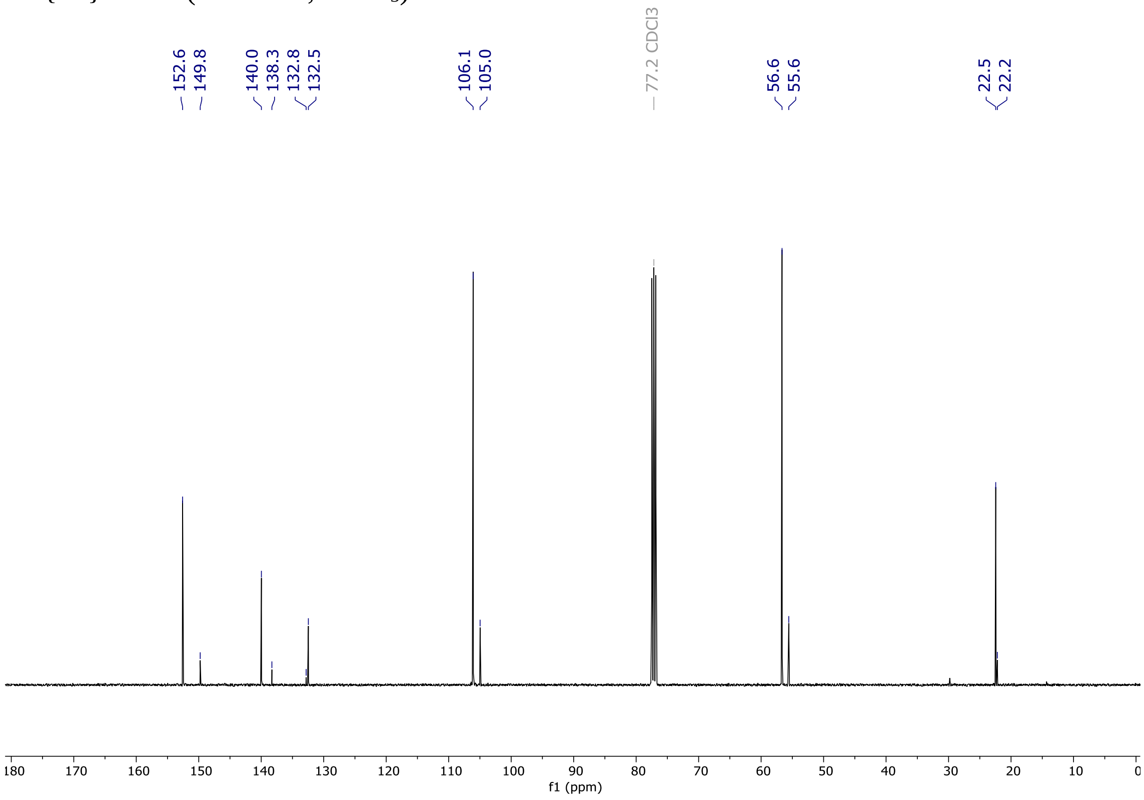

HSQC ($CDCl_3$)

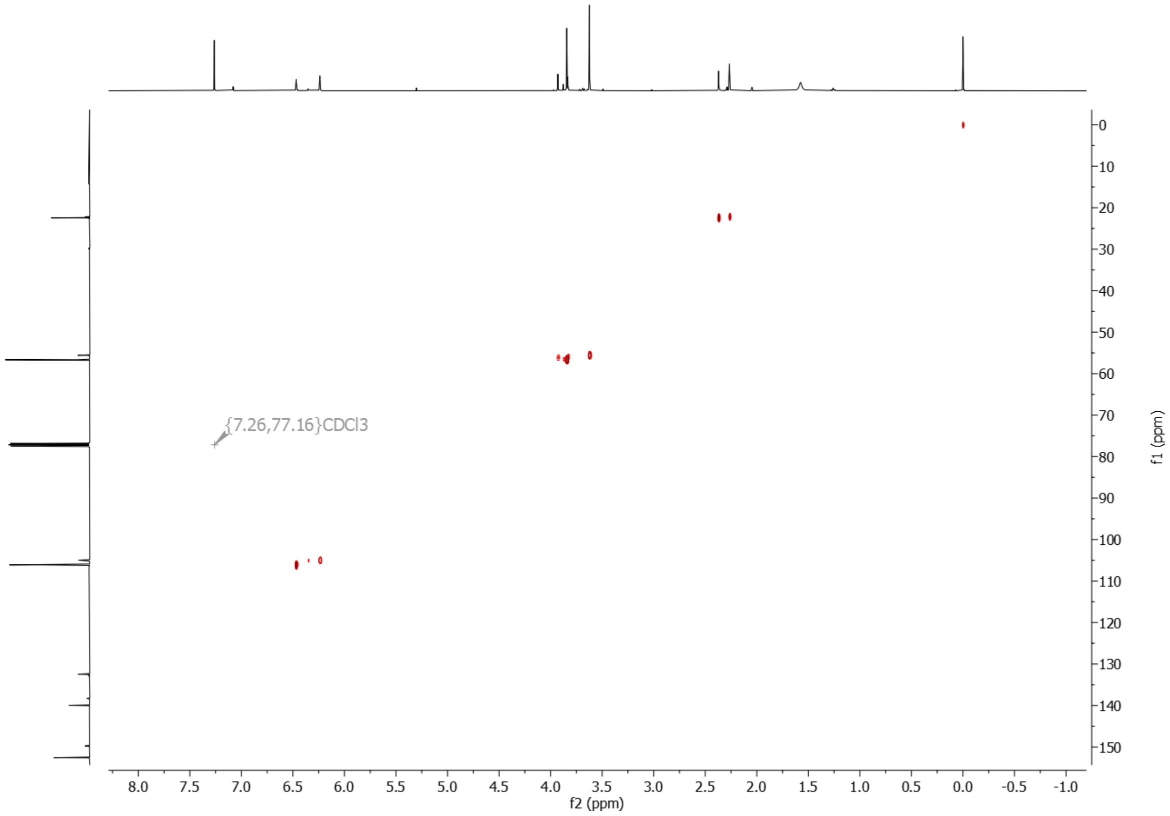

{7.26,77.16}CDCl3
8.0
7.5
7.0
6.5
6.0
5.5
5.0
4.5
4.0
3.5
3.0
2.5
2.0
1.5
1.0
0.5
0.0
-0.5
-1.0
f2 (ppm)
0
10
20
30
40
50
60
70
80
90
100
110
120
130
140
150
f1 (ppm)

(*E*)-1-(2,6-Dimethoxy-4-methoxyphenyl)-2-(2,6-dimethoxyphenyl)diazene (3d)

^{1}H-NMR (400 MHz, $CDCl_3$)

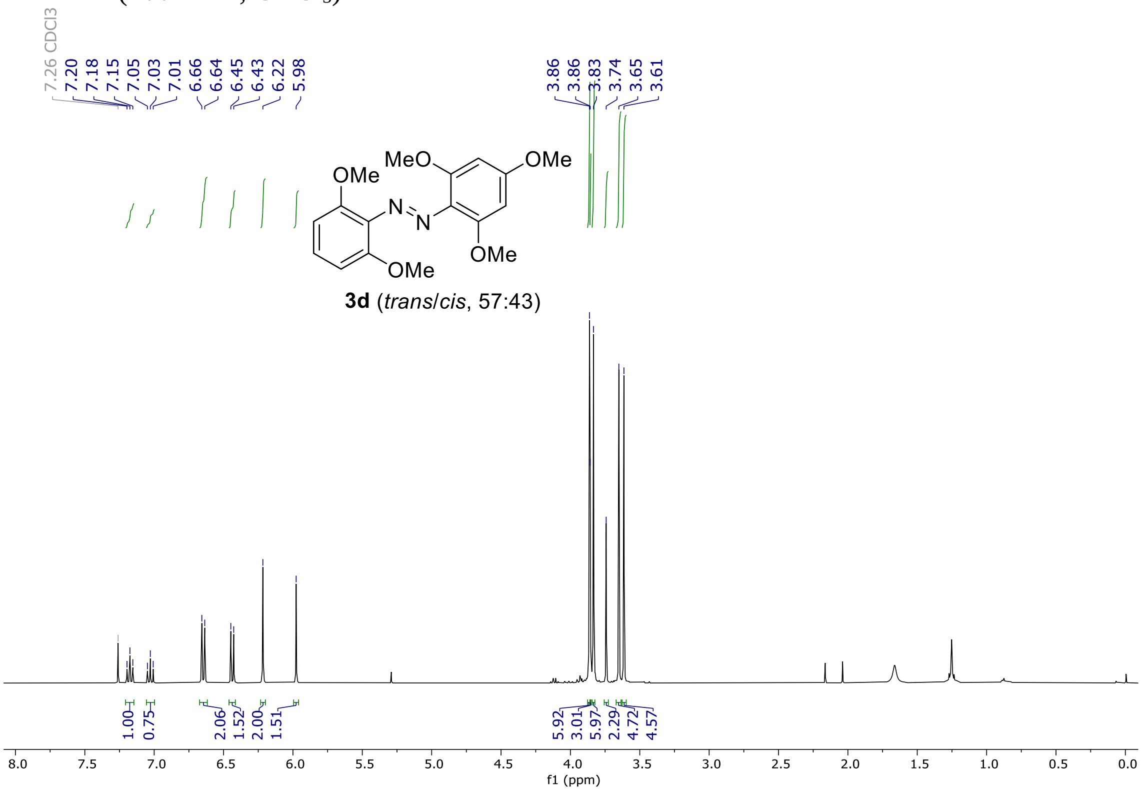

^{13}C{^{1}H}-NMR (100 MHz, $CDCl_3$)

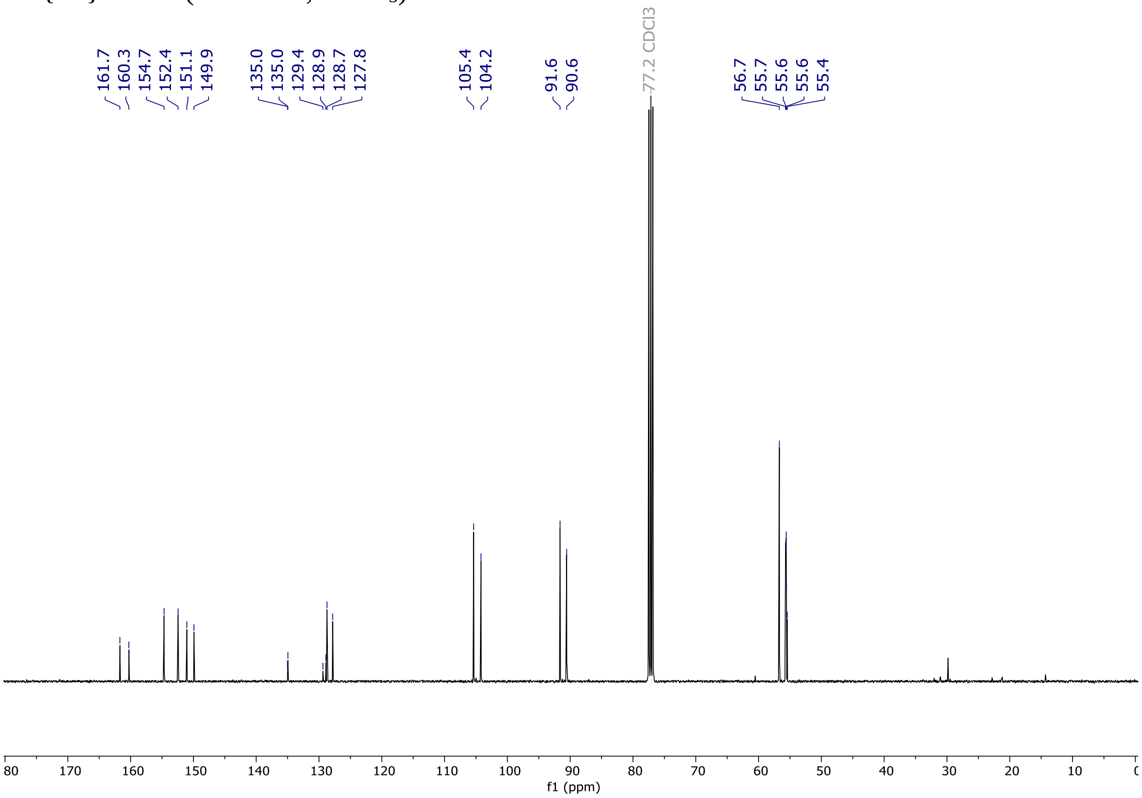

HSQC ($CDCl_3$)

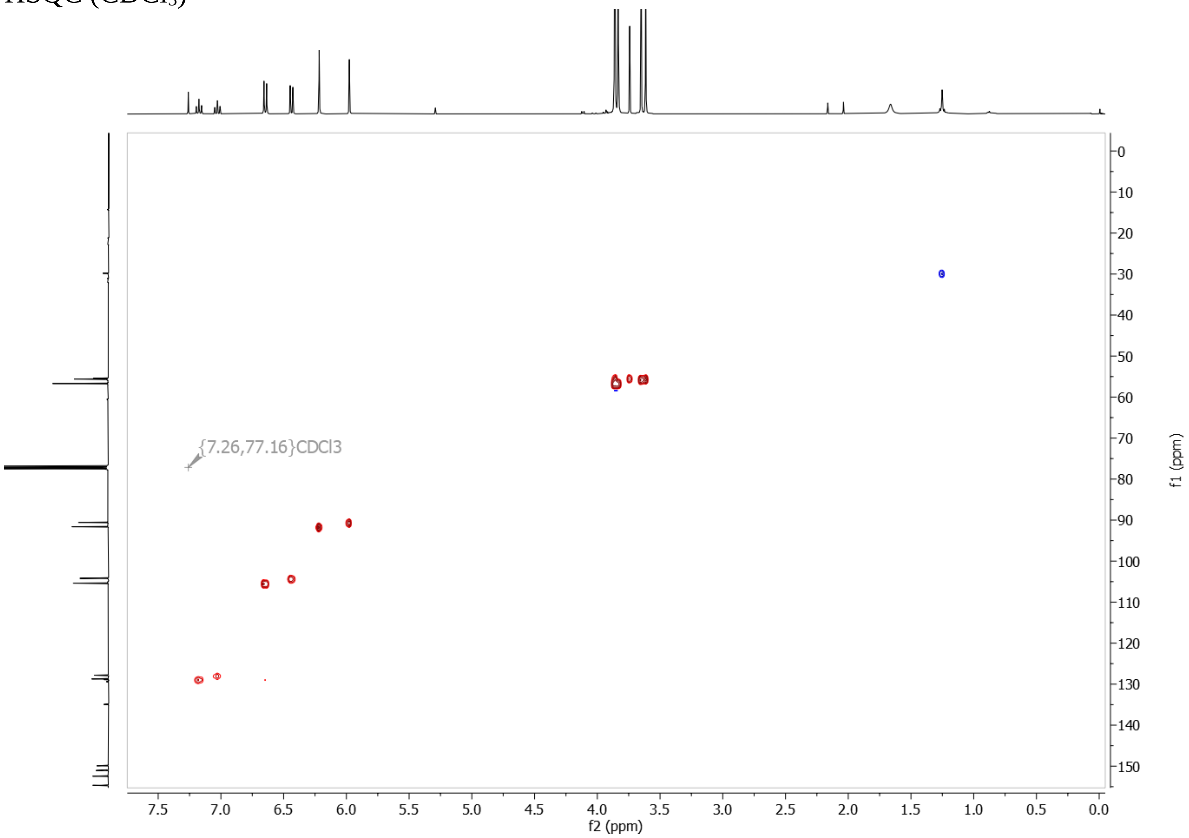

(*E*)-1,2-Bis(2,6-dimethoxyphenyl-4-chlorophenyl)diazene (3e)

^{1}H-NMR (400 MHz, $CDCl_3$)

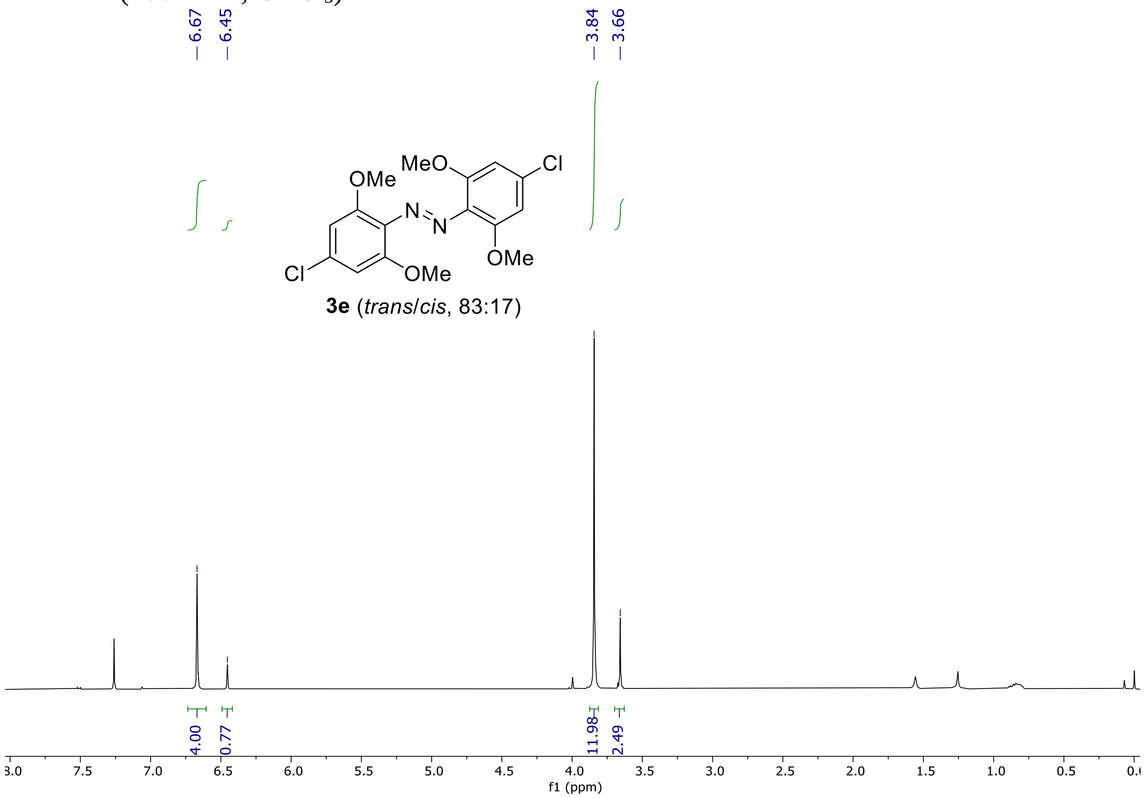

^{13}C{^{1}H}-NMR (100 MHz, $CDCl_3$)

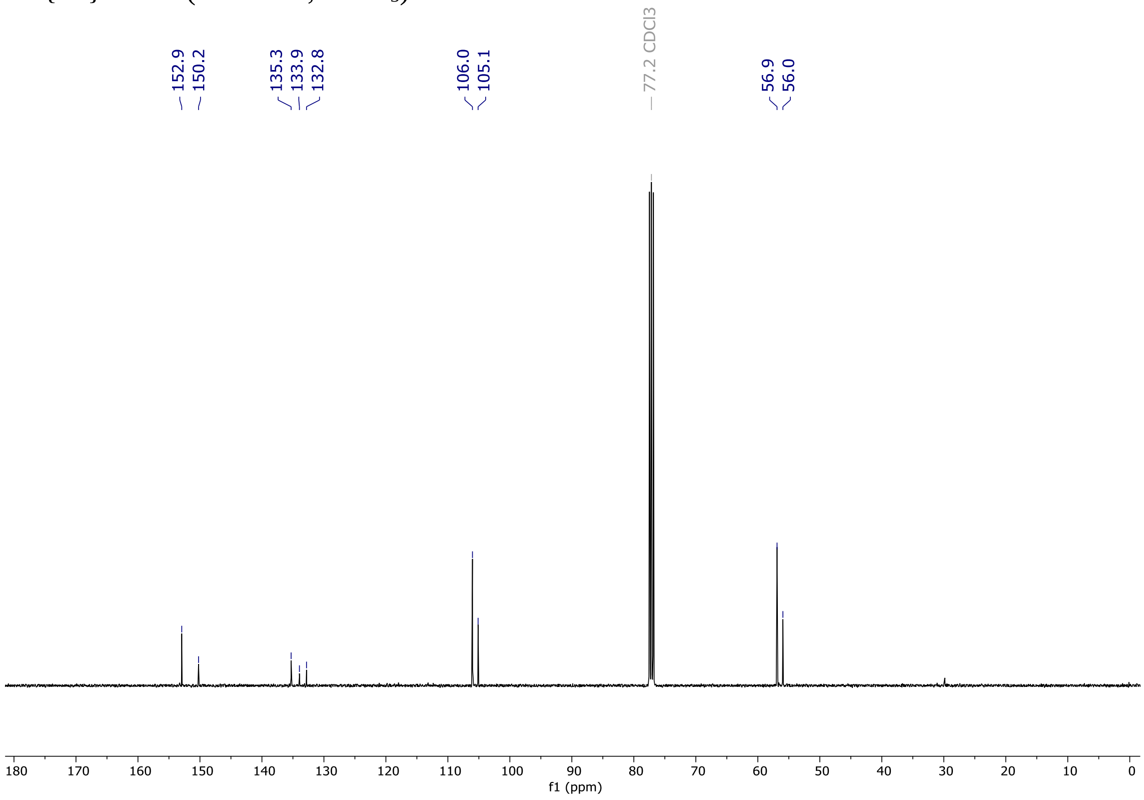

HSQC ($CDCl_3$)

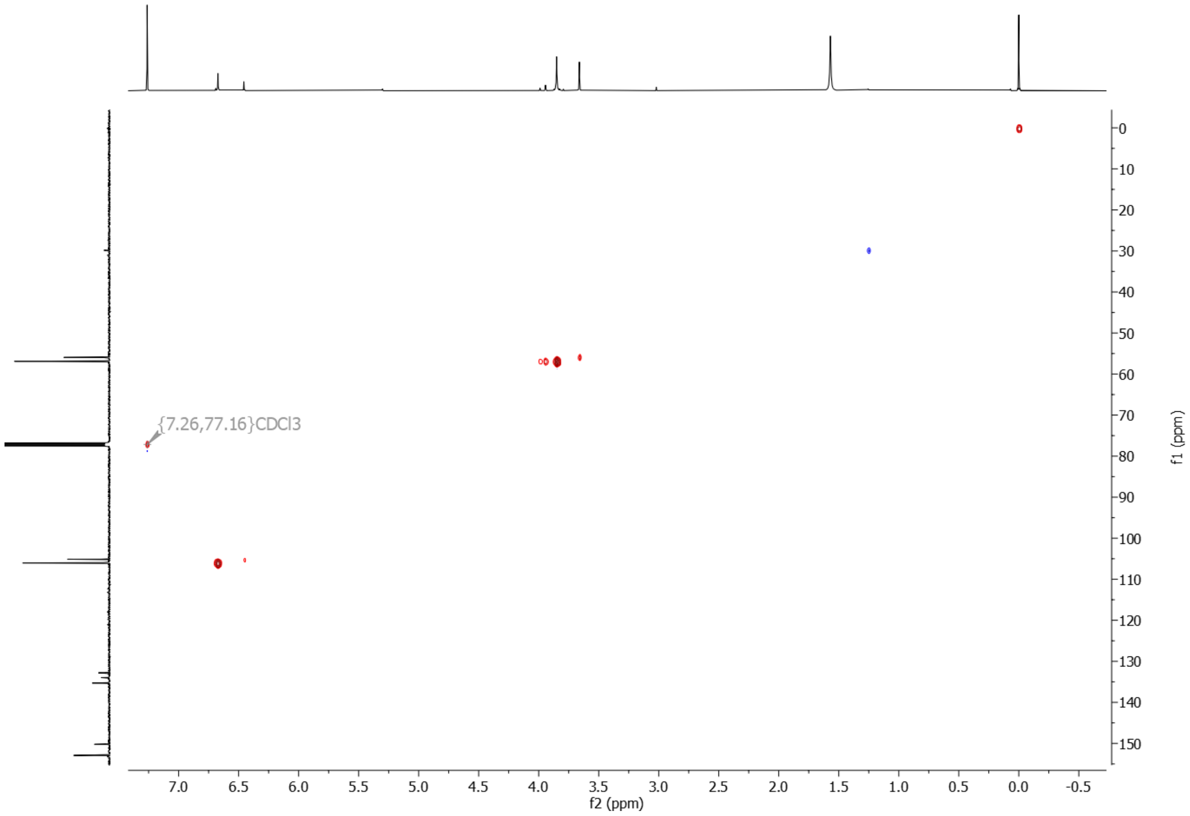

{7.26,77.16}CDCl3
7.0
6.5
6.0
5.5
5.0
4.5
4.0
3.5
3.0
2.5
2.0
1.5
1.0
0.5
0.0
-0.5
f2 (ppm)
0
10
20
30
40
50
60
70
80
90
100
110
120
130
140
150
f1 (ppm)

(*E*)-1,2-Bis(2,6-dimethoxyphenyl-4-bromophenyl)diazene (3f)

^{1}H-NMR (400 MHz, $CDCl_3$)

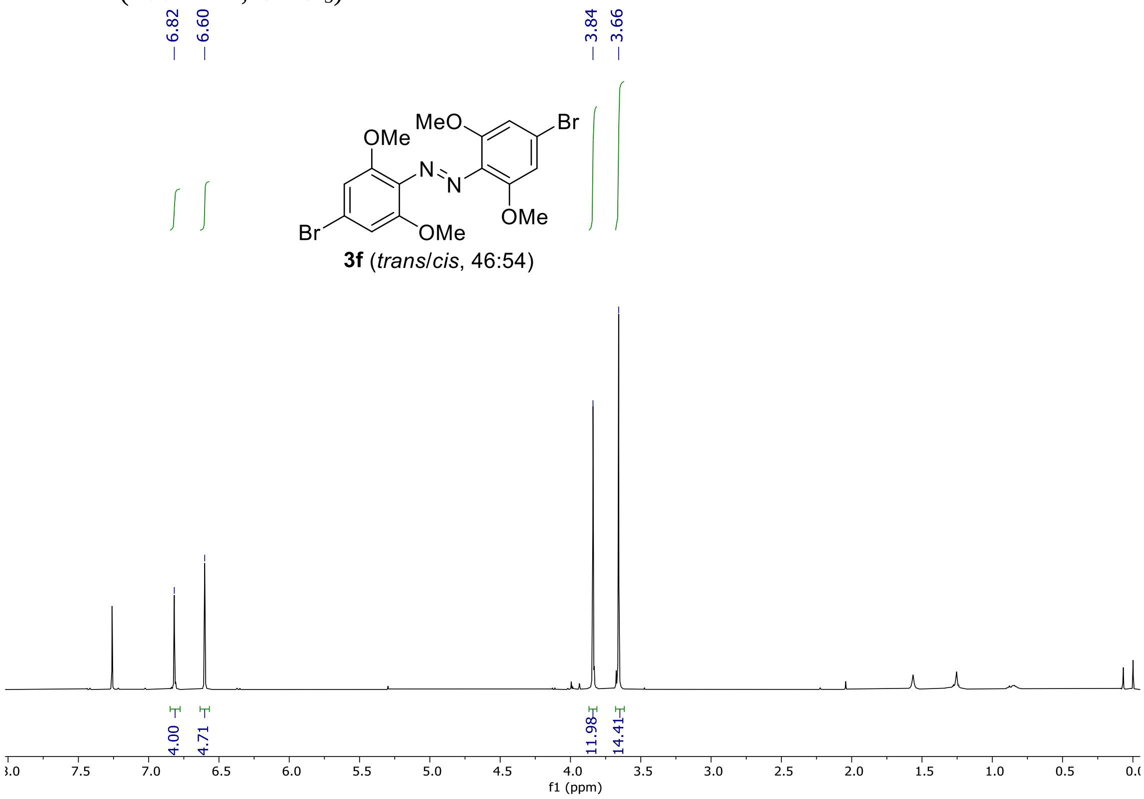

^{13}C{^{1}H}-NMR (100 MHz, $CDCl_3$)

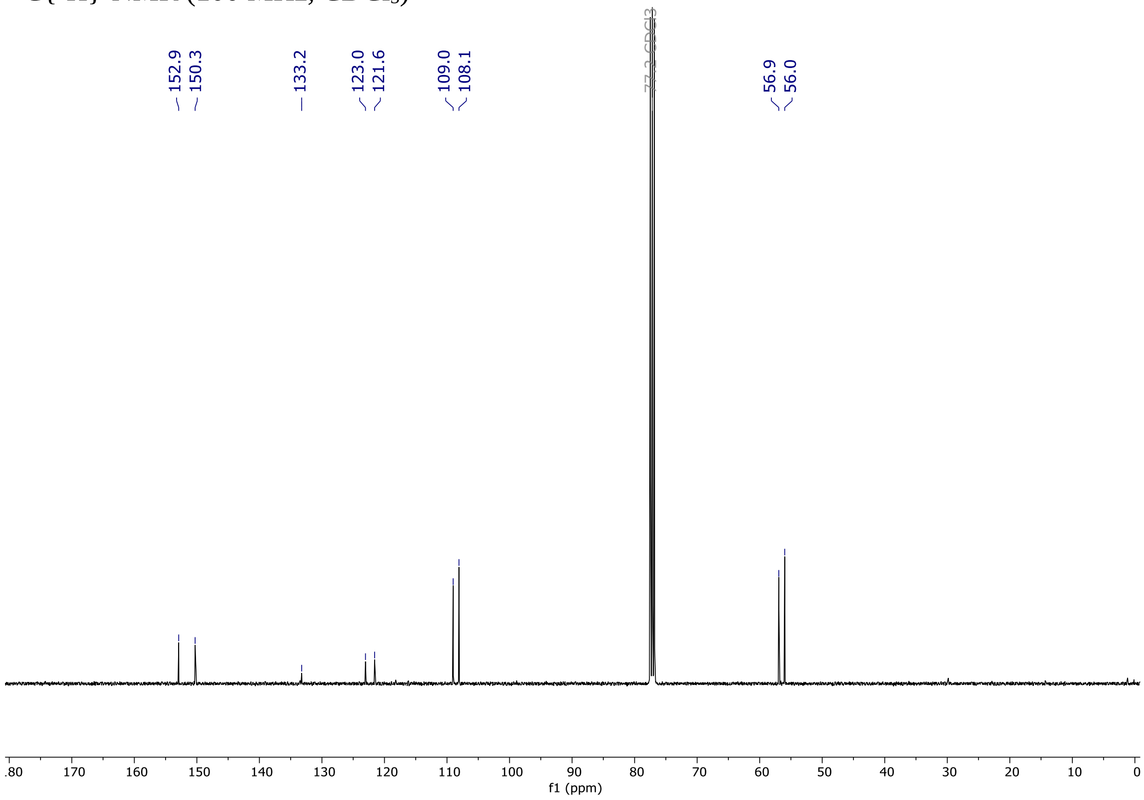

HSQC ($CDCl_3$)

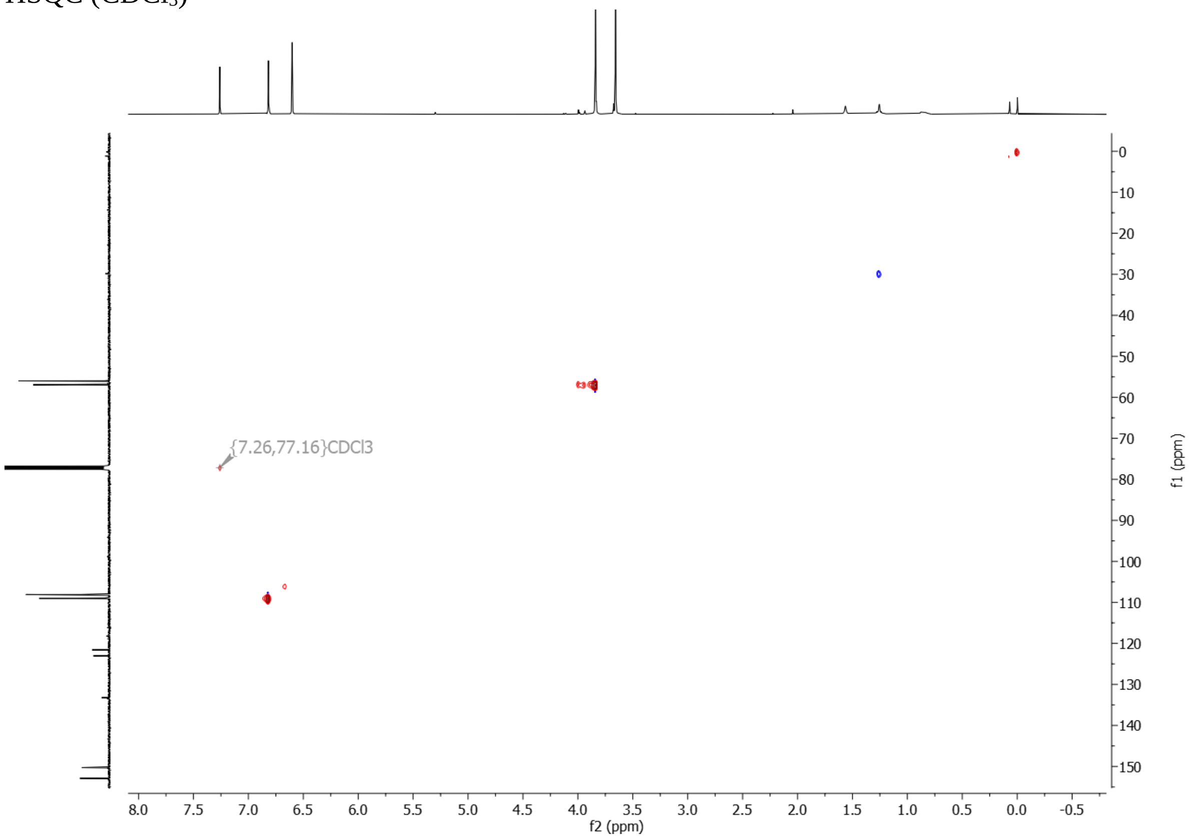

{7.26,77.16}CDCl3
8.0
7.5
7.0
6.5
6.0
5.5
5.0
4.5
4.0
3.5
3.0
2.5
2.0
1.5
1.0
0.5
0.0
-0.5
f2 (ppm)
0
10
20
30
40
50
60
70
80
90
100
110
120
130
140
150
f1 (ppm)

(*E*)-1,2-Bis(2,6-dimethoxyphenyl-4-iodophenyl)diazene (3g)

^{1}H-NMR (400 MHz, $CDCl_3$)

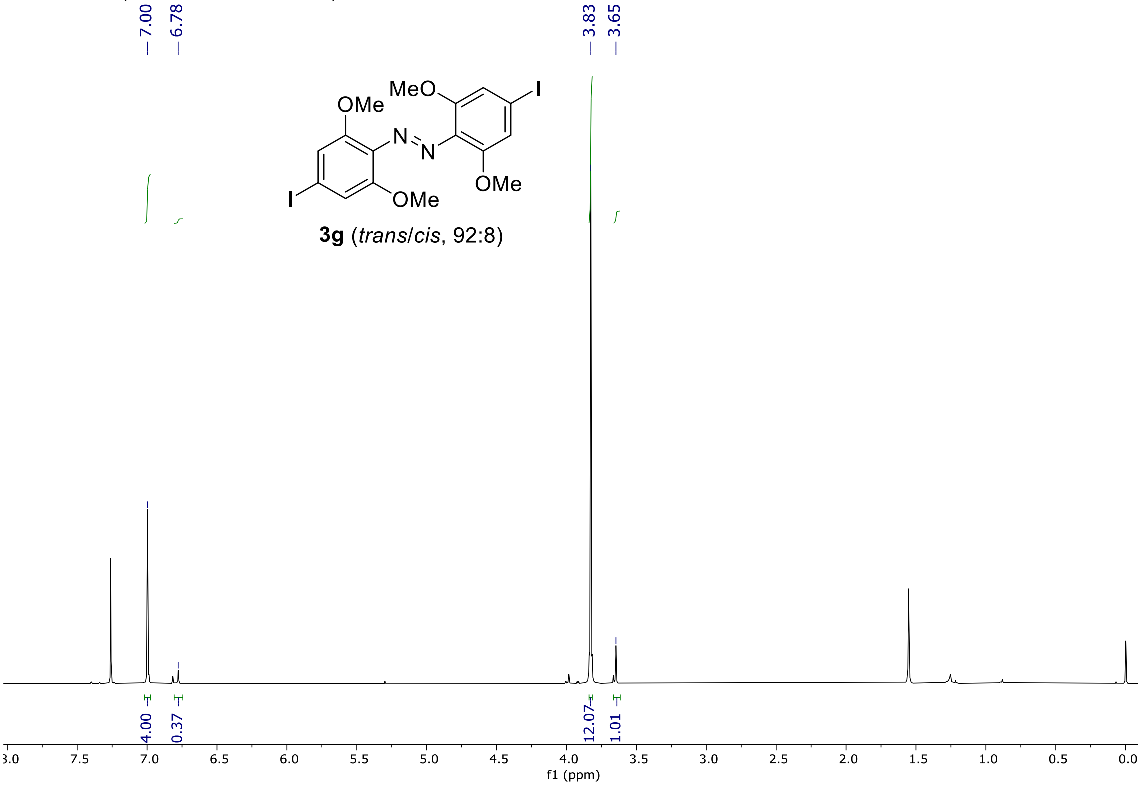

^{13}C{^{1}H}-NMR (100 MHz, $CDCl_3$)

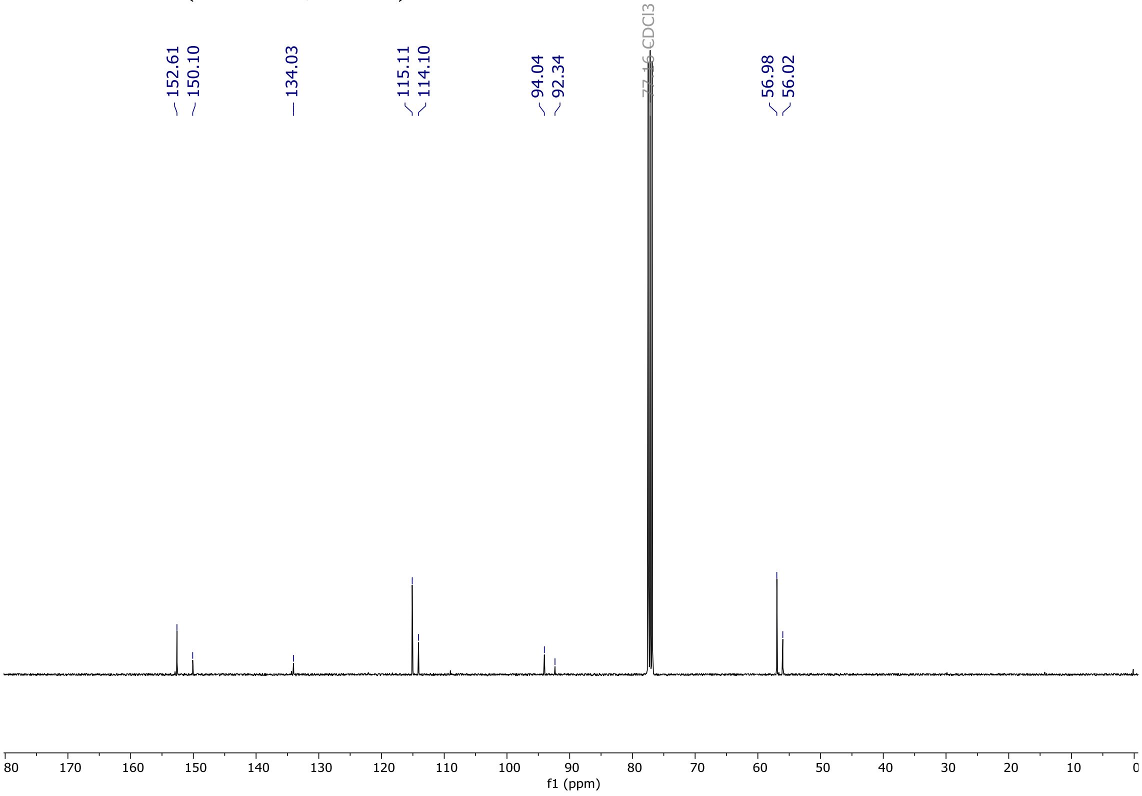

HSQC ($CDCl_3$)

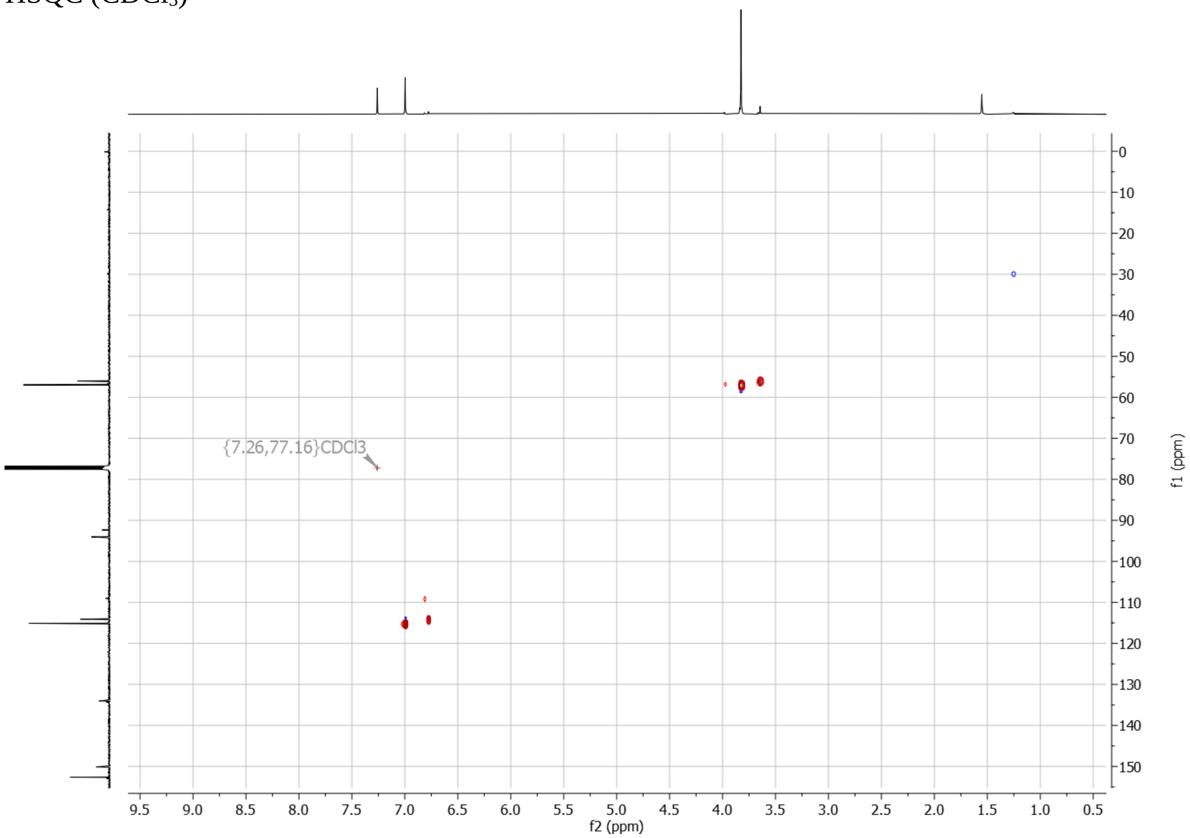

(*E*)-1,2-Bis(2,6-dimethoxyphenyl-4-trifluoromethylphenyl)diazene (3h)

^{1}H-NMR (400 MHz, $CDCl_3$)

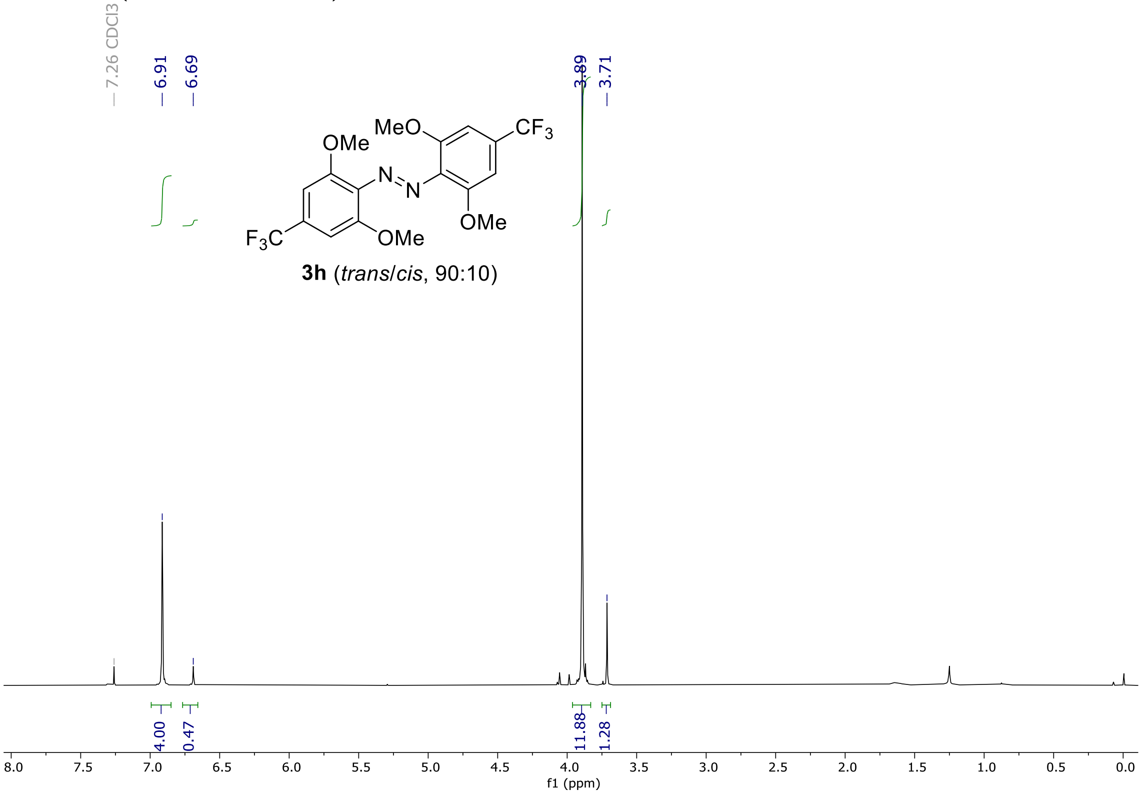

^{13}C{^{1}H}-NMR (100 MHz, $CDCl_3$)

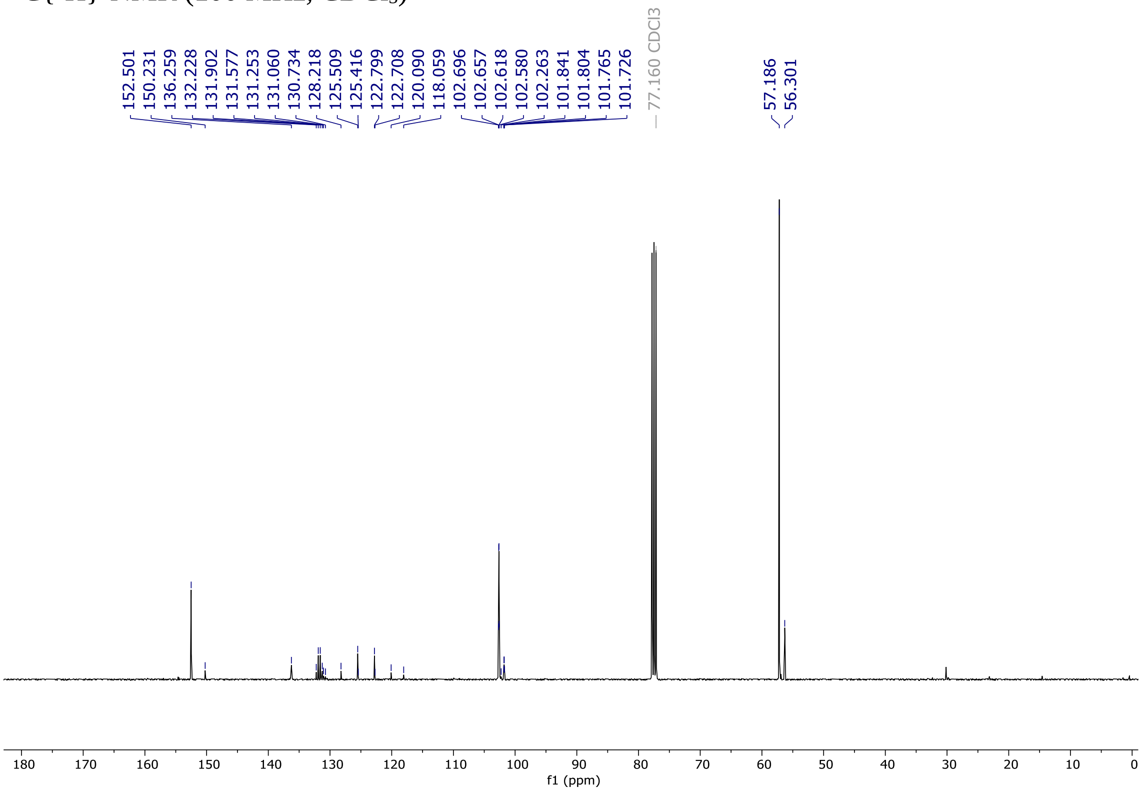

^{19}F-NMR (376 MHz, $CDCl_3$)

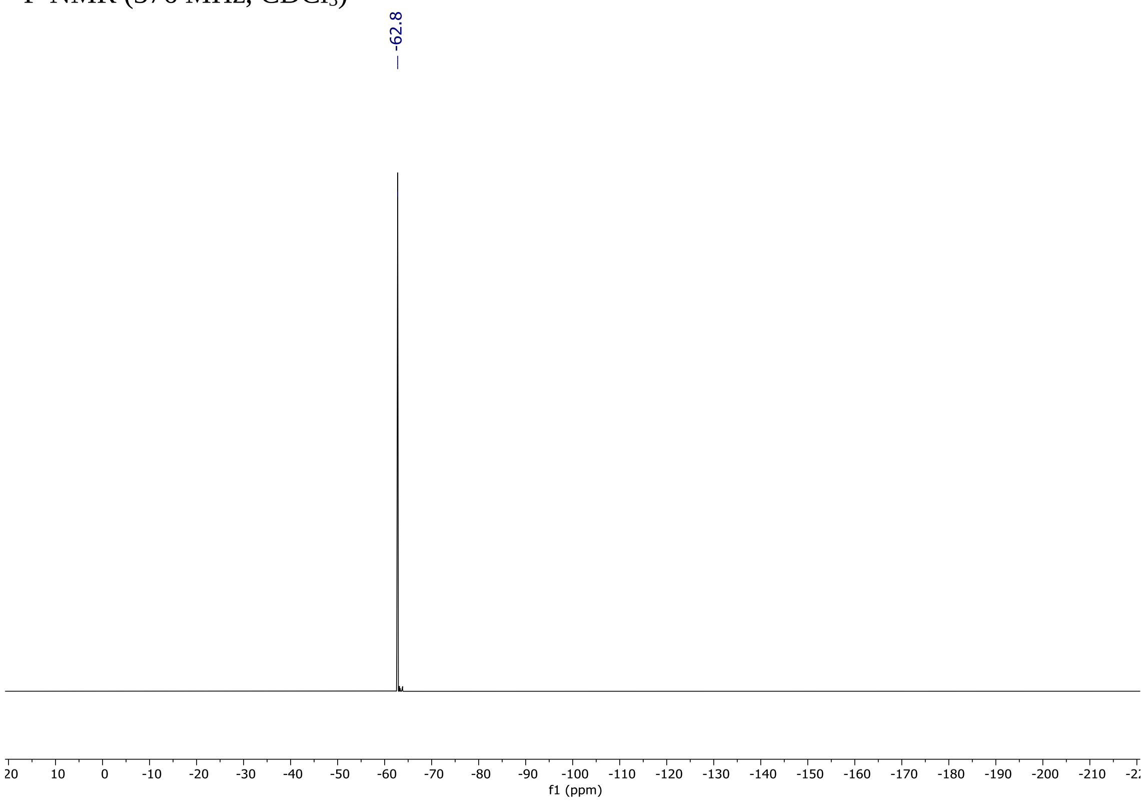

HSQC ($CDCl_3$)

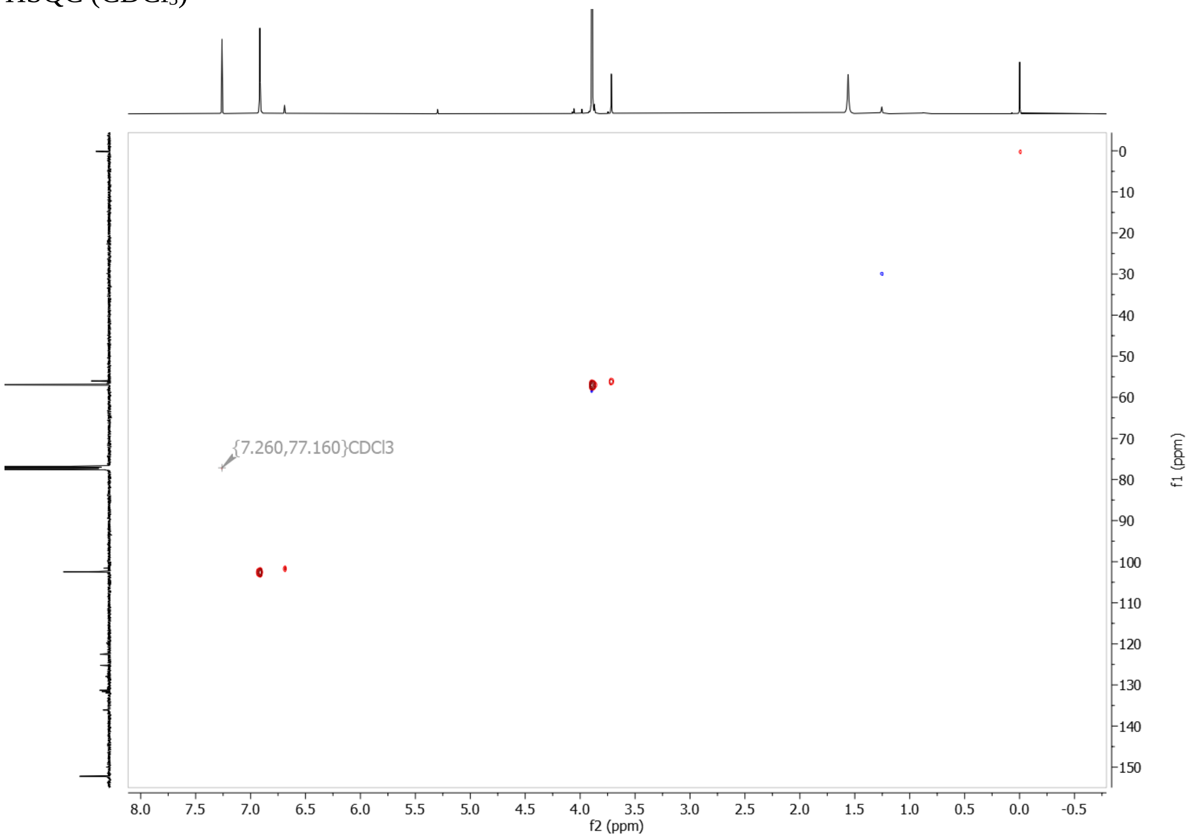

(*E*)-3-(3,5-Dimethoxy-4-((2,6-dimethoxyphenyl)diazenyl)phenyl)propanoic acid (3i)

^{1}H-NMR (400 MHz, $CDCl_3$)

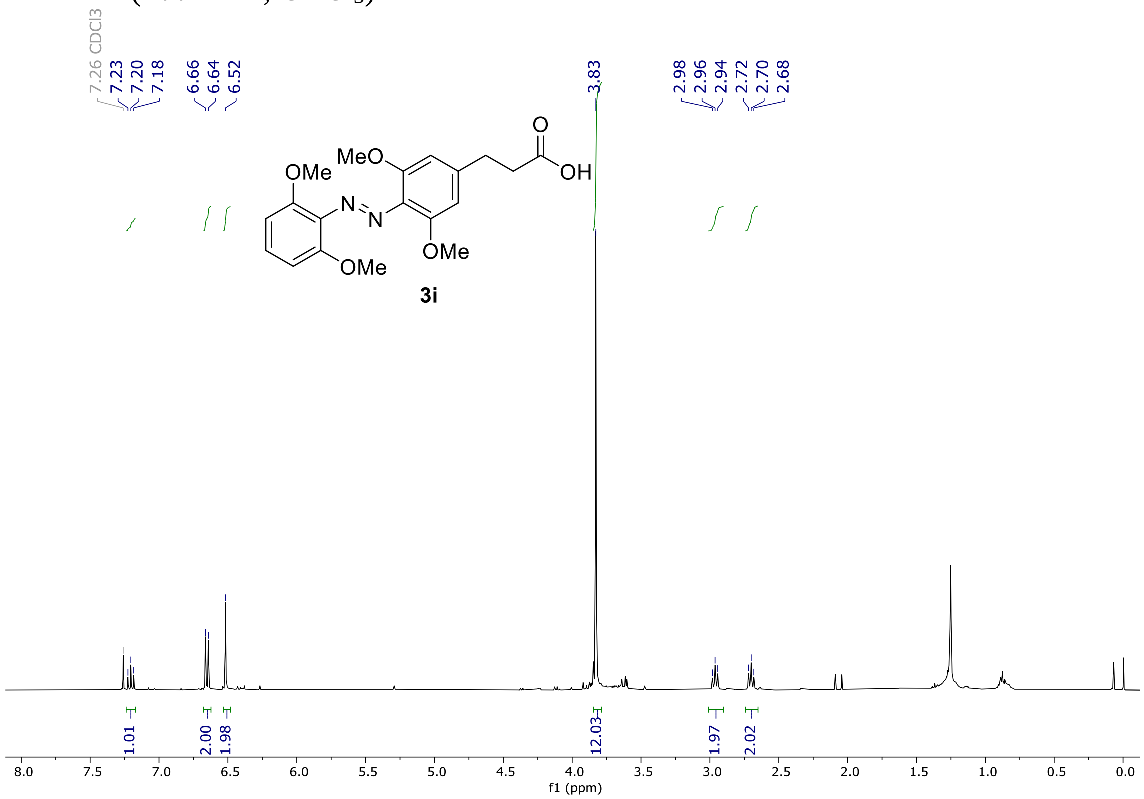

^{13}C{^{1}H}-NMR (100 MHz, $CDCl_3$)

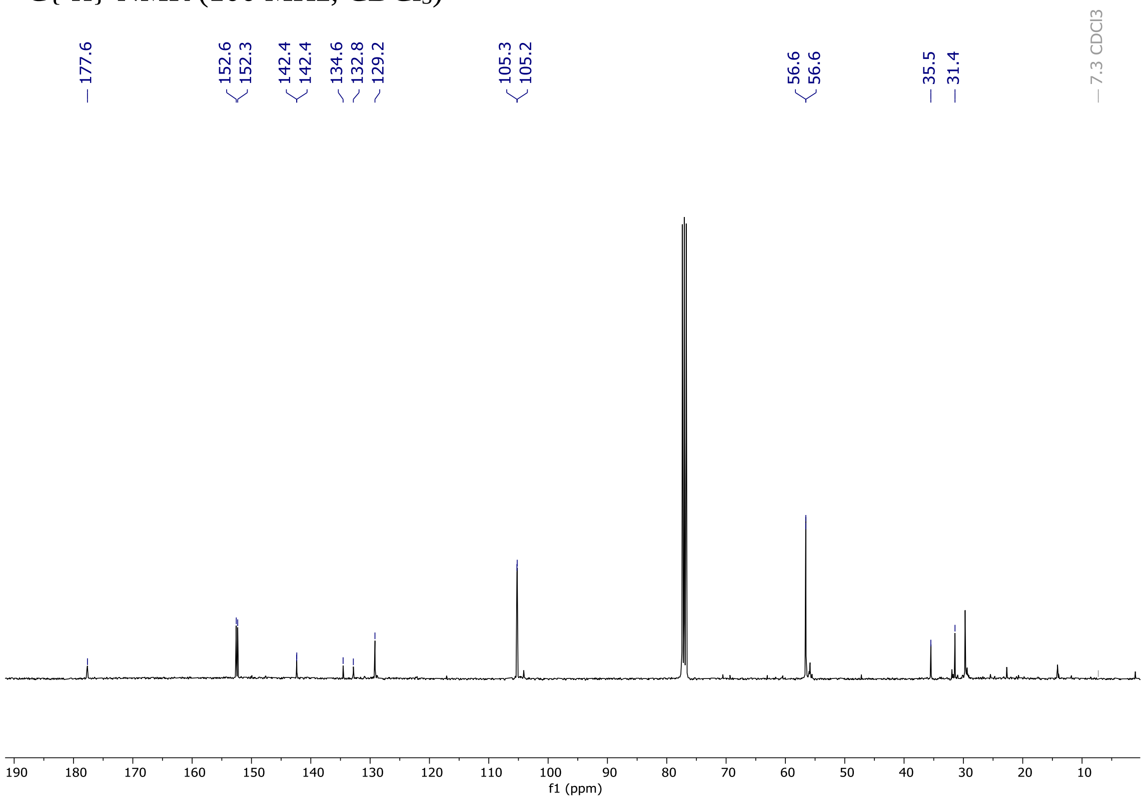

HSQC ($CDCl_3$)

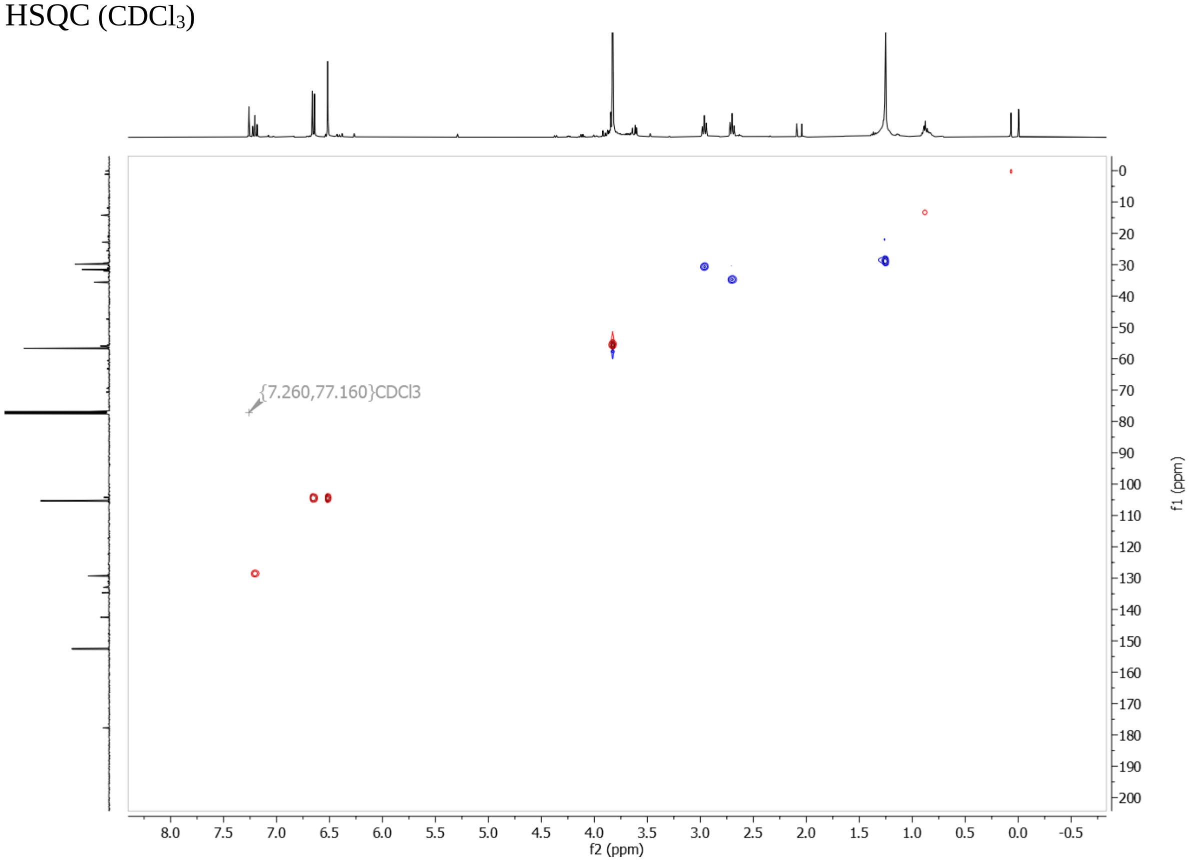

(*E*)-3-(3,5-Dimethoxy-4-((2,6-dimethoxyphenyl)diazenyl)benzoic acid (3j)

^{1}H-NMR (400 MHz, DMSO-d$_6$)

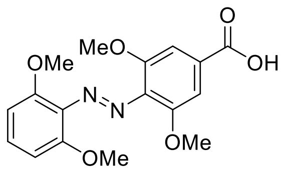

3j

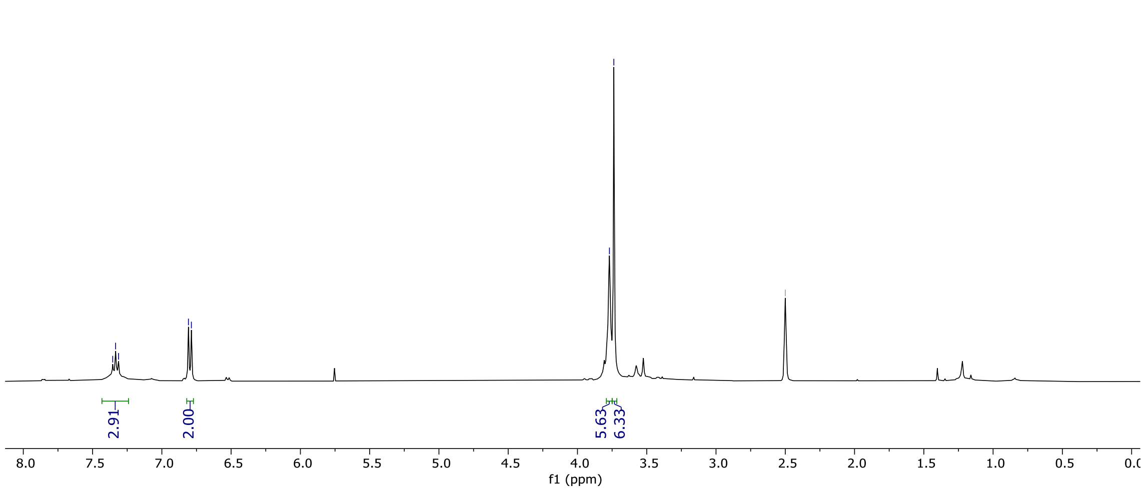

^{13}C{^{1}H}-NMR (100 MHz, DMSO-d$_6$)

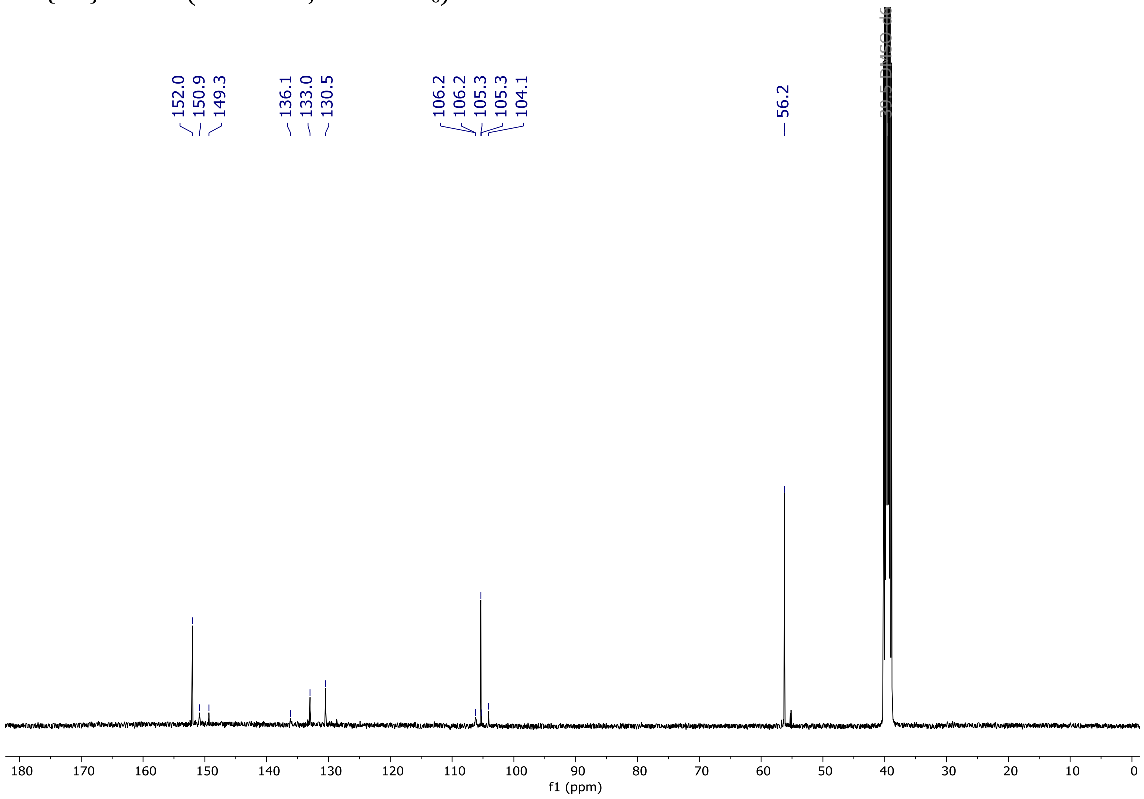

HSQC (DMSO-d$_6$)

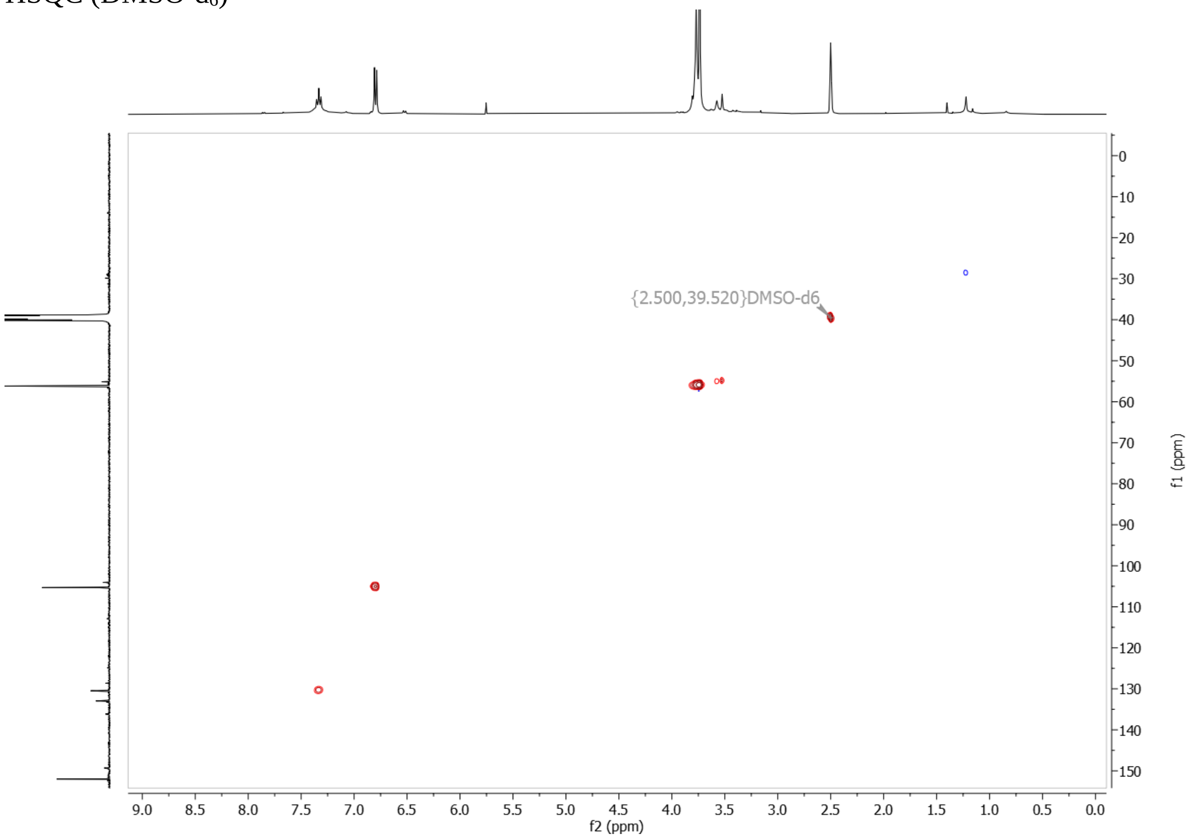

Dimethyl 3,3'-(diazene-1,2-diylbis(3,5-dimethoxy-4,1-phenylene))(*E*)-dipropionate (3k)

^{1}H-NMR (400 MHz, $CDCl_3$)

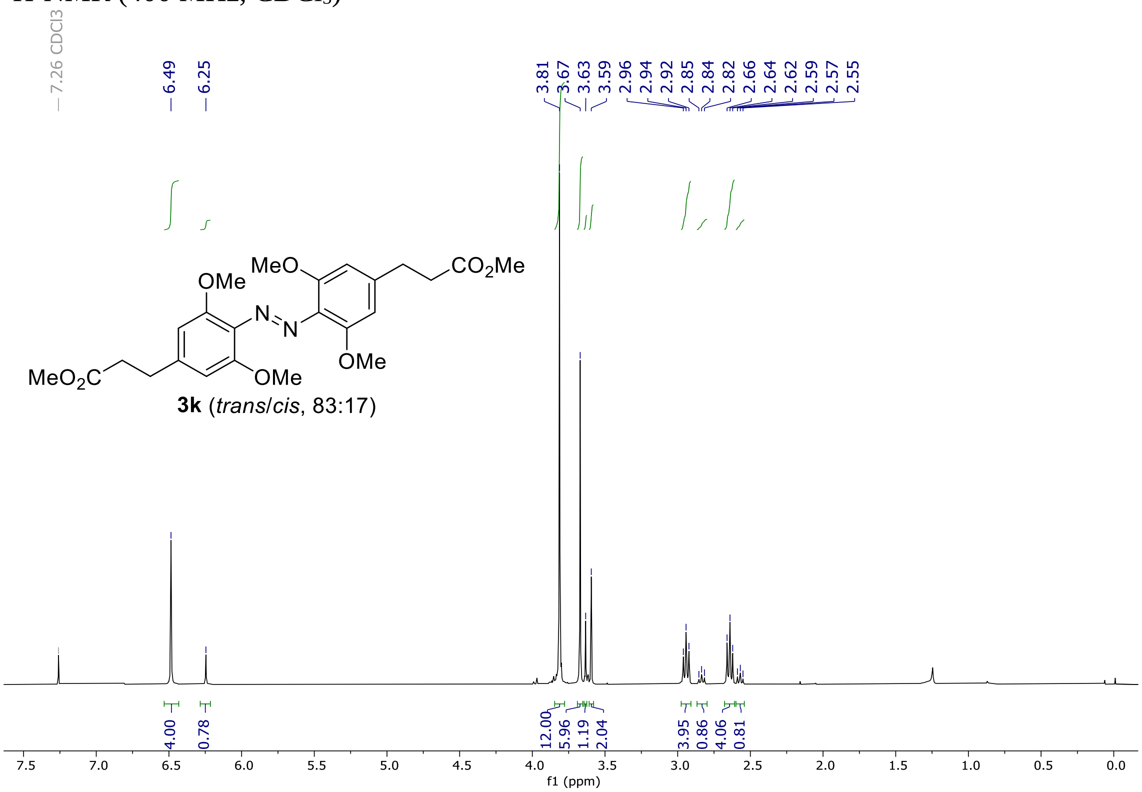

^{13}C{^{1}H}-NMR (100 MHz, $CDCl_3$)

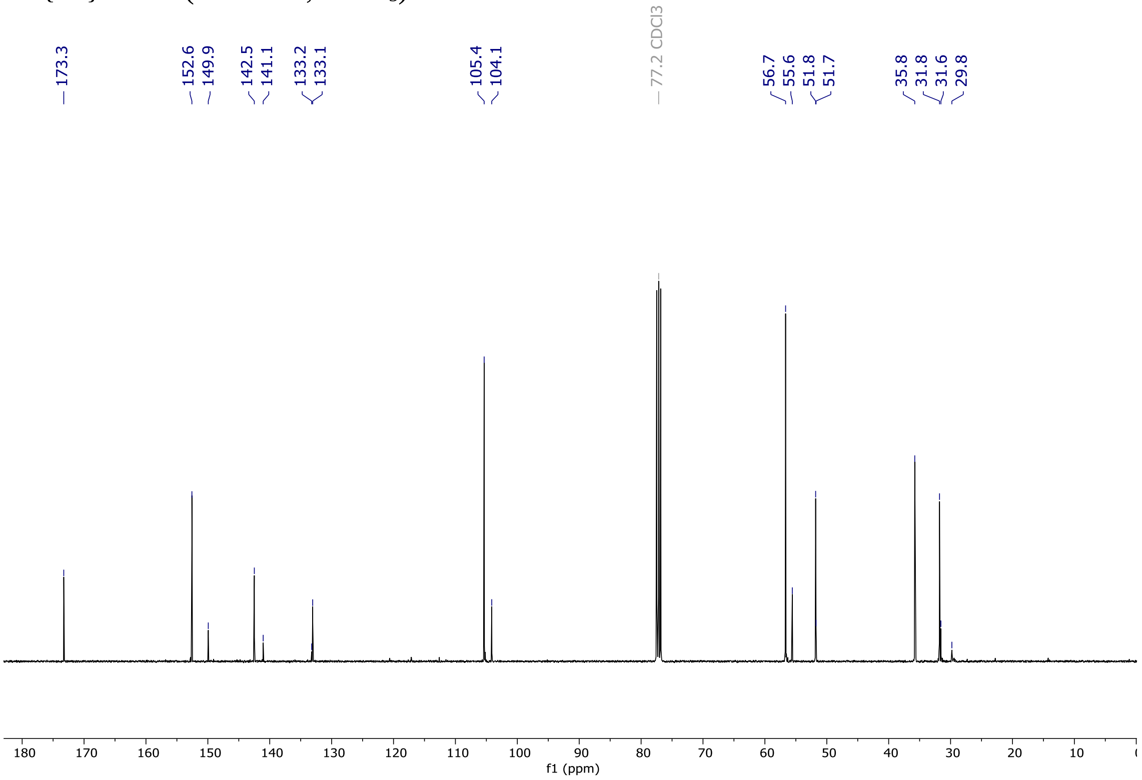

HSQC ($CDCl_3$)

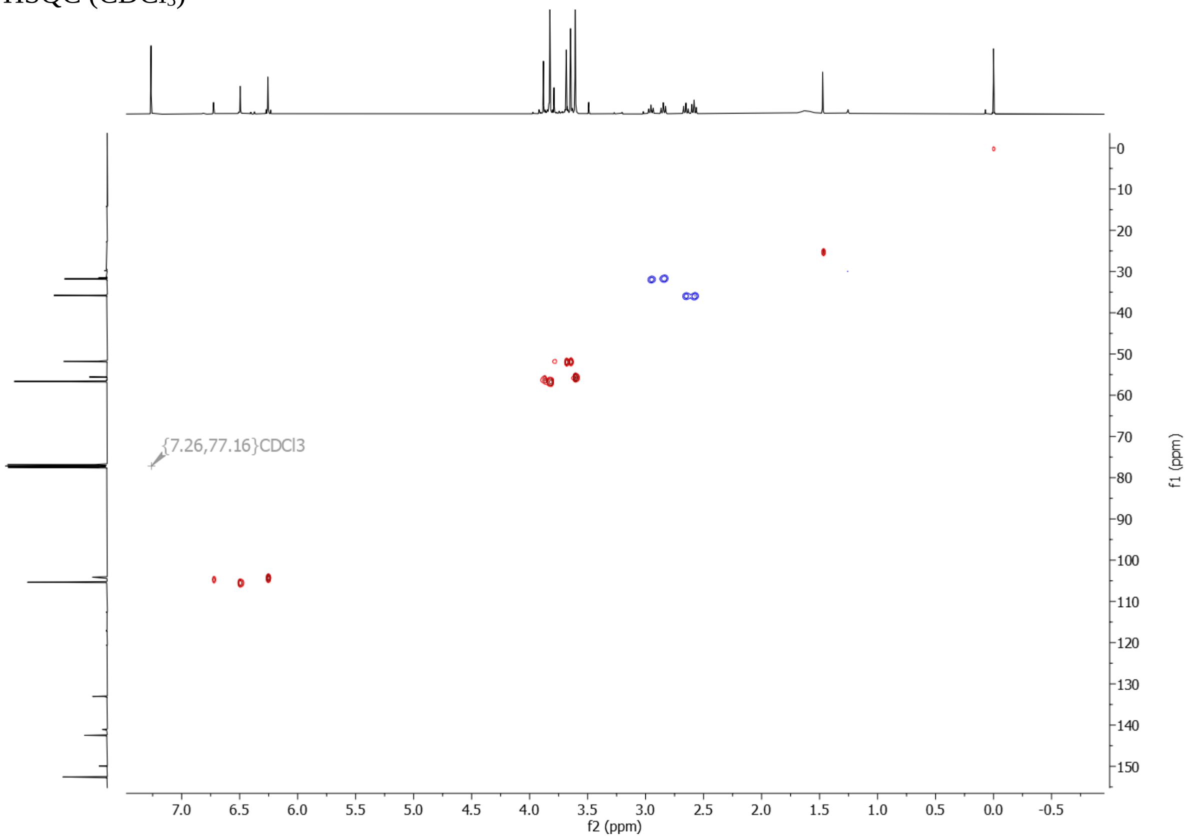
{7.26,77.16}CDCl3
7.0
6.5
6.0
5.5
5.0
4.5
4.0
3.5
3.0
2.5
2.0
1.5
1.0
0.5
0.0
-0.5
f2 (ppm)
0
10
20
30
40
50
60
70
80
90
100
110
120
130
140
150
f1 (ppm)

Methyl (*E*)-3-(3,5-dimethoxy-4-((2,6-dimethoxyphenyl)diazenyl)phenyl)propanoate (3l)

^{1}H-NMR (400 MHz, $CDCl_3$)

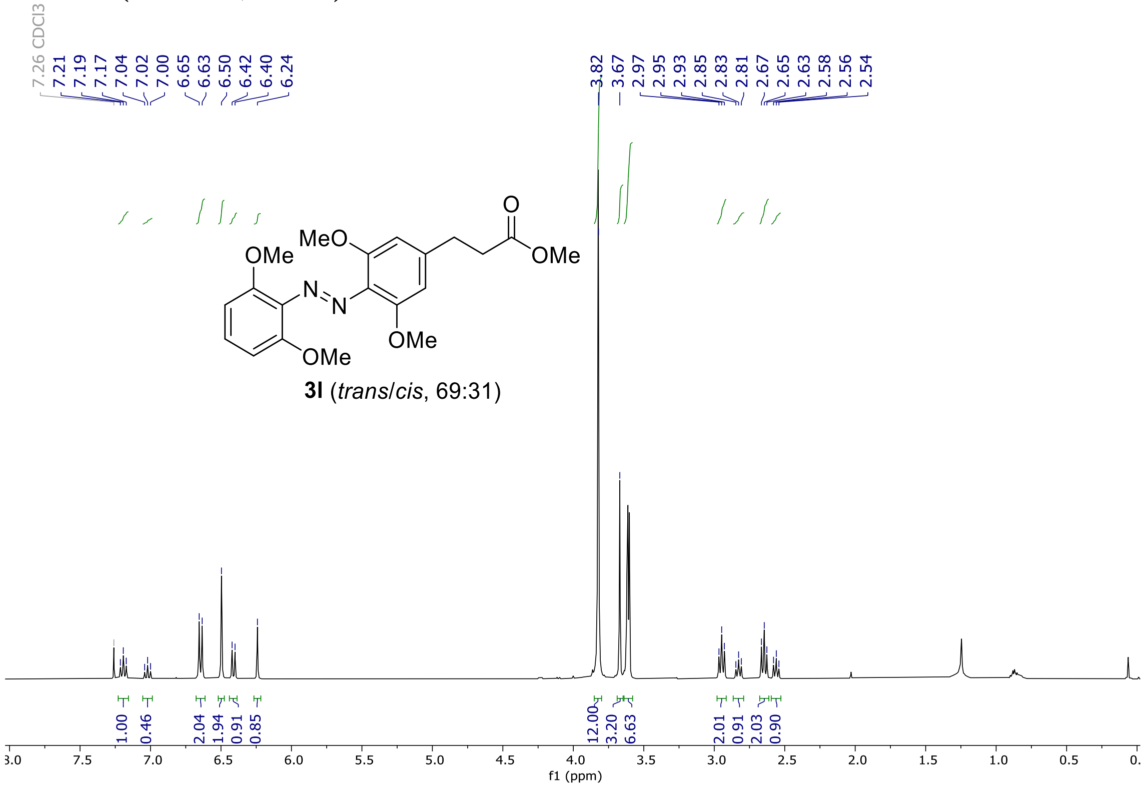

^{13}C{^{1}H}-NMR (100 MHz, $CDCl_3$)

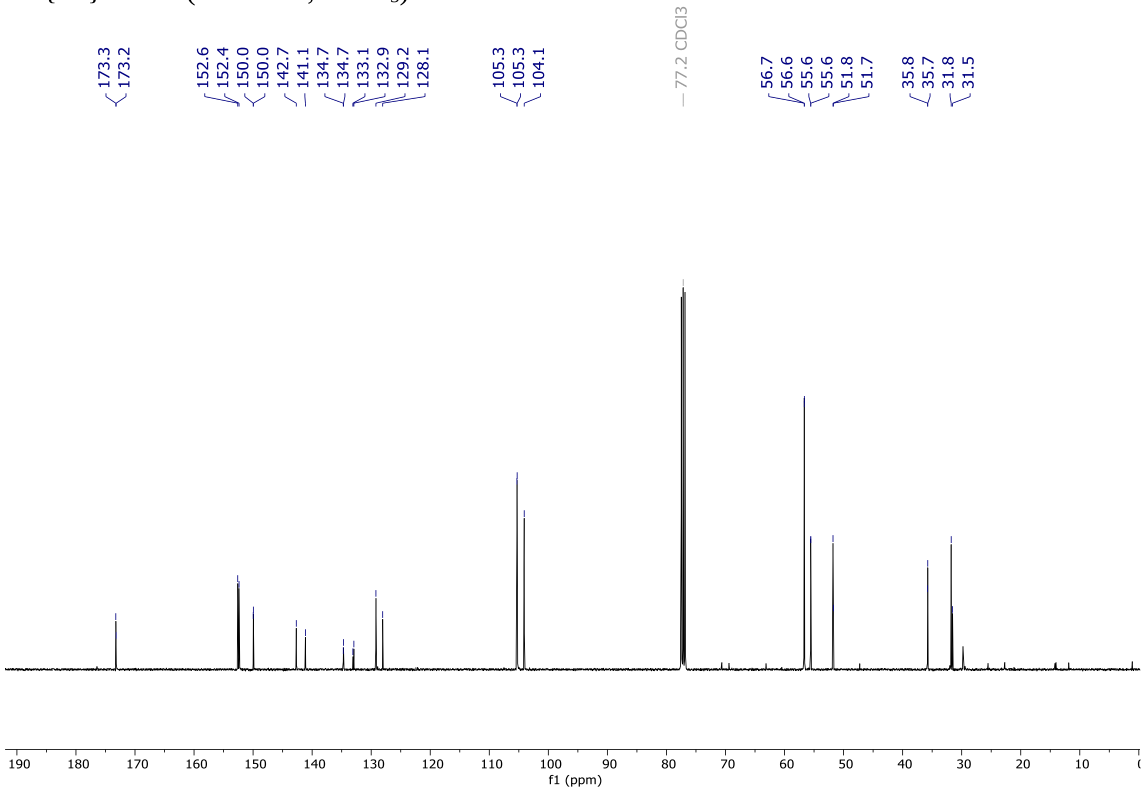

HSQC ($CDCl_3$)

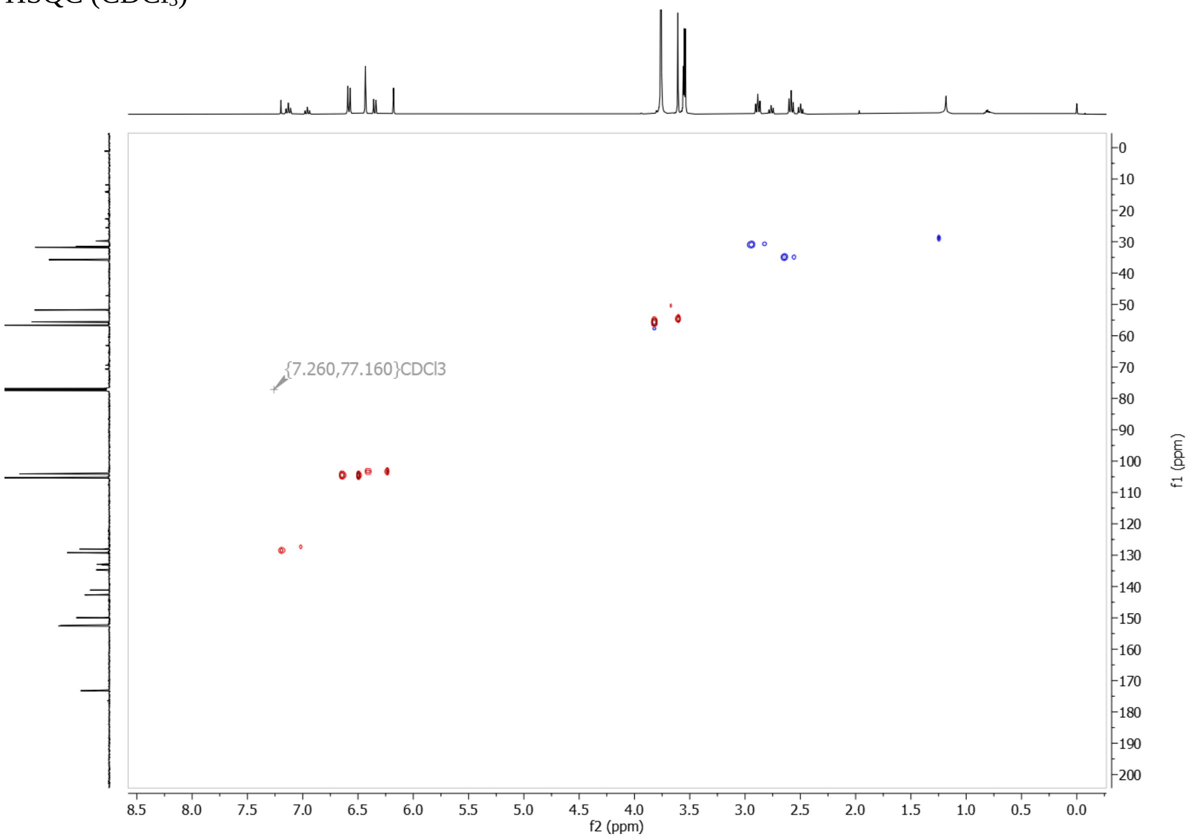

Methyl (*E*)-3-(3,5-dimethoxy-4-((2,6-dimethoxyphenyl)diazenyl)benzoate (3m)

^{1}H-NMR (400 MHz, $CDCl_3$)

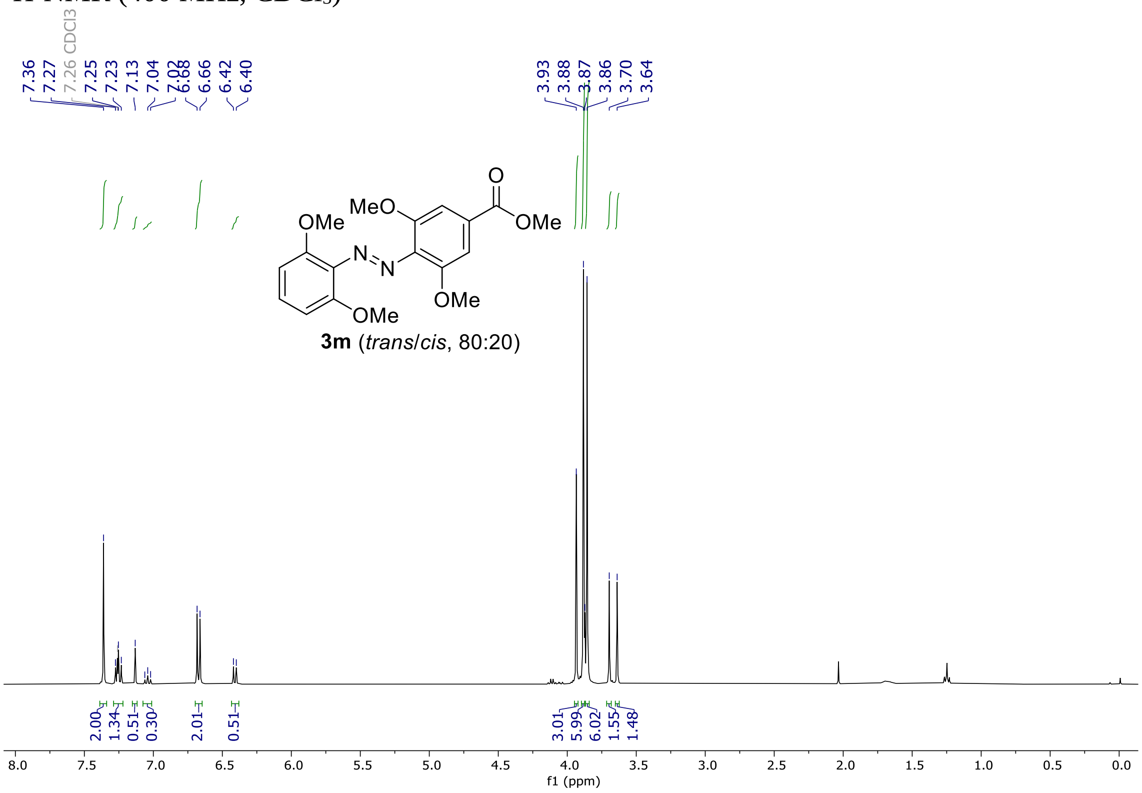

^{13}C{^{1}H}-NMR (100 MHz, $CDCl_3$)

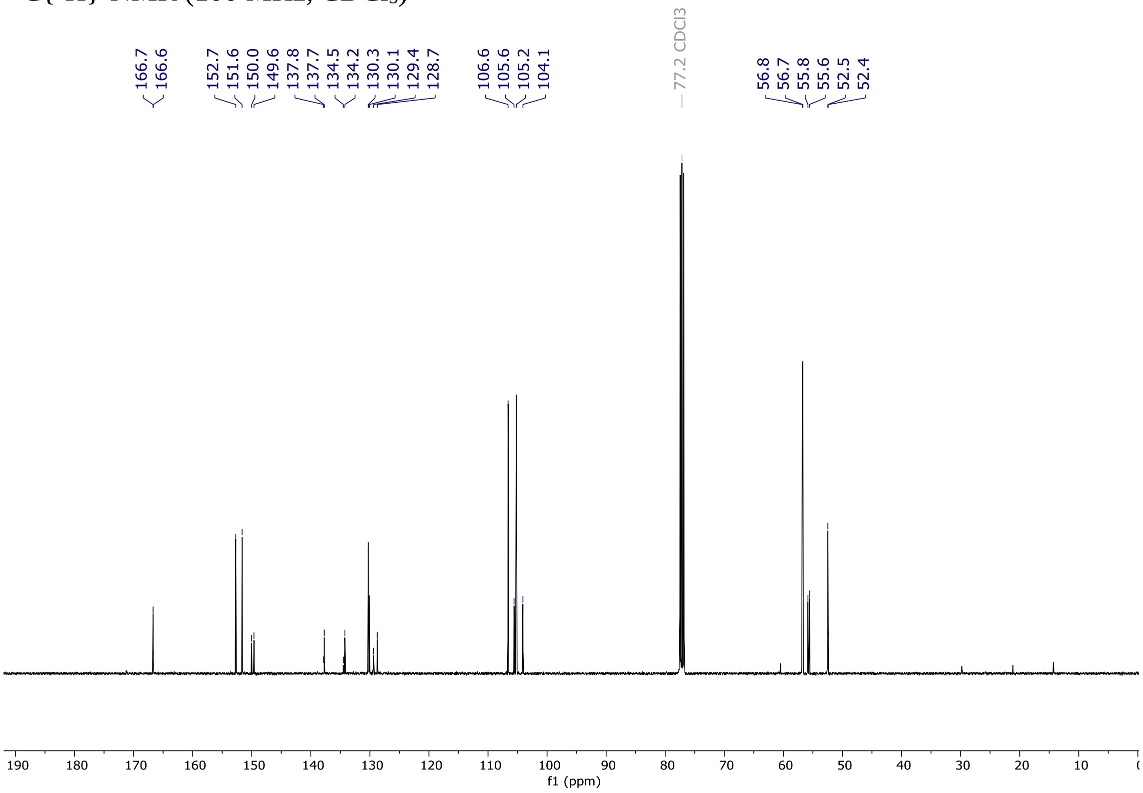

HSQC ($CDCl_3$)

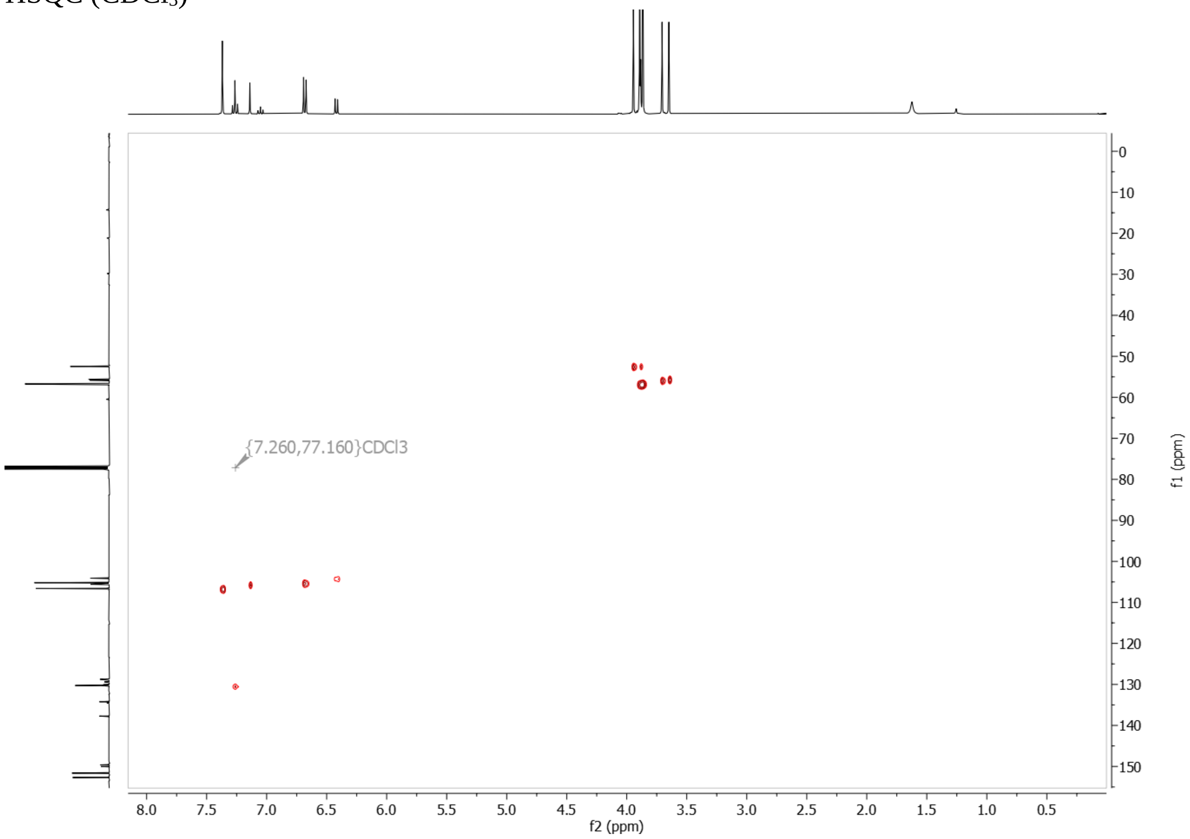

(*E*)-*N*-(3,5-Dimethoxy-4-((2,6-dimethoxyphenyl)diazenyl)phenethyl)-2,2,2-trifluoroacetamide (3n)

^{1}H-NMR (400 MHz, $CDCl_3$)

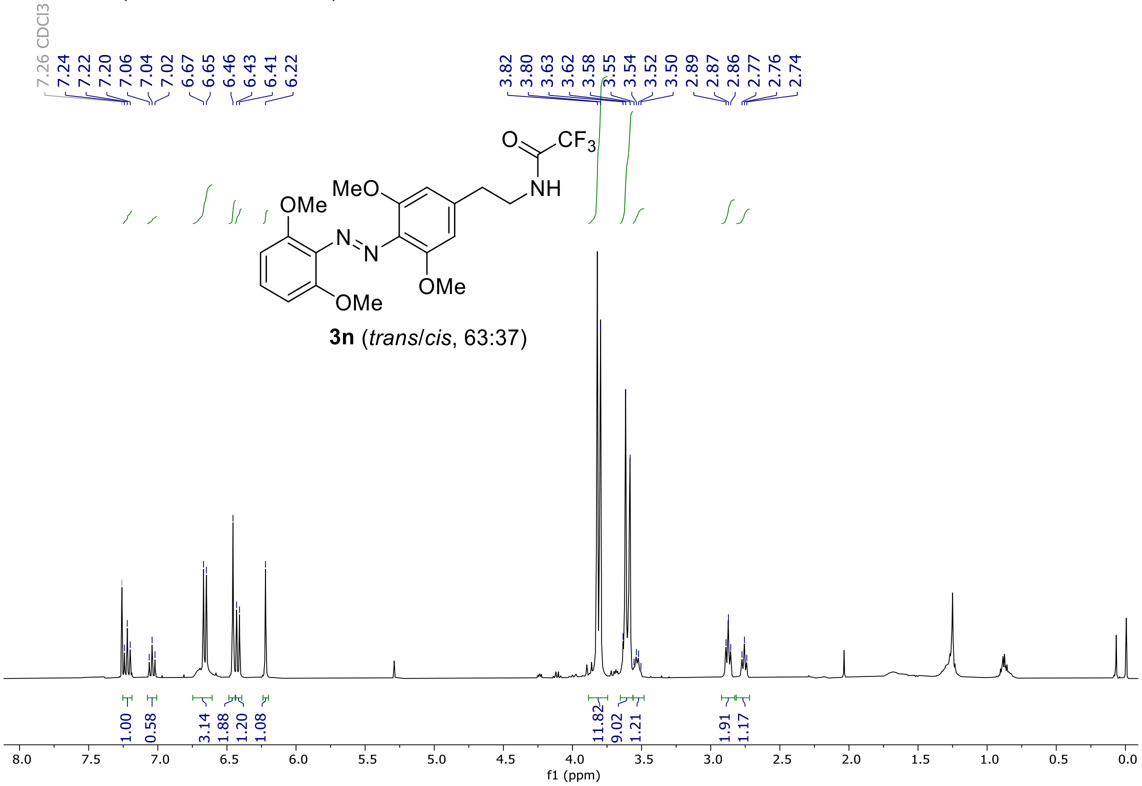

^{13}C{^{1}H}-NMR (100 MHz, $CDCl_3$)

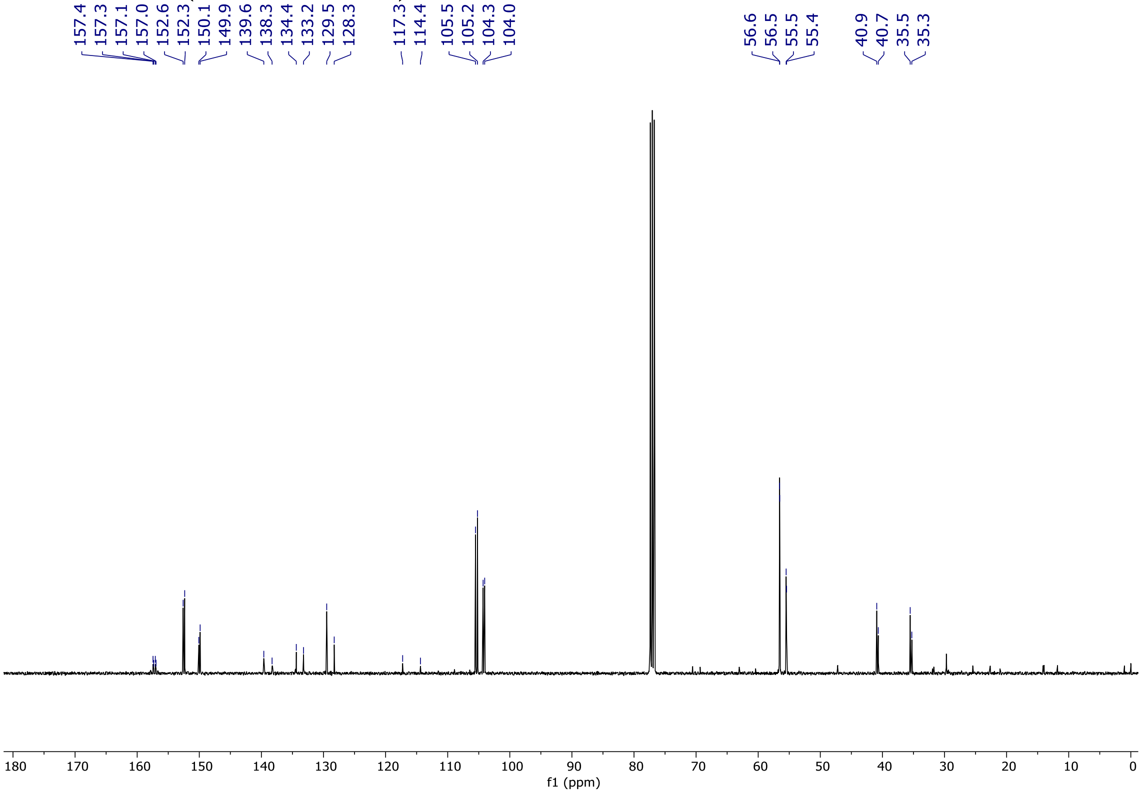

^{19}F-NMR (376 MHz, $CDCl_3$)

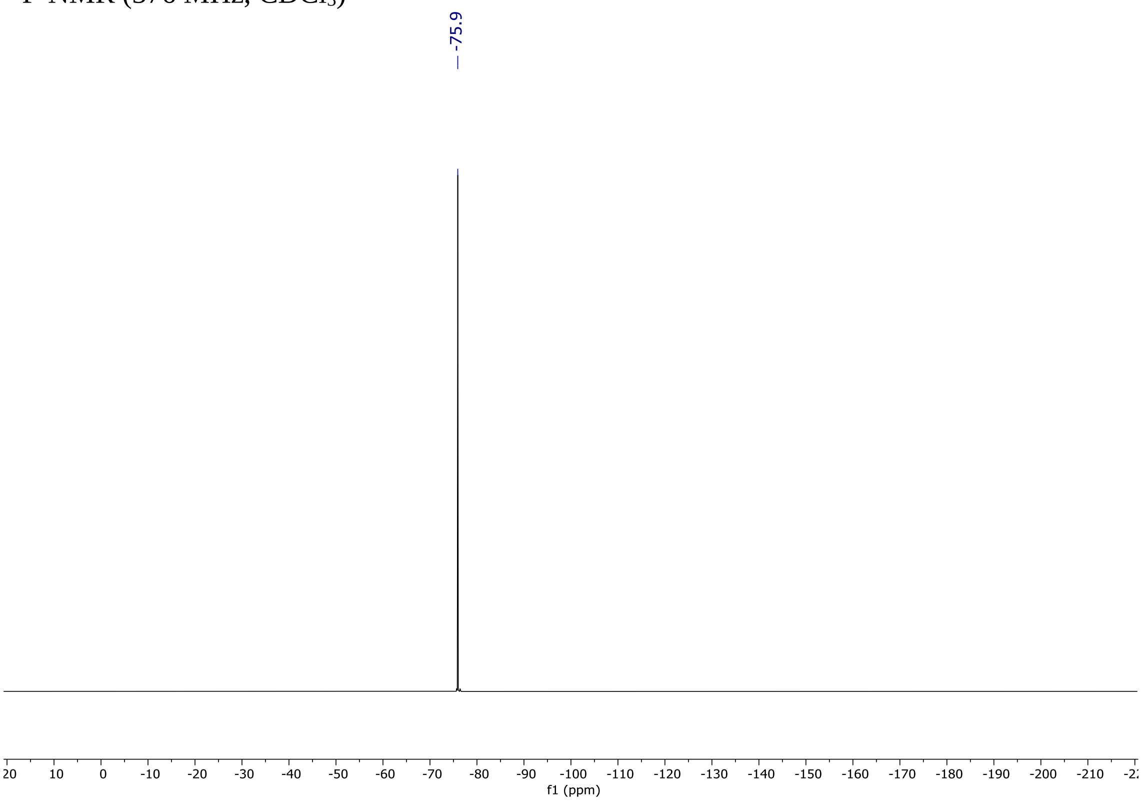

HSQC ($CDCl_3$)

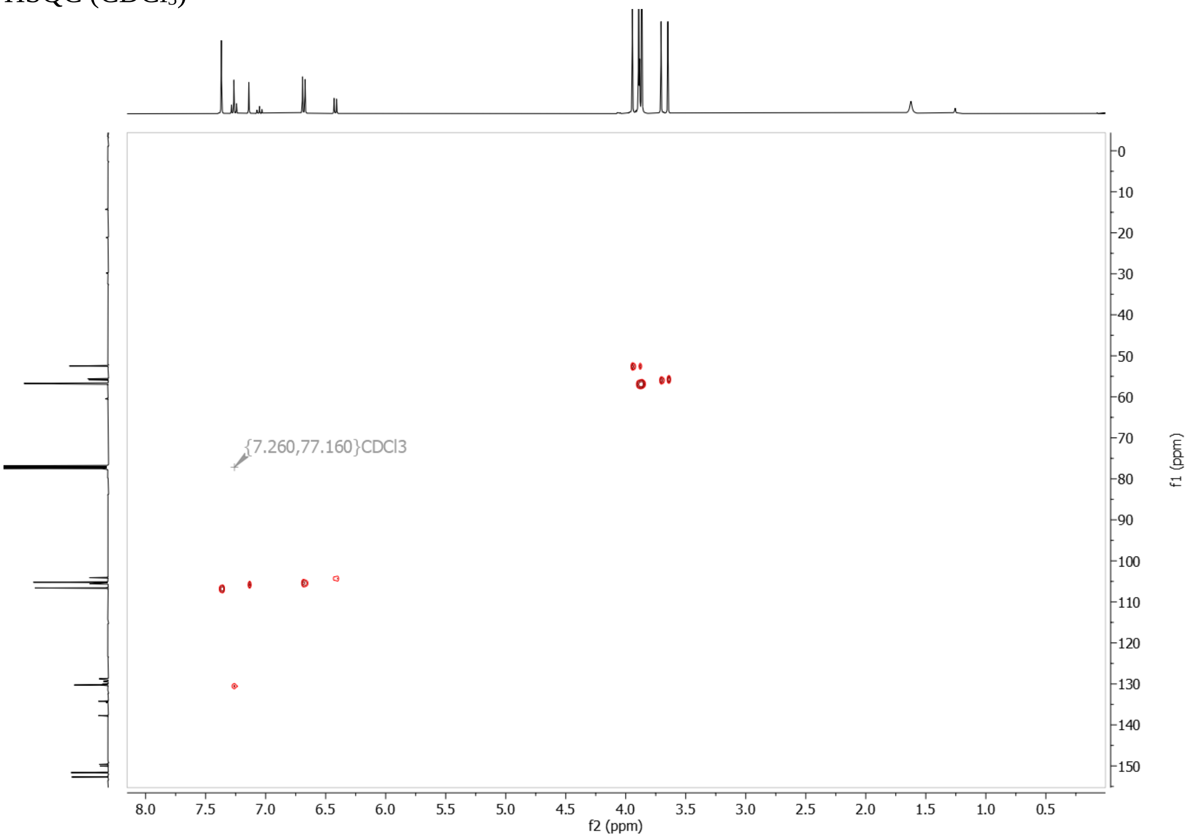

(*E*)-*N*-(3,5-Dimethoxy-4-((2,6-dimethoxyphenyl)diazenyl)benzyl)-2,2,2-trifluoroacetamide (3o)

^{1}H-NMR (400 MHz, $CDCl_3$)

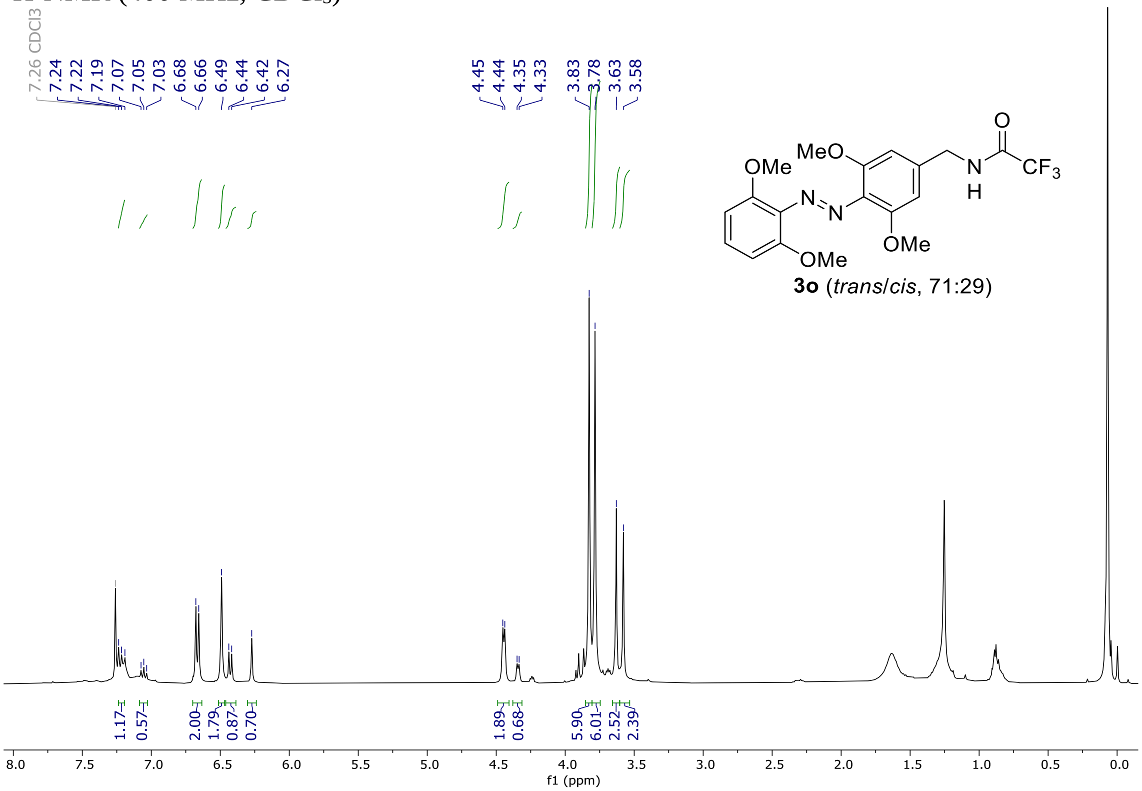

^{13}C{^{1}H}-NMR (100 MHz, $CDCl_3$)

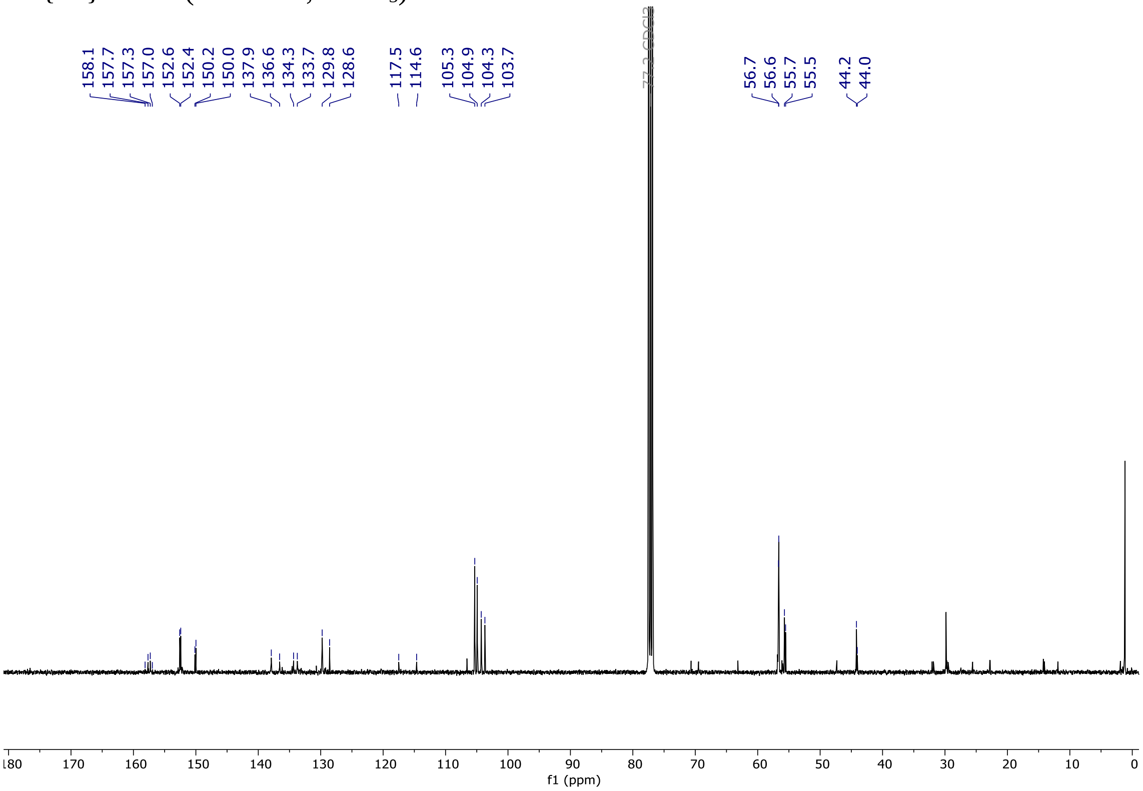

^{19}F-NMR (376 MHz, $CDCl_3$)

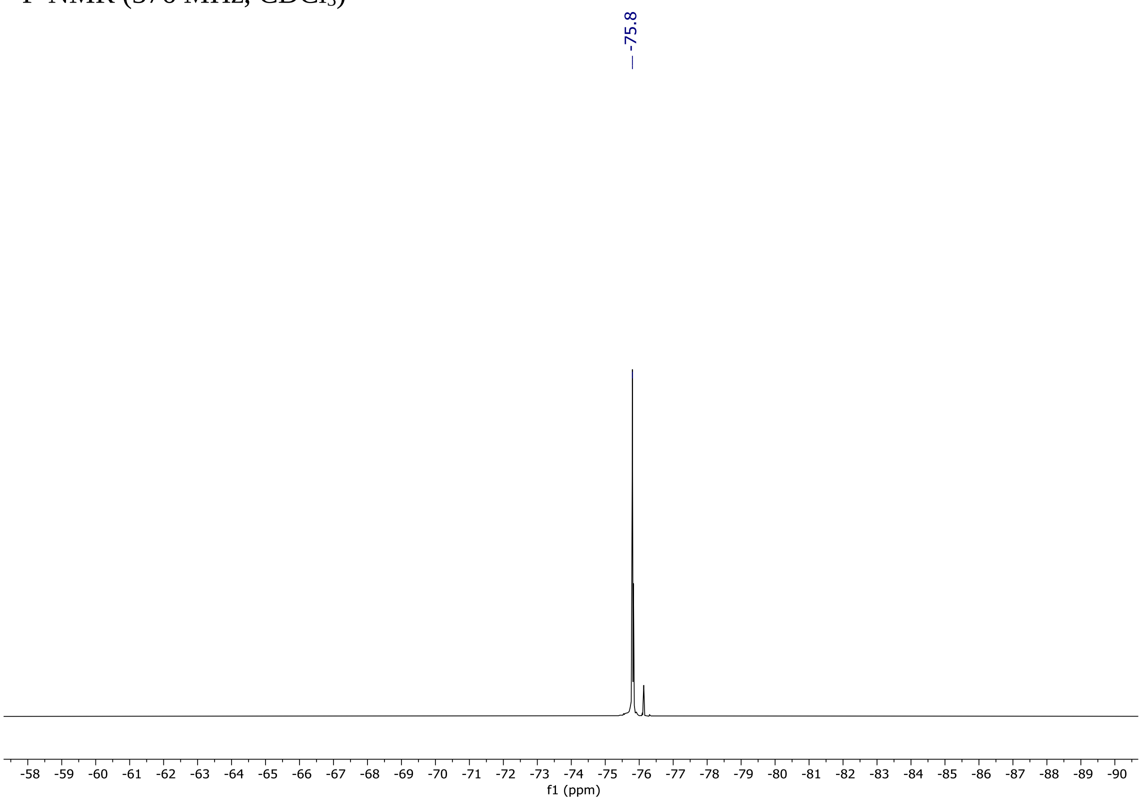

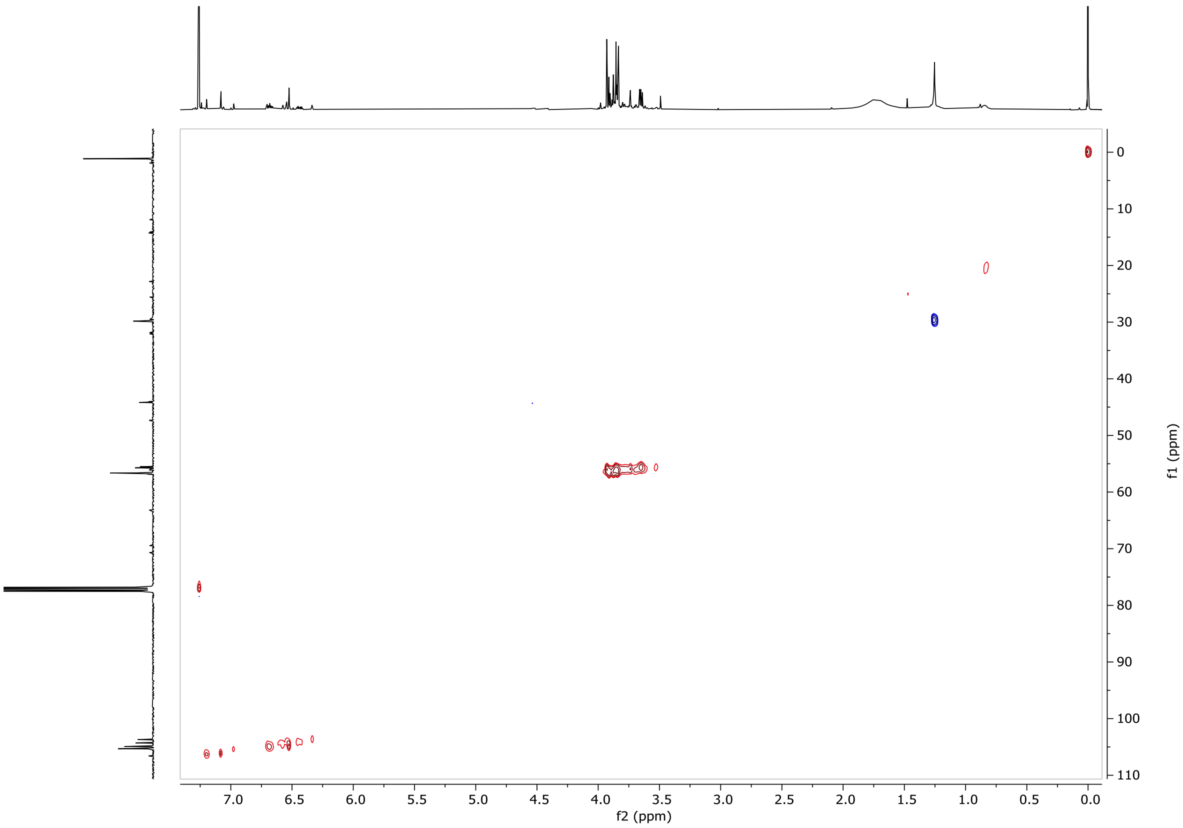

Methyl (*E*)-3- (3,5-dimethoxy-4-((2,6-dimethoxy-4-(2-(2,2,2-trifluoroacetamido)ethyl)phenyl)diazenyl)phenyl)propanoate (3p)

^{1}H-NMR (400 MHz, $CDCl_3$)

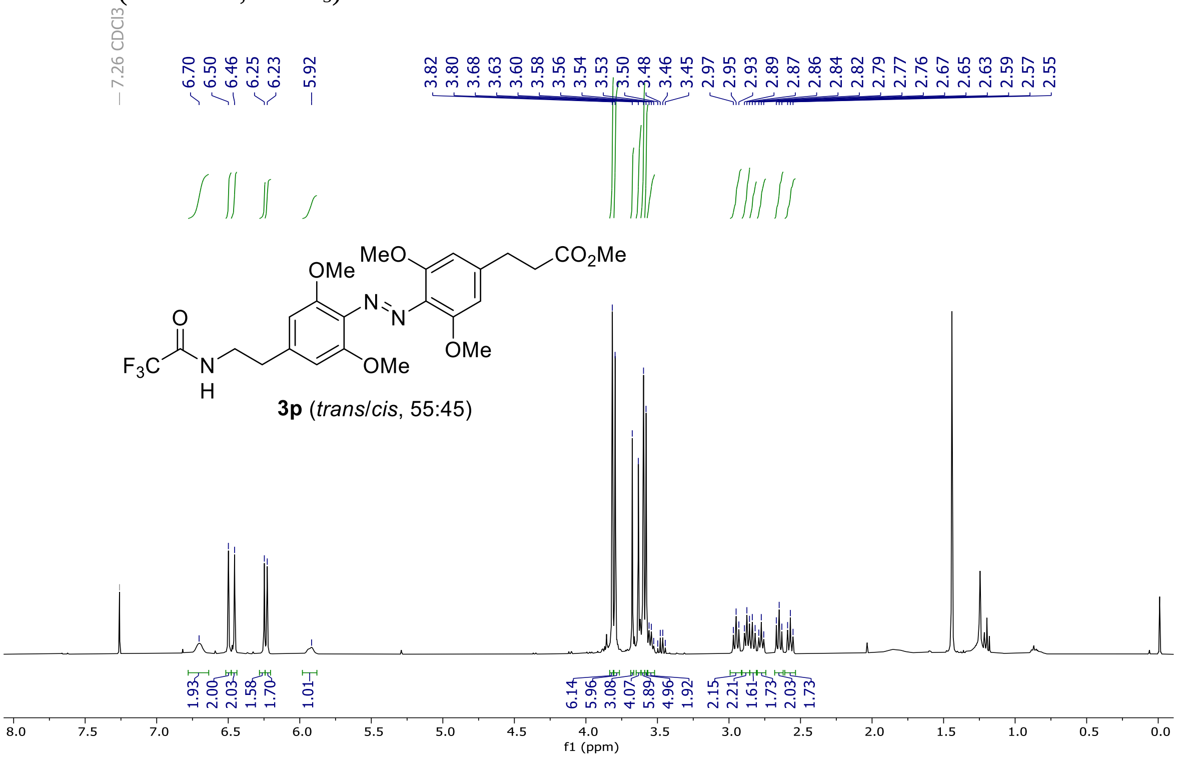

^{13}C{^{1}H}-NMR (100 MHz, $CDCl_3$)

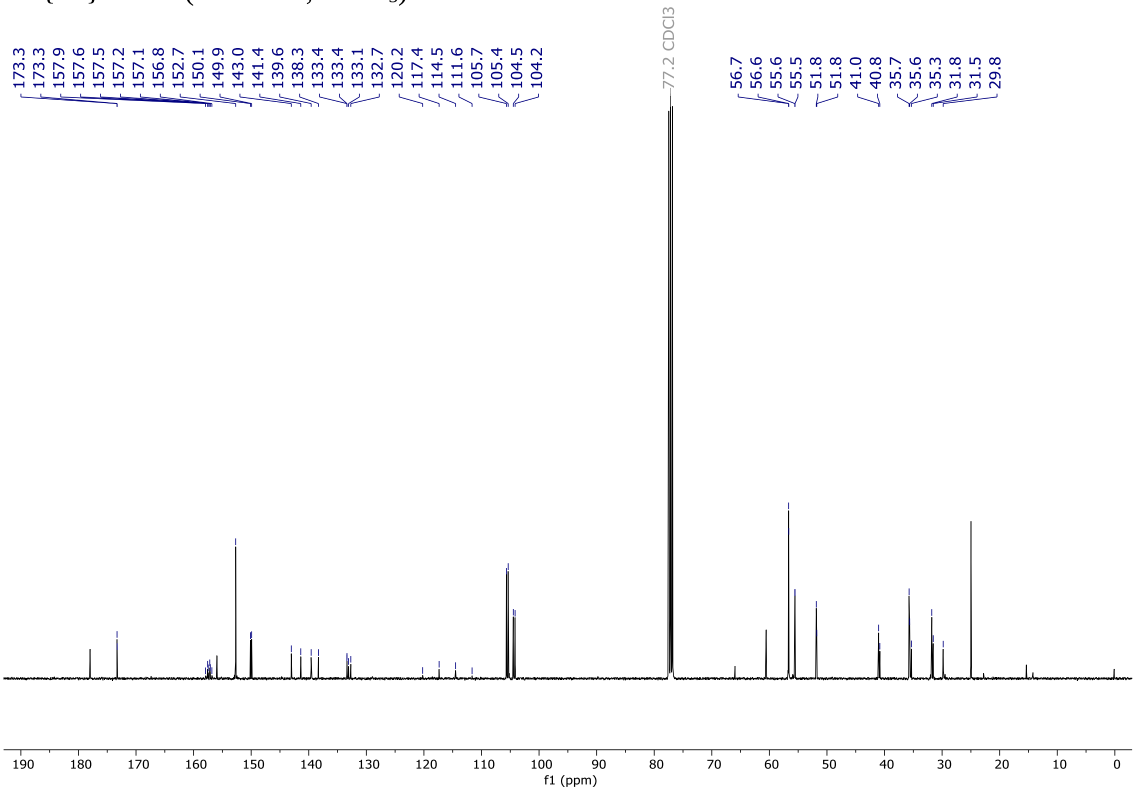

^{19}F-NMR (376 MHz, $CDCl_3$)

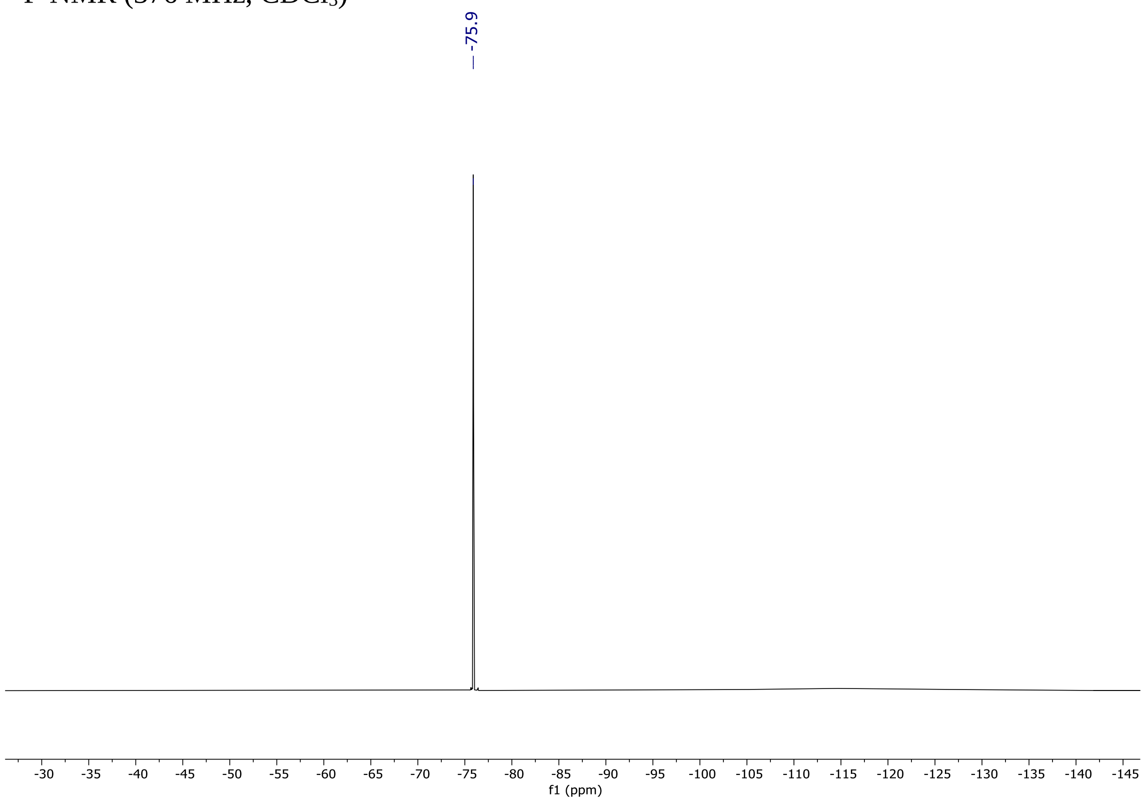

HSQC ($CDCl_3$)

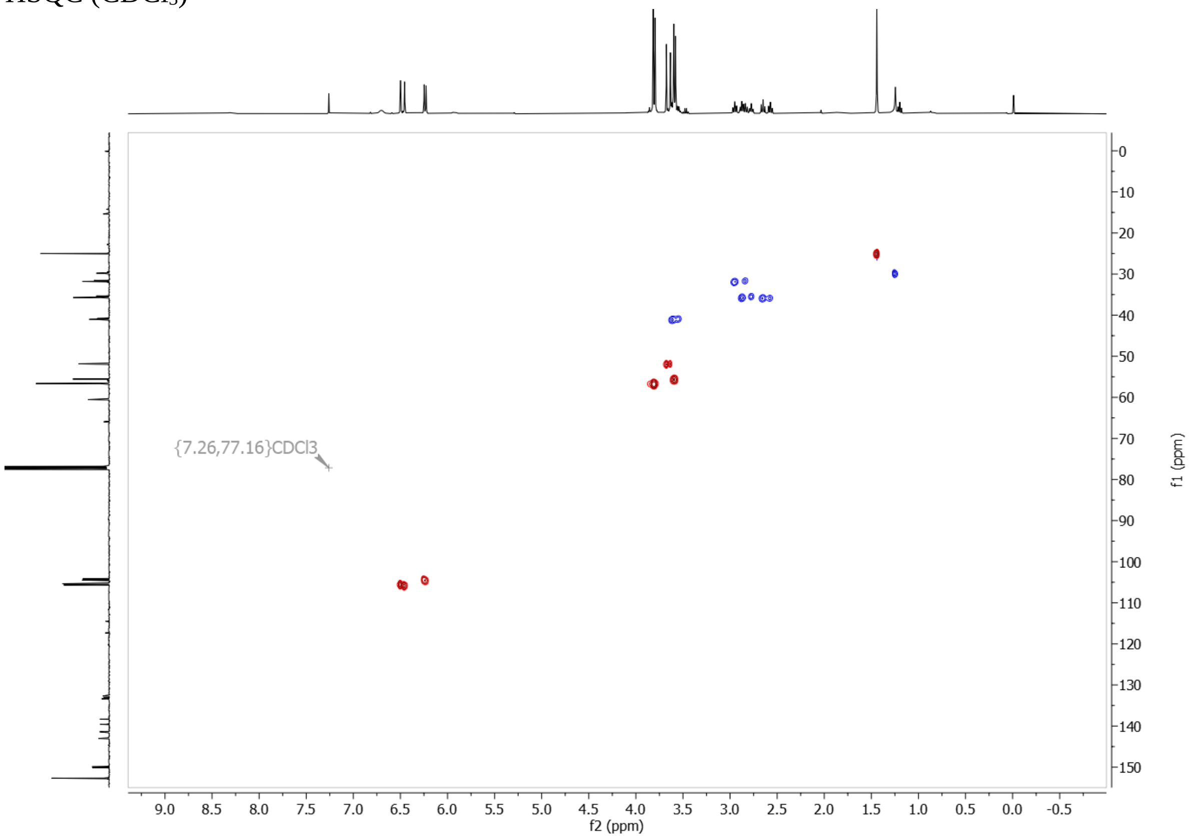

4. Solid Phase Peptide Synthesis (SPPS) Robustness Test

Manual attachment of the photoswitches bearing a carboxylic acid moiety was performed in propylene syringes fitted with a frit for filtration using RinkAmide ChemMatrix resin (functionalisation 0.54 mmol·g^{-1}) to obtain C-terminal amides upon cleavage (Scheme S5). The tetra-*ortho*-substituted azobenzenes were introduced by treating the resin with the appropriate carboxylic acid (3.0 equiv), Oxyma (3.3 equiv) and DIC (3.3 equiv) in DMF (0.81 M solution of the azobenzene carboxylic acid) during overnight gentle shaking. In order to assess the robustness of the photoswitches towards Fmoc deprotection conditions, 10 cycles of the common deprotection conditions were performed: 20% piperidine in DMF (2 × 1 min + 2 × 5 min treatments), adding to a total of 40 treatments with piperidine solution and a total exposure time of 120 min. Control experiments were performed for each photoswitch, which were bound to the resin as detailed above, but were not treated with piperidine. In order to cleave the photoswitch, the resin was treated with TFA/CH_2Cl_2 (95:5; 5 × 45 min treatments), then washed with CH_2Cl_2 (×5). The collected cleavage solution was concentrated under vacuum until all volatiles were removed. Crude amides thus obtained were analysed by HPLC-MS (ESI).

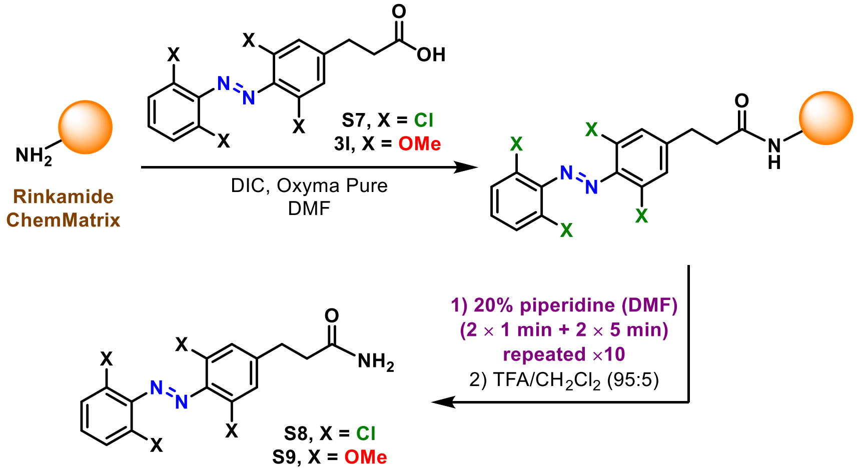

Scheme S6. Synthetic scheme for the robustness test of tetra-*ortho*-chloro- and tetra-*ortho*-methoxyazobenzenes during piperidine treatments during SPPS.

4.1. Tetra-*ortho*-Chlorinated Azobenzene Robustness Test

Tetra-ortho-chloroazobenzene carboxylic acid **S7** was bound to RinkAmide resin and either cleaved without any additional treatment of treated with 20% piperidine in DMF as detailed above. The crudes of the resulting amide (**S8**) obtained after cleavage show the formation of several new entities in the piperidine-treated sample, indicating the lack of stability of tetra-*ortho*-chloroazobenzenes during SPPS (Figure S2).

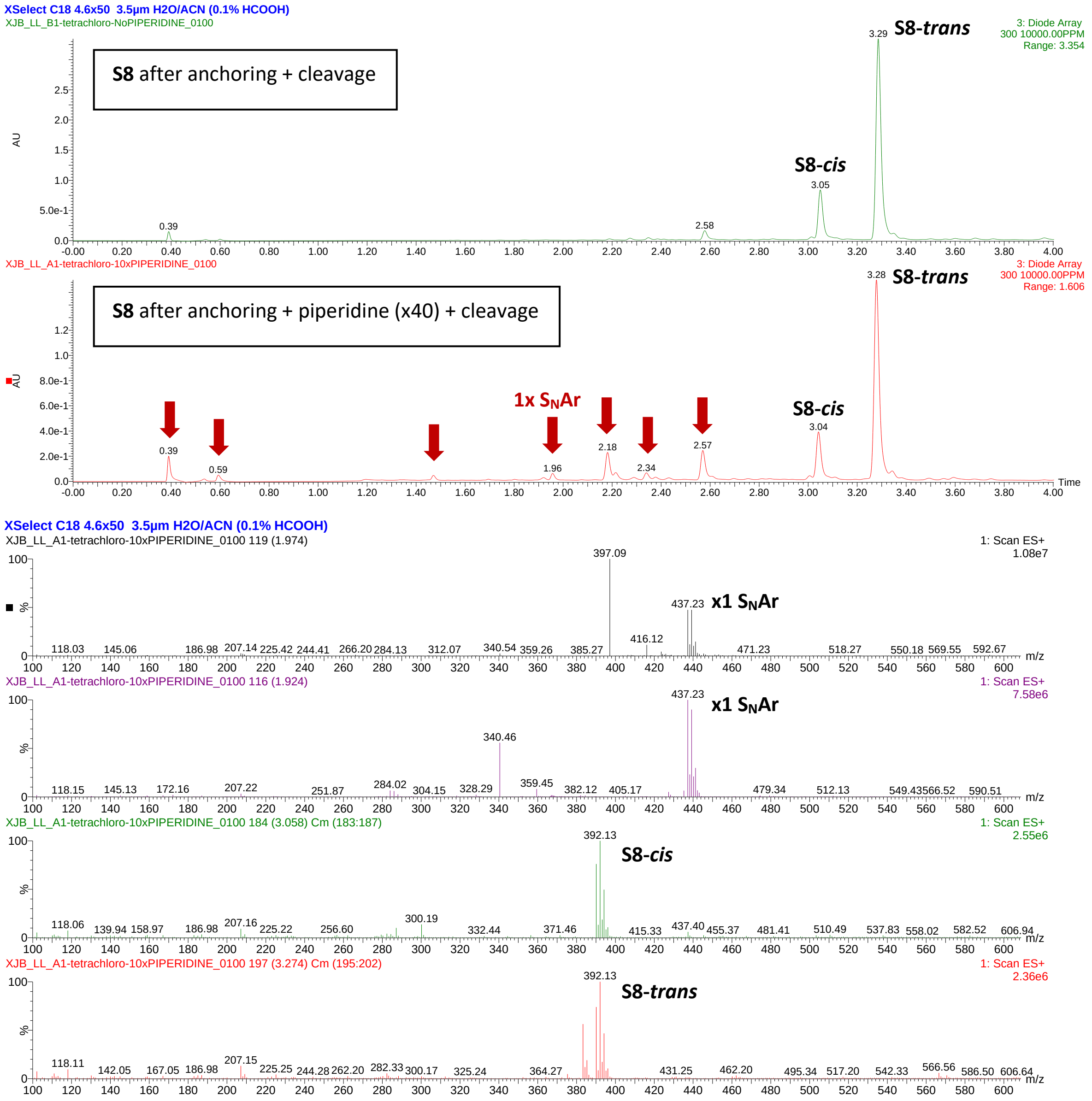

Figure S2. HPLC-MS analysis of control (top) and piperidine-treated (bottom) tetra-*ortho*-chloroazobenzene derivative **S8** after cleavage from RinkAmide resin.

4.2. Tetra-*ortho*-Methoxylated Azobenzene Robustness Test

Tetra-ortho-methoxyazobenzene carboxylic acid **3l** was bound to RinkAmide resin and either cleaved without any additional treatment of treated with 20% piperidine in DMF as detailed above. The crudes obtained after cleavage show that in both cases the chromatograms for the resulting amide (**S9**) are virtually identical, showcasing the superior stability of tetra-*ortho*-methoxyazobenzenes for SPPS (Figure S3).

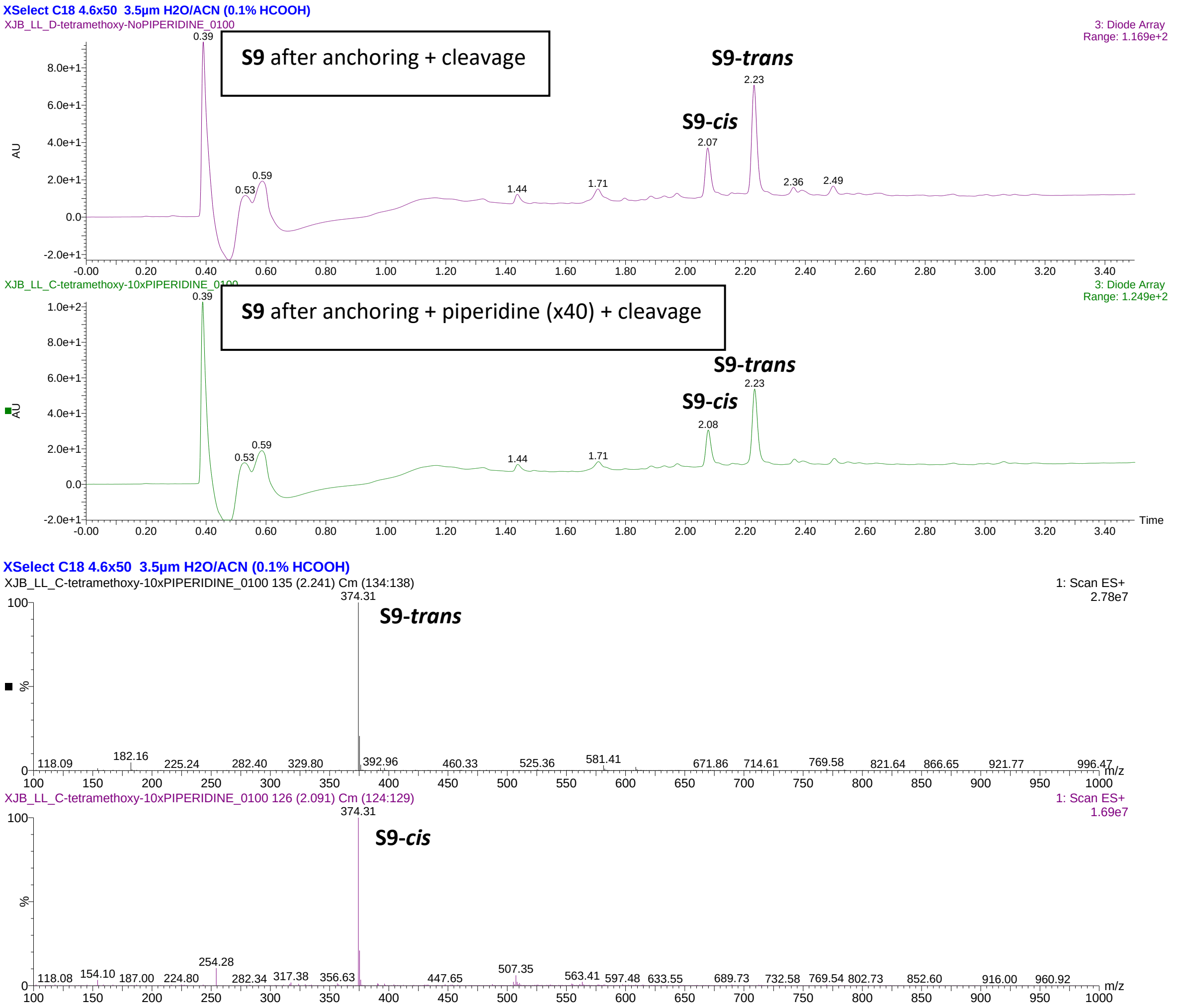

Figure S3. HPLC-MS analysis of control (top) and piperidine-treated (bottom) tetra-*ortho*-methoxyazobenzene derivative **S9** after cleavage from to RinkAmide resin.

5. Photocharacterization

5.1. UV-Vis Spectra of Photostationary States (PSS)

UV-Vis spectra of PSS of all isolated tetra-*ortho*-bromimated (Figure S4) and tetra-*ortho*-methoxylated azobenzenes (Figure S5) were recorded after irradiation at different wavelengths (405, 470, 530, 550 and 650 nm) of 50 μM solutions in DMSO in a 96-well plate.

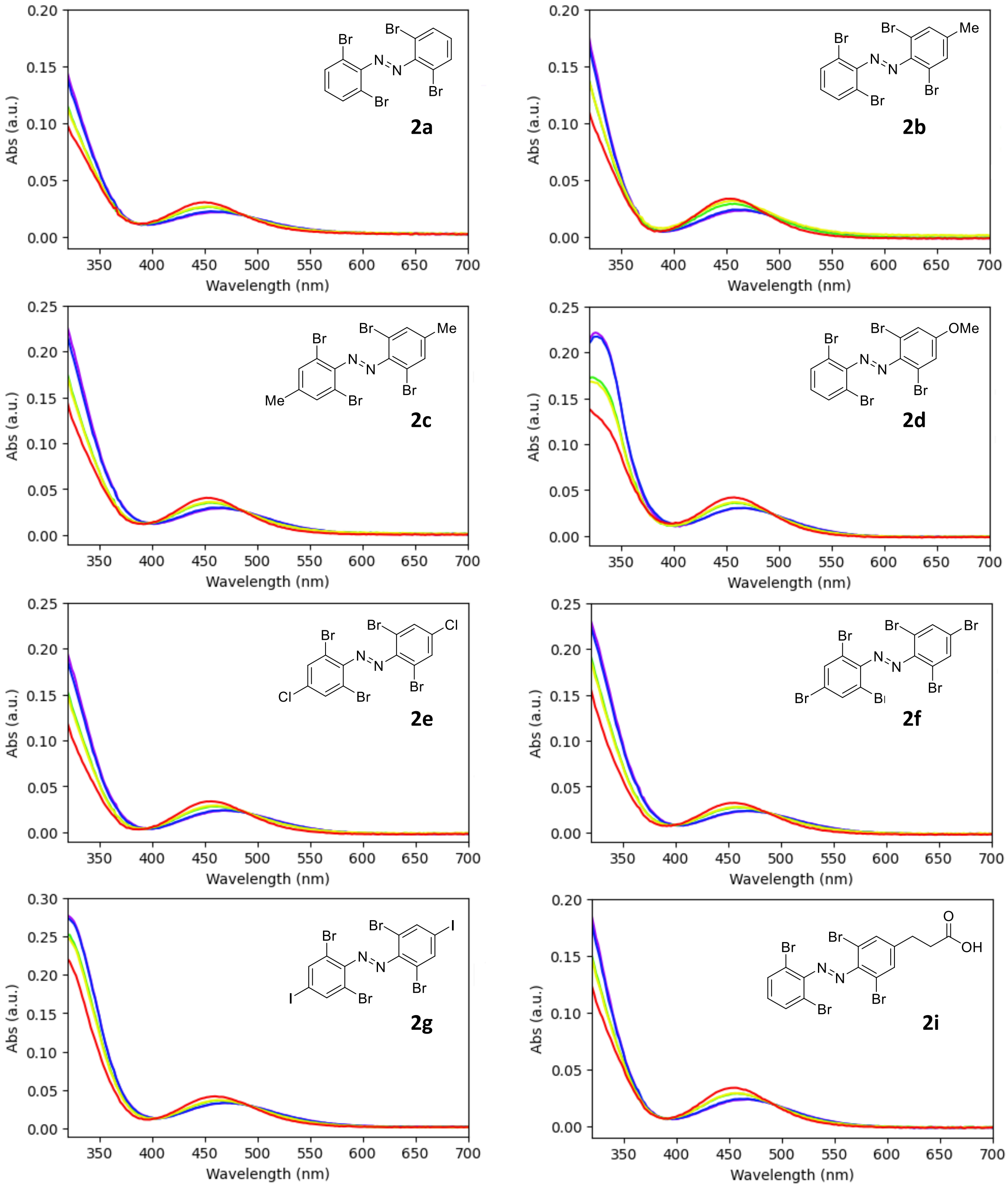

(Figure S4 continues in next page)

(Figure S4 continues from previous page)

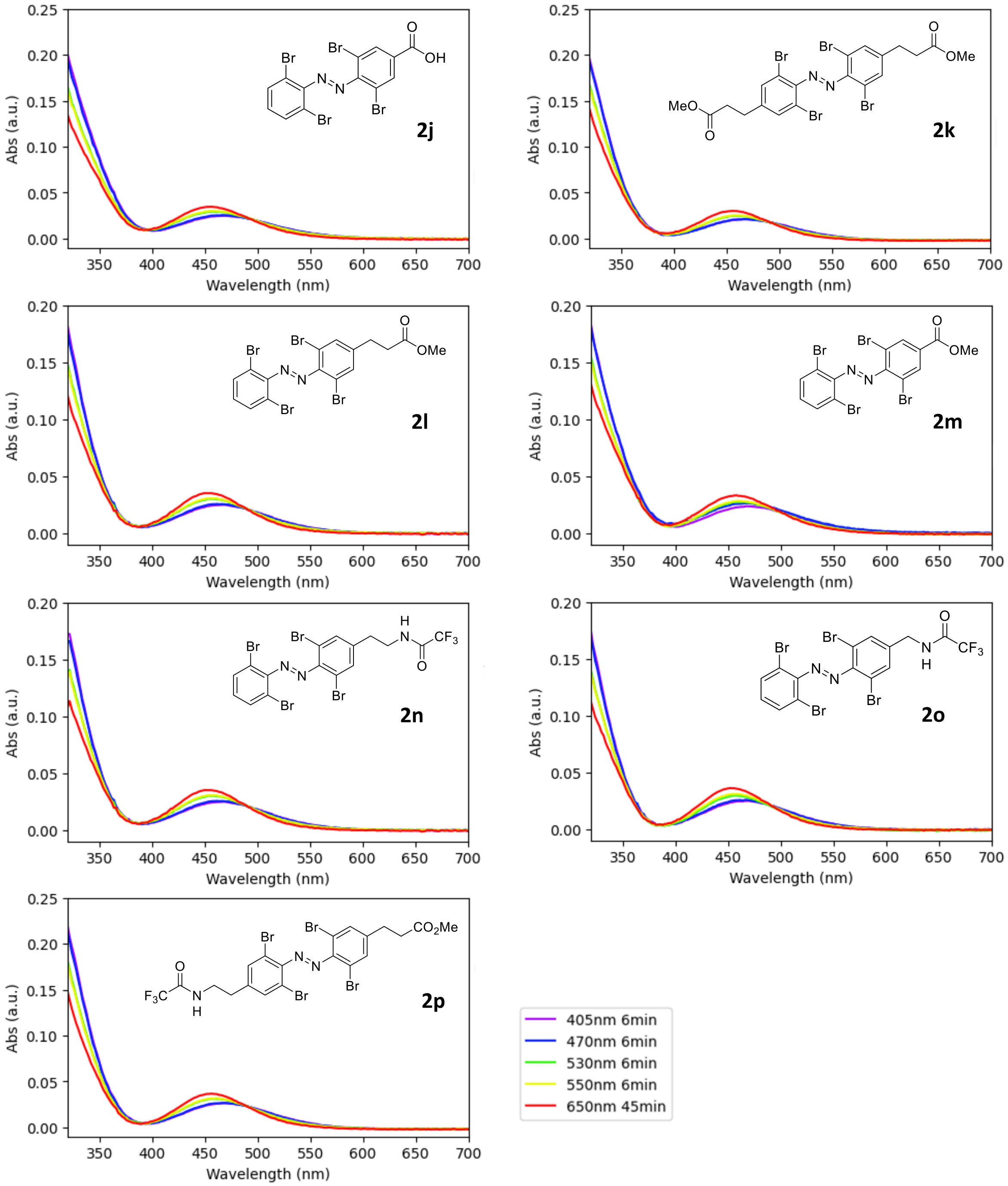

Figure S4. UV-Vis spectra of PSS of tetra-*ortho*-bromoazobenzenes (**2a-2g** and **2i-2p**).

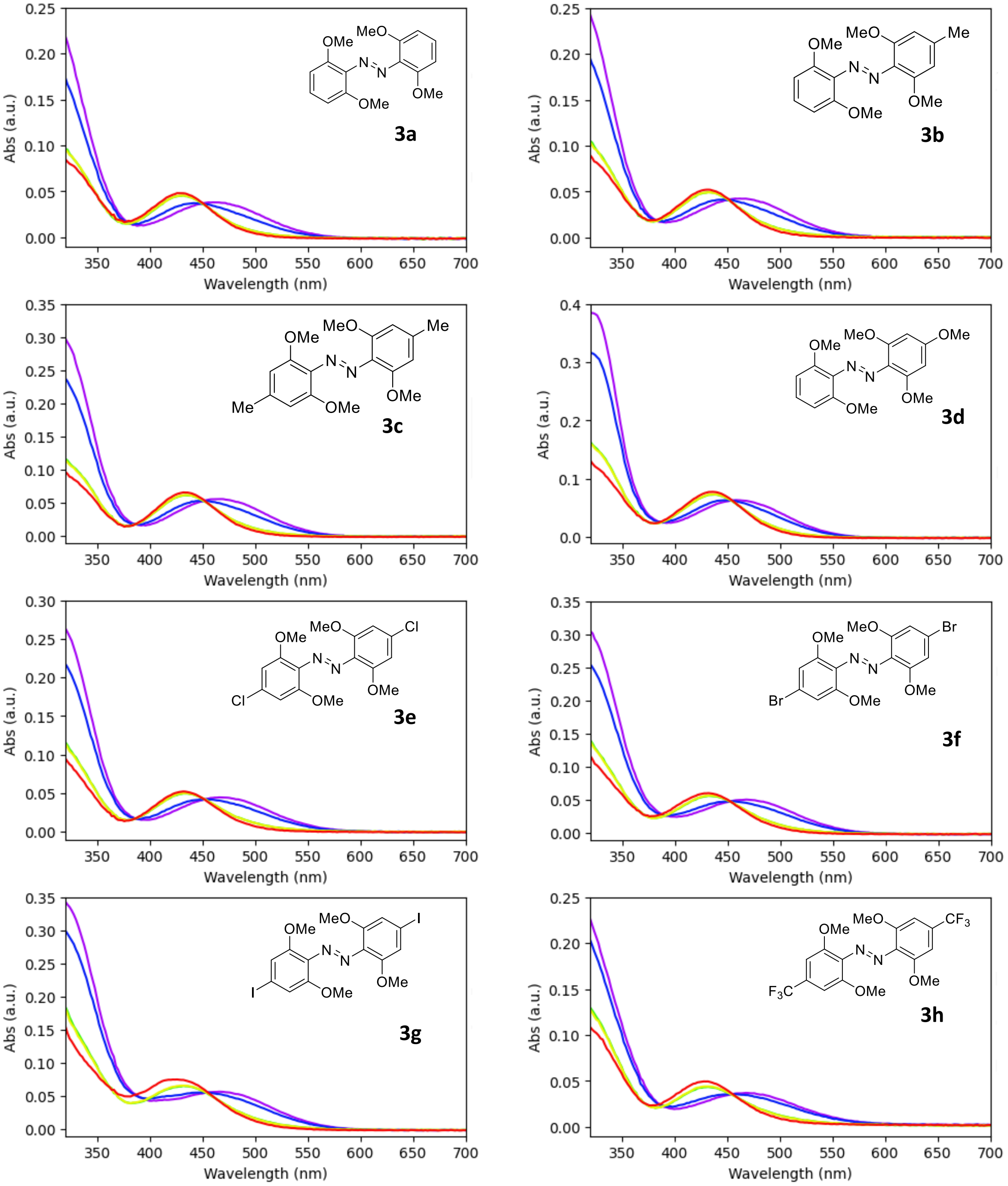

(Figure S5 continues in next page)

(Figure S5 continues from previous page)

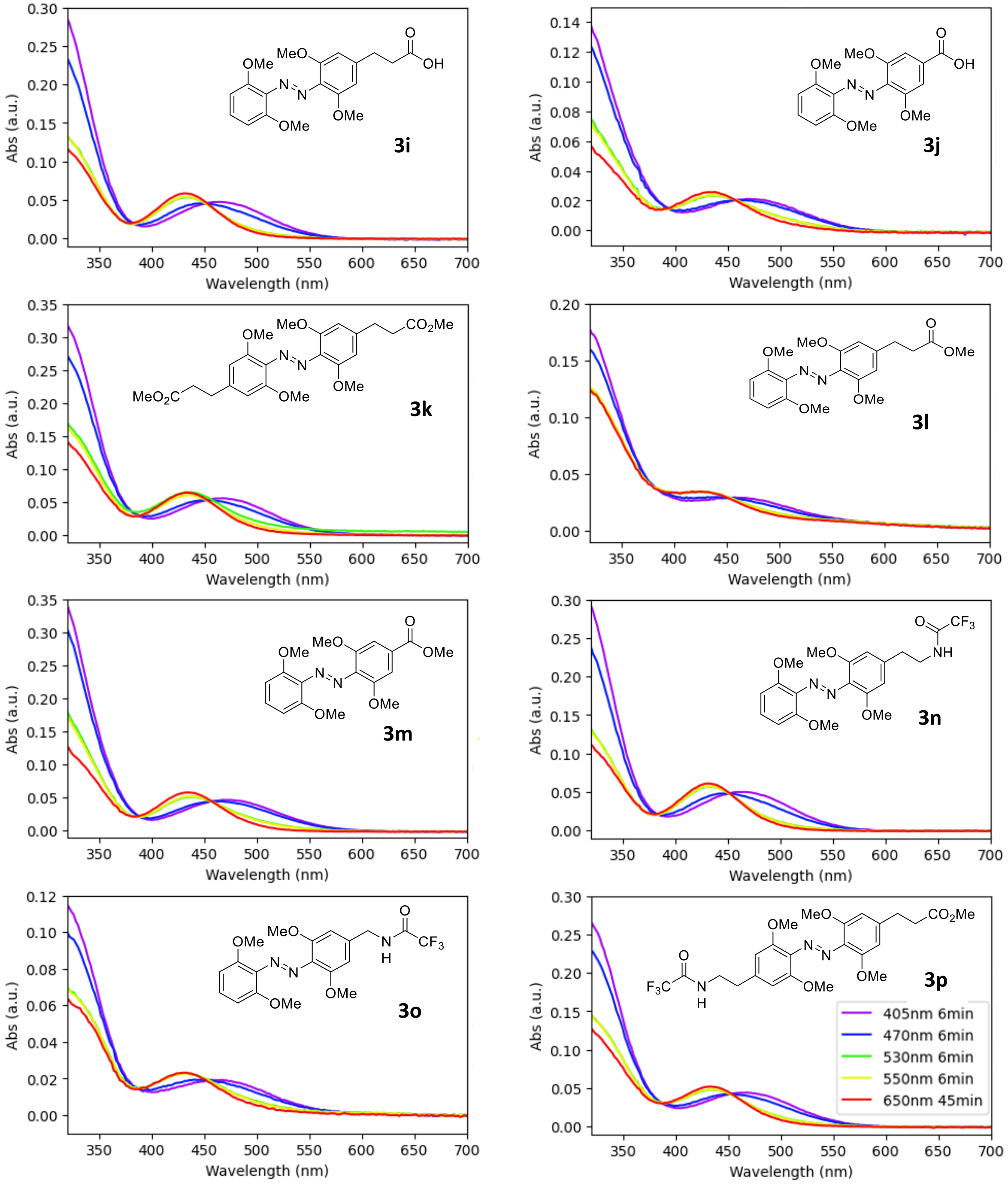

Figure S5. UV-Vis spectra of PSS of tetra-*ortho*-methoxyazobenzenes (**3a-3p**).

5.2. Isomer Ratios

Ratios of the *Z* and *E* isomers of all compounds in methanolic solution were determined by analysis by HPLC equipped with a Photo Diode Array (PDA) detector (Table S4). Samples were analysed after irradiation for 45 min at 365 nm and 650 nm and isomer ratios were determined by integrating the peaks of the corresponding isomer when reading at an isosbestic point wavelength (*ca.* 490 nm for tetra-*ortho*-bromoazobenzenes and *ca.* 450 nm for tetra-*ortho*-methoxyazobenzenes).

Table S4. Ratio of *Z* isomer of all isolated tetra-*ortho*-substituted azobenzenes in PSS after irradiation at 365 nm and 650 nm.

%*Z*	tetra-*ortho*-bromoazobenzenes		%*Z*	tetra-*ortho*-methoxyazobenzenes	
**PSS**	**365 nm**	**650 nm**	**PSS**	**365 nm**	**650 nm**
**2a**	23	78	**3a**	15	78
**2b**	22	81	**3b**	17	71
**2c**	19	75	**3c**	16	62
**2d**	25	71	**3d**	9	62
**2e**	17	70	**3e**	30	94
**2f**	14	63	**3f**	49	88
**2g**	10	60	**3g**	20	86
**2h**	N/A	N/A	**3h**	16	97
**2i**	19	82	**3i**	15	80
**2j**	7	51	**3j**	25	98
**2k**	19	77	**3k**	16	81
**2l**	35	78	**3l**	32	79
**2m**	2	28	**3m**	22	97
**2n**	20	80	**3n**	30	87
**2o**	19	77	**3o**	35	92
**2p**	19	77	**3p**	23	83
